# Metal-free intermolecular formal cycloadditions enable an orthogonal access to nitrogen heterocycles

**DOI:** 10.1038/ncomms10914

**Published:** 2016-03-15

**Authors:** Lan-Gui Xie, Supaporn Niyomchon, Antonio J. Mota, Leticia González, Nuno Maulide

**Affiliations:** 1 Institute of Organic Chemistry, University of Vienna, Währinger Strasse 38, 1090 Vienna, Austria; 2 Institute of Theoretical Chemistry, University of Vienna, Währinger Strasse 17, 1090 Vienna, Austria

## Abstract

Nitrogen-containing heteroaromatic cores are ubiquitous building blocks in organic chemistry. Herein, we present a family of metal-free intermolecular formal cycloaddition reactions that enable highly selective and orthogonal access to isoquinolines and pyrimidines at will. Applications of the products are complemented by a density functional theory mechanistic analysis that pinpoints the crucial factors responsible for the selectivity observed, including stoichiometry and the nature of the heteroalkyne.

## Abstract (web summary)

Nitrogen containing heterocycles play key roles as both bioactive compounds and synthetic building blocks in organic chemistry. Here, the authors report the synthesis of a range of heterocyclic structures via metal free cycloadditions and provide mechanistic analyses for the transformations.

## Graphical abstract

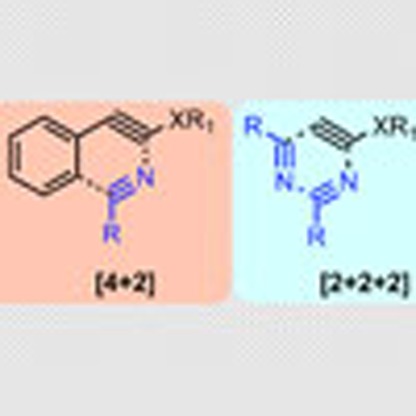

Heteroarenes constitute one of the privileged core structural motifs in organic chemistry^1^. Among them, isoquinolines and pyrimidines represent two big families in pharmaceutical agents, natural products and functional materials^2^,^3^,^4^,^5^,^6^,^7^,^8^,^9^. Therefore, continued effort is devoted to the exploration of new and efficient synthetic strategies for these backbones.

The classical strategies to prepare isoquinolines (Fig. 1a) generally focus on the crucial textbook disconnections C1-C8a (Bischler-Napieralski and Pictet-Spengler syntheses) or C4-C4a (Pomeranz-Fritsch synthesis). Recently developed routes centred on the bond-forming events N2–C3 or N2–C3/C4–C4a, employing electrophile-triggered annulation and transition metal-catalysed C–H or C–halogen bond activation, respectively^1^,^10^,^11^,^12^,^13^,^14^,^15^,^16^,^17^. A strategy relying on the simultaneous formation of N2–C3/C1–C8a is much less documented^18^.

Conversely, most of the known avenues towards pyrimidine synthesis rely on the condensation of N–C–N subunits (mostly amidines or guanidines) with 1,3-dicarbonyl derivatives or the stoichiometric activation of carbonyl moieties with triflic anhydride (Fig. 1a) (refs 19, 20, 21, 22, 23). Ynamides have recently shown to be suitable candidates for regioselective cycloaddition with nitriles in the presence of a gold catalyst, leading to 4-aminopyrimidine cores^24^. Although the reactivity of ynamides has received considerable recent attention^25^,^26^,^27^,^28^,^29^,^30^,^31^,^32^,^33^, analogous investigation of the potential enclosed in the triple bond of thioalkynes is surprisingly rare^34^,^35^,^36^,^37^, even though the resulting sulfide is a useful^38^ and versatile substituent^39^,^40^,^41^,^42^.

Herein we report a family of reactions that enable a high yielding, orthogonal access to either isoquinolines or pyrimidines at will (Fig. 1b), by Brønsted acid-mediated regioselective formal cycloaddition of ynamides and thioalkynes with nitriles (for a review of transition-metal mediated [2+2+2] cycloadditions) (ref. 43). Mechanistic studies reveal the subtle differences that are responsible for selectivity.

## Results

### Synthesis

Initial experiments involving the reaction of ynamide **1a** with various Brønsted acids in the presence of varying amounts of acetonitrile led to moderate yields of isoquinoline **3aa**. After optimization of conditions (see Supplementary Table 1 for details), we found that essentially equimolar amounts of **1a**, **2a** and TfOH in dichloroethane as solvent sufficed to enable preparation of **3aa** in 89% yield (for a discussion of stoichiometry in these reactions, *vide infra*).

**Figure 2:**
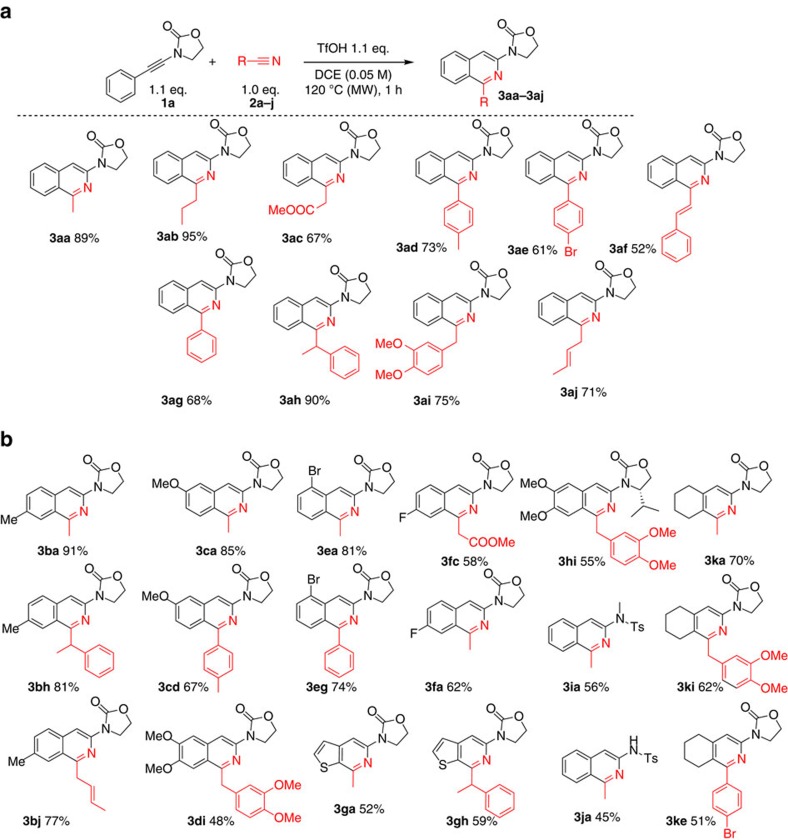
Scope of isoquinoline synthesis. (**a**) Scope of nitriles and (**b**) Scope of ynamides for the synthesis of isoquinolines. Yields are for isolated products.

Holding suitable conditions in hand, we then examined several nitriles **2a-j** under the optimized conditions. As shown in Fig. 2a, this direct formal cycloaddition is applicable to a broad range of substrates, generally affording good to excellent yields of isoquinoline products. Remarkably, alkyl nitriles bearing functional groups such as an ester (**2c**), aryl rings (**2h** and **2i**) or C–C double bonds (**2j**) are compatible with the reaction conditions. It is worth mentioning that the isolated double bond in product **3aj** does not migrate into conjugation with the isoquinoline ring under these conditions. Aryl nitriles (**2d**–**g**) and α,β-unsaturated nitrile **2f** are also viable partners delivering the corresponding substituted isoquinoline products in good to very good yields.

Subsequently, a broad range of ynamides were submitted to this protocol (Fig. 2b). In the event, both electron-donating (**1b**–**d**) and -withdrawing (**1f**) substituents were tolerated on the ynamide partner, leading to smooth isoquinoline assembly in good yields. Halogenated ynamides (**1e**–**f**) were also amenable to this reaction, delivering isoquinoline products ripe for subsequent divergent functionalization. Thienopyridine skeletons could be obtained in reasonable yields (**3ga** and **3gh**). Interestingly, *N*-tosyl-*N*-benzyl ynamide (**1j**) directly generated the corresponding debenzylated product: the tosyl-protected, pharmacologically relevant 3-aminoisoquinoline (**3ja**) (refs 44, 45). Moreover, the use of an alkenyl-substituted ynamide (**1k**) led to the annulated pyridine product (**3ke**).

**Figure 3:**
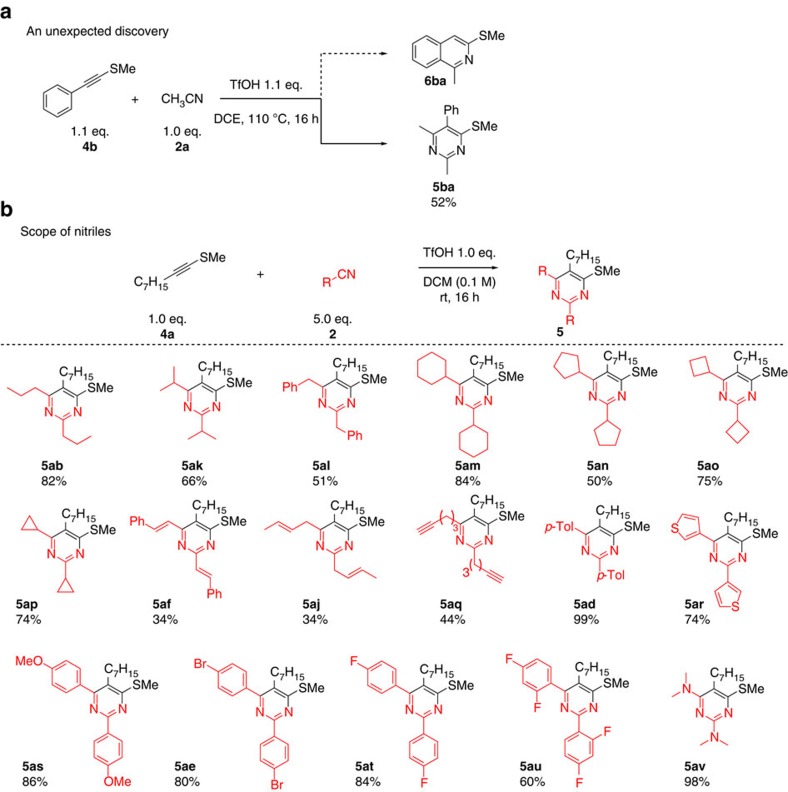
Synthesis of pyrimidine. (**a**) Unexpected synthesis of pyrimidine **5ba**. (**b**) Scope of nitriles in the synthesis of pyrimidines **5**.

After this initial success, we hypothesized that other heteroatom-substituted alkynes might prove amenable to a similar modular assembly of isoquinolines. In particular, we were drawn to the use of thioalkynes such as **4b**, with the expectation of obtaining an (alkylthio)-isoquinoline **6ba** where the sulfur residue could serve as a useful synthetic handle (Fig. 3a).

Much to our surprise, treatment of **4b** with acetonitrile **2a** under conditions identical to those employed previously led exclusively to the pyrimidine **5ba** in 52% yield (Fig. 3a). Remarkably, product **5ba** is the result of a formal, regioselective cycloaddition of one molecule of **4b** with two molecules of **2a**. This dramatic shift in product selectivity between ynamides and thioalkynes eventually presented us with a versatile cycloaddition route towards pyrimidines. Reaction optimization showed that this transformation proceeds most effectively at room temperature in the presence of an excess of acetonitrile (see Supplementary Table 2 for details).

Figure 3b depicts the full scope of nitriles **2b**–**v** compatible with this metal-free pyrimidine synthesis. Secondary aliphatic (**2k**) and alicyclic (**2m**-**2p**) carbonitriles smoothly coupled with thioalkyne **4a** under the reaction conditions. This formal cycloaddition was also tolerant of nitriles bearing triple (**2q**) and double bonds (**2j**), including conjugated olefins (**2f**). Both electron-rich (**2d** and **2s**) and electron-deficient (**2t** and **2u**) substituted benzonitriles could be employed, providing the desired pyrimidine products in good to excellent yields. It is worth noting that heteroarylnitriles such as 3-cyanothiophene (**2r**) were also tolerated. The possibility of using dimethylcyanamide (**2v**), delivering an aminated pyrimidine in excellent yield, further highlights the generality of this synthetic method. Pyrimidine **5ae** yielded crystals suitable for X-ray diffraction analysis, unambiguously confirming its structure (see Supplementary Fig. 64 and Supplementary Tables 4 and 5 for details).

**Figure 4:**
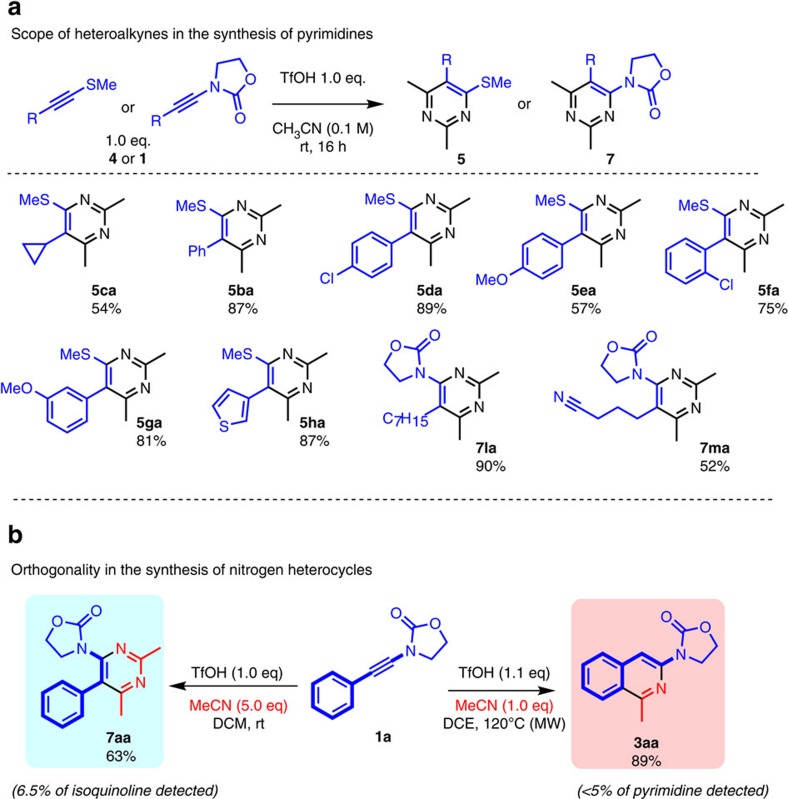
Scope and orthogonality of heterocycles. (**a**) Scope of heteroalkynes for the synthesis of pyrimidines **5** or **7**. (**b**) Orthogonality in the synthesis of isoquinolines or pyrimidines from ynamides. See Supplementary Figs 62 and 63 for details.

Further studies focused on the scope of heteroalkynes for this pyrimidine synthesis (Fig. 4a). We were pleased to find that a cyclopropyl substituent (**4c**) was tolerated, as a cyclopropyl appended to a pyrimidine ring is a common feature in drug-like, biologically active cores^46^,^47^,^48^. Both electron-rich (**4e** and **4g**) and electron-poor (**4d** and **4f**) arylalkynes afforded the corresponding pyrimidine products in good yields. Furthermore, considerable flexibility can be exerted, concerning the location of substituents on the aryl ring (**4d**–**g**).

Strikingly, we found that 4-aminosubstituted pyrimidines can also be obtained by exposing ynamides (**1l**, **1a** and **1m**) to the standard conditions developed for pyrimidine synthesis. A distal nitrile group carried by the ynamide partner could be successfully introduced into the pyrimidine product (**7ma**). Remarkably, when phenyl-substituted ynamide **1a** was submitted to these conditions, a 4-amino-5-aryl pyrimidine product (**7aa**) was obtained in good yield (Fig. 4b). Together with the reactions described previously (*cf*. Figures 2–3), these results offer an entirely new orthogonal access to either isoquinoline or pyrimidine motifs at will, while unifying this novel, powerful family of formal cycloaddition reactions.

### Density functional theory study

**Figure 5:**
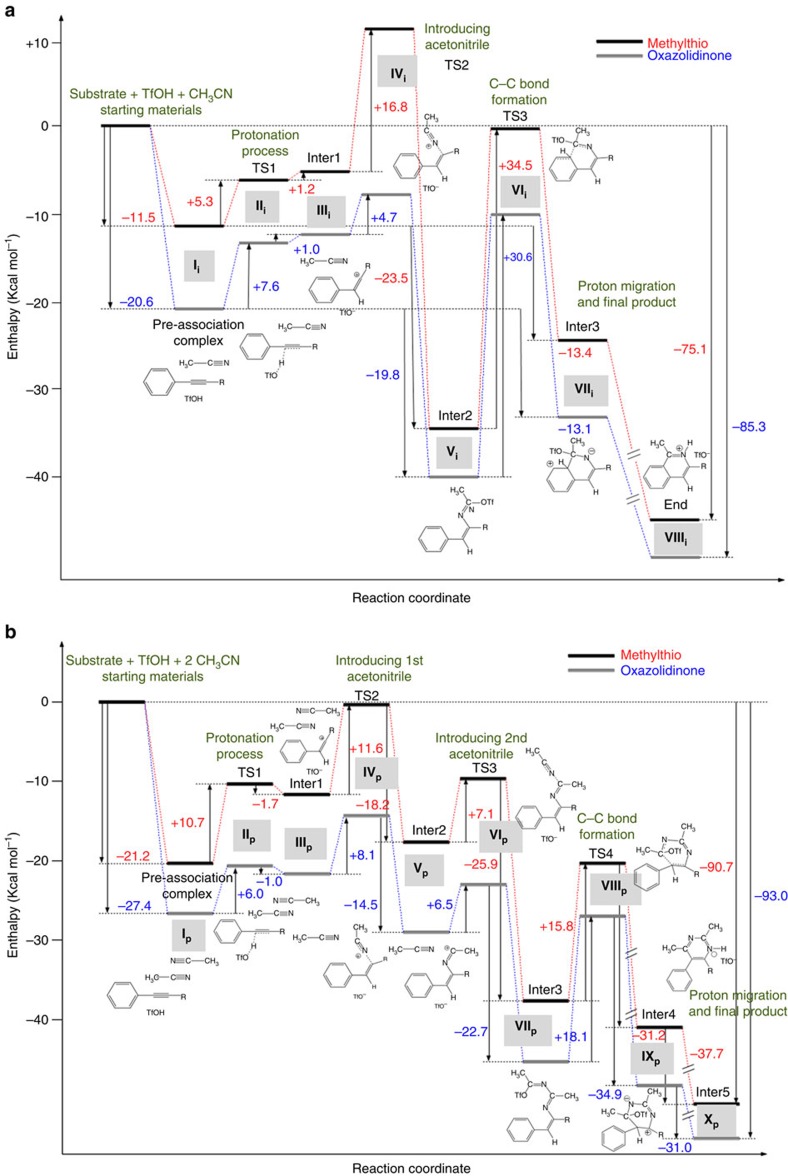
Energy profiles. Isoquinoline (**a**, up, i subscript in roman numerals) and pyrimidine (**b**, down, p subscript in roman numerals) formation for the oxazolidinone (blue) and methylthio (red) derivatives. The corresponding three-dimensional structure sequence is exemplified for the methylthio derivative, for both **a** and **b** pathways, in Supplementary Figs 66 and 67, respectively

We approached the mechanistic study of this reaction performing density functional theory (DFT) calculations of two reaction manifolds: the first leading to isoquinoline products (by modelling the entire pathway introducing a single acetonitrile molecule, see Fig. 5a) and the second leading to pyrimidine adducts (by computing the mechanism with two acetonitrile molecules, see Fig. 5b). The first question that arises is what occurs when all these species are in the presence of the TfOH promoter, as there are many potential protonation sites. DFT calculations (see Computational details in the Supplementary Figs 65–67) show that protonation would take place preferably on the heteroalkyne partner. Indeed, calculated transition states for the oxazolidinone (+7.6 and +6.0) and methylthio (+5.3 and +10.7) derivatives (in the presence of either one or two acetonitrile molecules, **II**_**i**_ and **II**_**p**_, respectively) are much lower than the acetonitrile protonation (+17.0 Kcal mol^−1^). Furthermore, we confirmed that this protonation takes place regioselectively β- to the heteroatom as anticipated, leading to either a keteniminium **III**_**i**_ or ketenethionium **III**_**p**_ species, as the TfO^−^ anion is stabilized by acetonitrile (which in these reactions coincides with the nucleophilic species). In the second mechanistic step, a nucleophilic attack by acetonitrile takes place stereoselectively from the face opposite to the β-proton due to shielding by TfO^−^ (see **IV**_**i**_ and **IV**_**p**_ in Fig. 5). In fact, in both cases the introduction of acetonitrile, giving respectively **V**_**i**_ and **V**_**p**_, is more stable than the corresponding TfO^−^ bonded derivative by 6.1 and 7.7 Kcal mol^−1^ for the oxazolidinone and methylthio derivatives, respectively. Interestingly, and very important for the reaction outcome, in the absence of acetonitrile the TfO^−^ species readily adds to the positively charged intermediate effectively blocking further reaction with acetonitrile. Moreover, we verified that nucleophilic attack by acetonitrile can only take place after the first protonation event as the highest occupied molecular orbital of acetonitrile (−0.3264 H) and the neutral ynamide's lowest unoccupied molecular orbital (−0.0242 H) are energetically too far apart. The protonation process, however, results in an alkyne-centred lowest unoccupied molecular orbital turned by 90° at −0.2443 H, whereas the highest occupied molecular orbital of the TfO^−^ counteranion lies at −0.0742 H (see Supplementary Figs 65 in the Computational details section of the Supplementary Information). A similar trend is observed for the methylthio derivative. These two first processes (protonation+nucleophilic attack of acetonitrile) are common to both pathways. At this juncture, we can separately analyse the mechanisms leading to the isoquinoline and the pyrimidine scaffolds.

### Isoquinoline formation

Following the addition of acetonitrile, the TfO^−^ anion immediately adds to the resulting carbocation (as the former lost its prior stabilization by acetonitrile) delivering a neutral and highly stable imino-triflate **V**_**i**_. The last step consists of a Friedel–Crafts-like cyclization, **VII**_**i**_, with further elimination of TfOH giving rise to the isoquinoline skeleton (**VIII**_**i**_). This final addition process is characterized by high energy-transition states: +30.6 and +34.5 Kcal mol^−1^, respectively, for the oxazolidinone and methylthio derivatives (**VI**_**i**_; Fig. 5a). This is why heating is necessary in this case. The higher enthalpic barrier for the methylthio derivative stands in agreement with the experimental findings, as no isoquinoline product is observed for this derivative. In addition, nuclear magnetic resonance studies carried out with the starting heteroalkynes in the presence of TfOH suggest that the thermal stability of the methylthio-derivative is notably low. This could be a determining factor towards the experimental observations.

### Pyrimidine formation

In this case, with two acetonitrile molecules (used for simplicity of the model, although the experimentally optimized molar ratio is higher), a different situation arises as a second addition becomes a more probable event. In fact, this process takes place through low energy-transition states (+6.5 and +7.1 Kcal mol^−1^, respectively, for the oxazolidinone and methylthio derivatives), **VI**_**p**_, giving rise to rather stable intermediates (**VII**_**p**_; see Fig. 5b). Once the second molecule is added, the system could conceivably undergo a polymerization process with continued further addition of more acetonitrile molecules to the newly generated carbocationic species (+0.546 and +0.529 e^−^, respectively, for the oxazolidinone and methylthio derivatives). Instead, the negatively charged (–0.208 and –0.210 e^−^, for the oxazolidinone and methylthio derivatives, respectively) β-carbon atom can attack (**VIII**_**p**_) the newly generated carbocation to form an entropically favoured, six-membered pyrimidine ring (after a very exothermic re-aromatization promoted by TfO^−^, **IX**_**p**_→**X**_**p**_). This is the driving force for pyrimidine formation.

**Figure 6:**
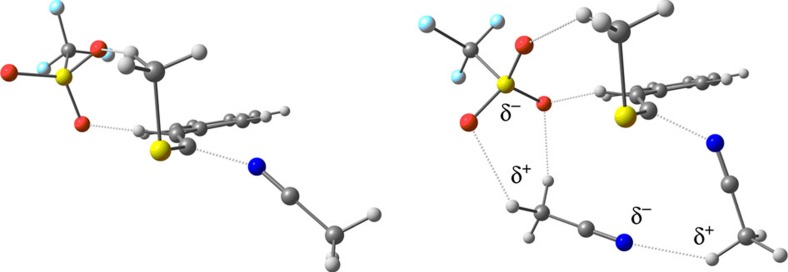
Transition states for the first acetonitrile addition. Isoquinoline (left) and pyrimidine (right) pathways, the latter presenting additional cyclic, electrostatic stabilizing interactions.

A comparative analysis of both calculated mechanisms reveals at a glance that those involving the oxazolidinone derivative proceed generally with lower energies than the thioalkyne one. The main reason for that is the greater stabilization of the positive charge in the former case. In addition, although pathways for formation of either heterocycle could exist for both heteroalkynes, the pathway for isoquinoline formation through the thioalkyne derivative (Fig. 5a) has a prohibitive energy barrier when considering the addition of a single acetonitrile molecule. This barrier is much lower in the case of pyrimidine formation (Fig. 5b). This is due to the fact that in the latter case, there is a significant stabilization of the corresponding transition state introduced by the presence of the second acetonitrile molecule. Yet, the transition state for the first acetonitrile attack is 5.2 Kcal mol^−1^ higher in the case of isoquinoline formation. In this value, 3.2 Kcal mol^−1^ are purely due to the stabilization offered by the second acetonitrile molecule, as calculations made considering cationic structures (namely just the substrate and the acetonitrile molecule(s) without the TfO^−^ species) show this same energy difference. Therefore, the remaining 2 Kcal mol^−1^ should derive from stabilization by the counteranion (which is present in our mechanistic studies) through cooperative cyclic δ^+^·δ^−^ interactions between the different molecular units (see Fig. 6). In fact, we expect this transition state to be very low-lying, taking into account more solvent molecules. The preceding mechanistic analysis also permits a rationalization of why isoquinoline synthesis (formal [4+2]; (refs 49, 50, 51, 52)) requires high temperatures (highest energy barriers), whereas pyrimidine formation (formal [2+2+2]) typically occurs at room temperature.

In both cases, stoichiometry plays a crucial role and imposes the final result, as both pathways are irreversible. In the presence of several molecules of acetonitrile (Fig. 5b), the corresponding transition state for a real [2+2+2] approximation is either transition state **IV**_**p**_ or **VI**_**p**_, depending on the initial geometry conditions. These transition state also appear in a sequential pathway (as described in Fig. 5), thus indicating a natural direction for this molecular set. In the case of isoquinoline formation (Fig. 5a), the reaction of the unique acetonitrile molecule (imposed by stoichiometry) creates a positive charge that is readily neutralized by the negatively charged triflate present in the surrounding (as a remnant from the initial protonation event). The latter reaction should be faster than the time required for another acetonitrile molecule to approach, to follow pathway B. Once triflate blocks this position, a quite stable intermediate (**V**_**i**_) is formed and no additional acetonitrile molecules can be added.

It is noteworthy that although all the computed reaction pathways would only require a catalytic amount of TfOH to proceed (owing to its regeneration on aromatization, *vide supra*), the most stable final product in either pathway is the corresponding nitrogen-protonated heterocycle (readily converted into the experimentally isolated products following basic workup). This neatly accommodates the experimental need for stoichiometric amounts of acid, to obtain high yields.

### Further studies

**Figure 7:**
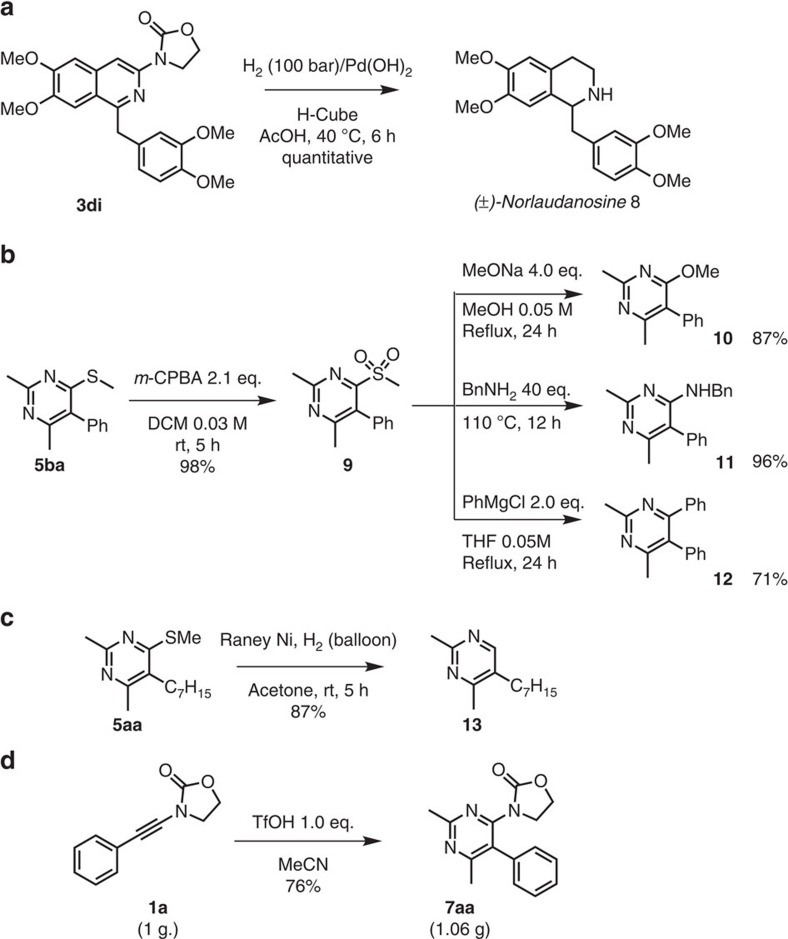
Synthetic application and modification. (**a**) Preparation of norlaudanosine **8** by hydrogenation of **3di**. (**b**) Transformations of compound **5ba**. (**c**) Reductive desulfurization of compound **5aa**. (**d**) Pyrimidine **7aa** can be prepared in gram scale.

Given the prevalence of isoquinoline motifs in the core of bioactive molecules^53^,^54^,^55^, we were eager to showcase the synthetic utility of our products (Fig. 7). The tetramethoxy adduct **3di**, carrying two electronically differentiated fused rings in its isoquinoline system, could be hydrogenated in acetic acid to deliver (±)-norlaudanosine **8** (Fig. 7a). Removal of the oxazolidinone takes place under these conditions analogously to previous work by Glorius *et al.*^56^ on related pyridines and quinolones^57^. It is noteworthy that the use of a chiral 2-oxazolidone analogue, as in **3hi**, enabled the hydrogenation to proceed with some level of asymmetric induction (55% *e.e.*). See Supplementary Fig. 60 for more detail.

The (methylthio)pyrimidine **5ba** could be easily oxidized to the corresponding methylsulfonyl derivative **9**, in which the methylsulfonyl group is available for substitution. As shown in Fig. 7b, this can be achieved by the action of alcohols, amines or Grignard reagents, delivering substituted pyrimidines **10–12** in very good to excellent yields.

In addition, Raney-Ni-mediated hydrogenation of **5aa** smoothly excises the sulfide residue to afford the 2,5,6-trisubstituted pyrimidine **13** in 87% yield (Fig. 7c). These simple transformations outline the versatility and usefulness of the methods reported herein. Moreover, the reaction can be readily carried out in gram scale (Fig. 7d).

## Discussion

A family of reactions selectively leading to isoquinoline and pyrimidine motifs has been developed, by Brønsted acid-promoted regioselective merger of alkynes and nitriles. These methodologies benefit from the strategic use of readily available nitriles as the C–N sources. Most importantly, the orthogonality of the methods enables the preparation of either family of heterocycles from the same starting materials. The practicality of these metal-free formal cycloadditions is illustrated by the large scope of alkynes and nitriles that can be employed. DFT calculations reveal the crucial role of TfOH and the reaction stoichiometry in these processes. With one equivalent of acetonitrile, the preferred pathway leads to isoquinoline products through a Friedel–Crafts-like process; with larger amounts of nitrile, a second addition is allowed *en route* to the formation of a pyrimidine derivative. Furthermore, subtle differences between the classes of heteroalkynes employed control which products can be formed. We believe that the simple yet powerful heterocycle syntheses presented here will be eagerly adopted into the repertoire of synthetic chemistry.

## Methods

Full experimental details, characterization of compounds, Cartesian coordinates and energies of all the structures appearing in Supplementary Figs 66 and 67, and computational details can be found in the Supplementary Information (Supplementary Figs 1–67 and Supplementary Methods).

## Additional information

**Accession codes:** The X-ray crystallographic coordinates for structures reported in this study have been deposited at the Cambridge Crystallographic Data Centre (CCDC), under deposition numbers CCDC 1423496. These data can be obtained free of charge from The Cambridge Crystallographic Data Centre via www.ccdc.cam.ac.uk/data_request/cif.

**How to cite this article:** Xie, L.-G. *et al.* Metal-free intermolecular formal cycloadditions enable an orthogonal access to nitrogen heterocycles. *Nat. Commun.* 7:10914 doi: 10.1038/ncomms10914 (2016).

## Supplementary Material

Supplementary Supplementary Figures 1-67, Supplementary Tables 1-5, Supplementary Methods and Supplementary References

## Figures and Tables

**Figure 1:**
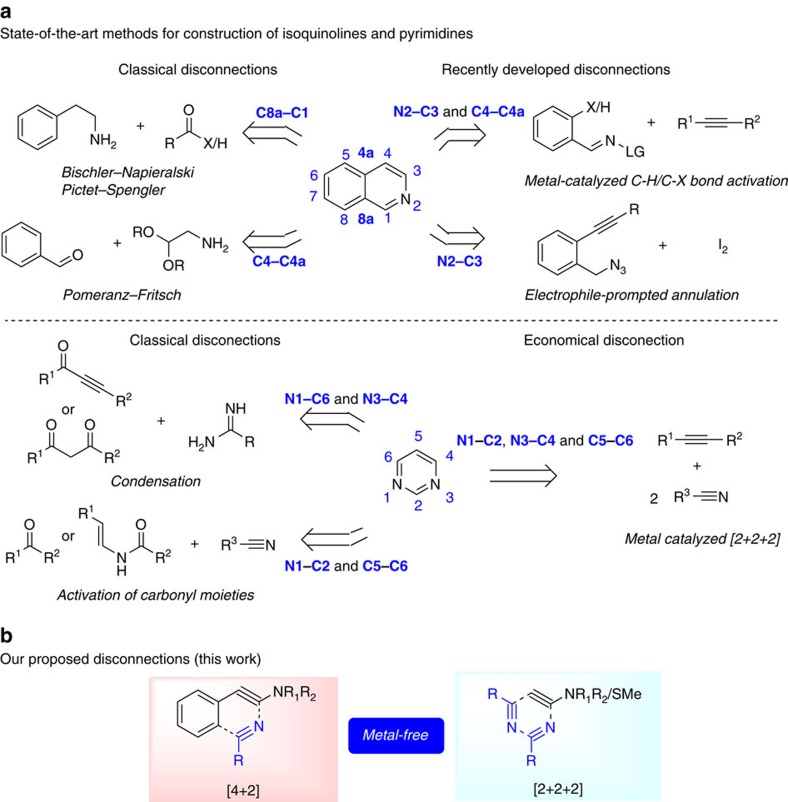
Synthetic disconnections. (**a**) Known synthetic disconnections for the isoquinoline and pyrimidine backbone. (**b**) Proposed direct disconnections through intermolecular metal-free alkyne/nitrile cycloadditions.
