# Supplementary material for: Metal-free intermolecular formal cycloadditions enable an orthogonal access to nitrogen heterocycles
Source: Nat Commun. 2016 Mar 15;7:10914. doi: 10.1038/ncomms10914 (PMC4796318; doi:10.1038/ncomms10914)
Supplement: Supplementary — Figures 1-67, Supplementary Tables 1-5, Supplementary Methods and Supplementary References [file ncomms10914-s1.pdf]

Supplementary Figures

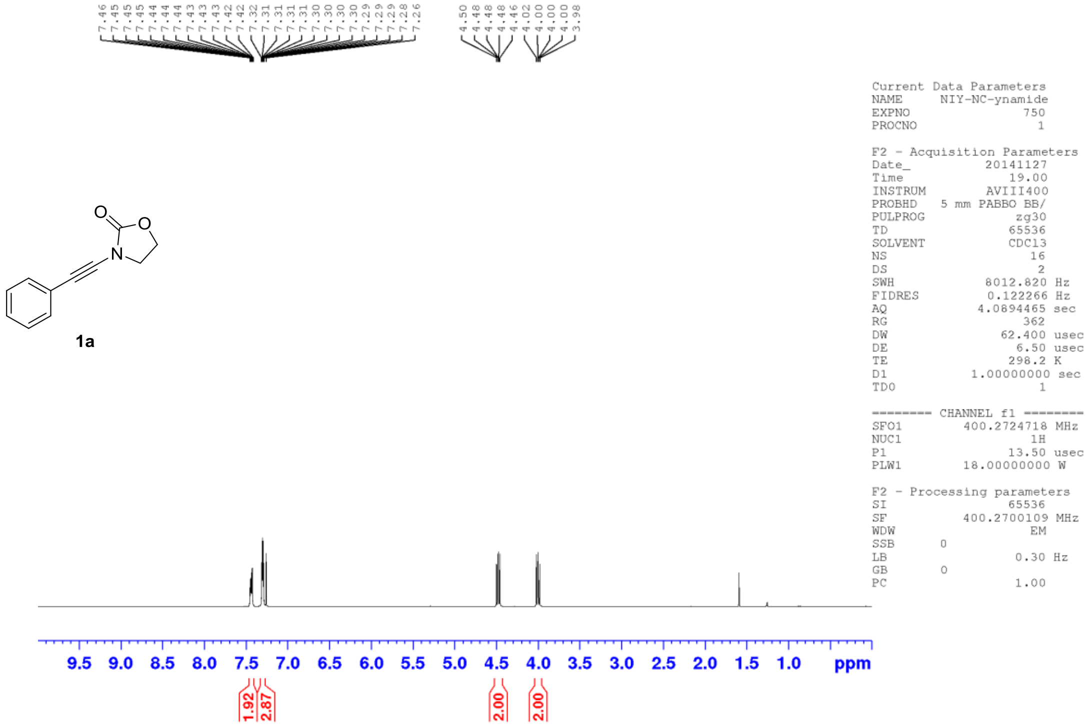

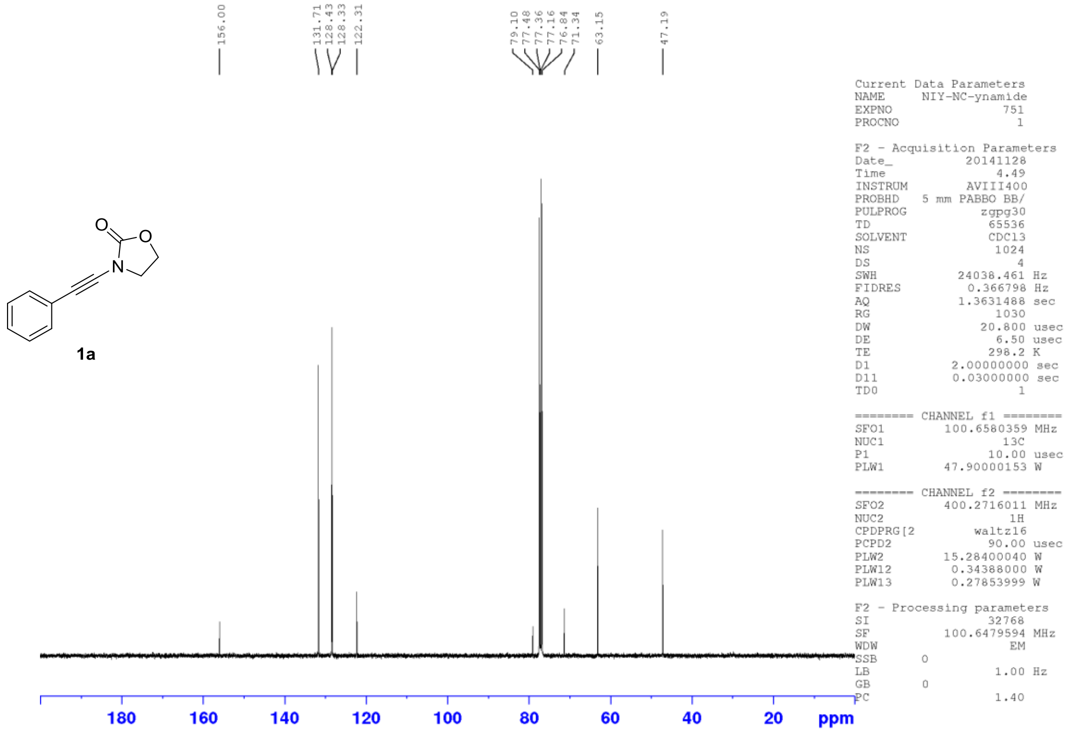

Supplementary Figure 1. ^{1}H NMR and ^{13}C NMR spectra of substrate **1a**

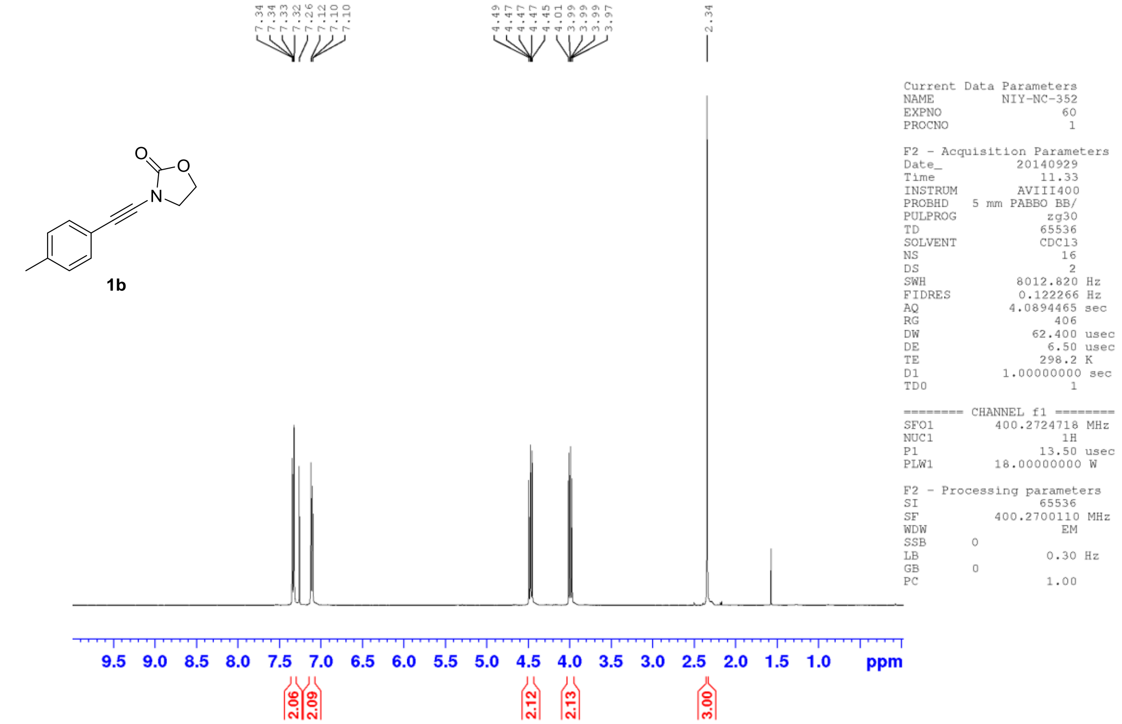

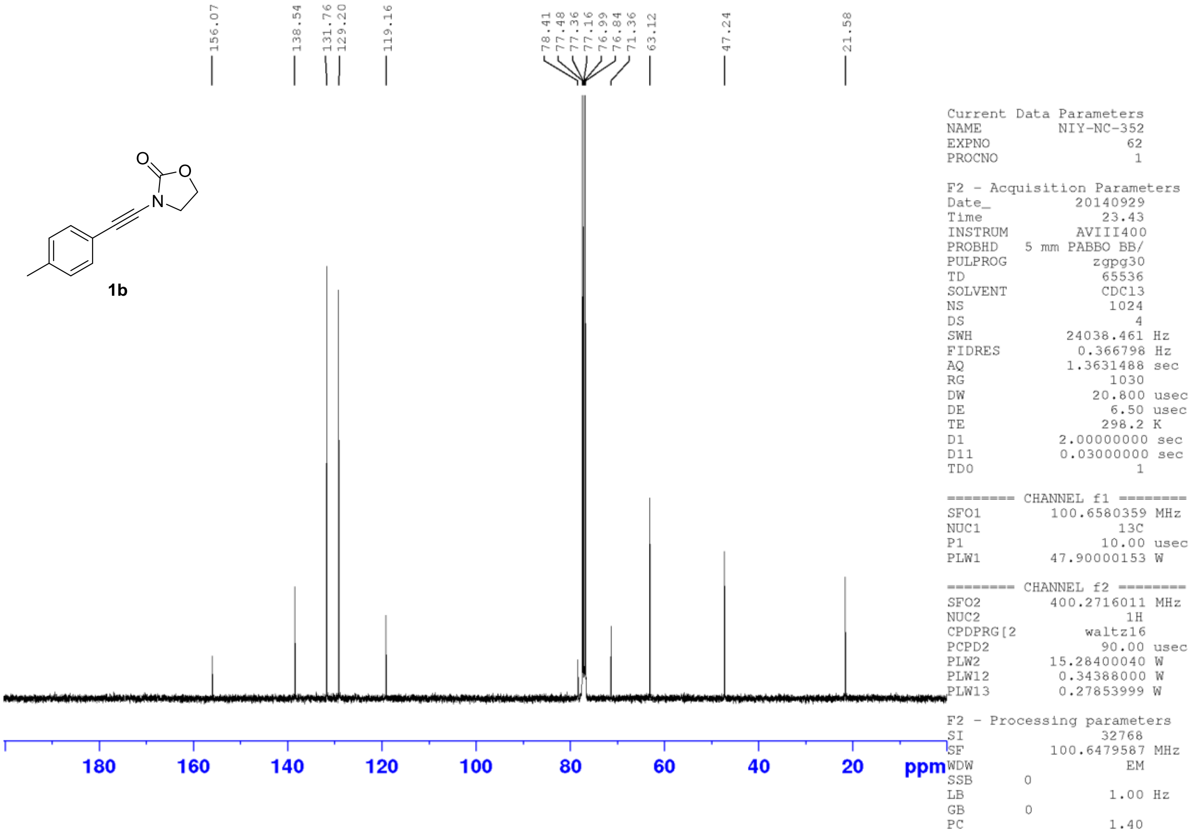

Supplementary Figure 2. ^{1}H NMR and ^{13}C NMR spectra of substrate **1b**

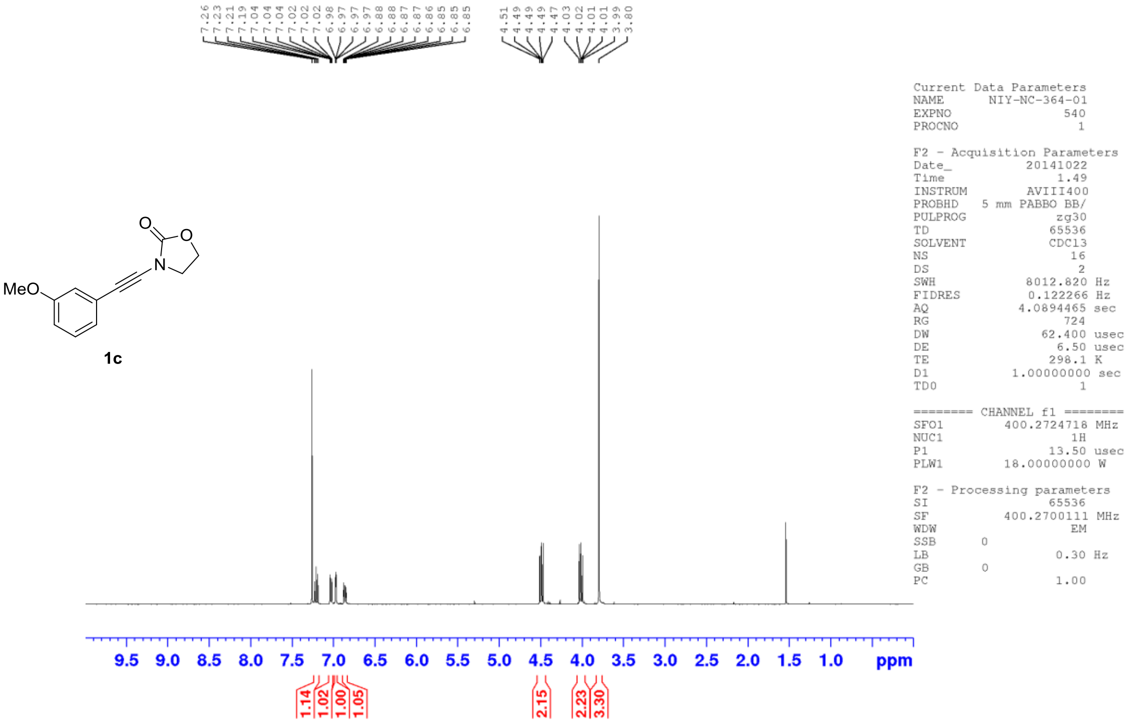

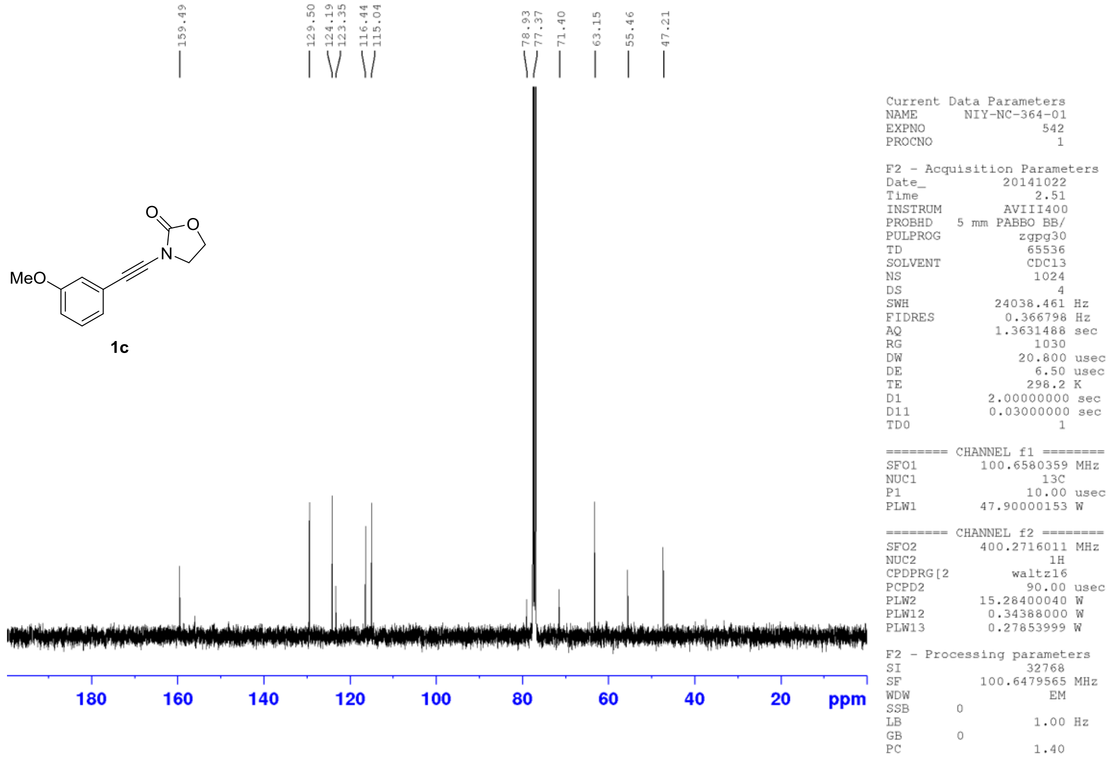

Supplementary Figure 3. ^{1}H NMR and ^{13}C NMR spectra of substrate **1c**

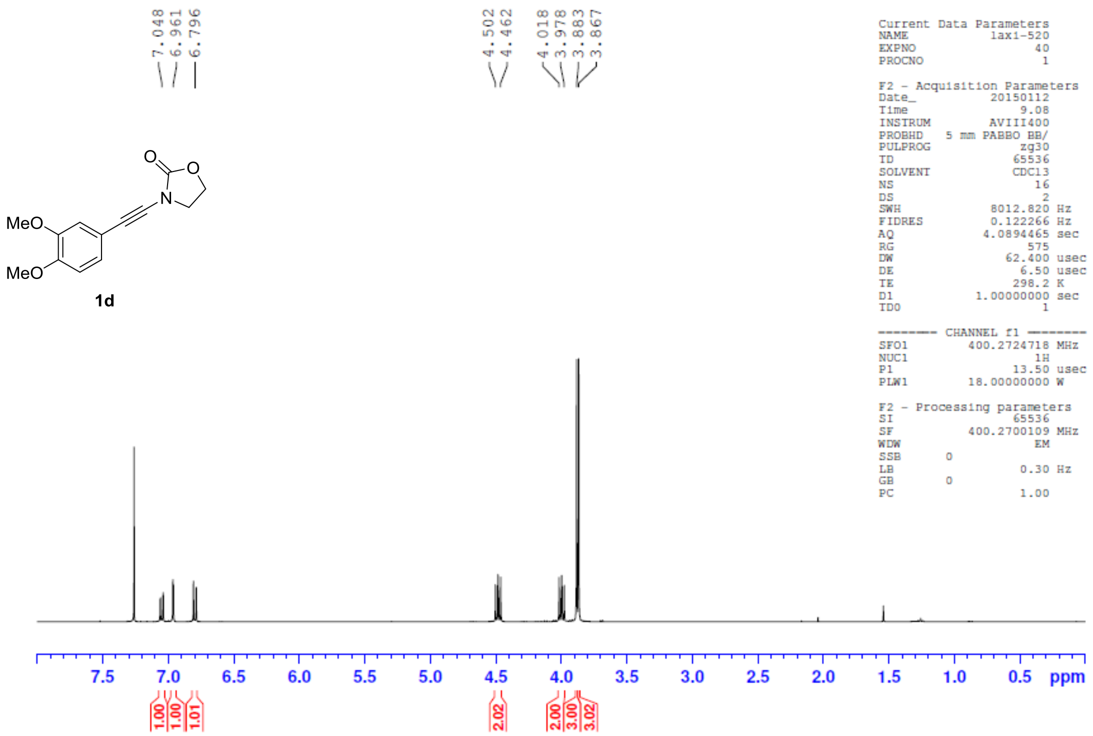

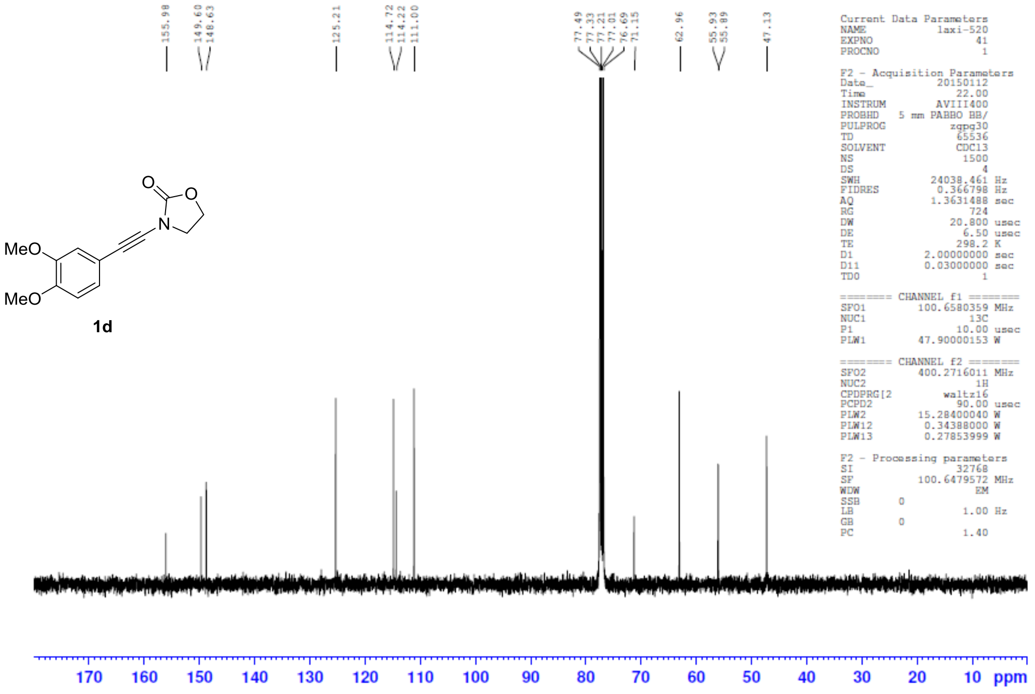

Supplementary Figure 4. ^{1}H NMR and ^{13}C NMR spectra of substrate **1d**

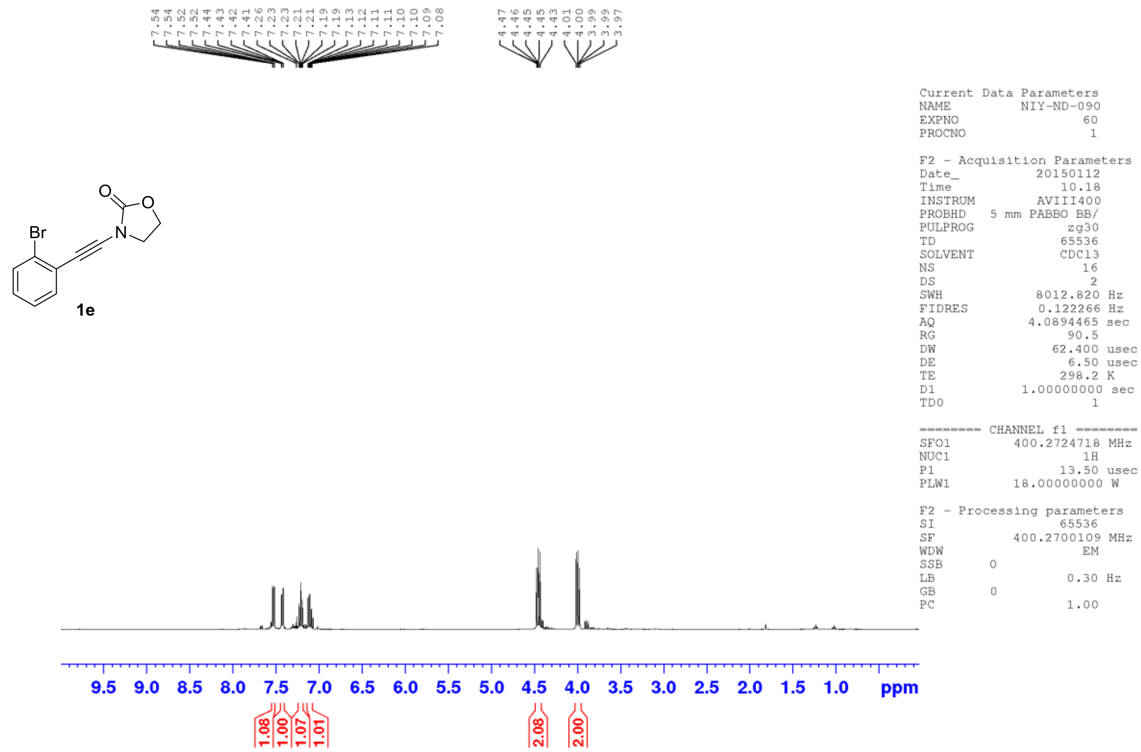

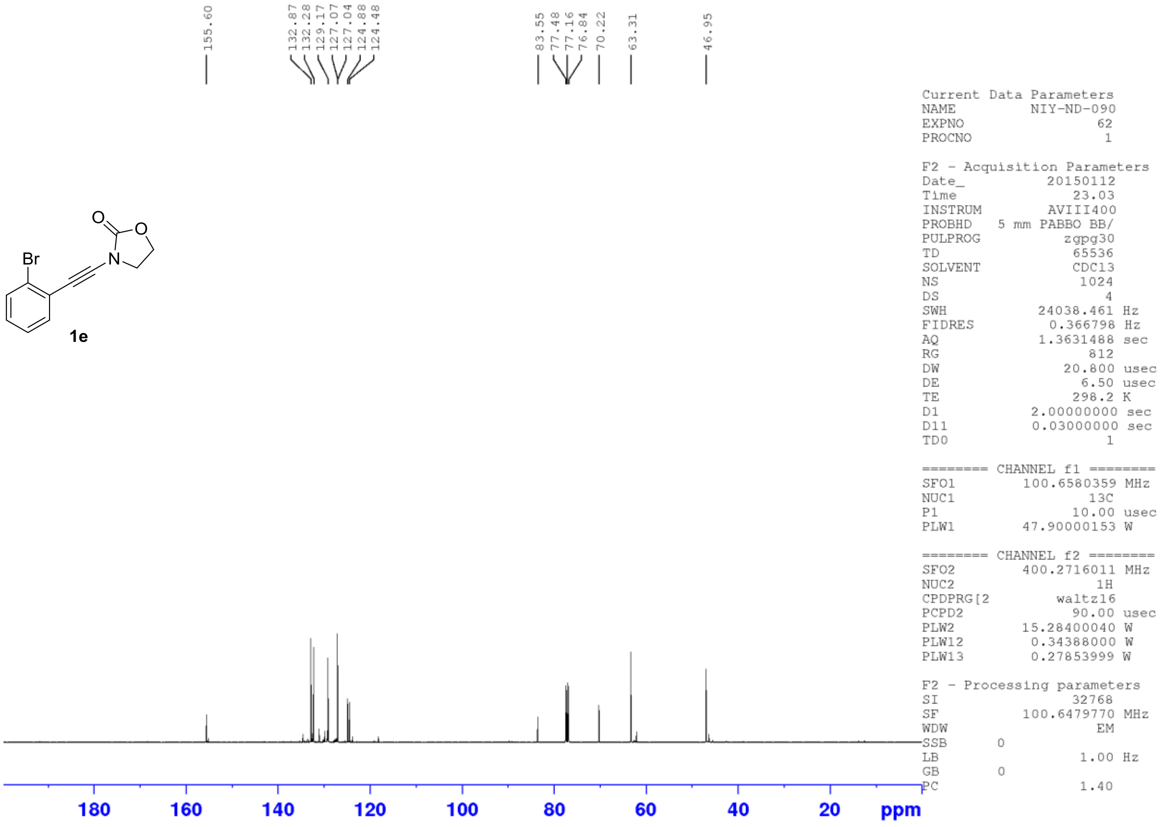

Supplementary Figure 5. . ^{1}H NMR and ^{13}C NMR spectra of substrate **1e**

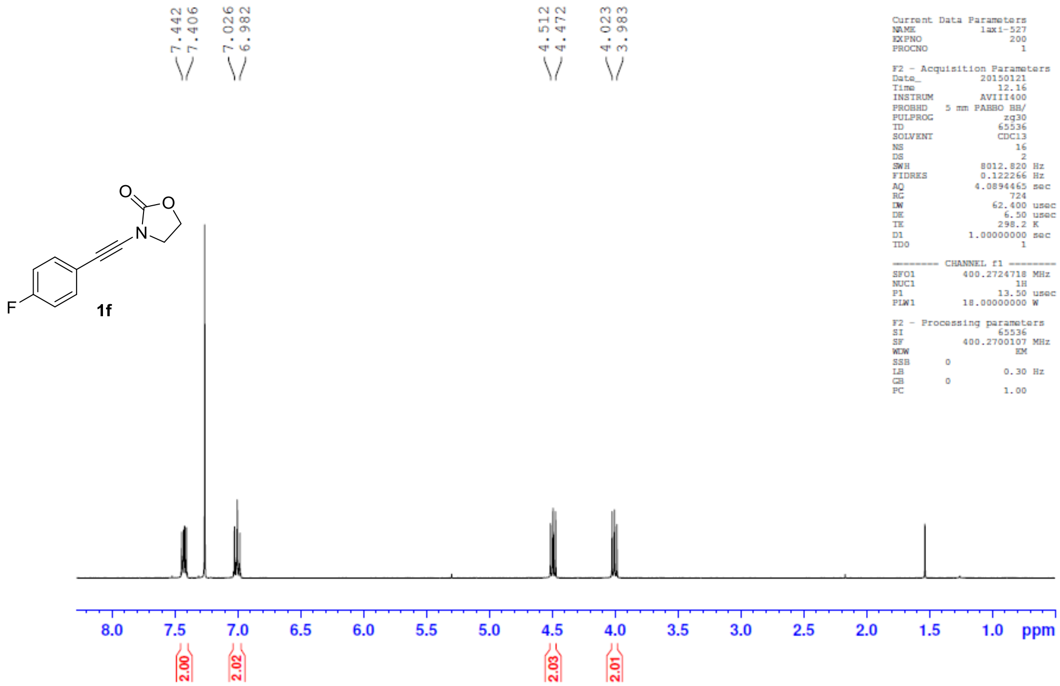

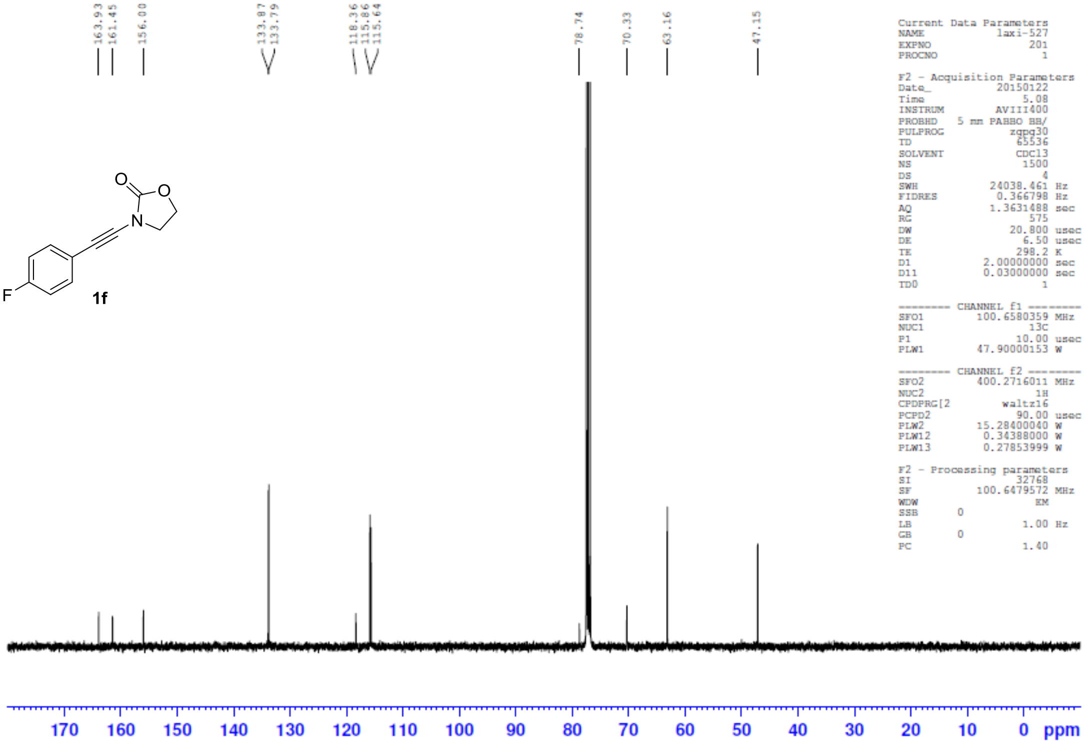

Supplementary Figure 6. ^{1}H NMR and ^{13}C NMR spectra of substrate **1f**

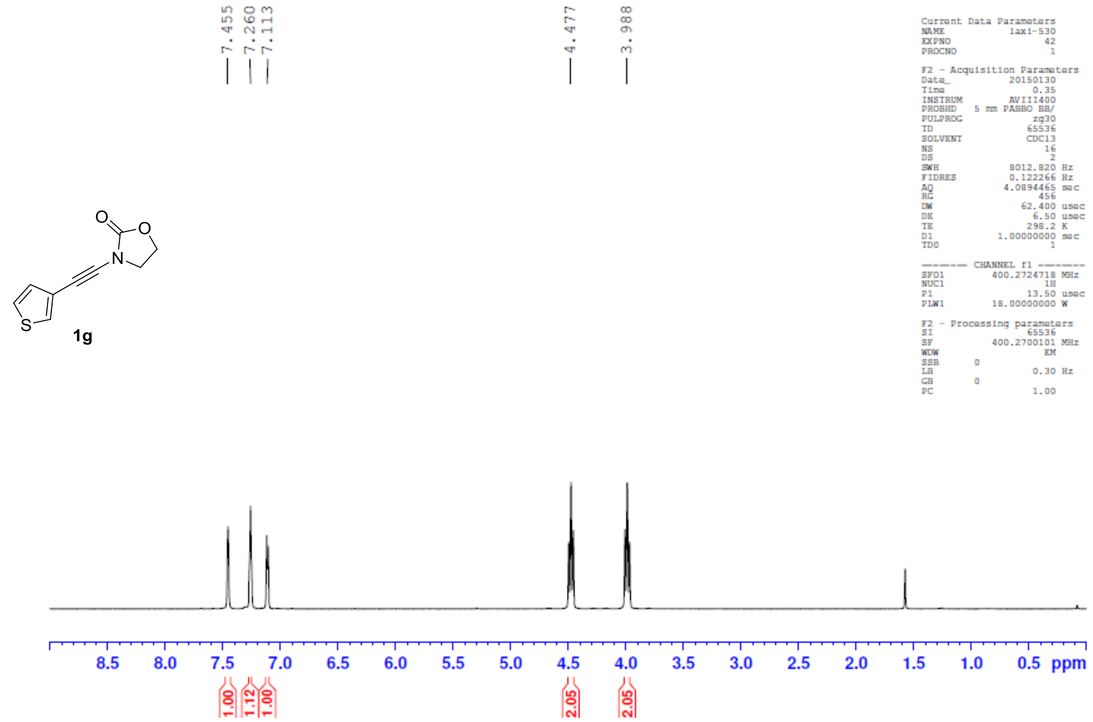

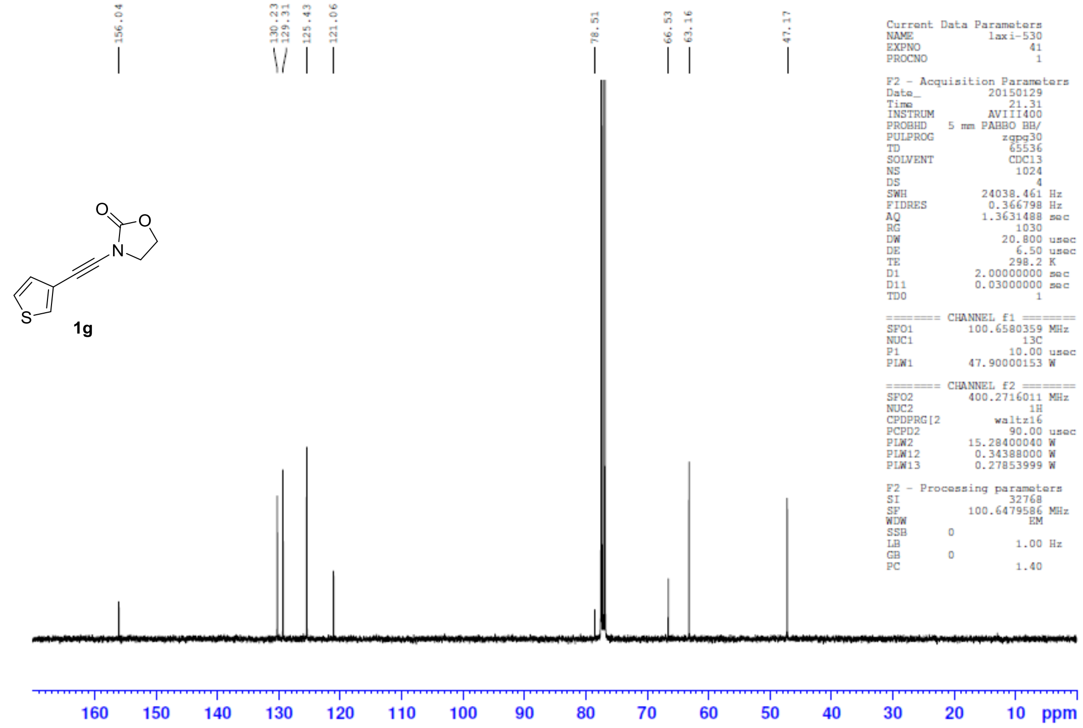

Supplementary Figure 7. ^{1}H NMR and ^{13}C NMR spectra of substrate **1g**

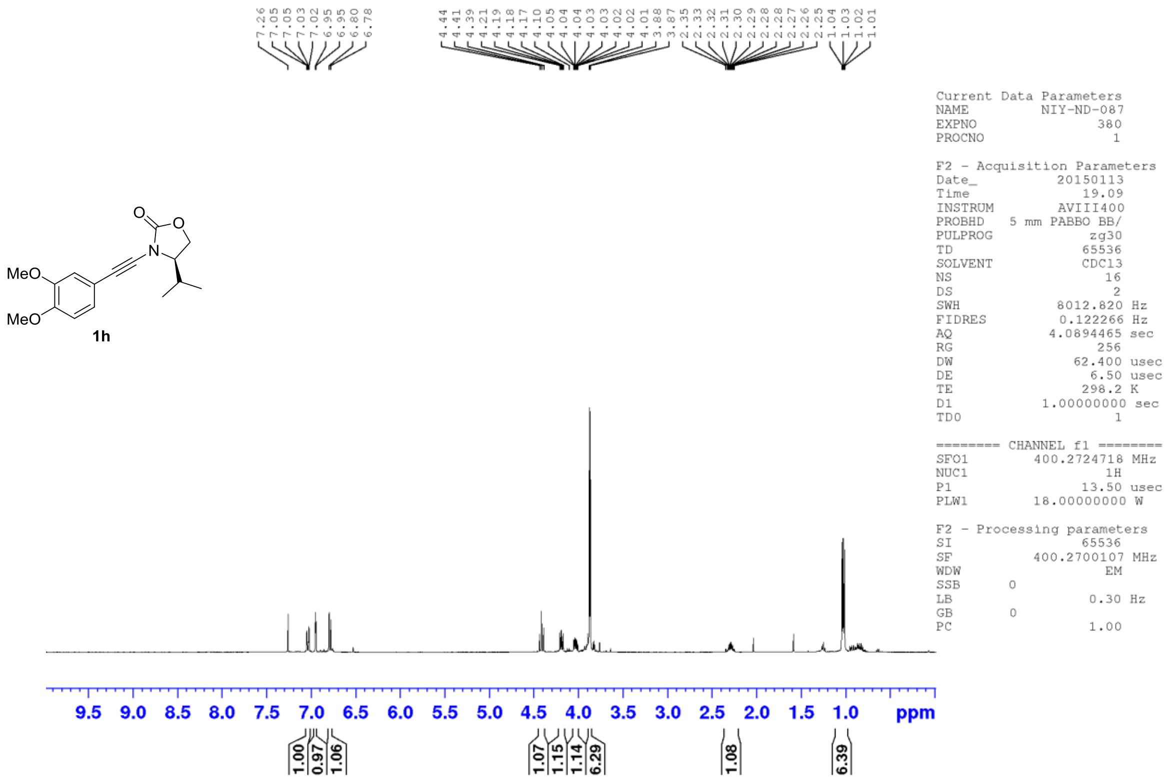

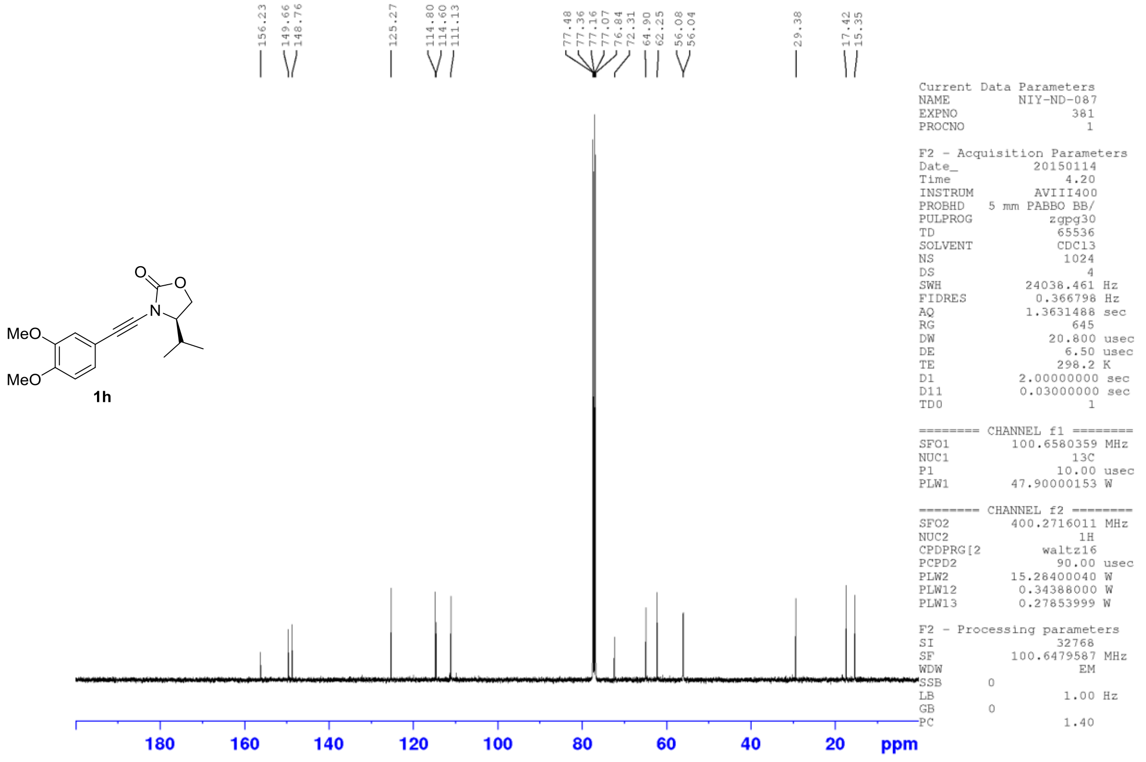

Supplementary Figure 8. ^{1}H NMR and ^{13}C NMR spectra of substrate **1h**

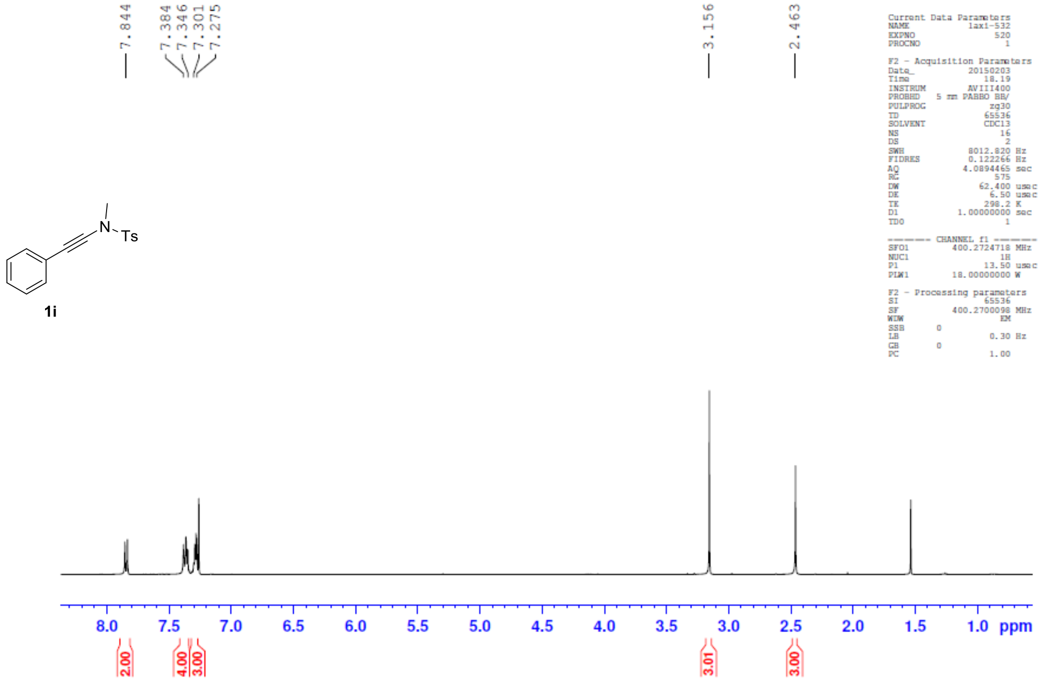

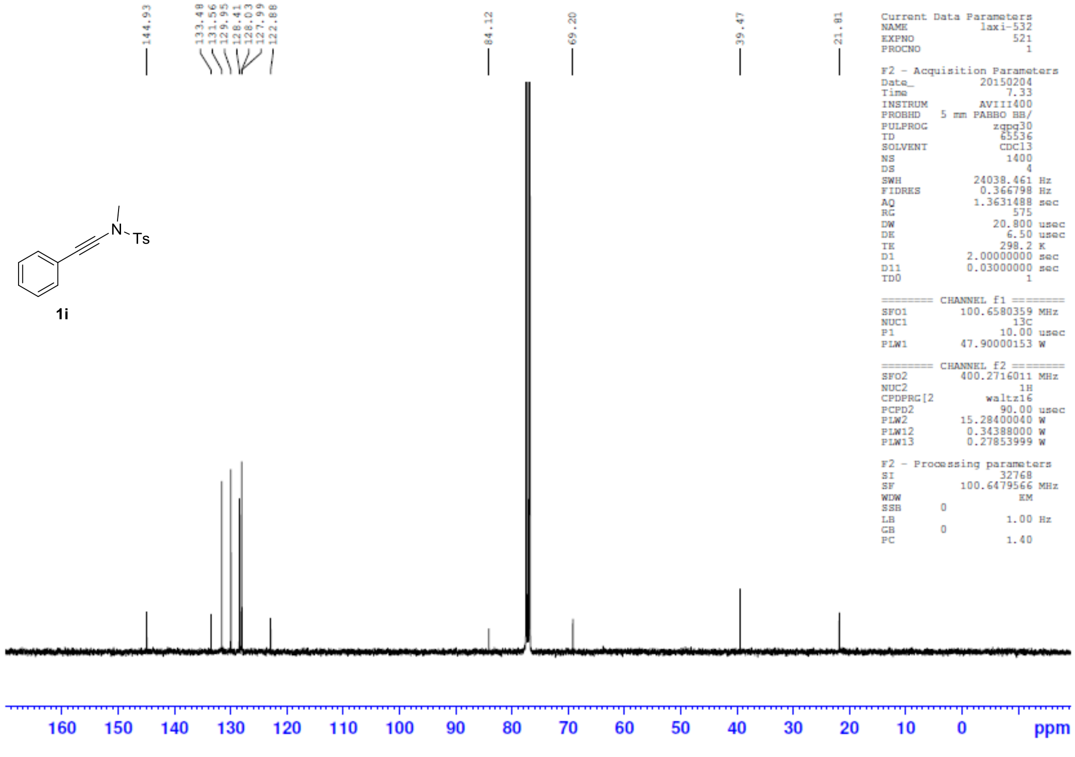

Supplementary Figure 9. ^{1}H NMR and ^{13}C NMR spectra of substrate **1i**

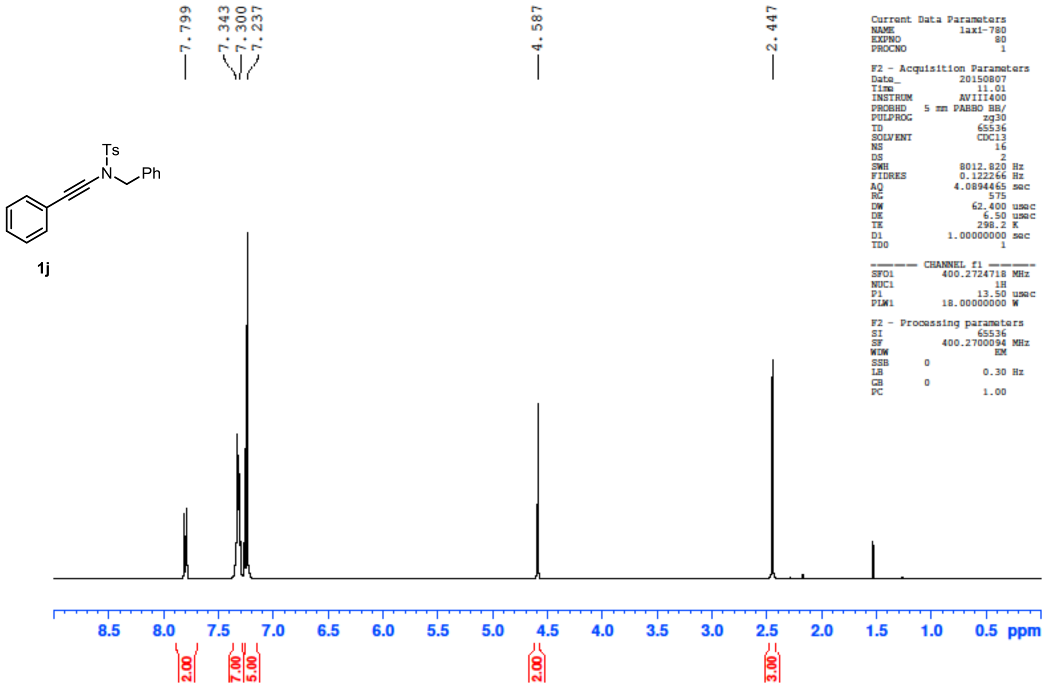

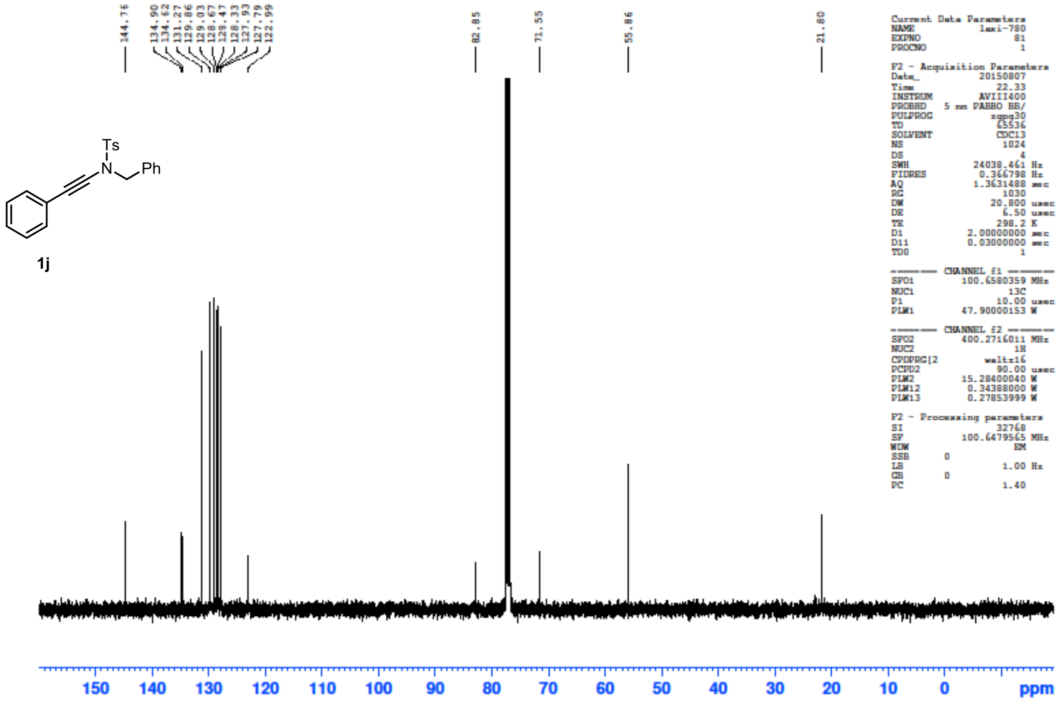

Supplementary Figure 10. ^{1}H NMR and ^{13}C NMR spectra of substrate **1j**

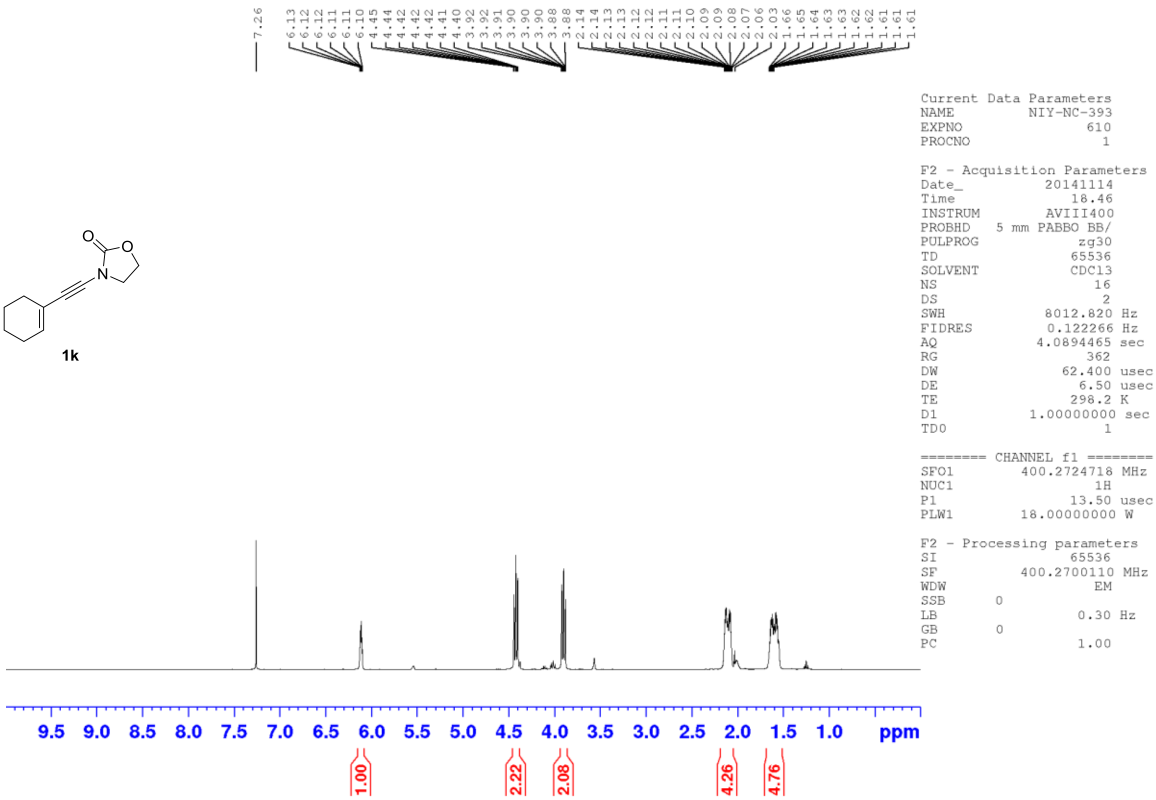

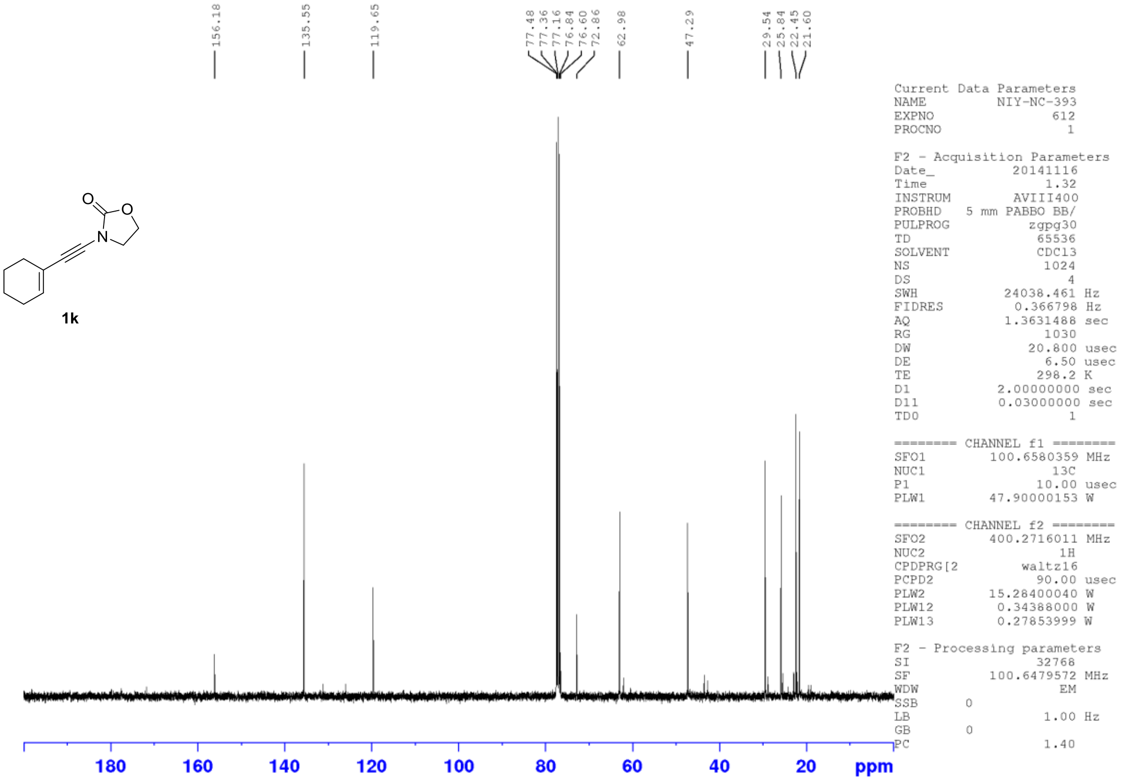

Supplementary Figure 11. ^{1}H NMR and ^{13}C NMR spectra of substrate **1k**

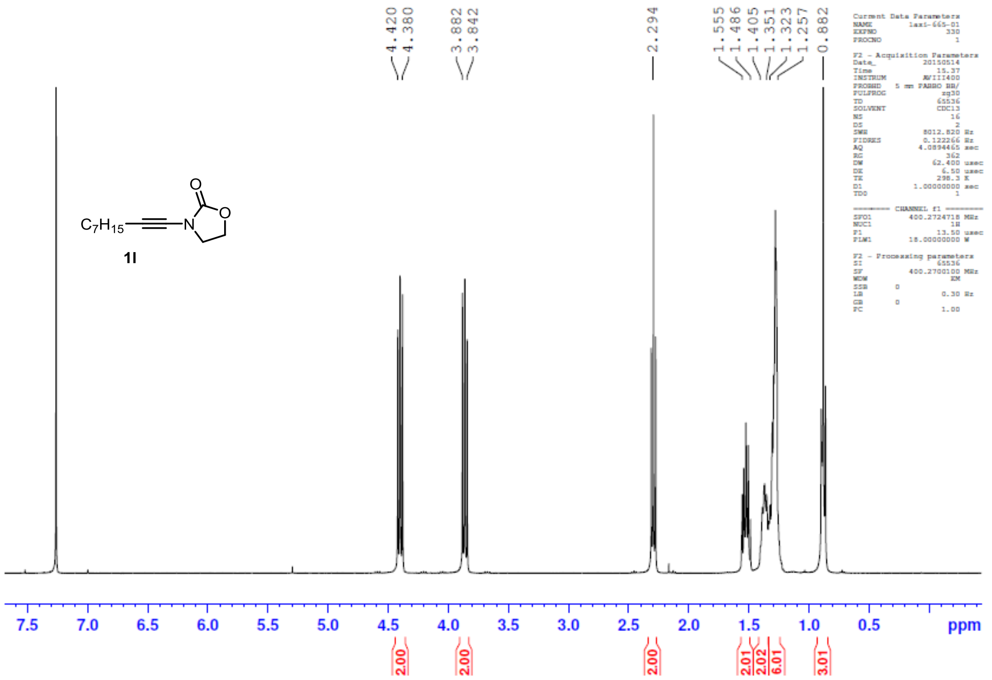

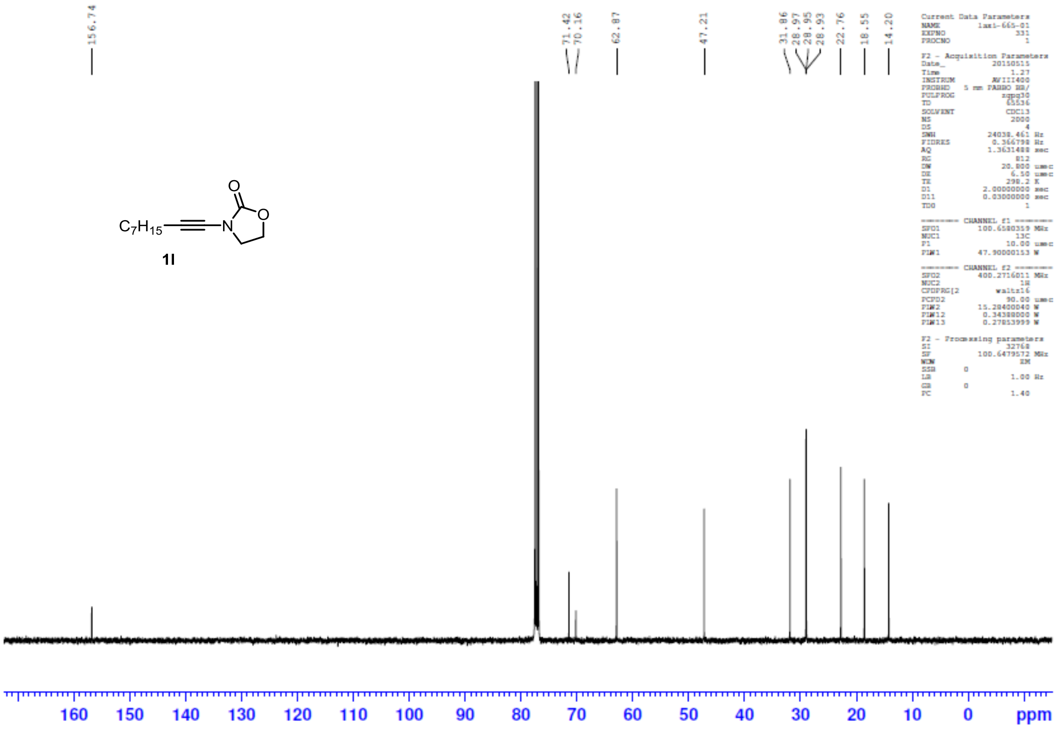

Supplementary Figure 12. ^{1}H NMR and ^{13}C NMR spectra of substrate **1l**

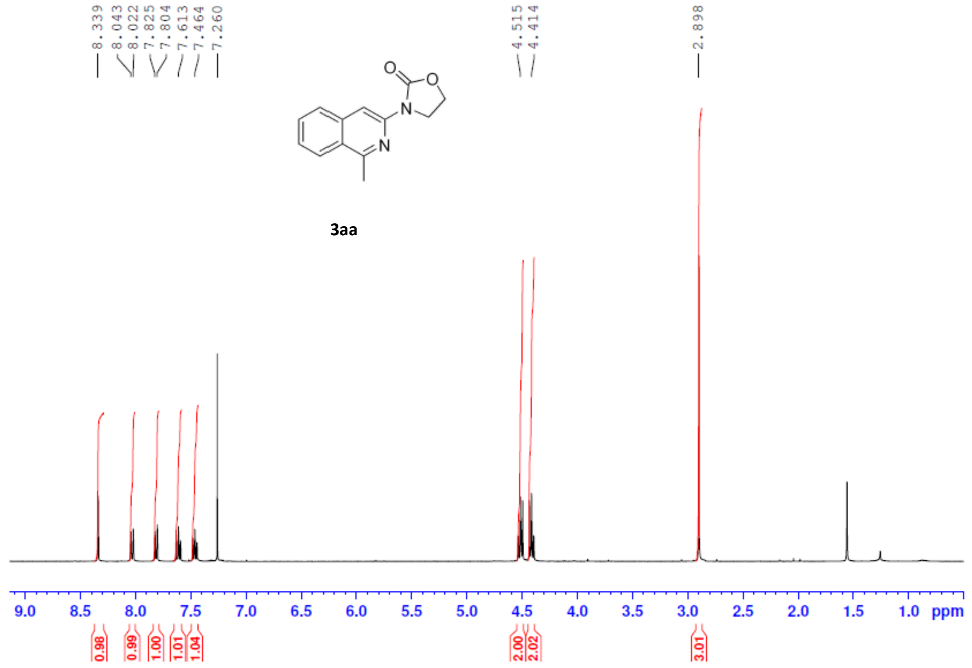

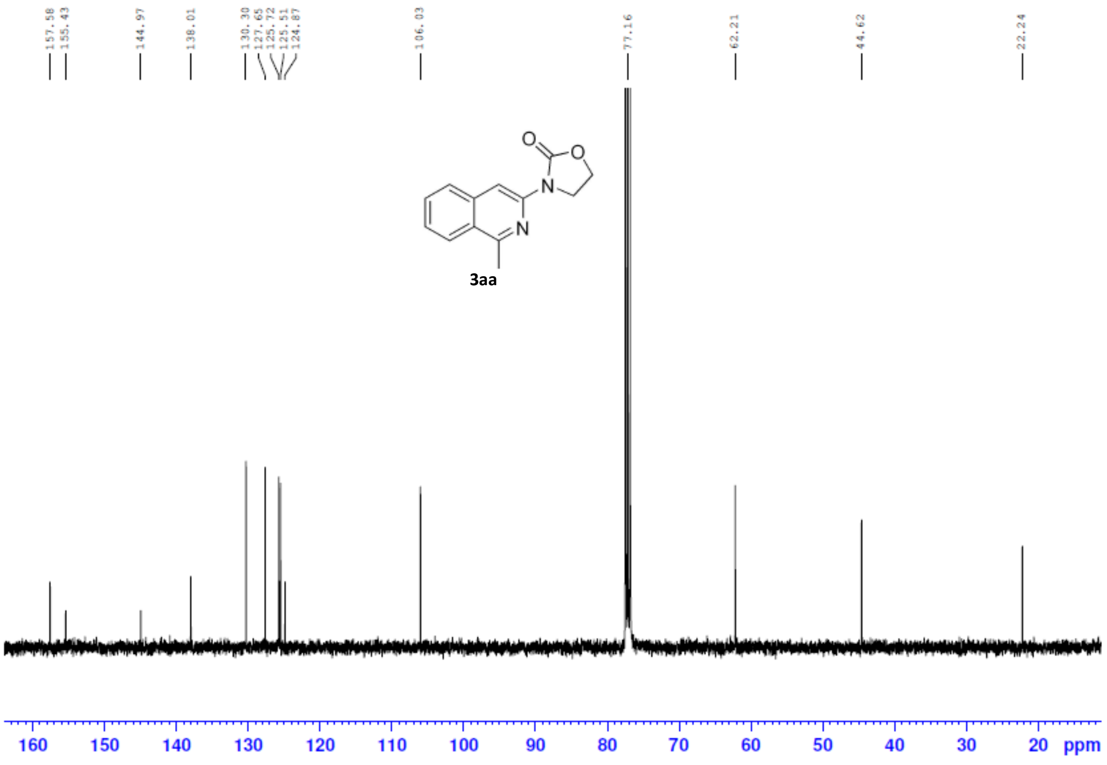

Supplementary Figure 13. ^{1}H NMR and ^{13}C NMR spectra of substrate **3aa**

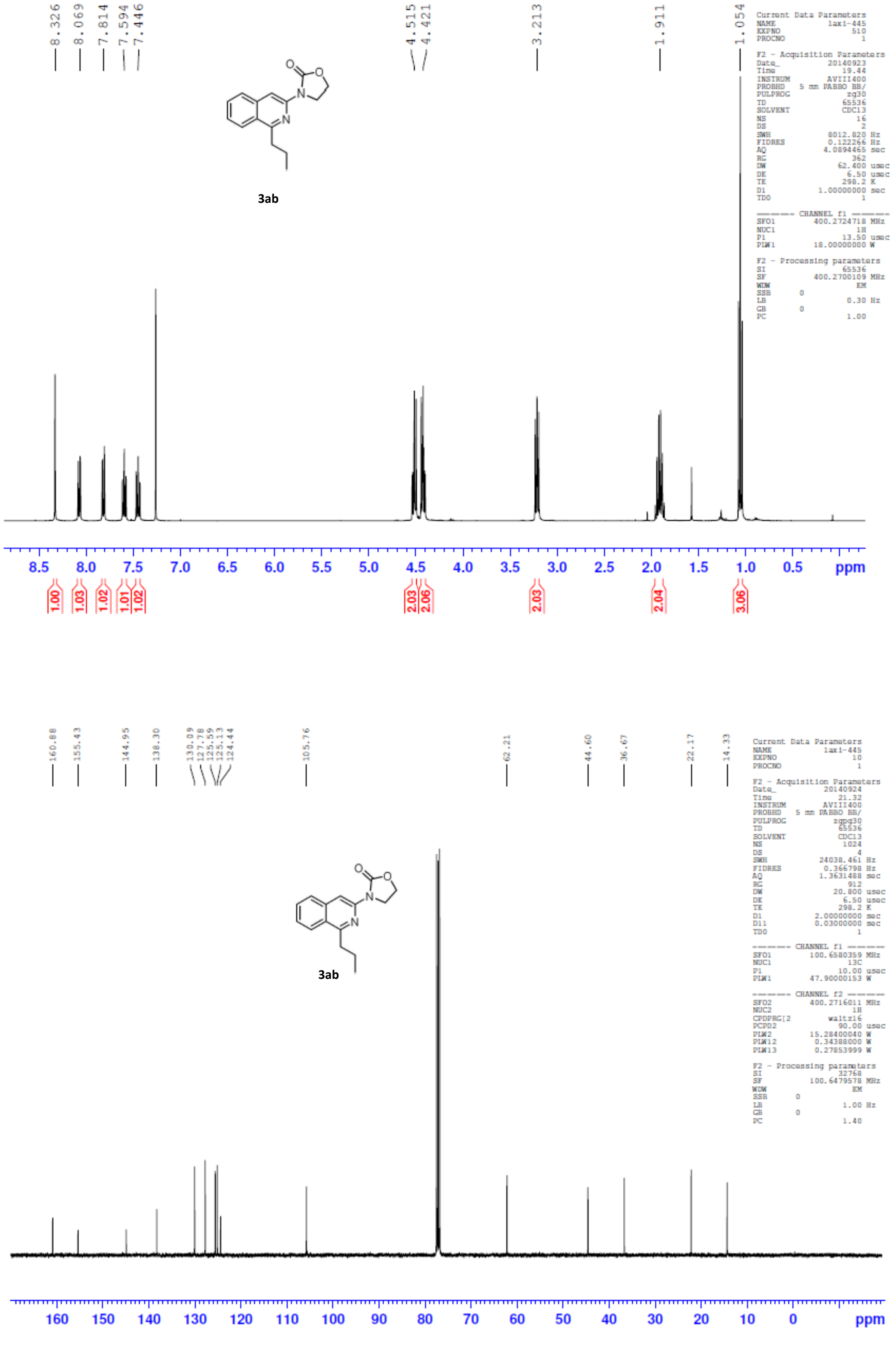

Supplementary Figure 14. ^{1}H NMR and ^{13}C NMR spectra of substrate **3ab**

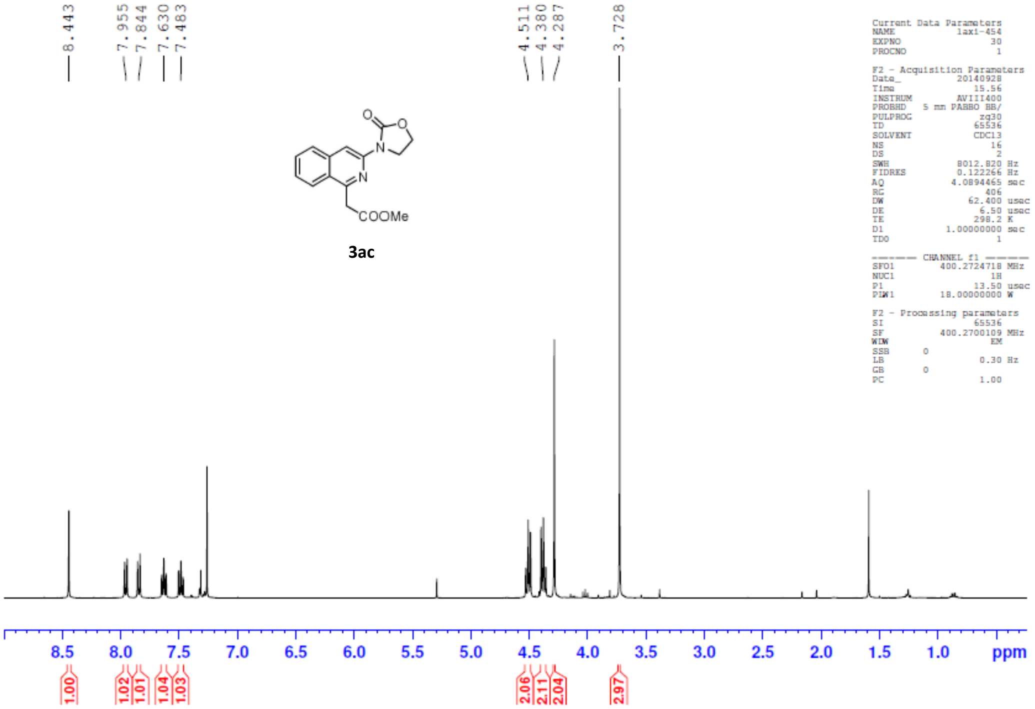

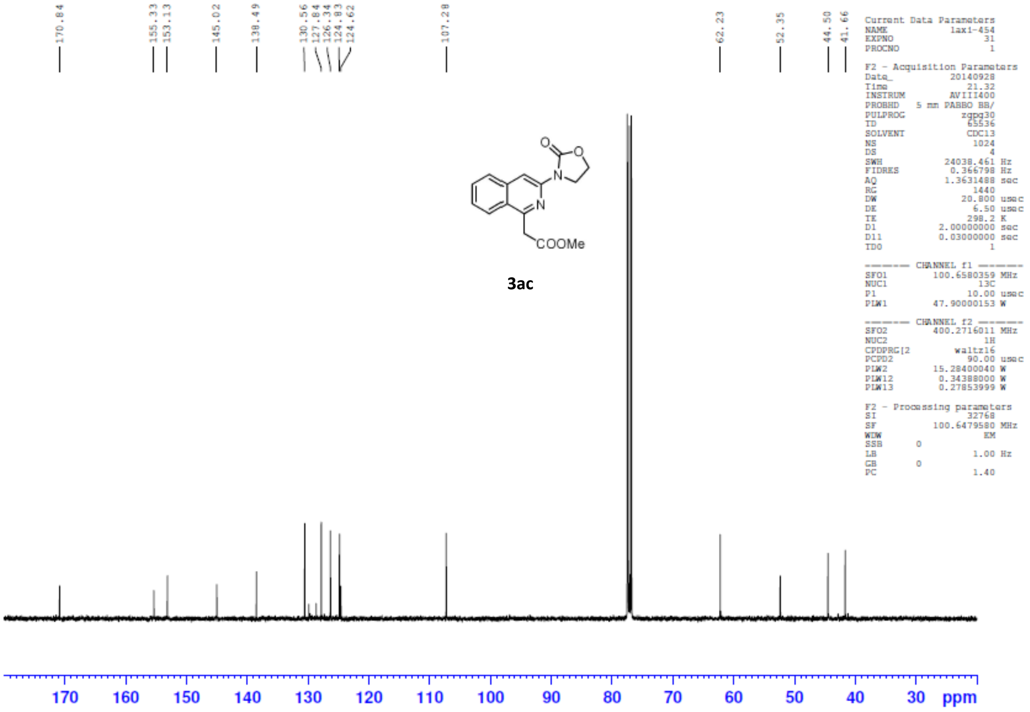

Supplementary Figure 15. ^{1}H NMR and ^{13}C NMR spectra of substrate **3ac**

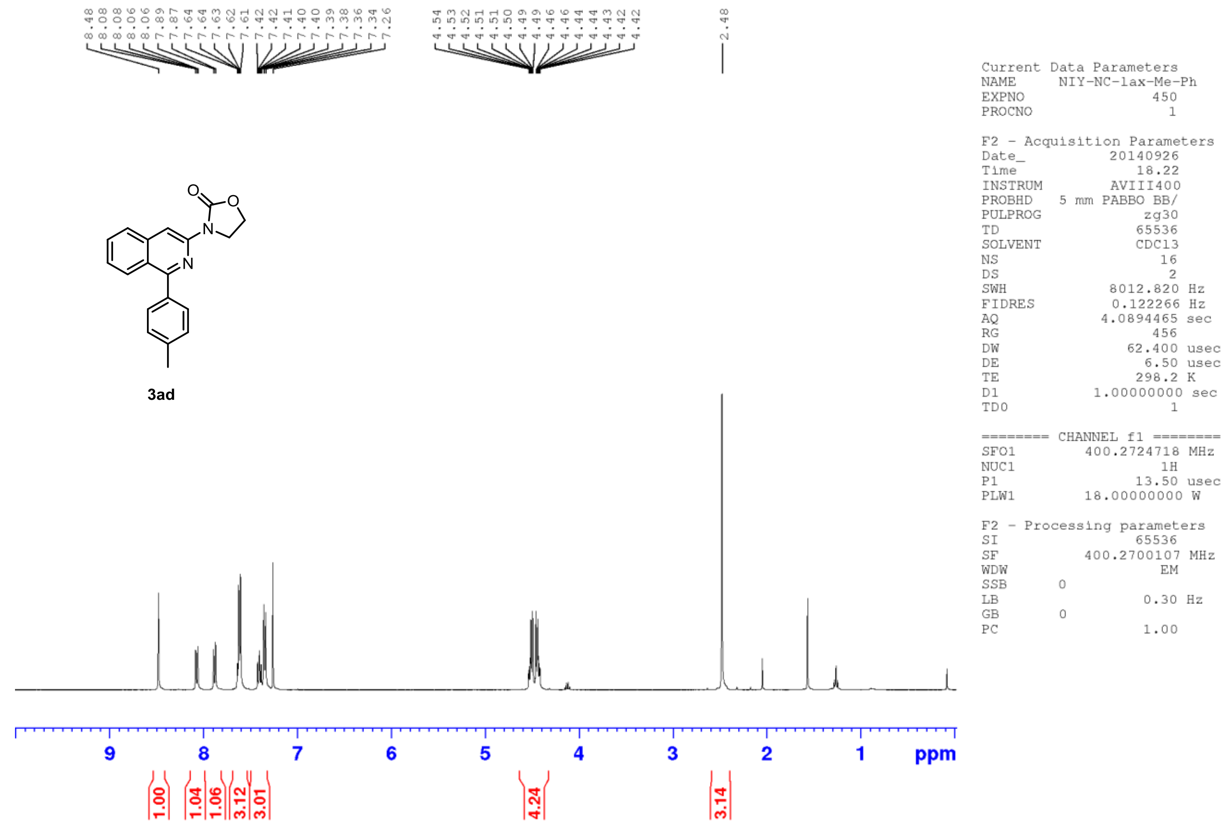

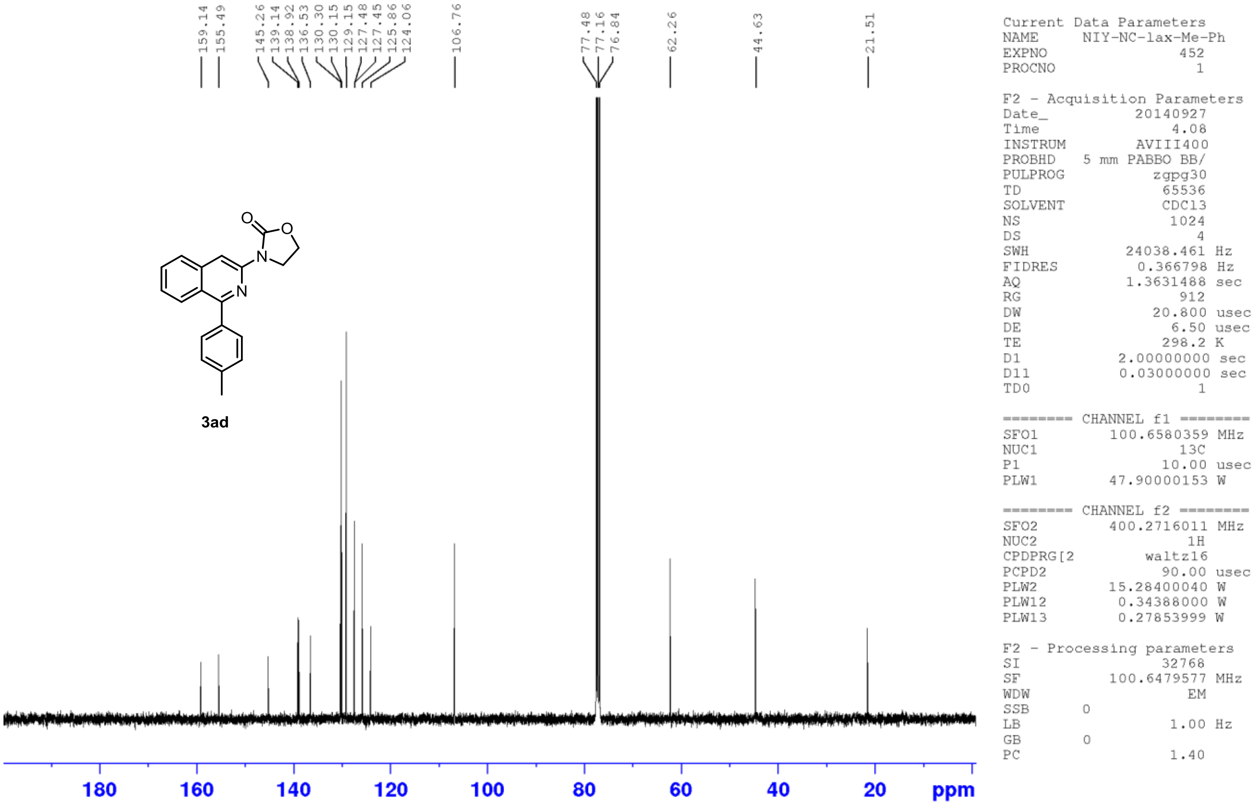

Supplementary Figure 16. ^{1}H NMR and ^{13}C NMR spectra of substrate **3ad**

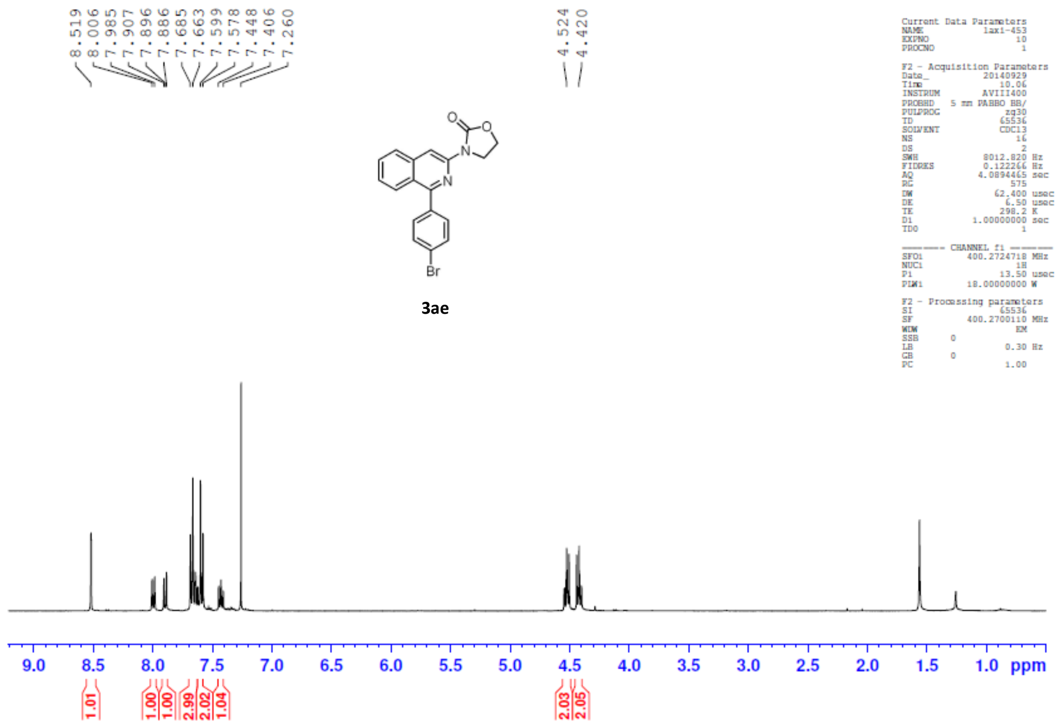

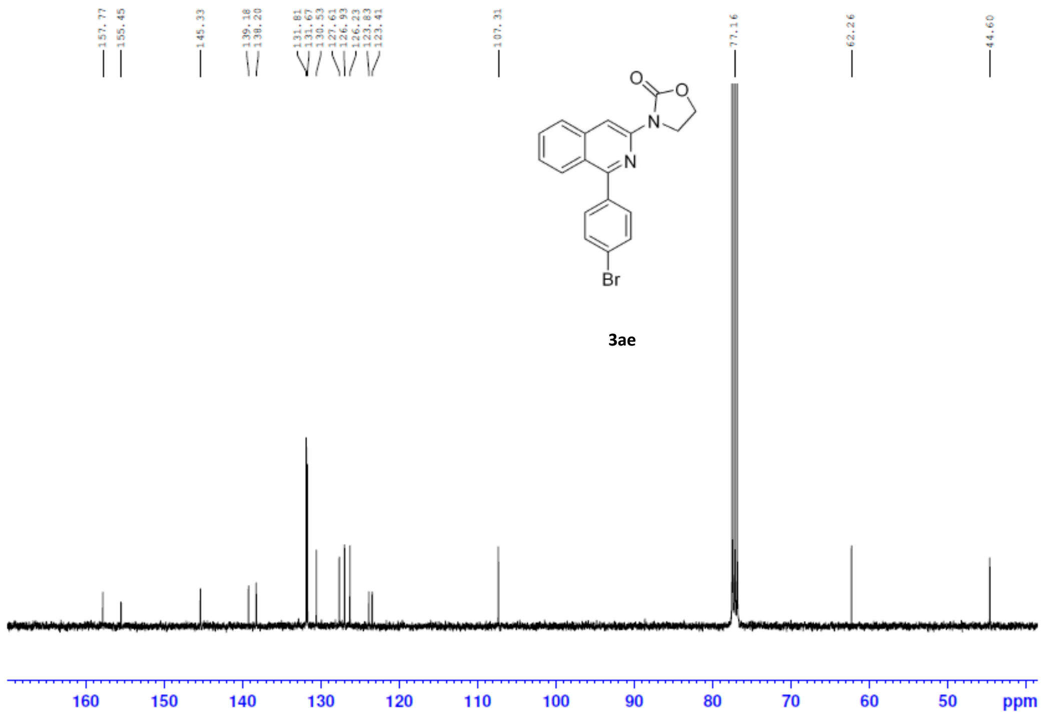

Supplementary Figure 17. ^{1}H NMR and ^{13}C NMR spectra of substrate **3ae**

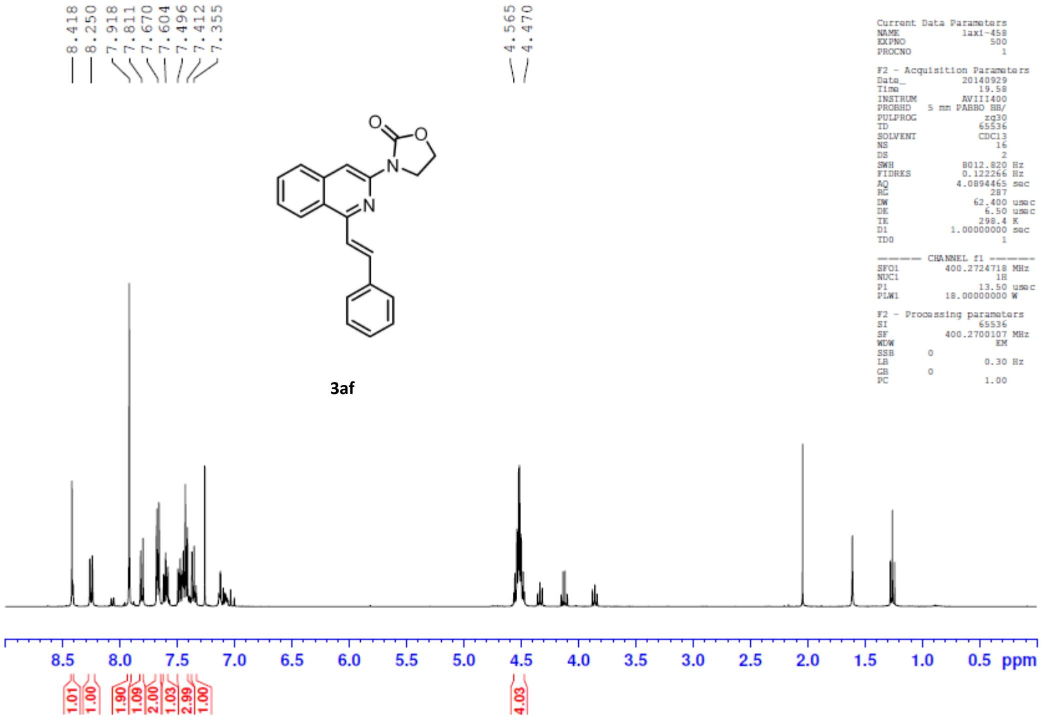

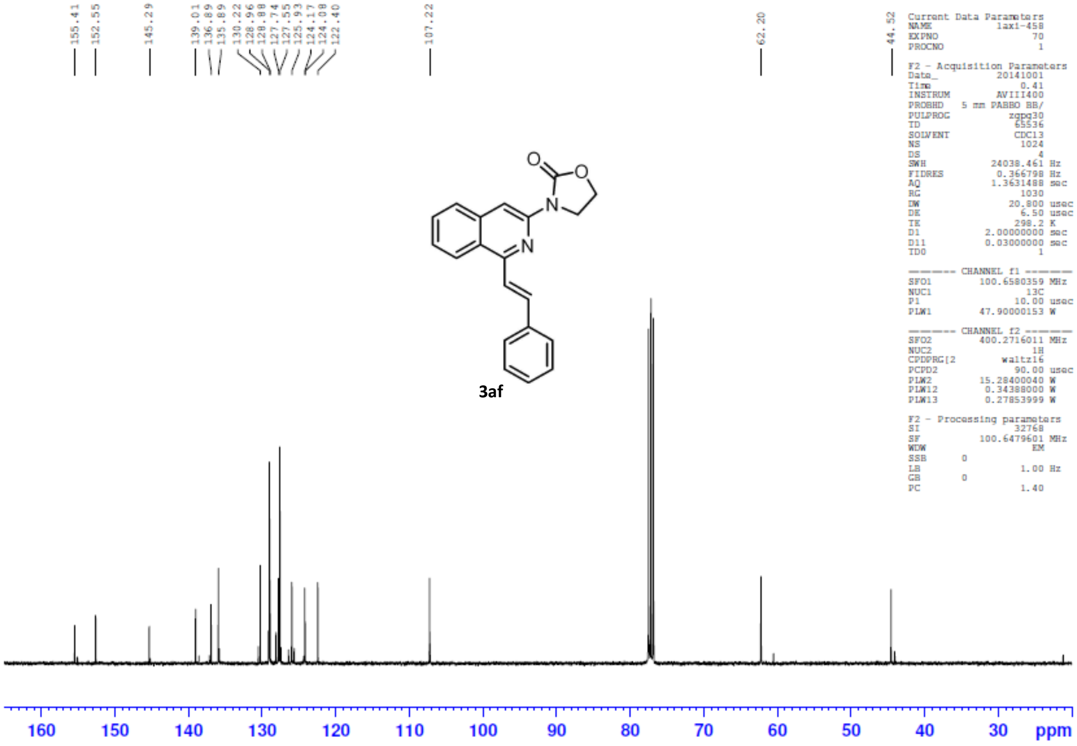

Supplementary Figure 18. ^{1}H NMR and ^{13}C NMR spectra of substrate **3af**

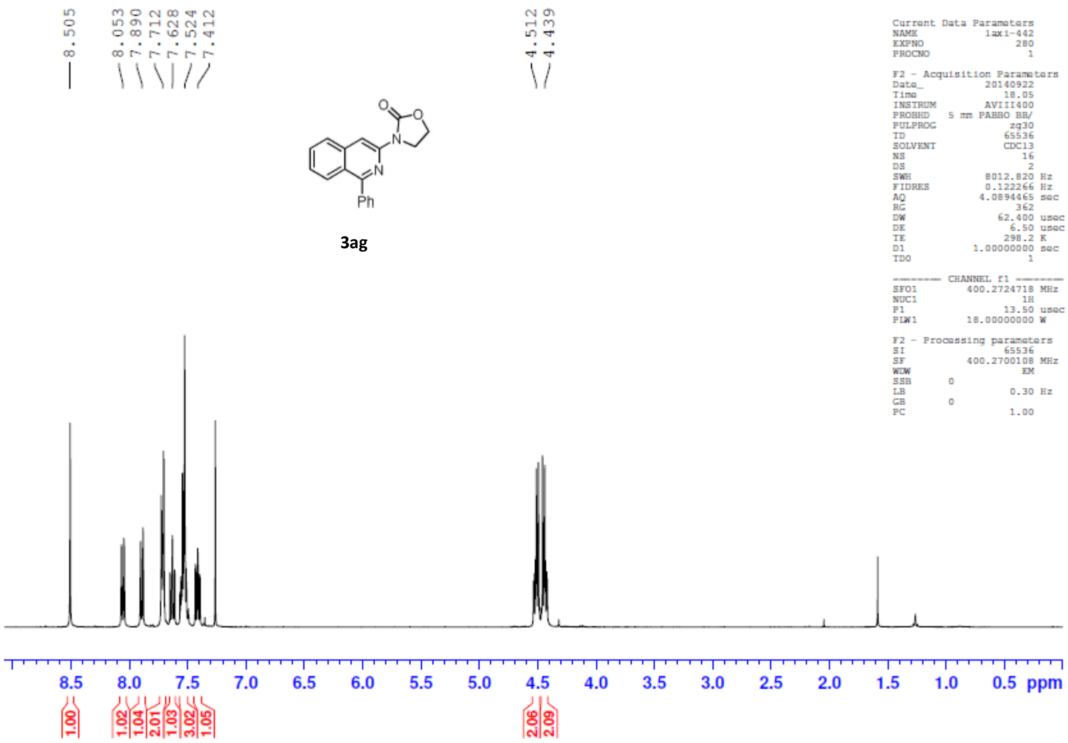

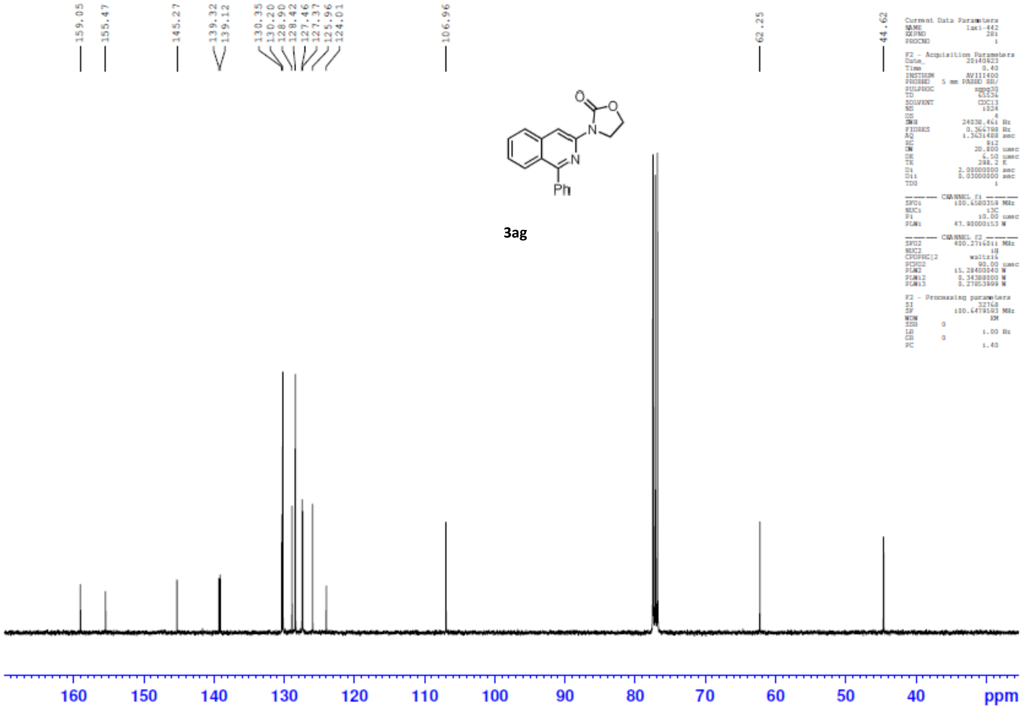

Supplementary Figure 19. ^{1}H NMR and ^{13}C NMR spectra of substrate **3ag**

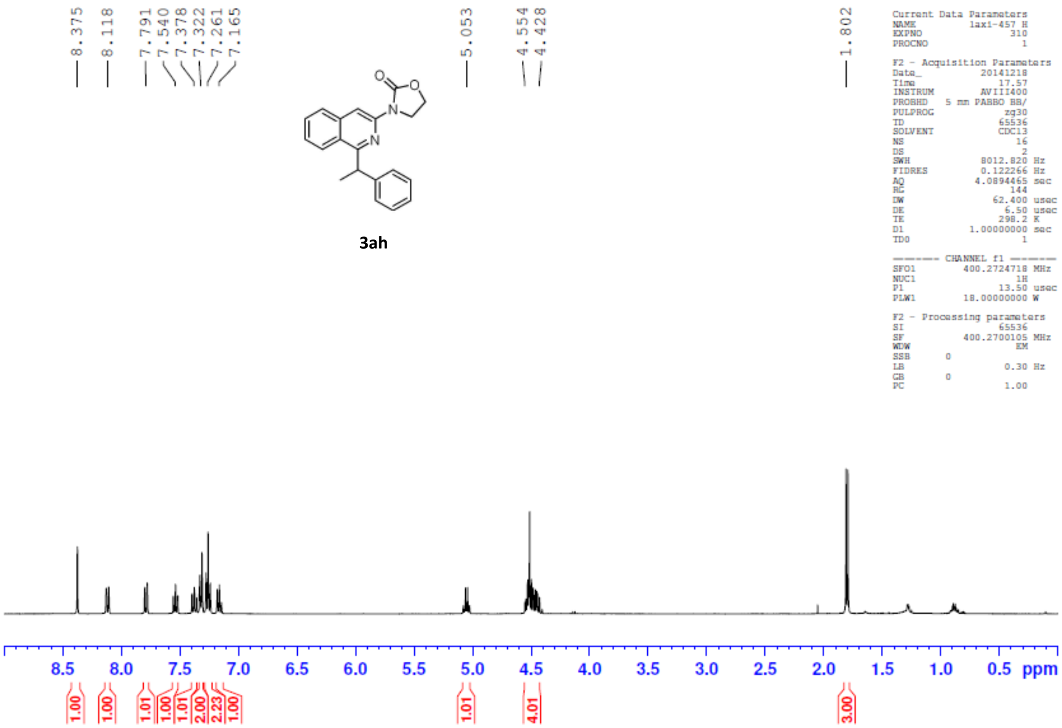

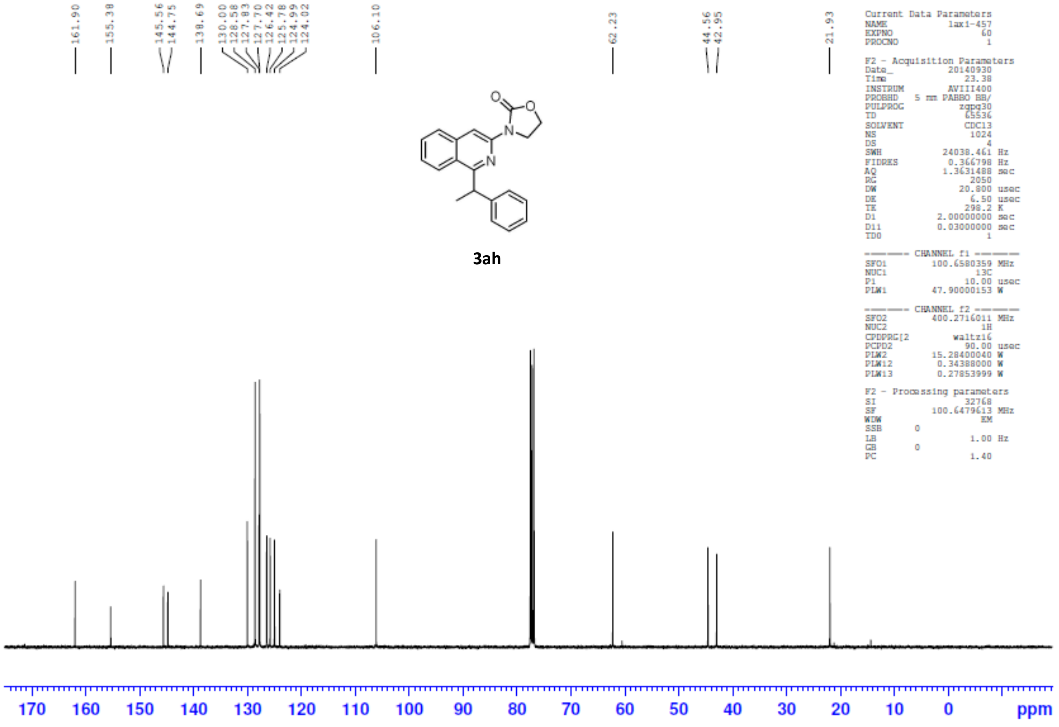

Supplementary Figure 20. ^{1}H NMR and ^{13}C NMR spectra of substrate **3ah**

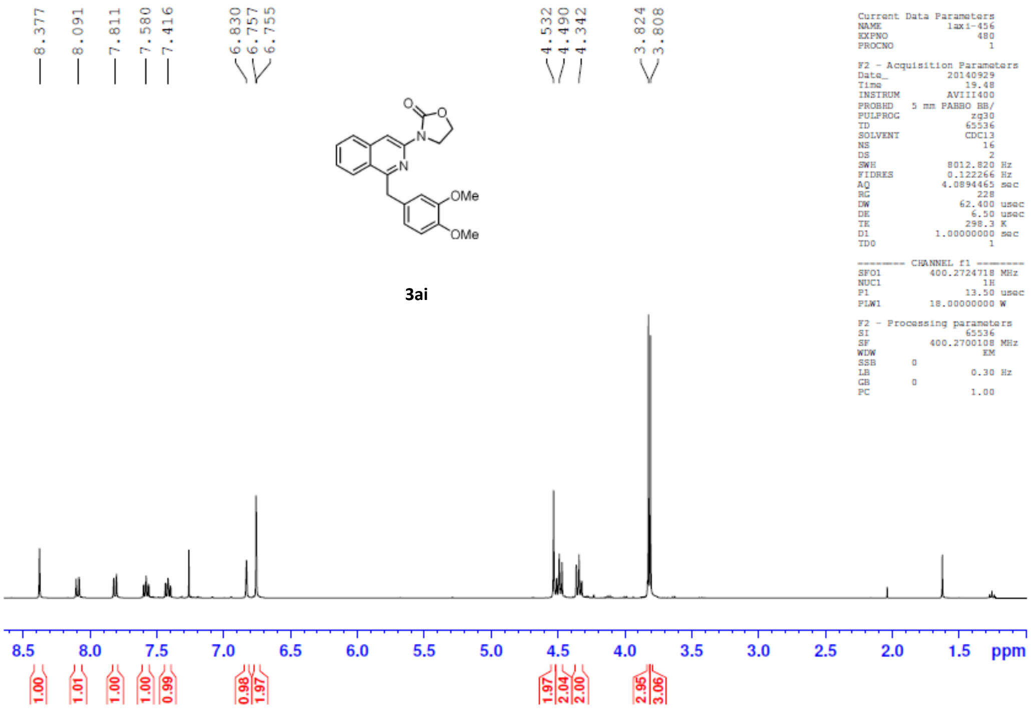

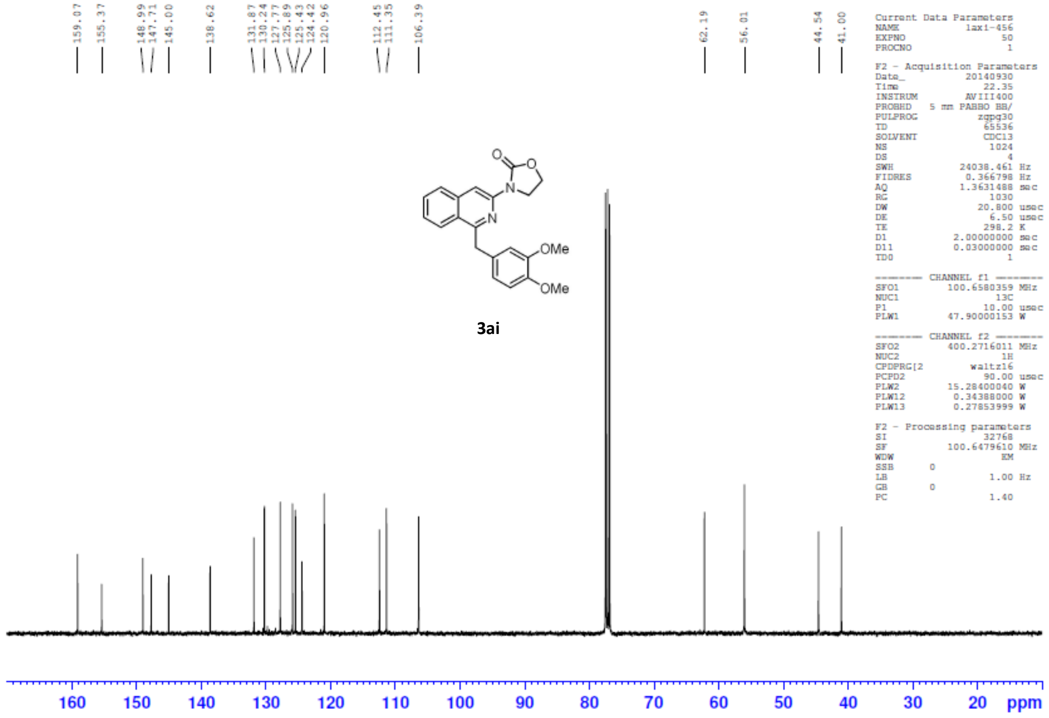

Supplementary Figure 21. ^{1}H NMR and ^{13}C NMR spectra of substrate **3ai**

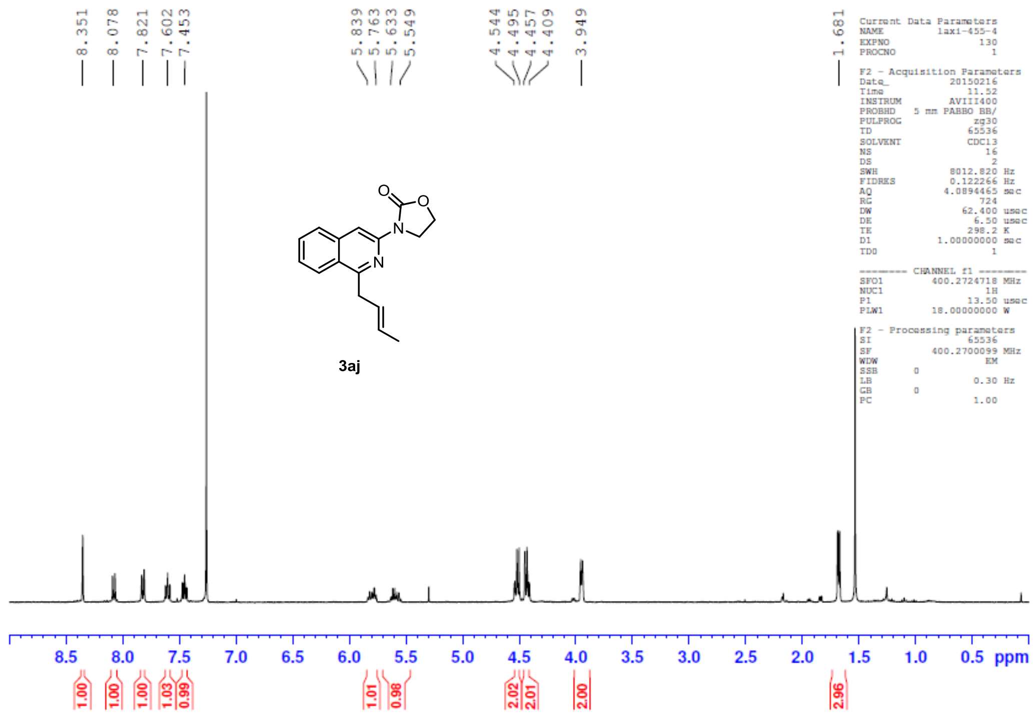

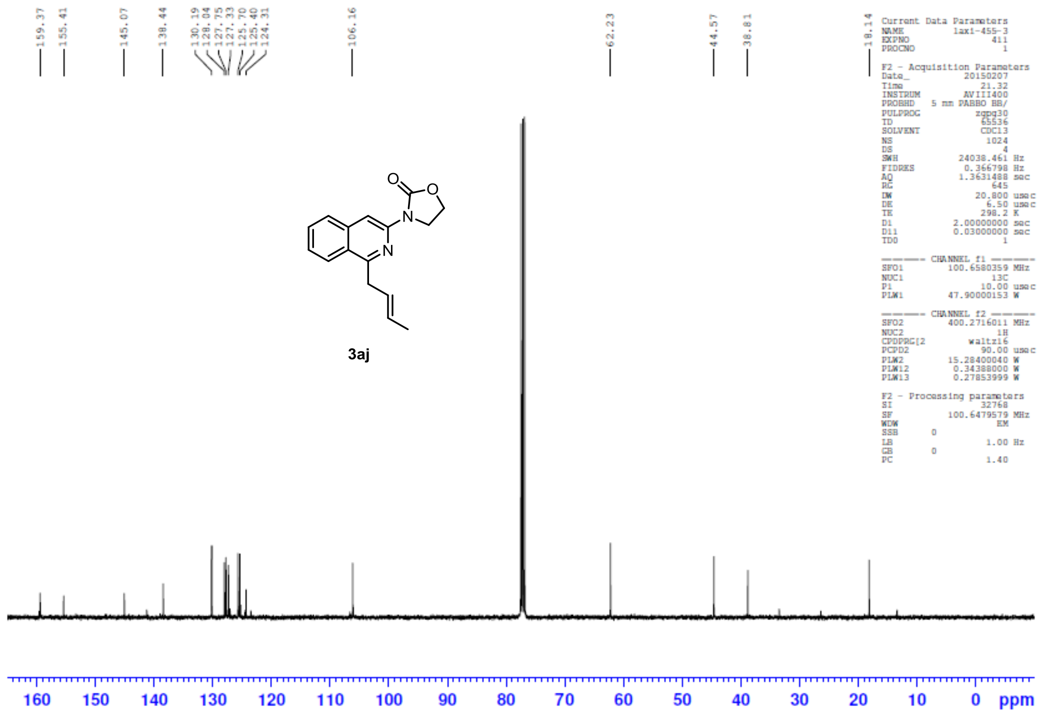

Supplementary Figure 22. ^{1}H NMR and ^{13}C NMR spectra of substrate **3aj**

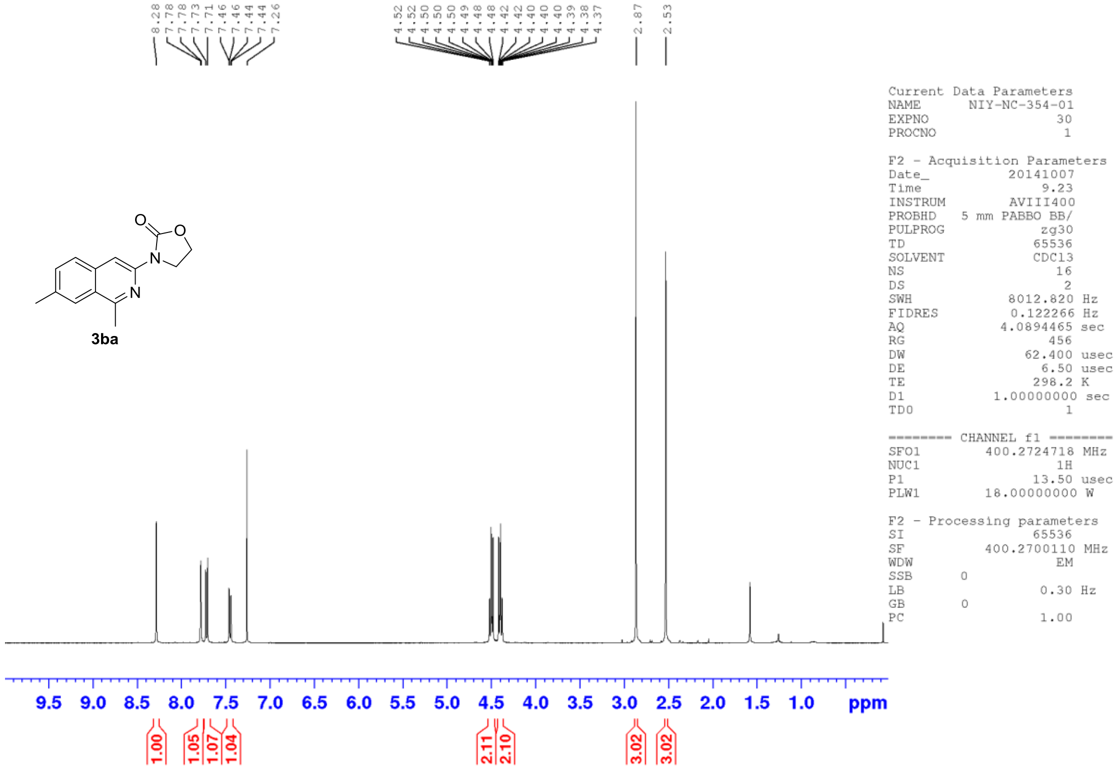

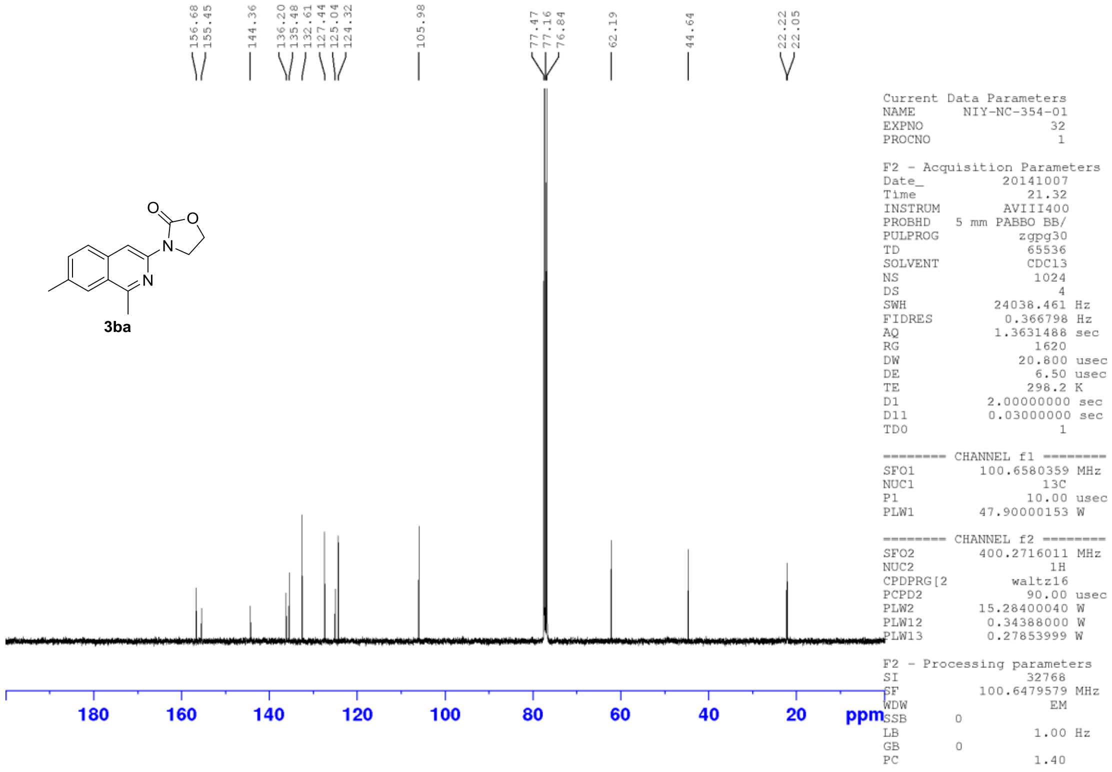

Supplementary Figure 23. ^{1}H NMR and ^{13}C NMR spectra of substrate **3ba**

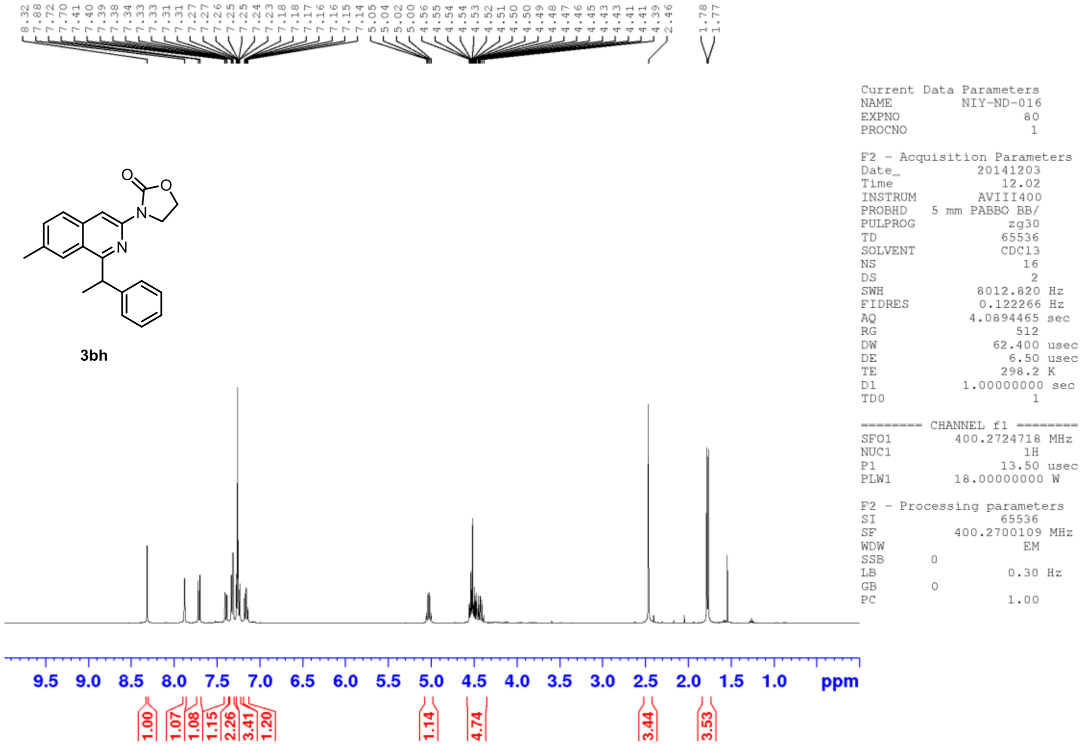

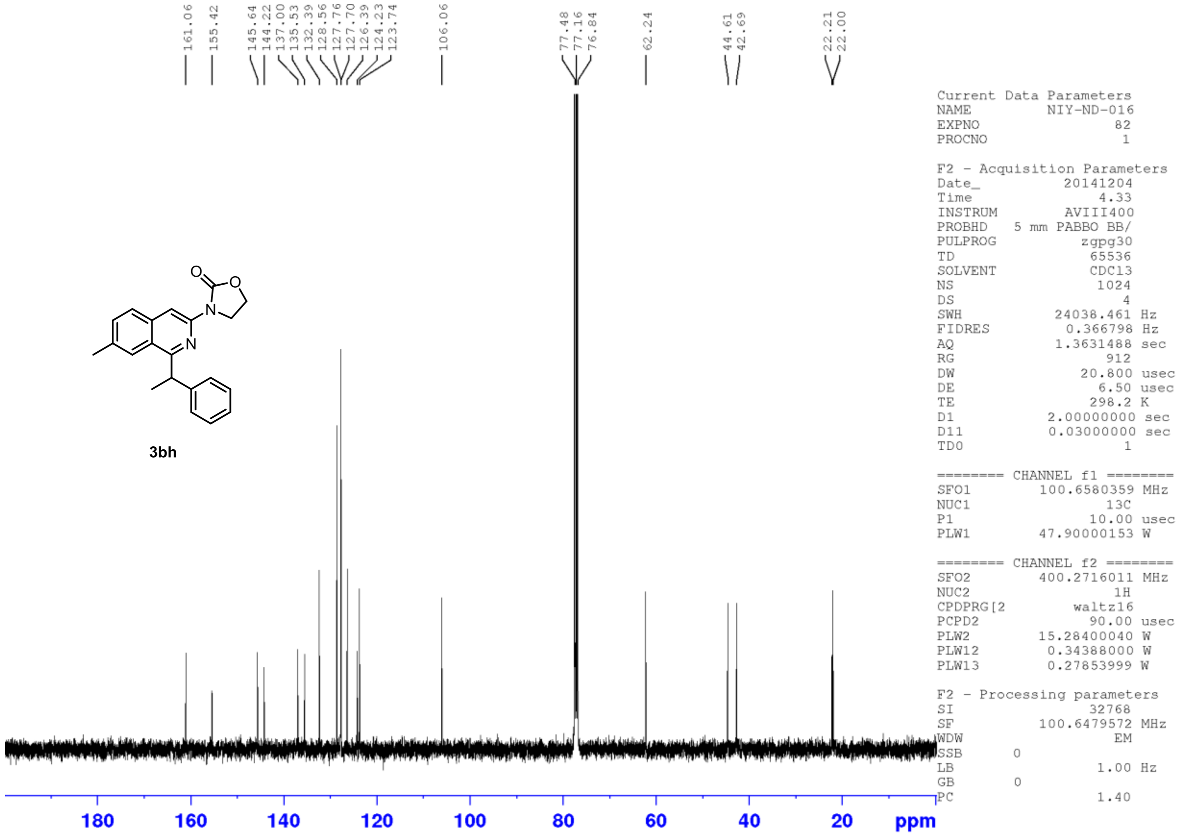

Supplementary Figure 24. ^{1}H NMR and ^{13}C NMR spectra of substrate **3bh**

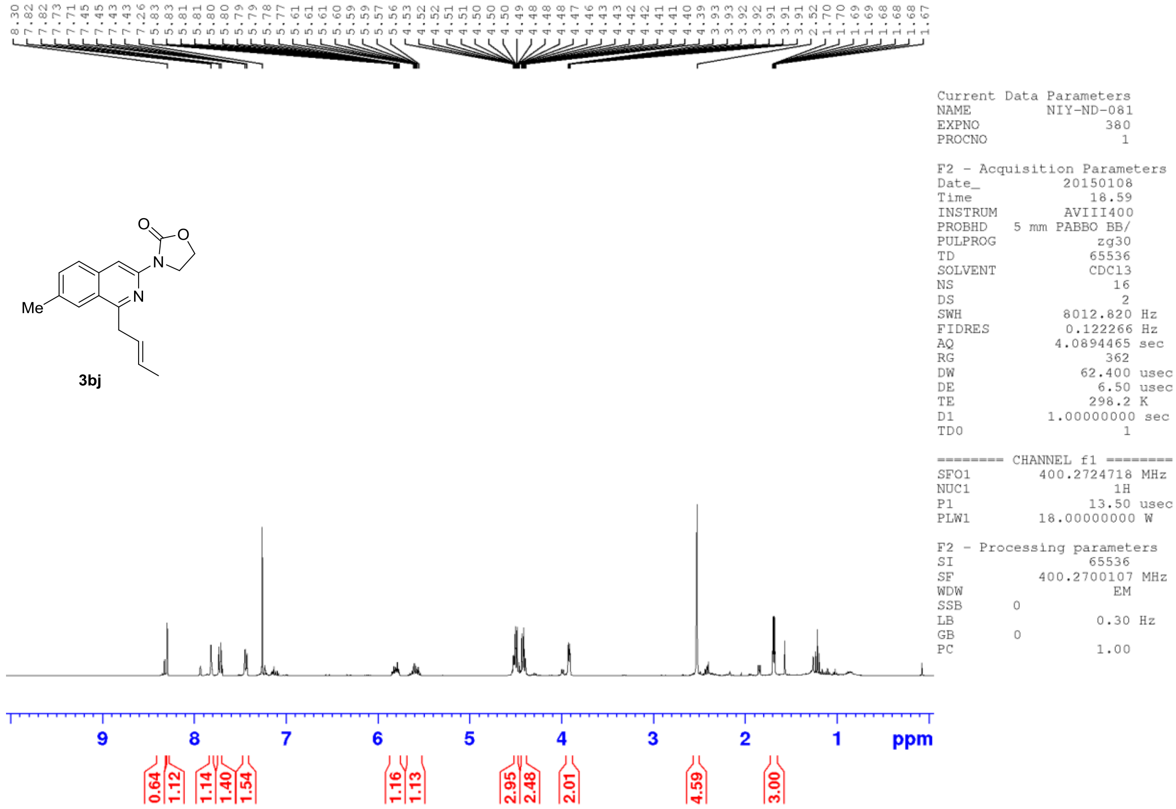

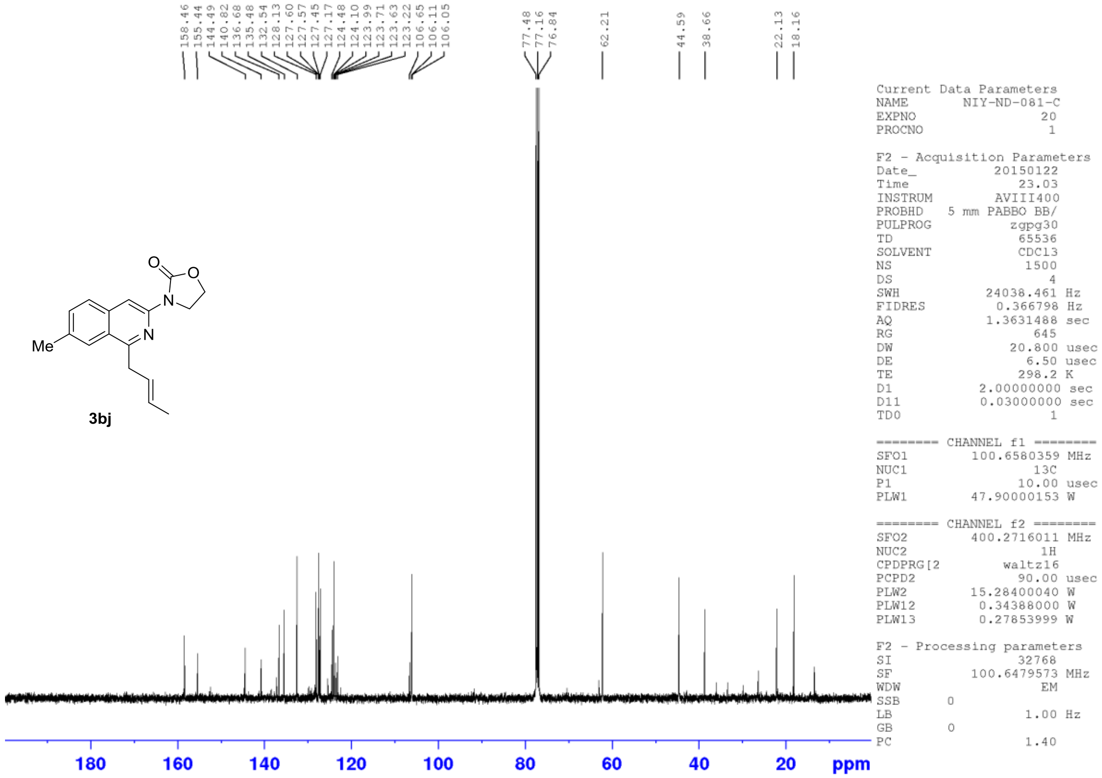

Supplementary Figure 25. ^{1}H NMR and ^{13}C NMR spectra of substrate **3bj**

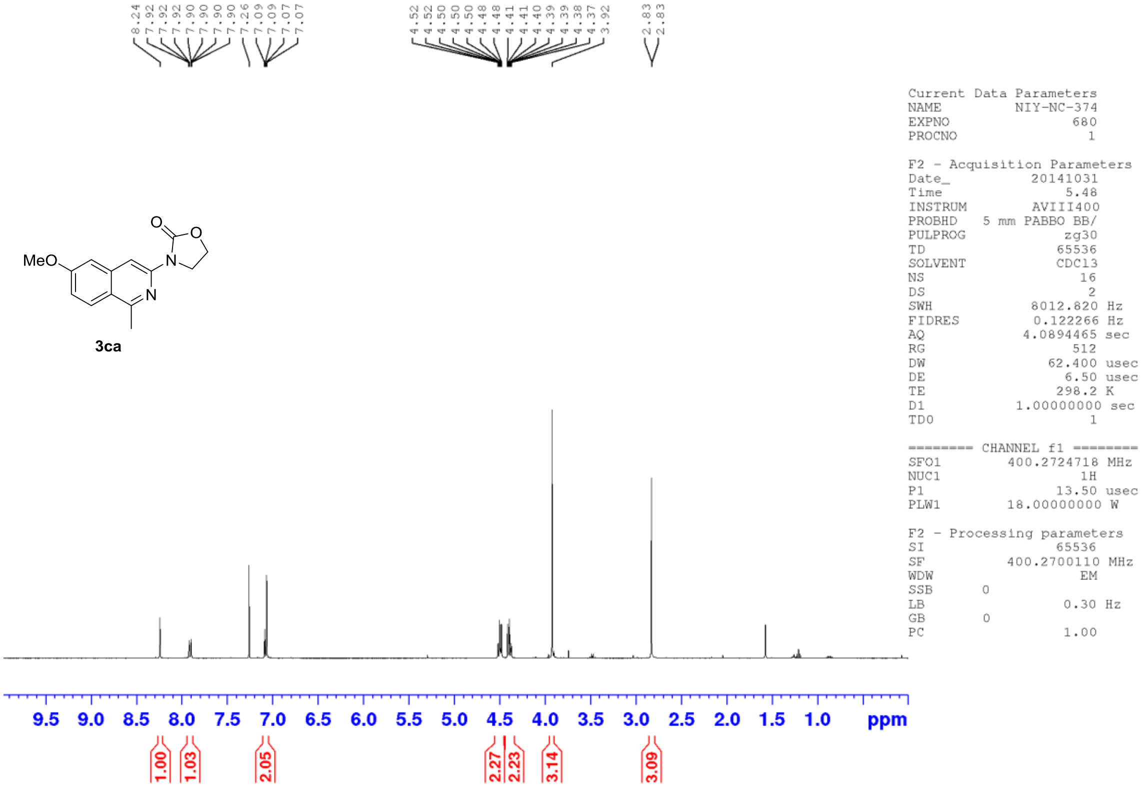

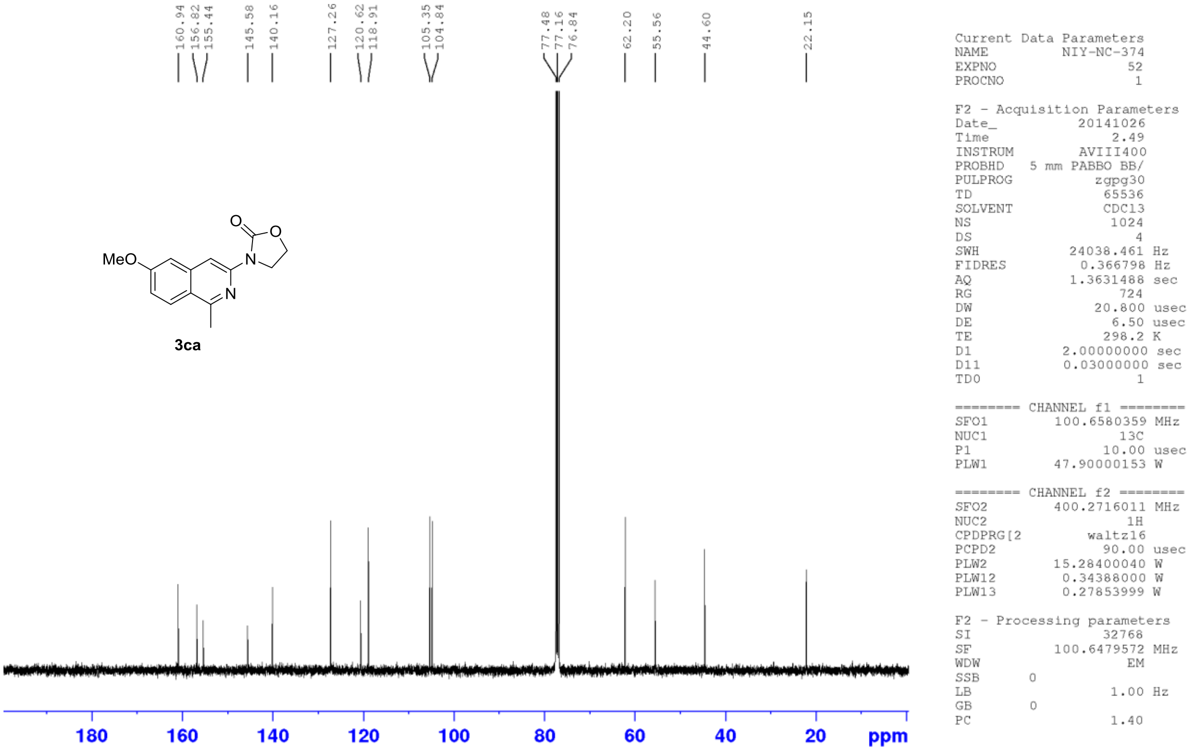

Supplementary Figure 26. ^{1}H NMR and ^{13}C NMR spectra of substrate **3ca**

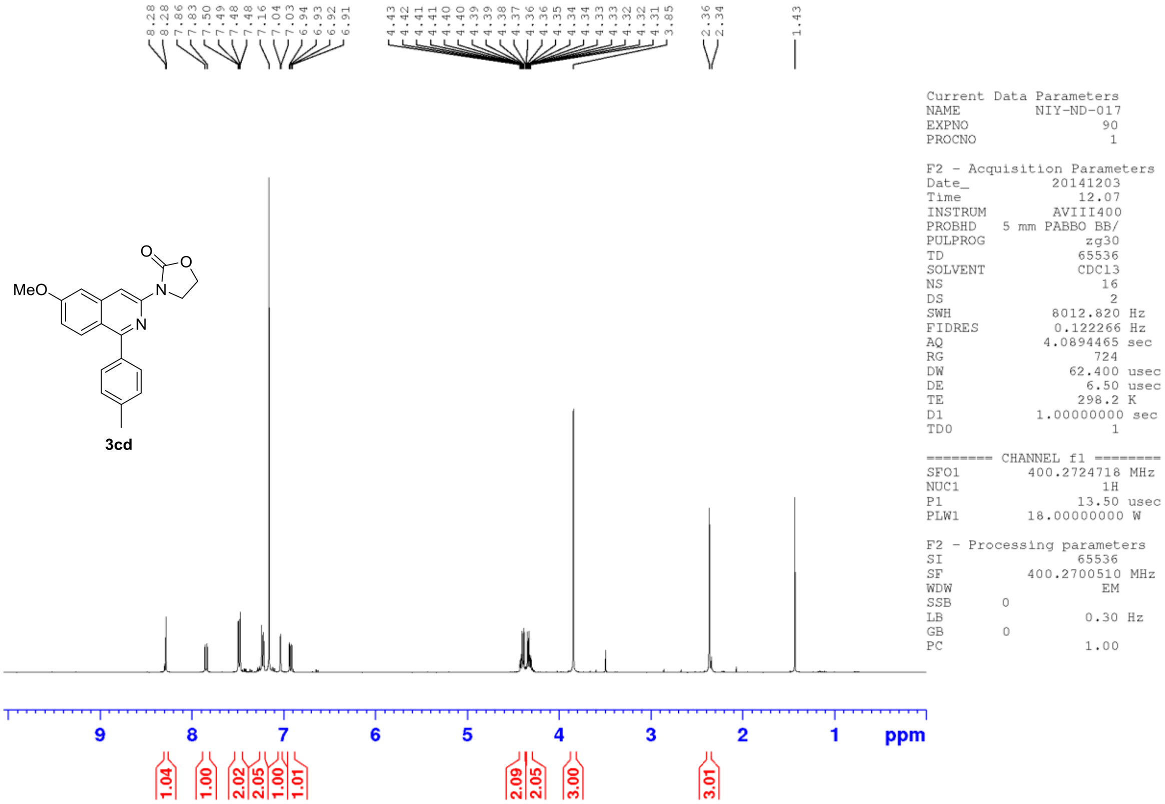

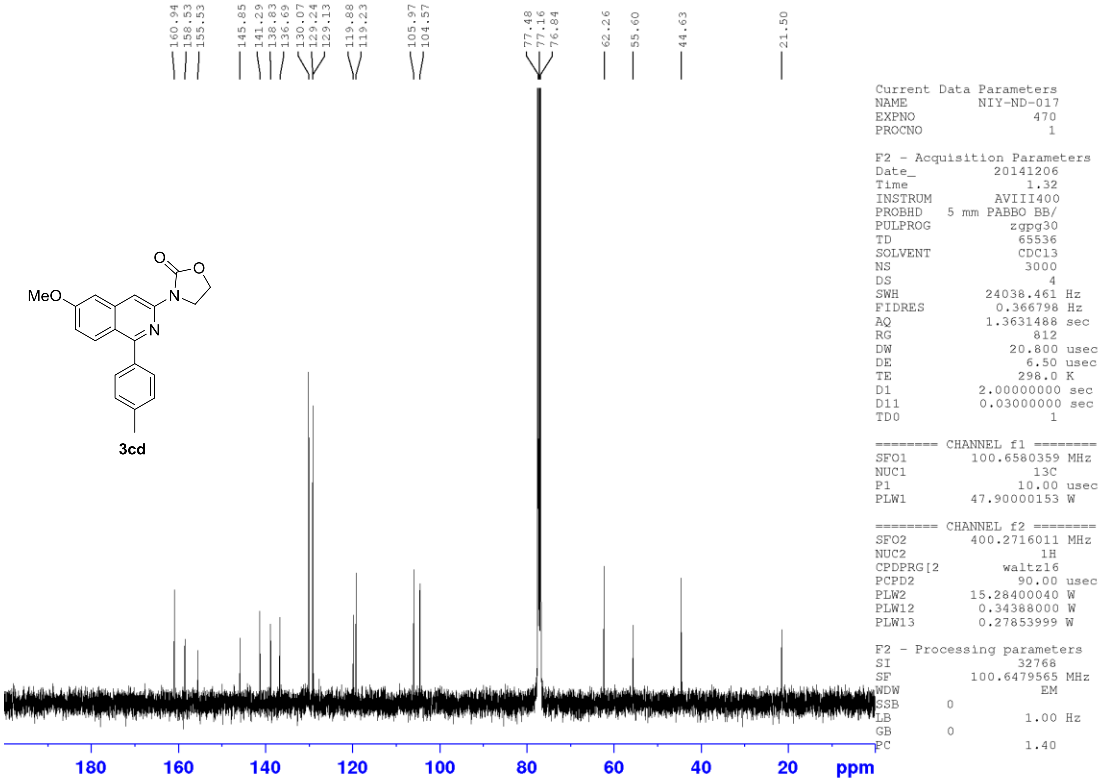

Supplementary Figure 27. ^{1}H NMR and ^{13}C NMR spectra of substrate **3cd**

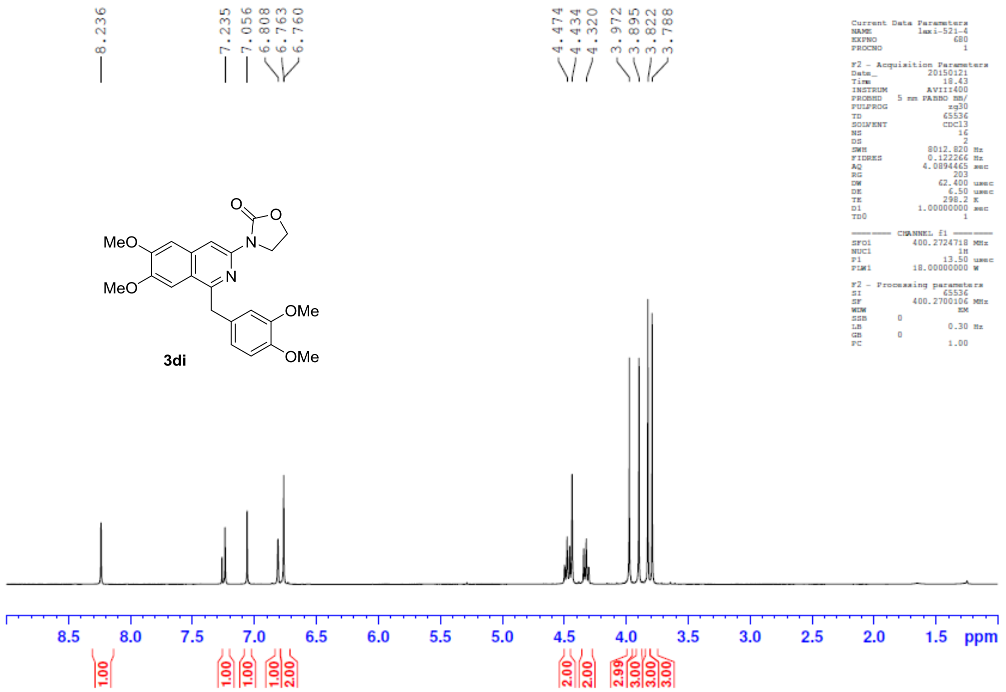

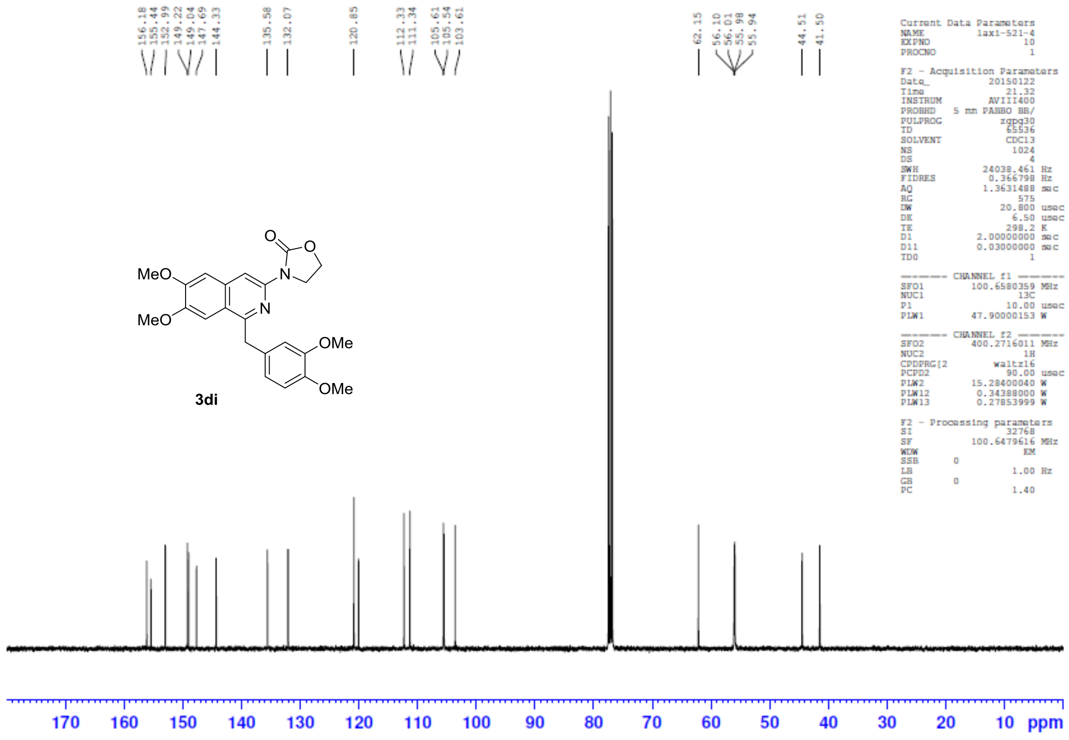

Supplementary Figure 28. ^{1}H NMR and ^{13}C NMR spectra of substrate **3di**

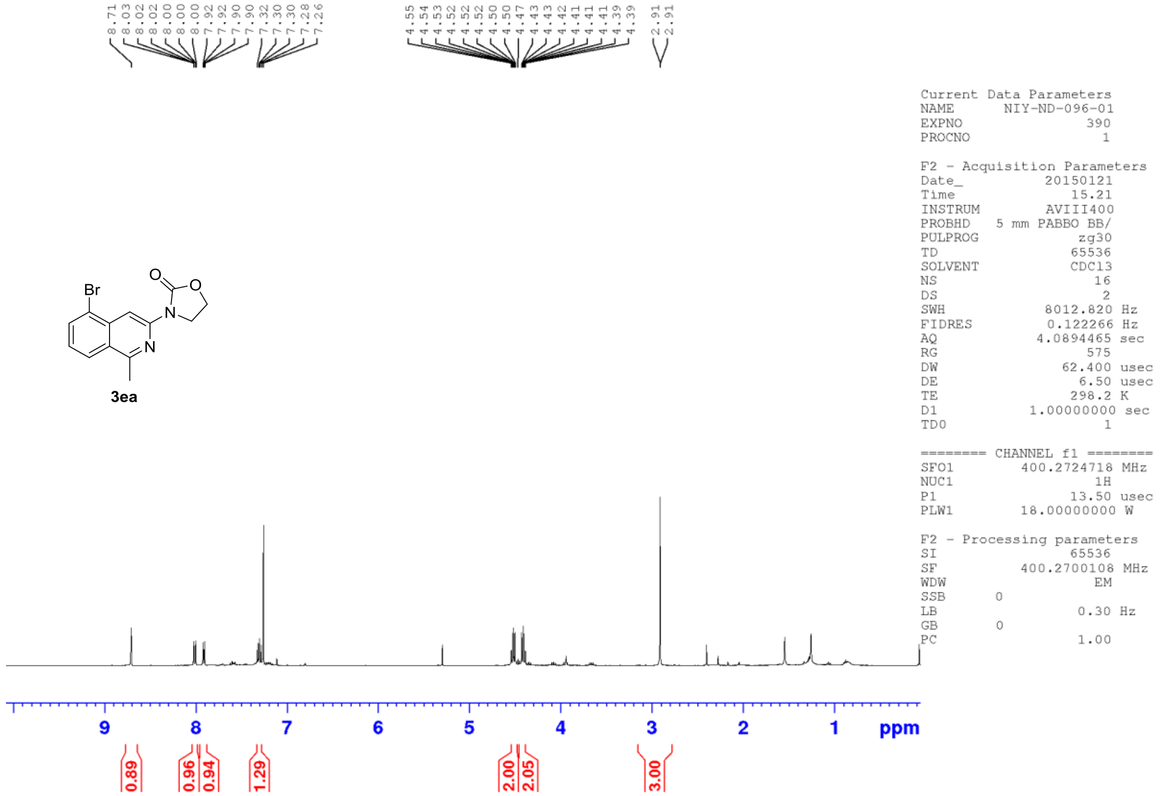

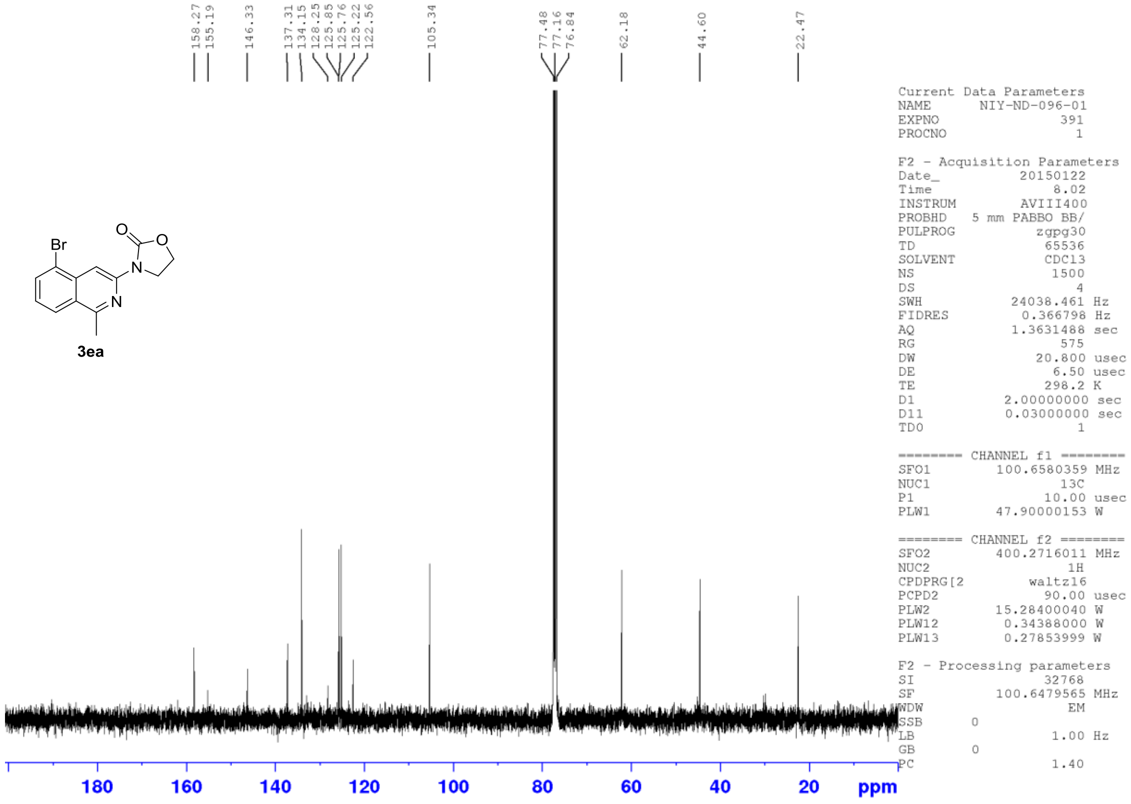

Supplementary Figure 29. ^{1}H NMR and ^{13}C NMR spectra of substrate **3ea**

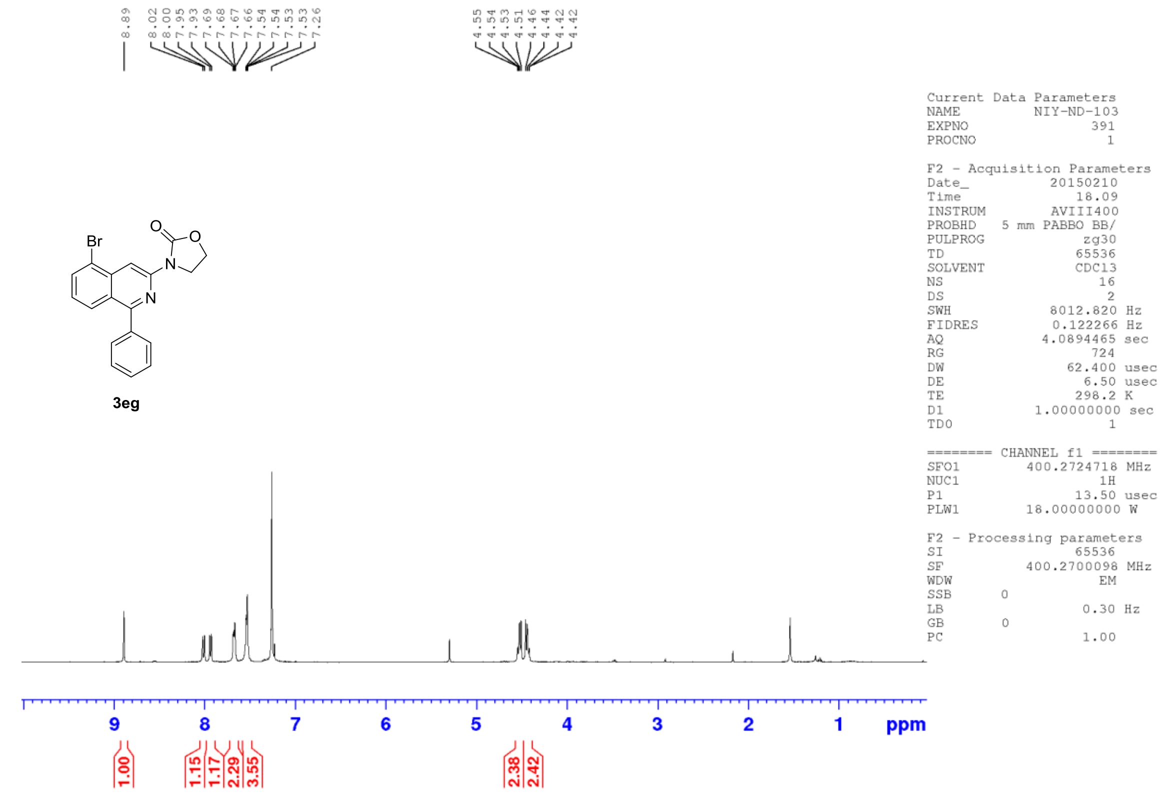

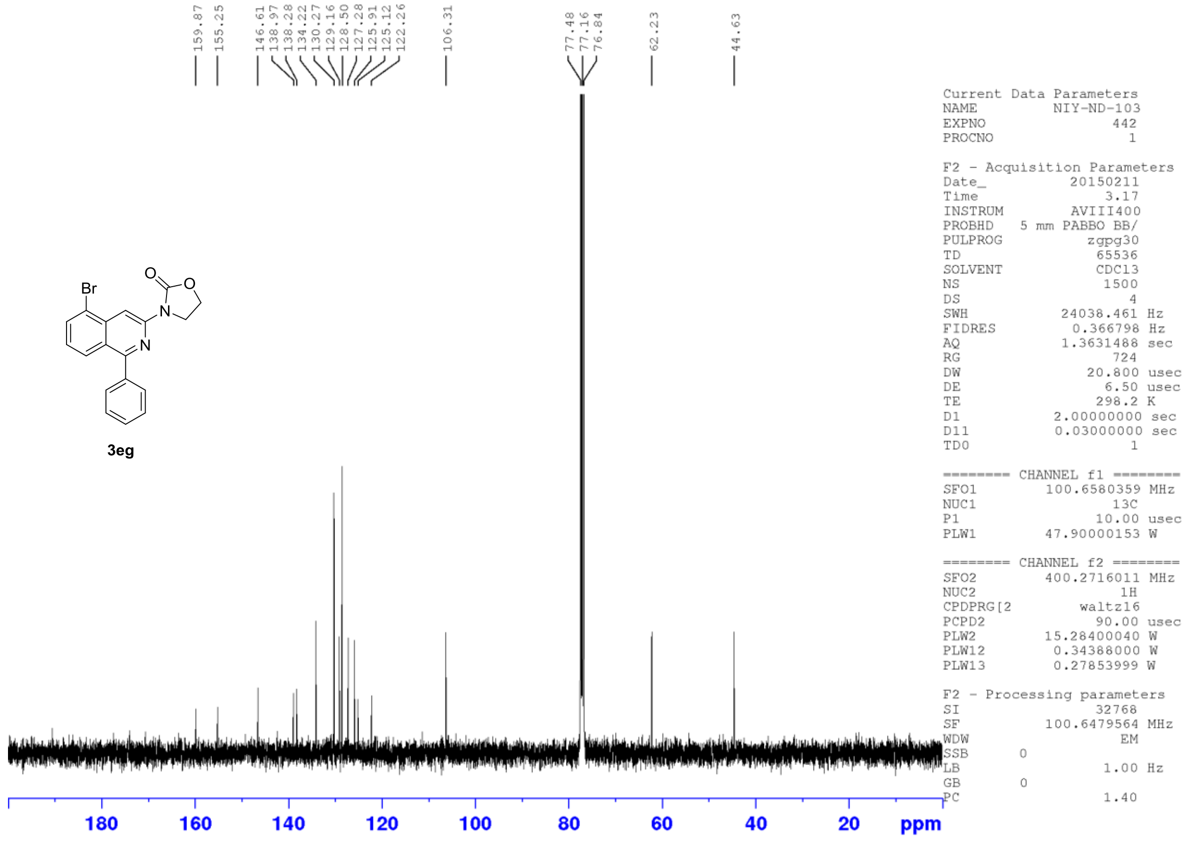

Supplementary Figure 30. ^{1}H NMR and ^{13}C NMR spectra of substrate **3eg**

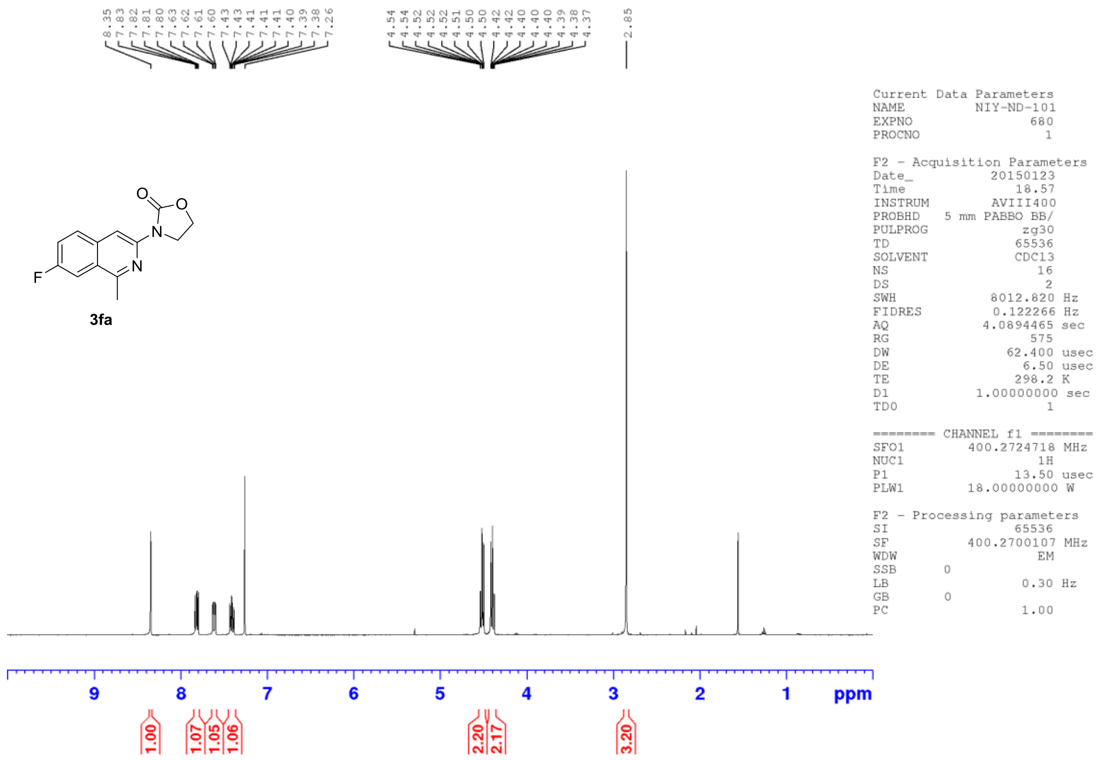

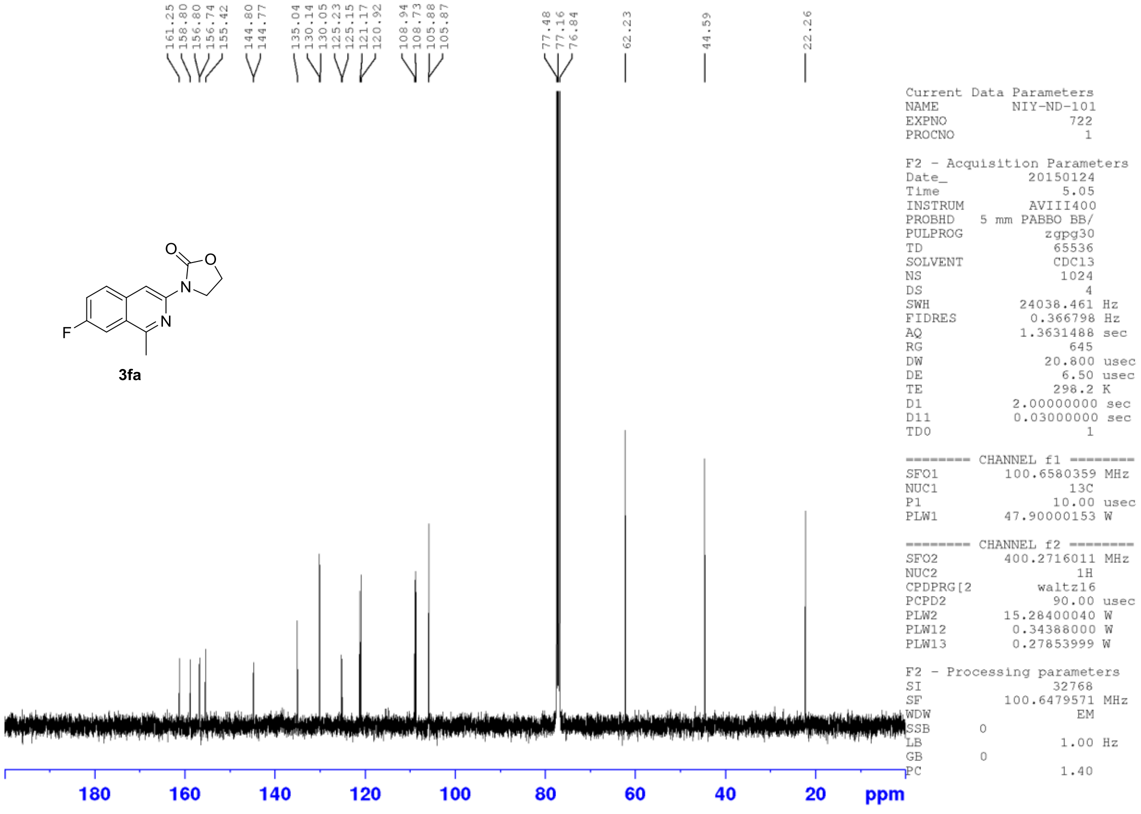

Supplementary Figure 31. ^{1}H NMR and ^{13}C NMR spectra of substrate **3fa**

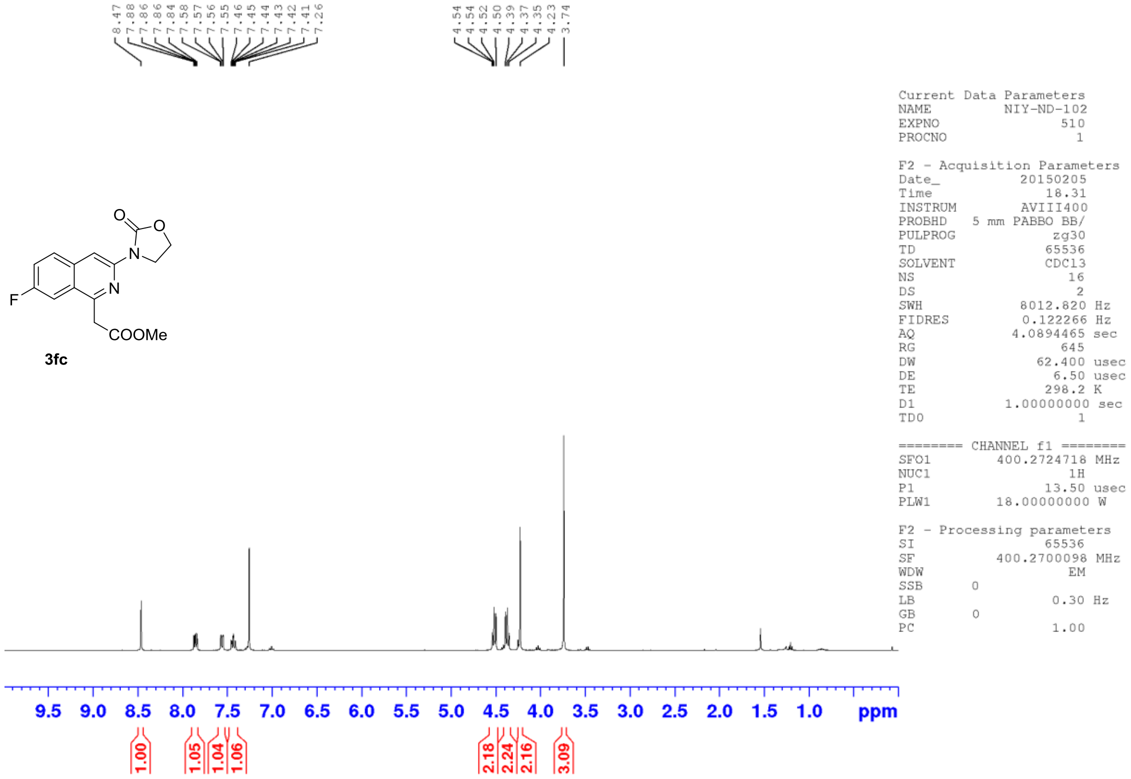

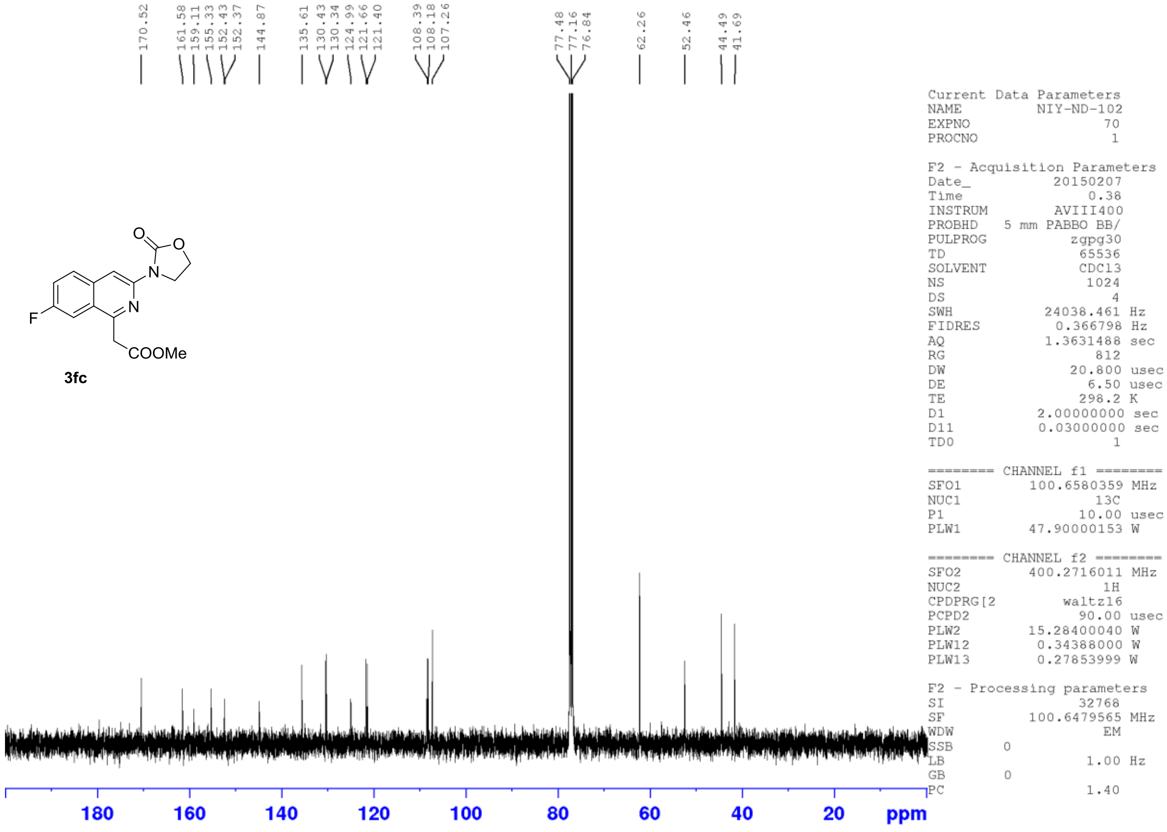

Supplementary Figure 32. ^{1}H NMR and ^{13}C NMR spectra of substrate **3fc**

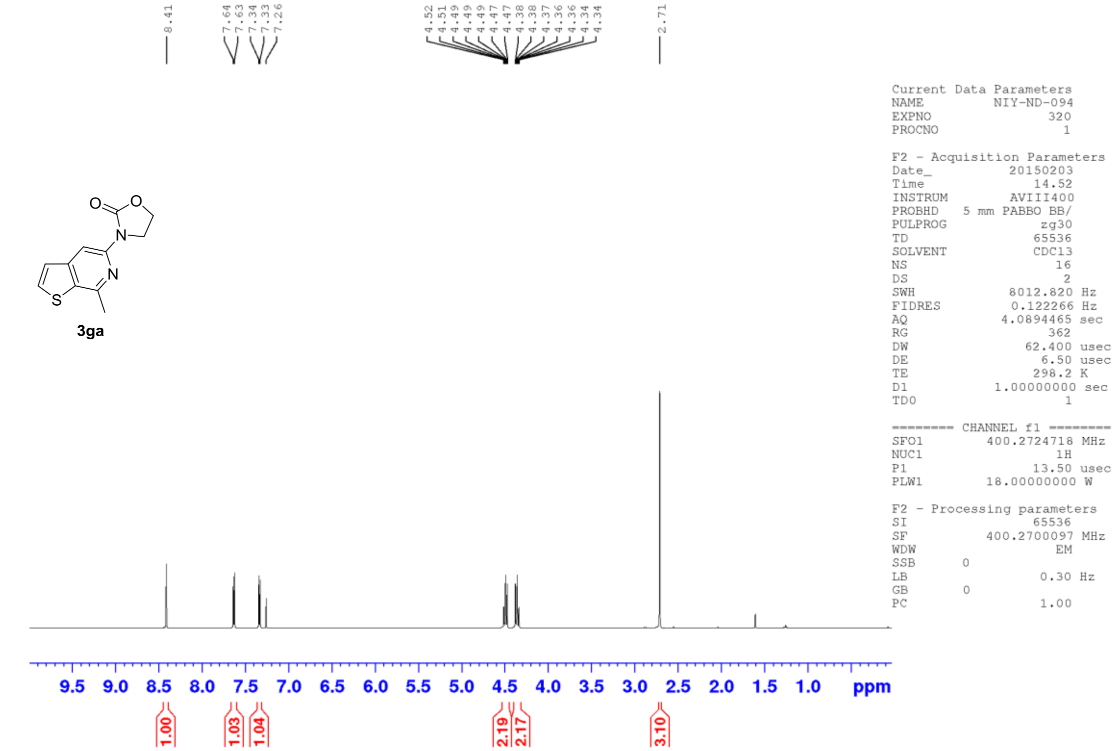

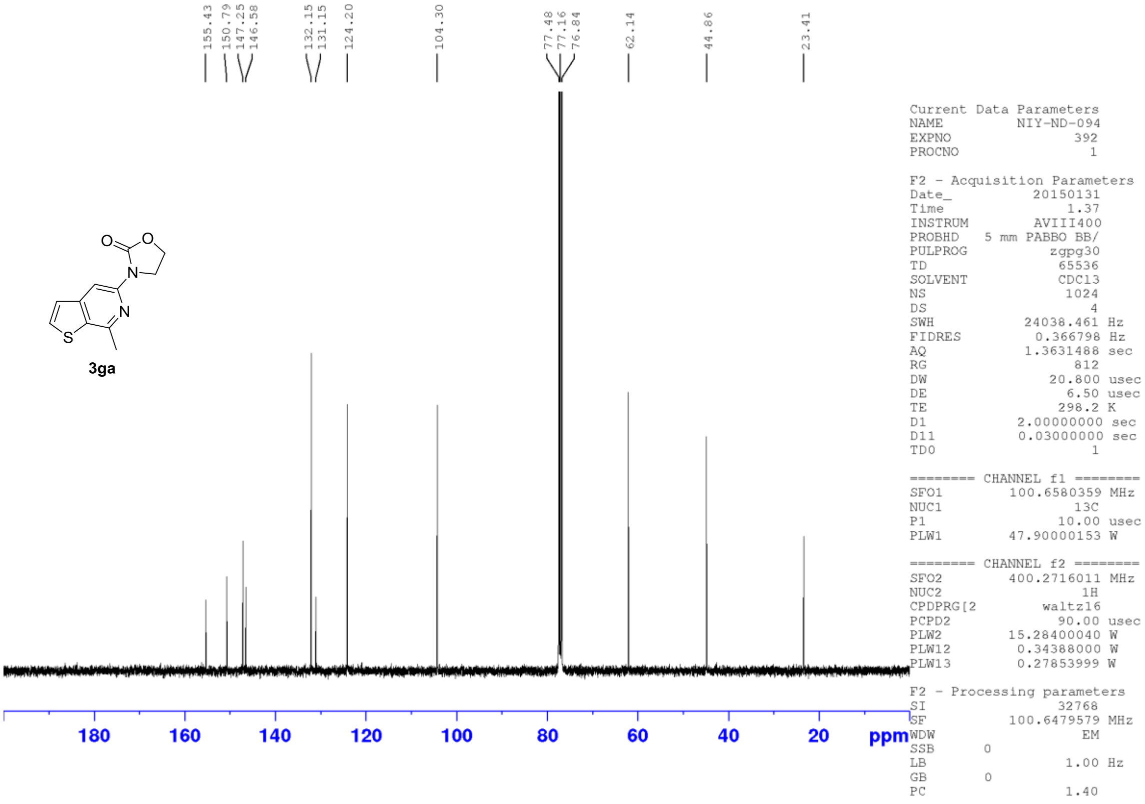

Supplementary Figure 33. ^{1}H NMR and ^{13}C NMR spectra of substrate **3ga**

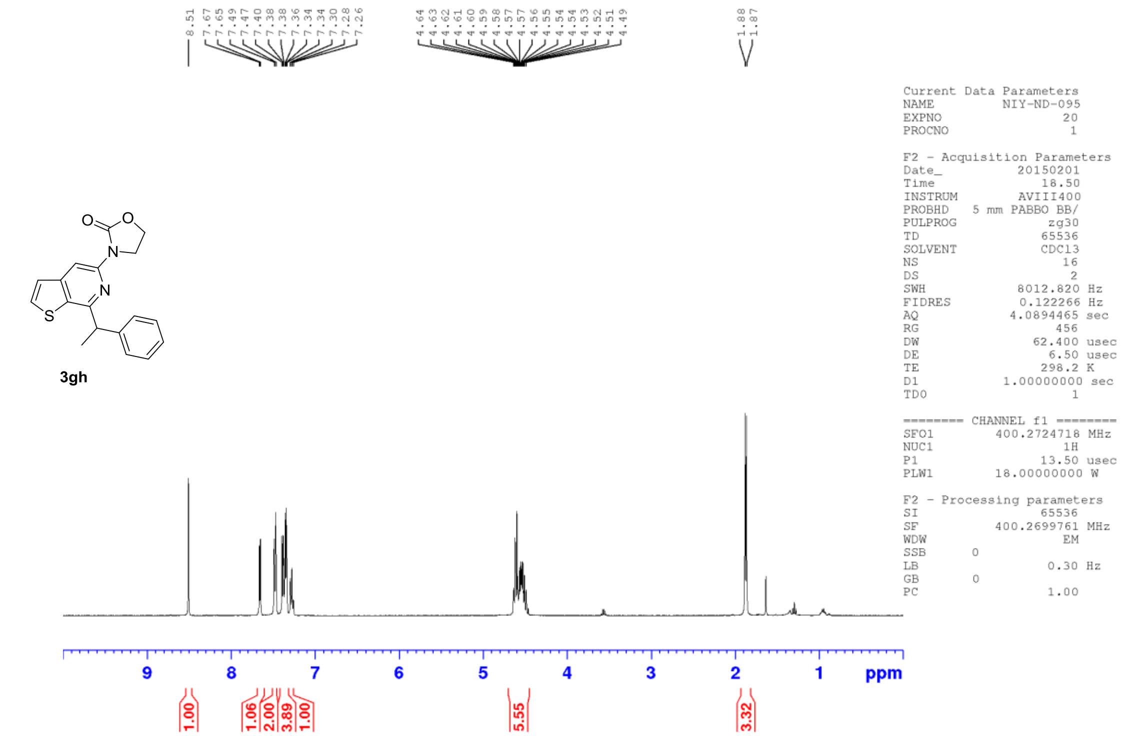

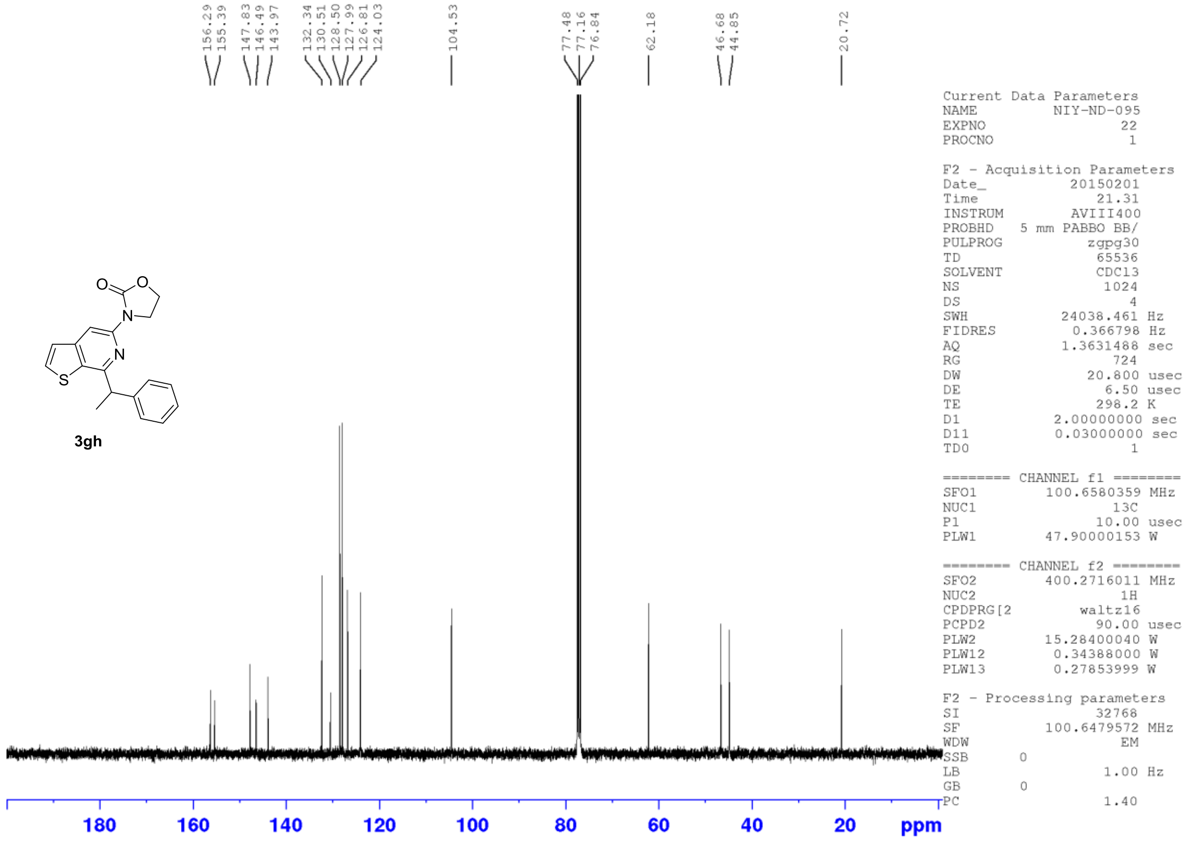

Supplementary Figure 34. ^{1}H NMR and ^{13}C NMR spectra of substrate **3gh**

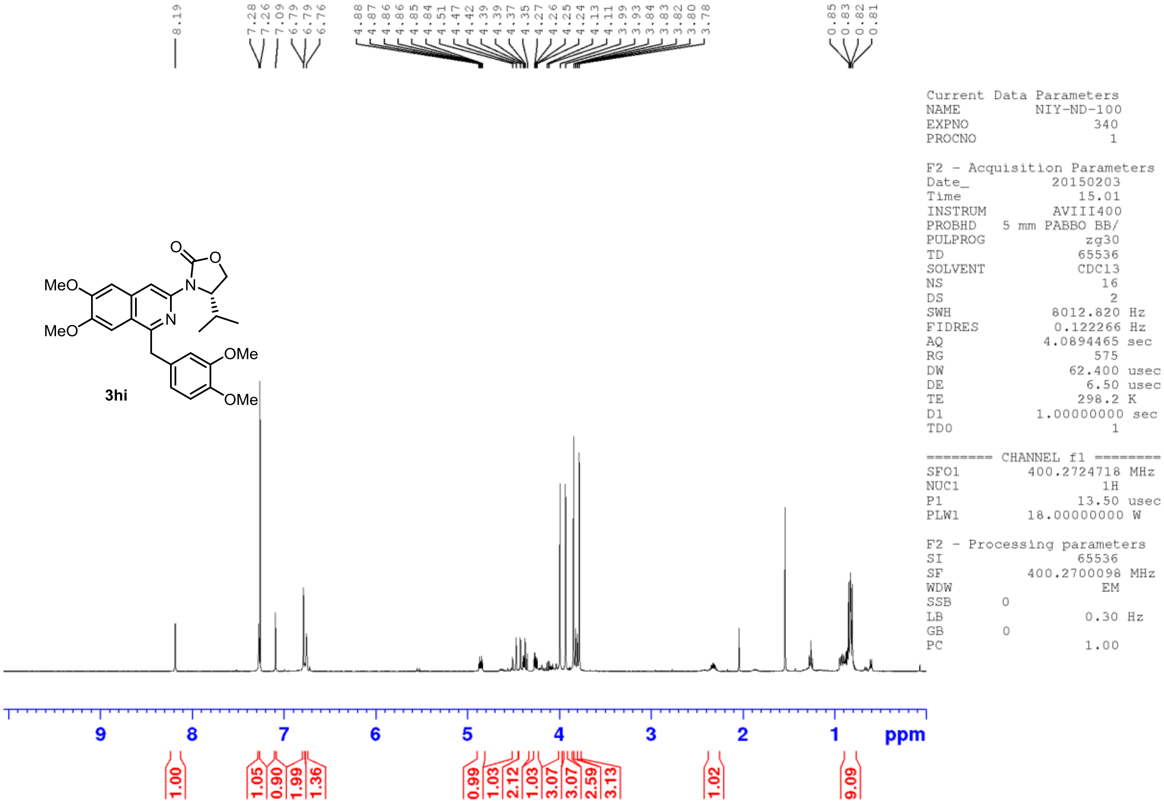

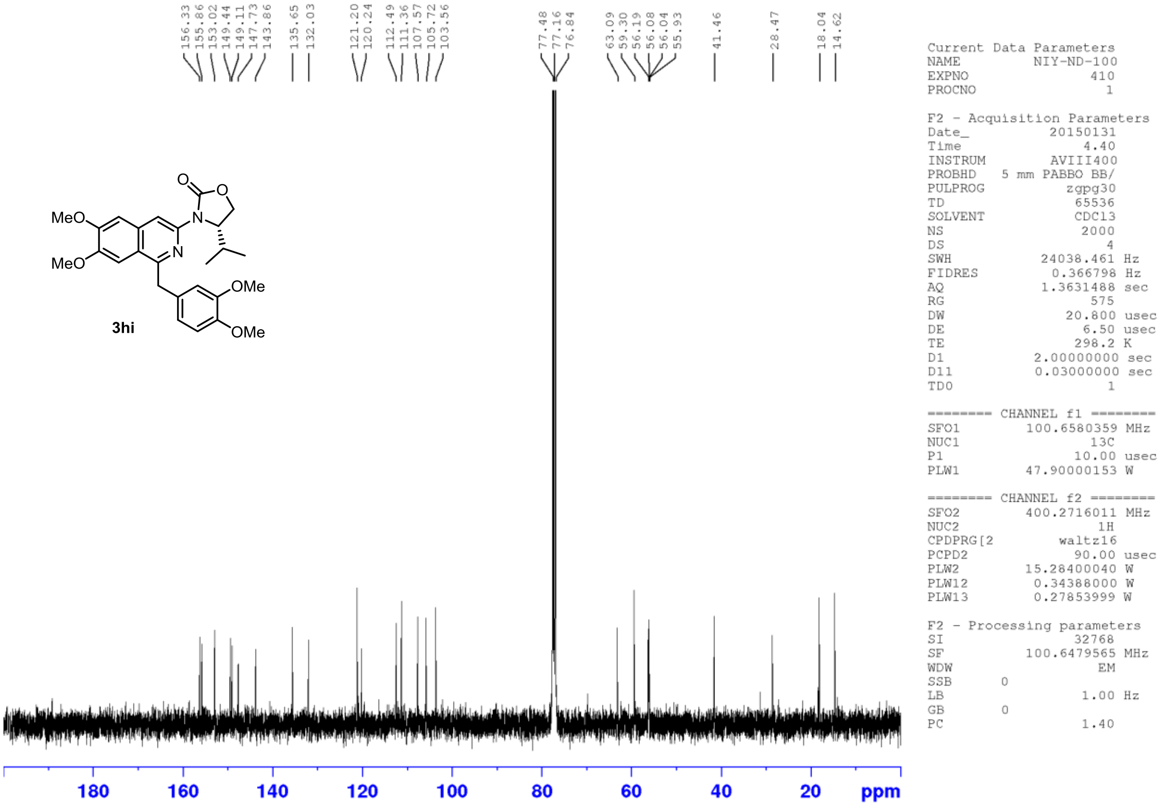

Supplementary Figure 35. ^{1}H NMR and ^{13}C NMR spectra of substrate **3hi**

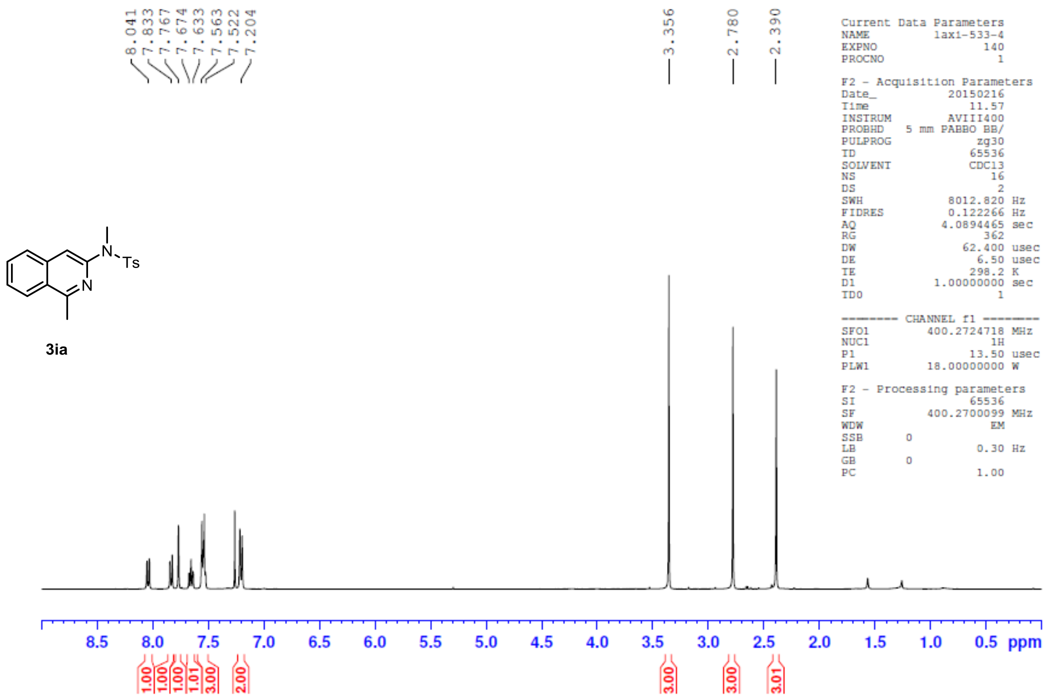

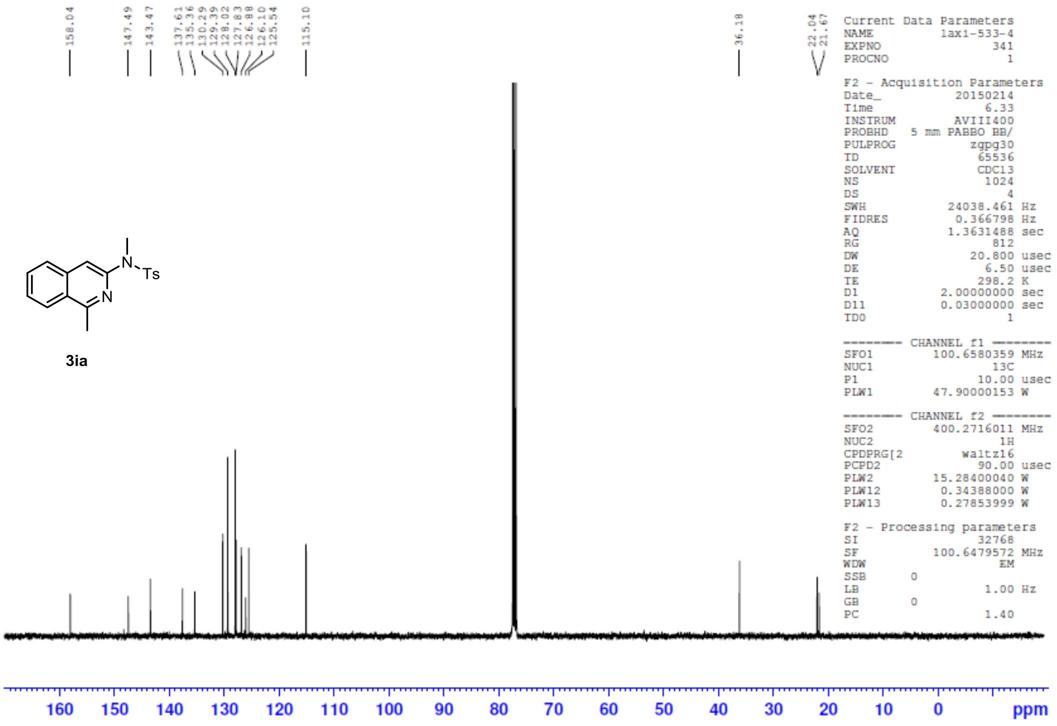

Supplementary Figure 36. ^{1}H NMR and ^{13}C NMR spectra of substrate **3ia**

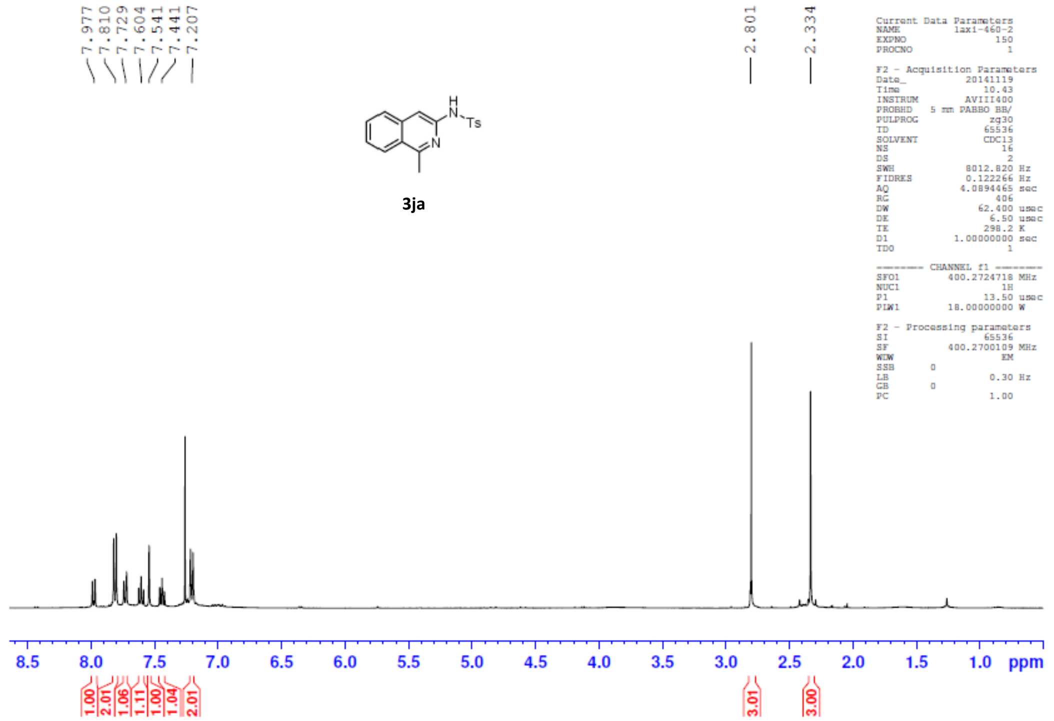

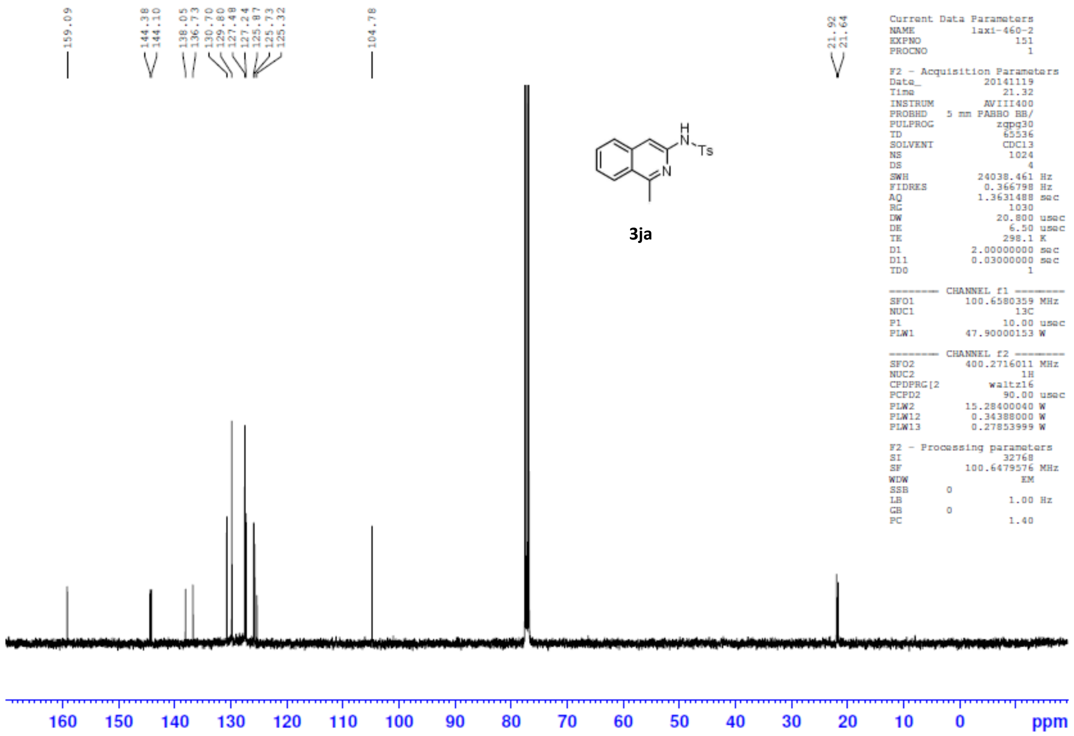

Supplementary Figure 37. ^{1}H NMR and ^{13}C NMR spectra of substrate **3ja**

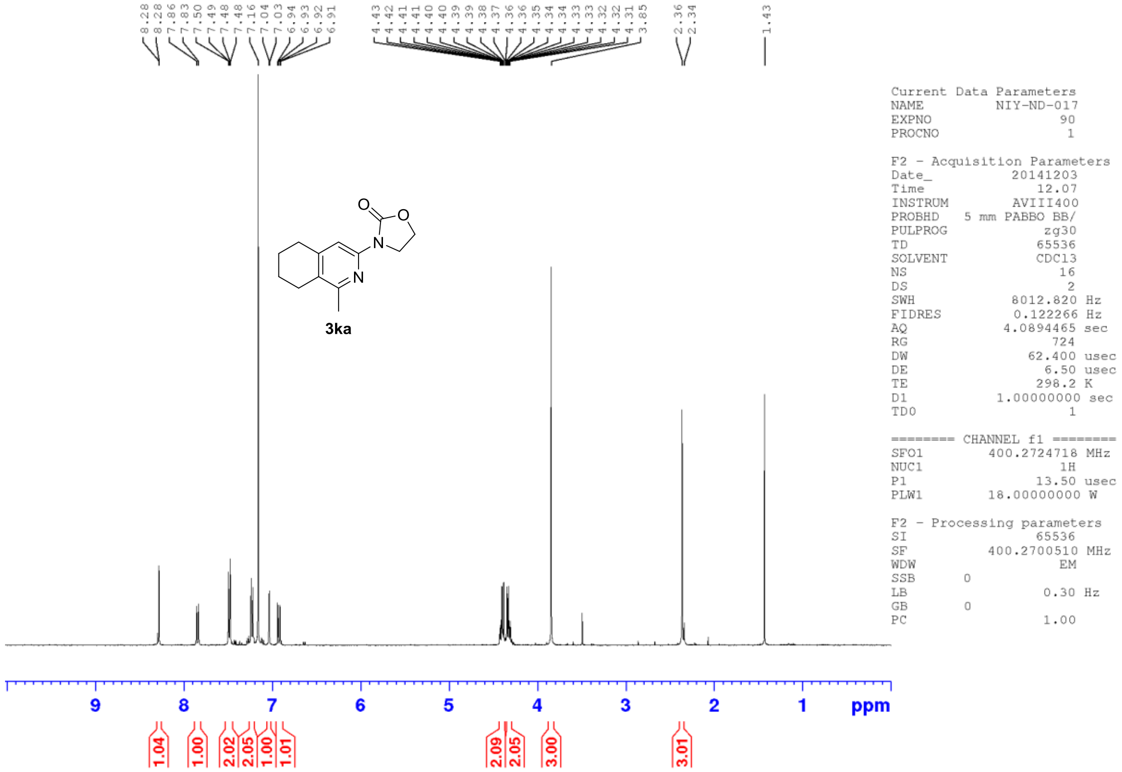

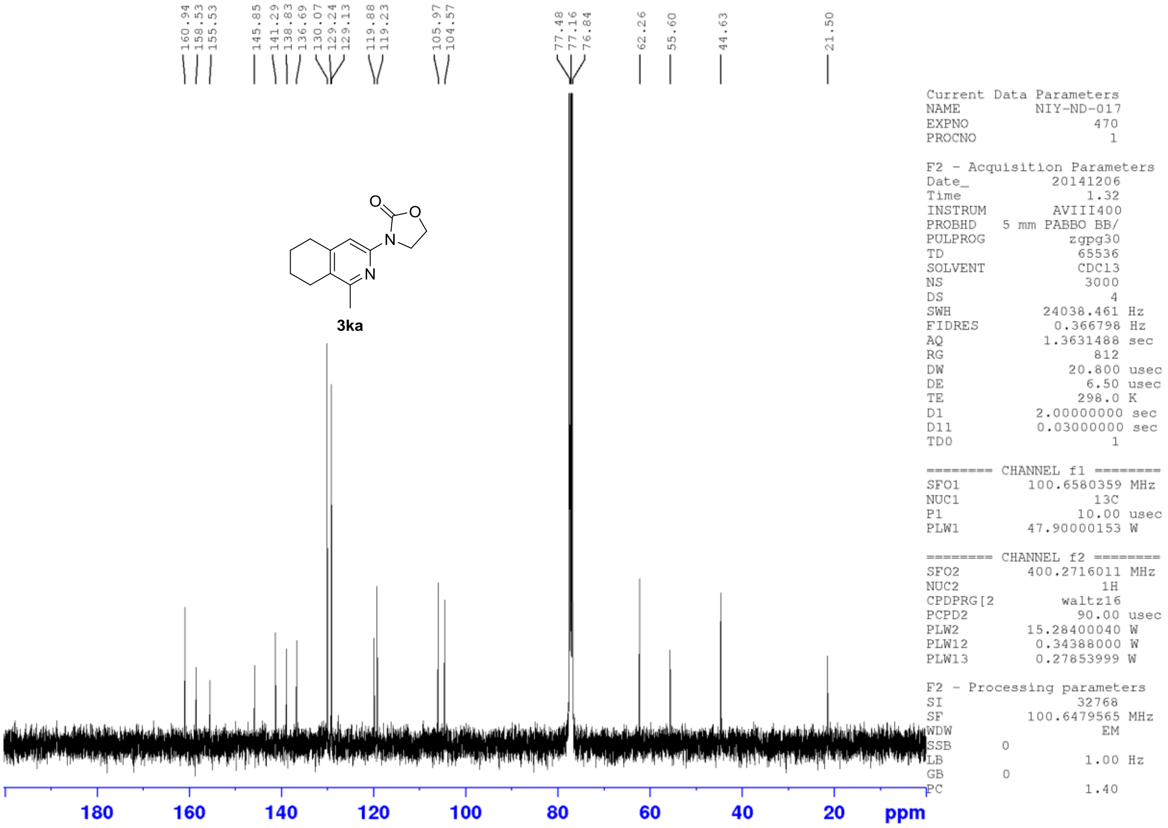

Supplementary Figure 38. ^{1}H NMR and ^{13}C NMR spectra of substrate **3ka**

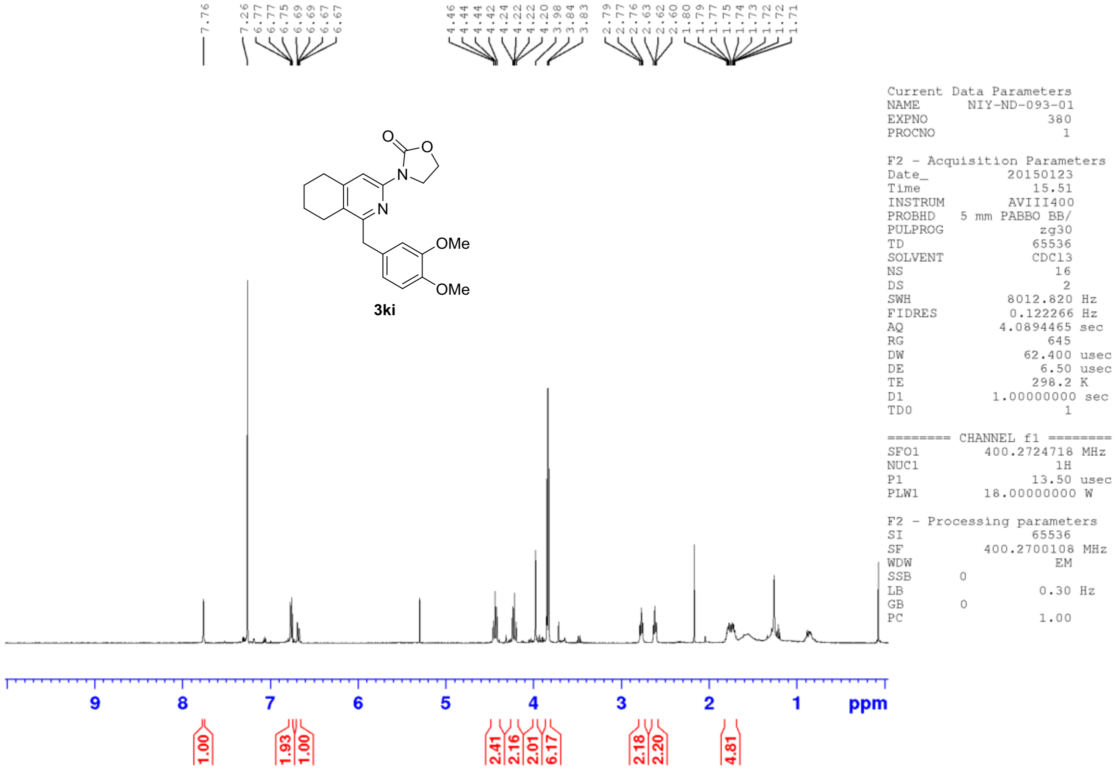

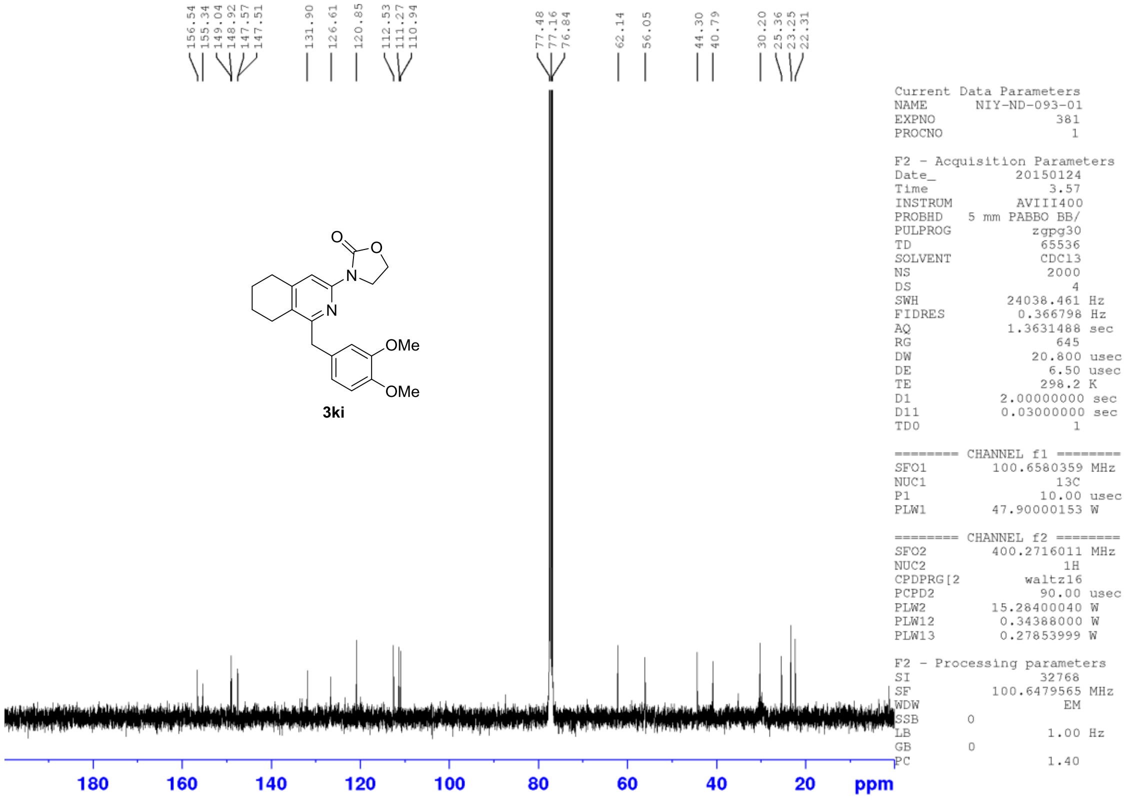

Supplementary Figure 39. ^{1}H NMR and ^{13}C NMR spectra of substrate **3ki**

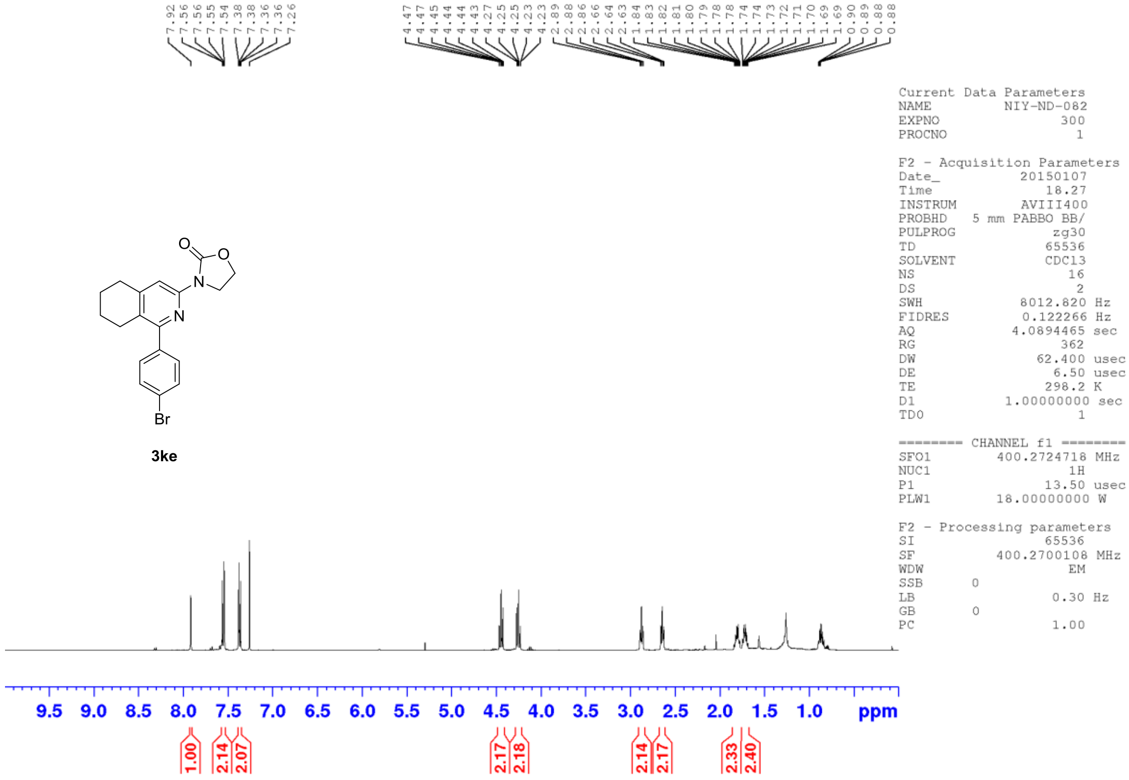

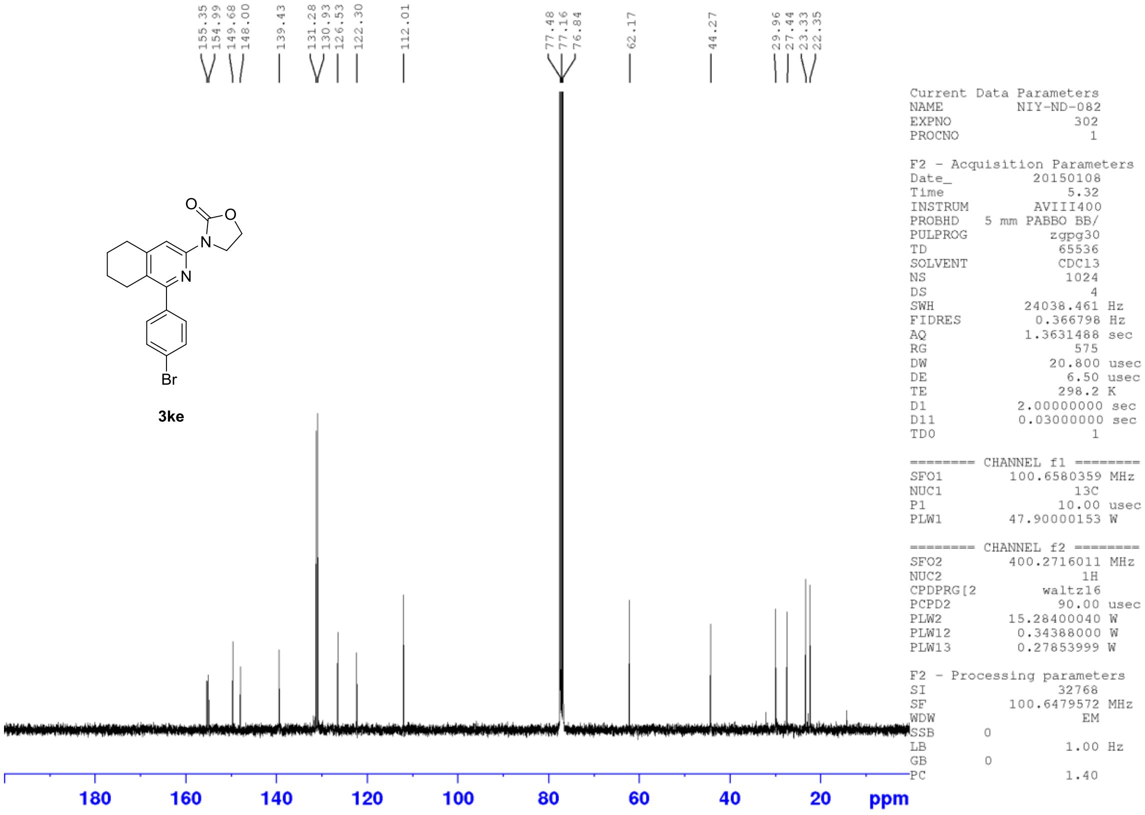

Supplementary Figure 40. ^{1}H NMR and ^{13}C NMR spectra of substrate **3ke**

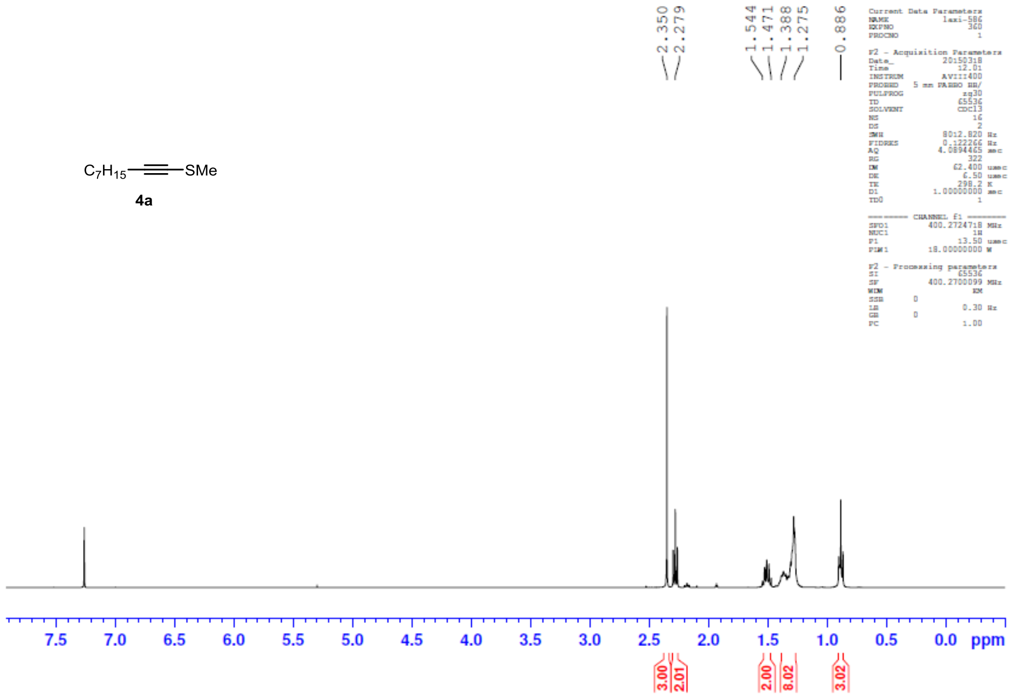

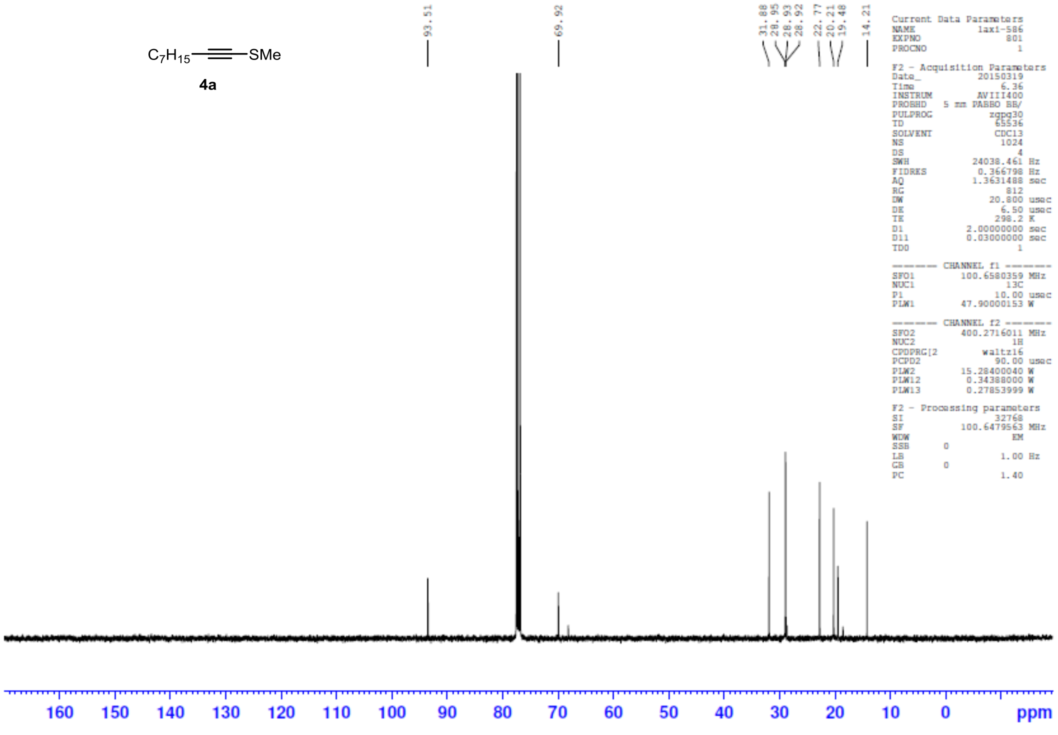

Supplementary Figure 41. ^{1}H NMR and ^{13}C NMR spectra of substrate **4a**

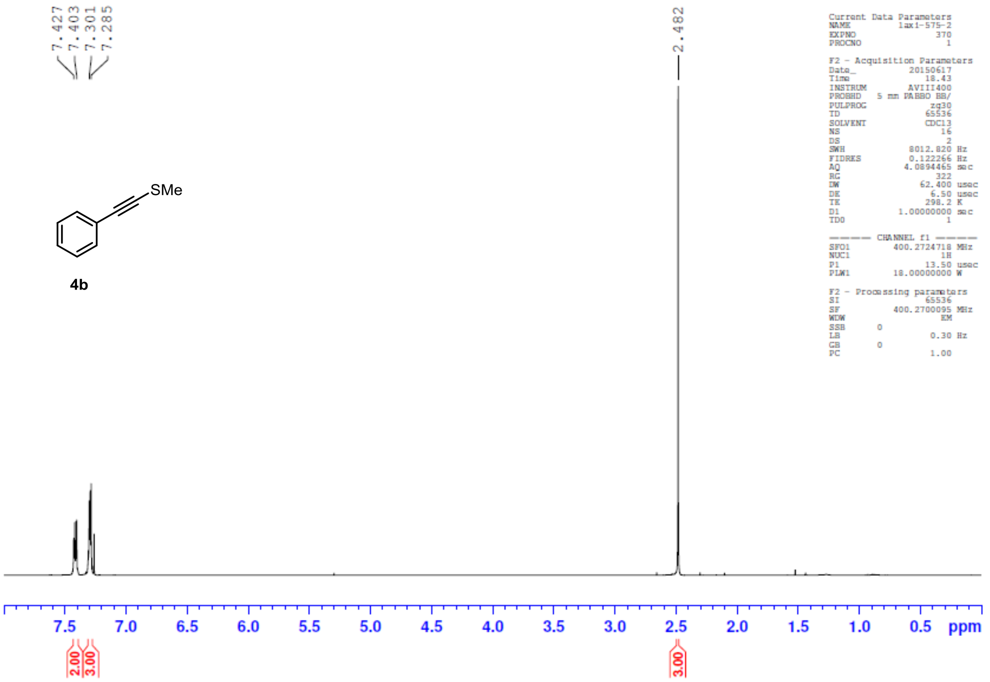

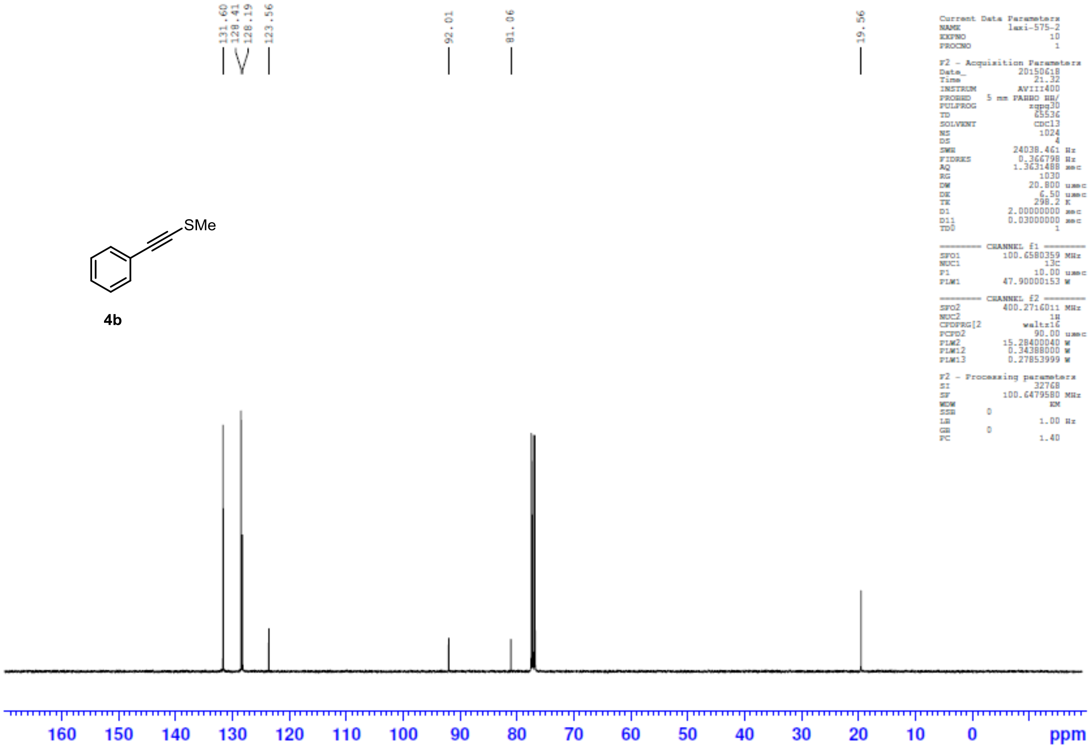

Supplementary Figure 42. ^{1}H NMR and ^{13}C NMR spectra of substrate **4b**

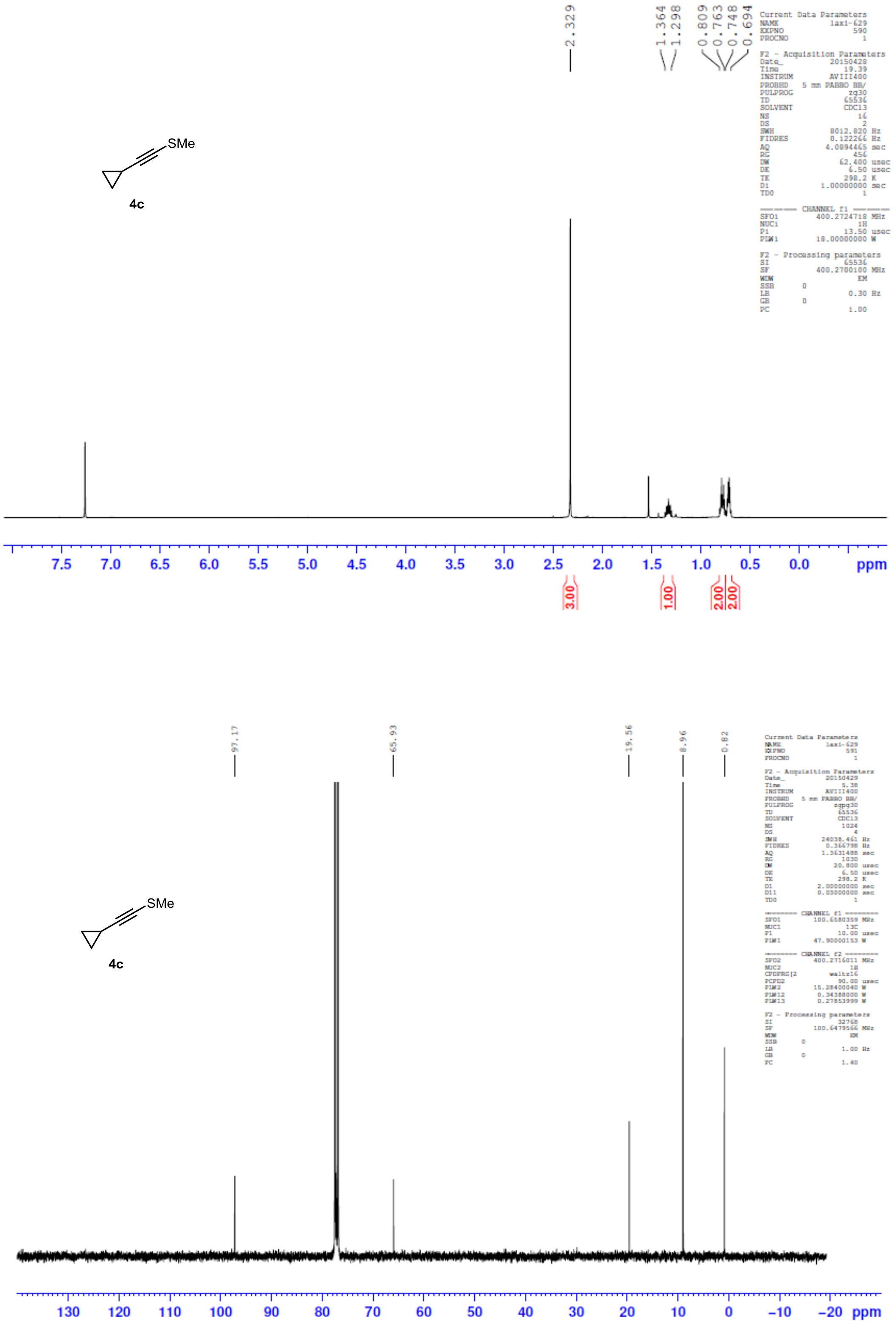

Supplementary Figure 43. ^{1}H NMR and ^{13}C NMR spectra of substrate **4c**

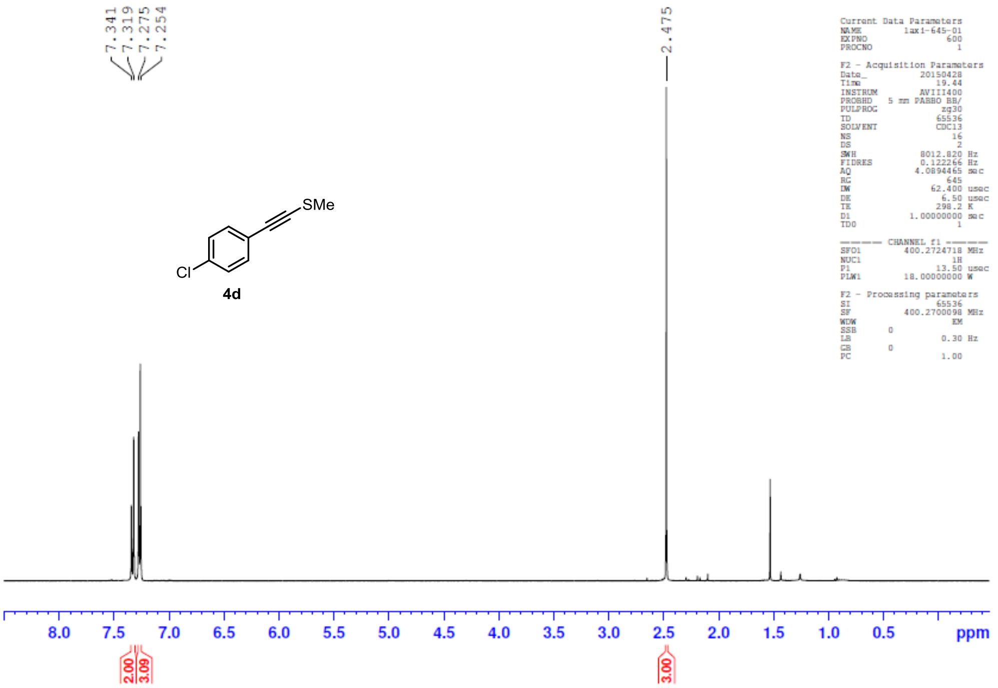

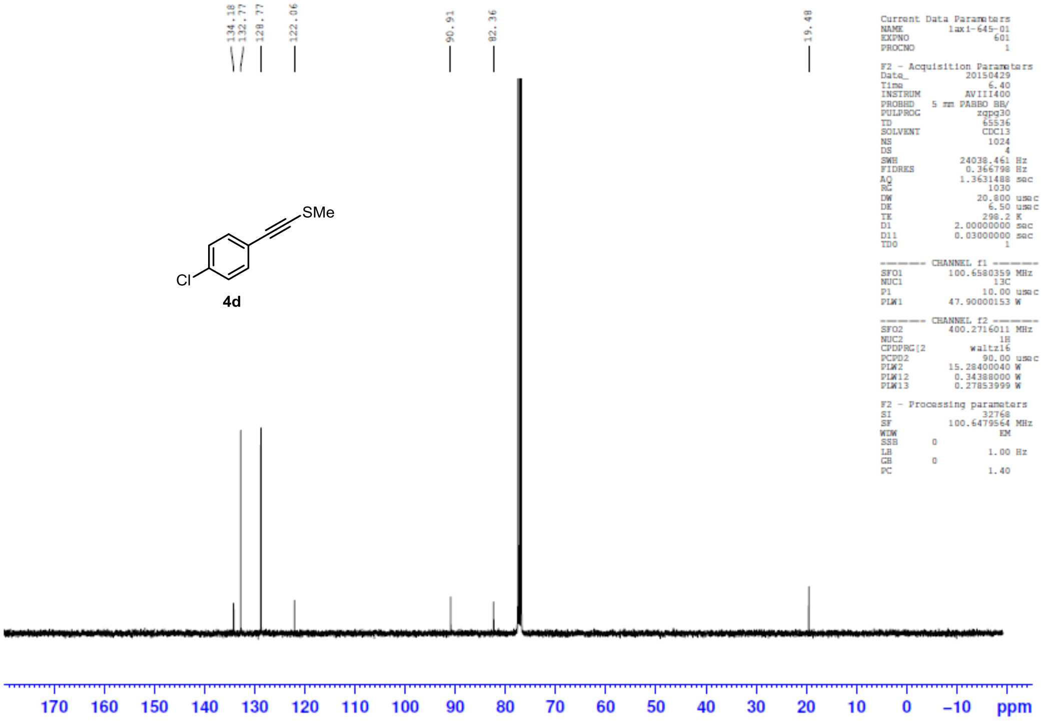

Supplementary Figure 44. ^{1}H NMR and ^{13}C NMR spectra of substrate **4d**

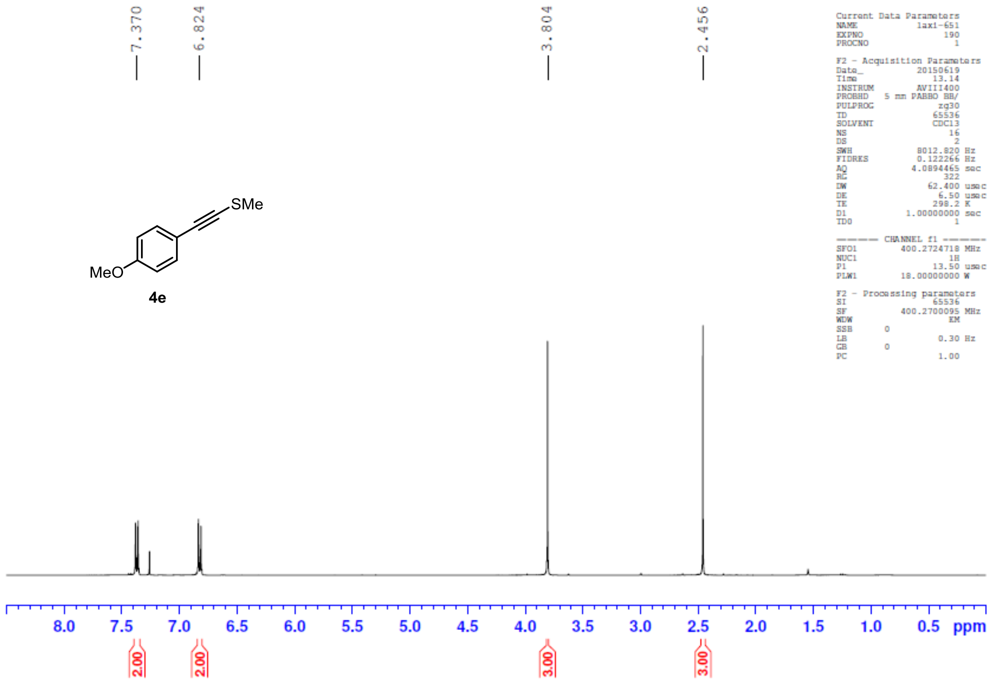

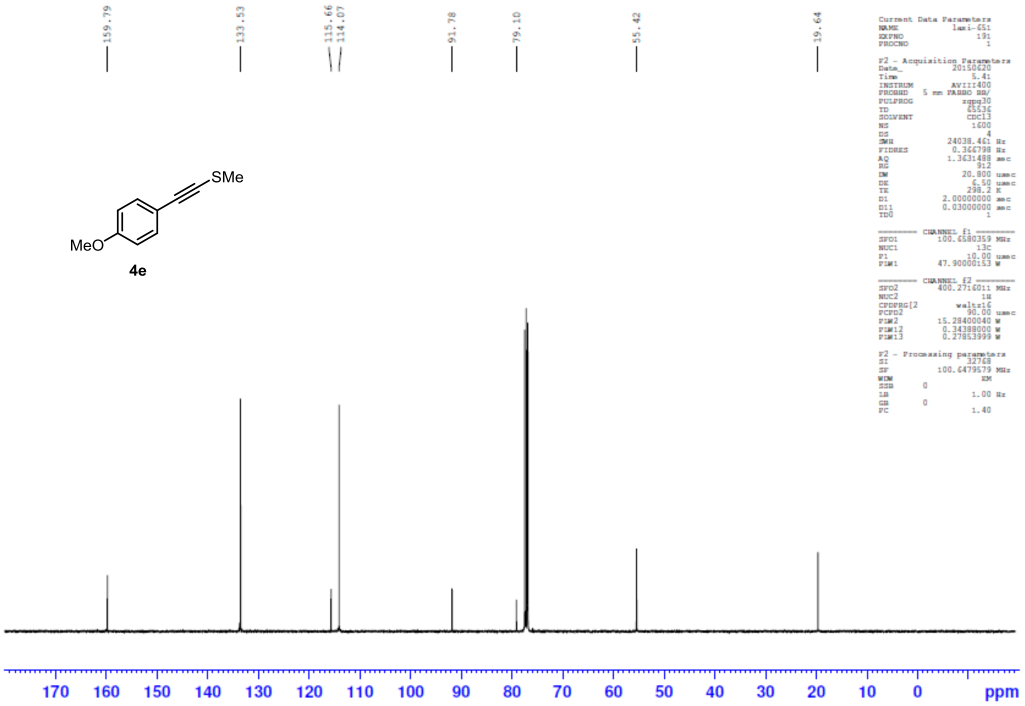

Supplementary Figure 45. ^{1}H NMR and ^{13}C NMR spectra of substrate **4e**

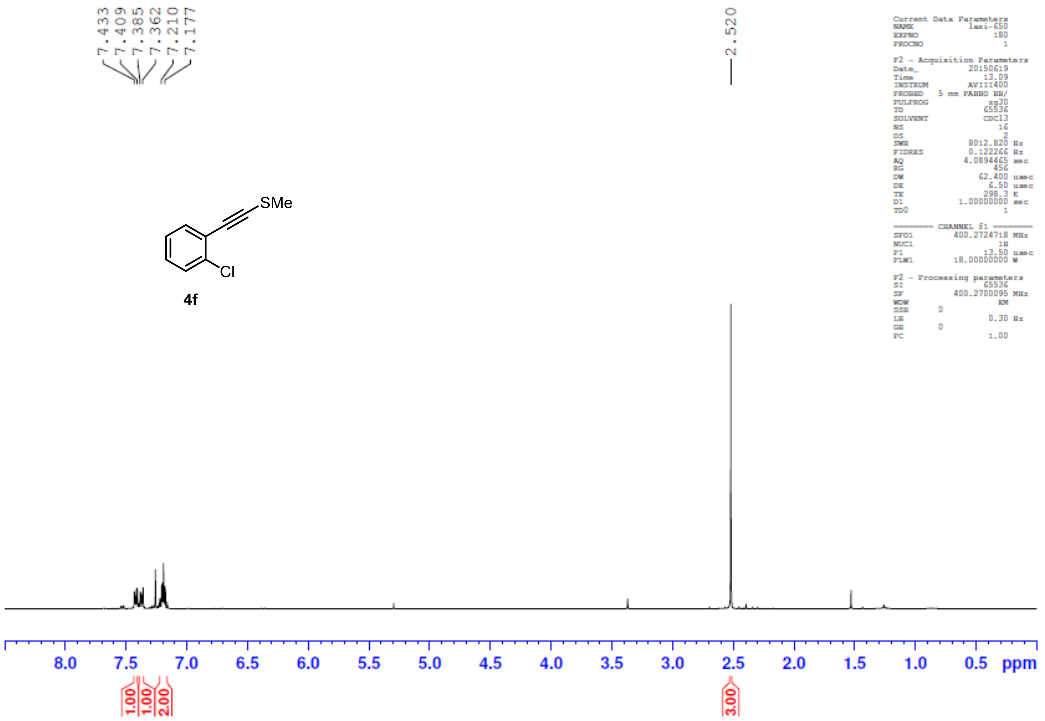

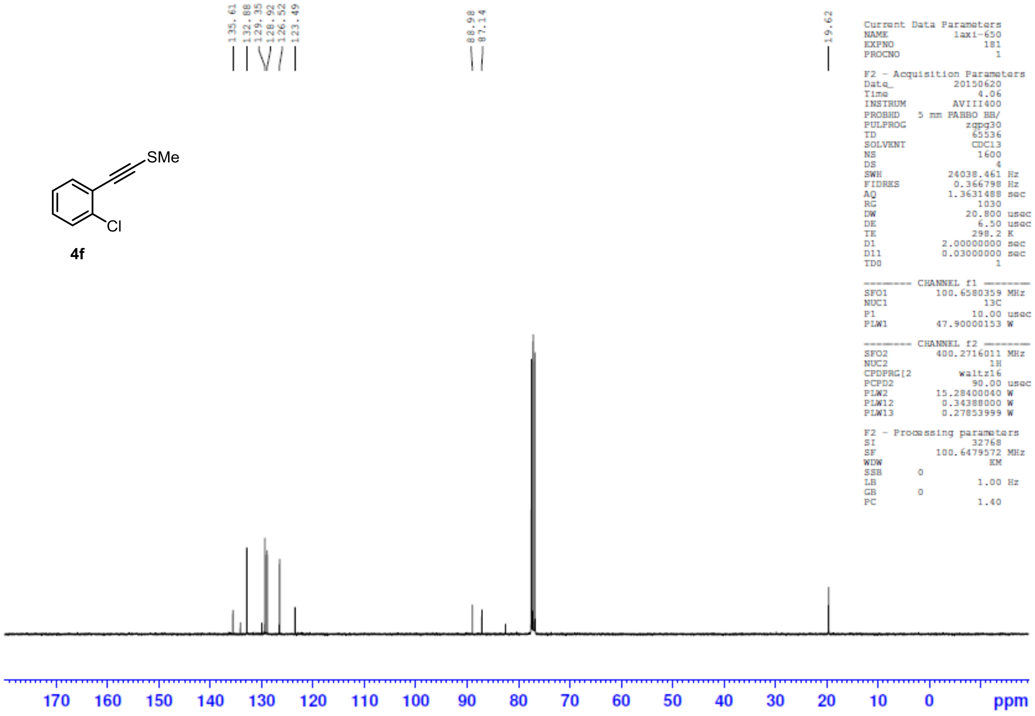

Supplementary Figure 46. ^{1}H NMR and ^{13}C NMR spectra of substrate **4f**

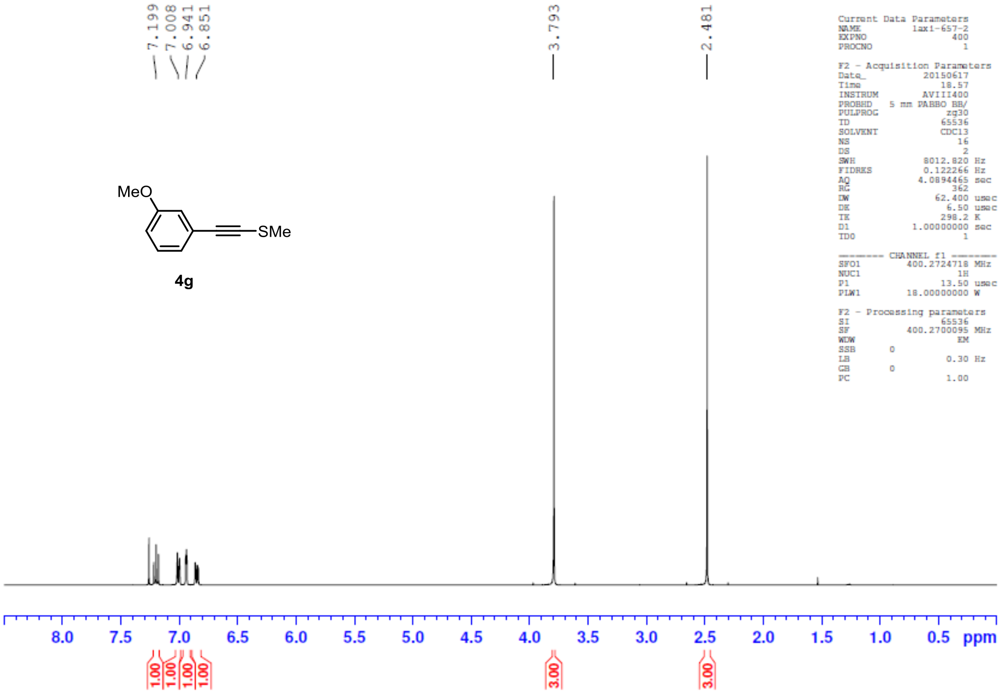

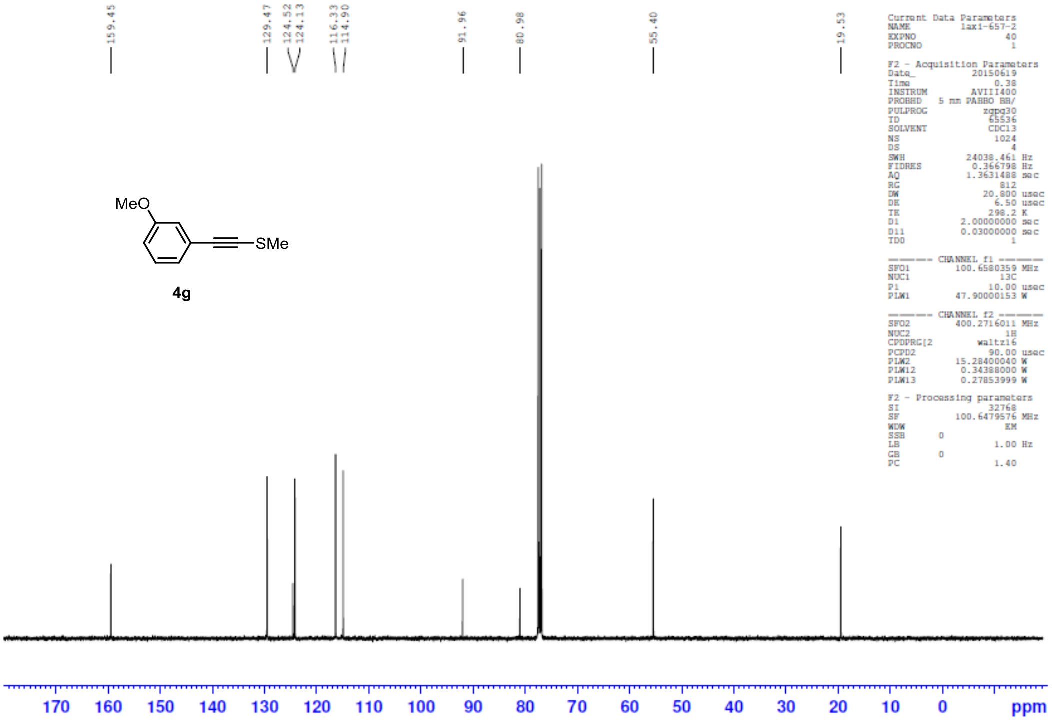

Supplementary Figure 47. ^{1}H NMR and ^{13}C NMR spectra of substrate **4g**

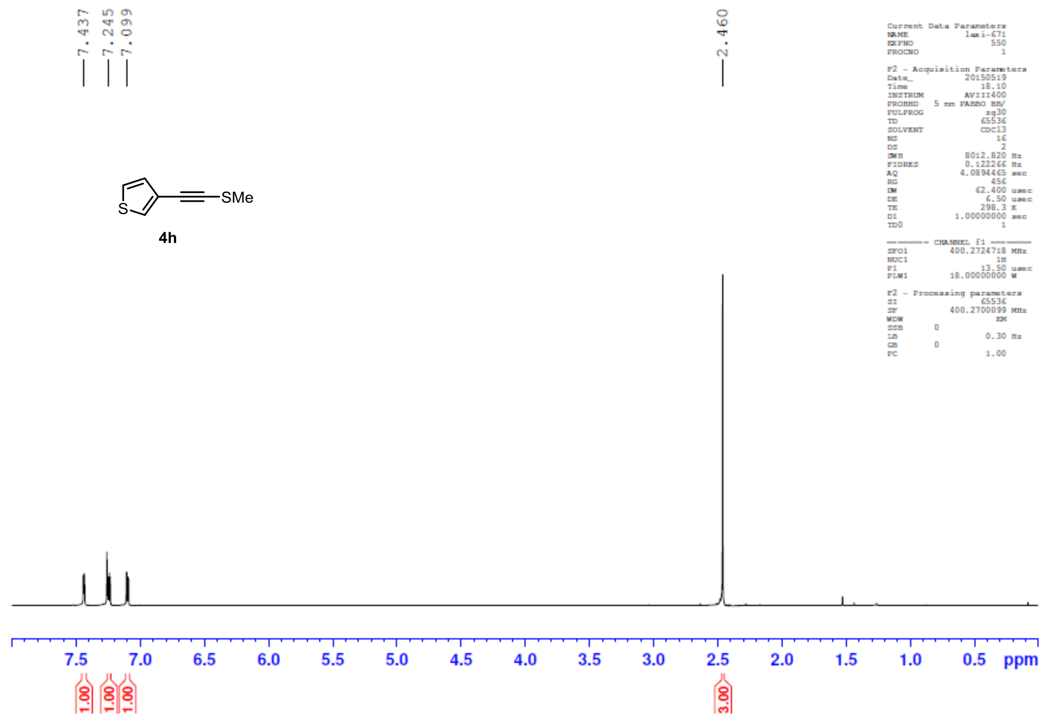

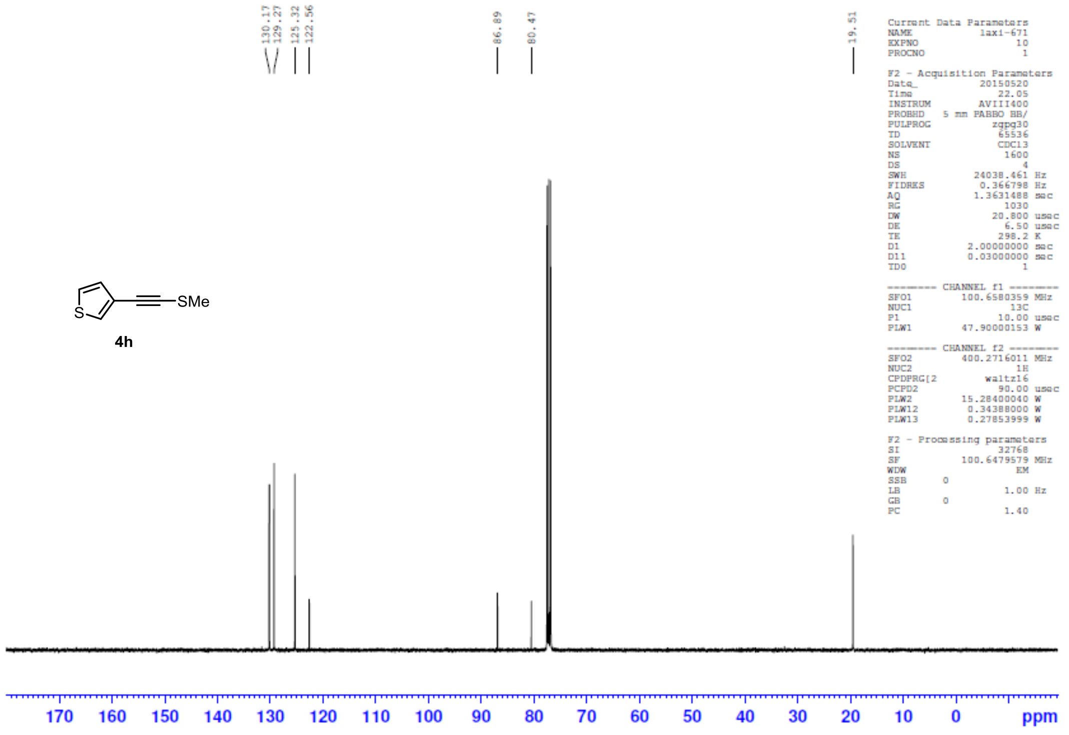

Supplementary Figure 48. ^{1}H NMR and ^{13}C NMR spectra of substrate **4h**

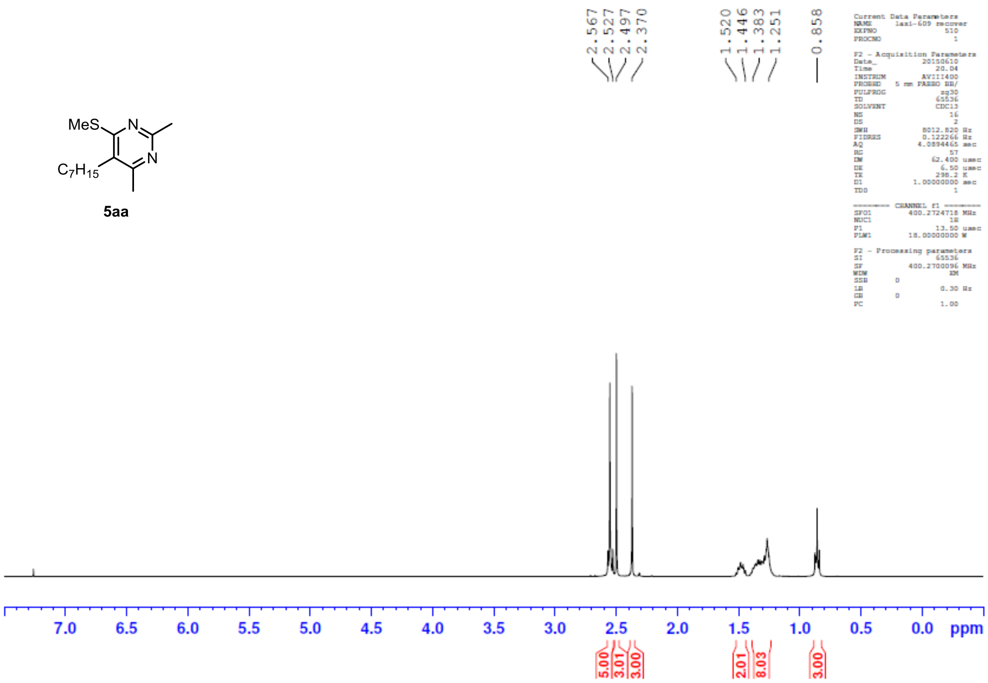

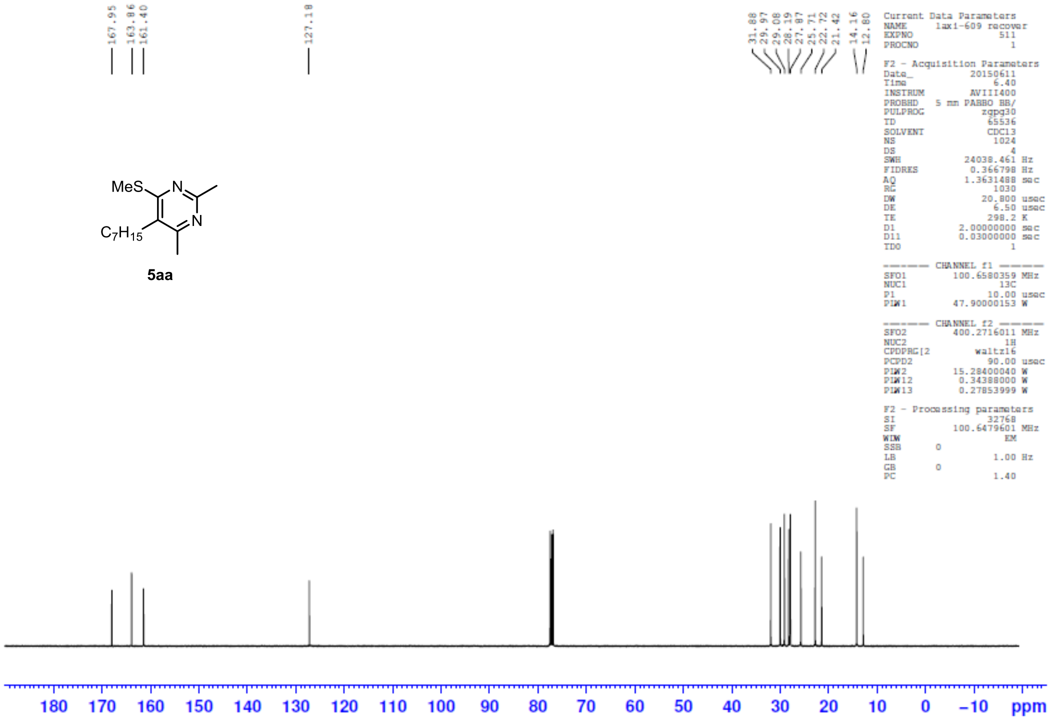

Supplementary Figure 49. ^{1}H NMR and ^{13}C NMR spectra of substrate **5aa**

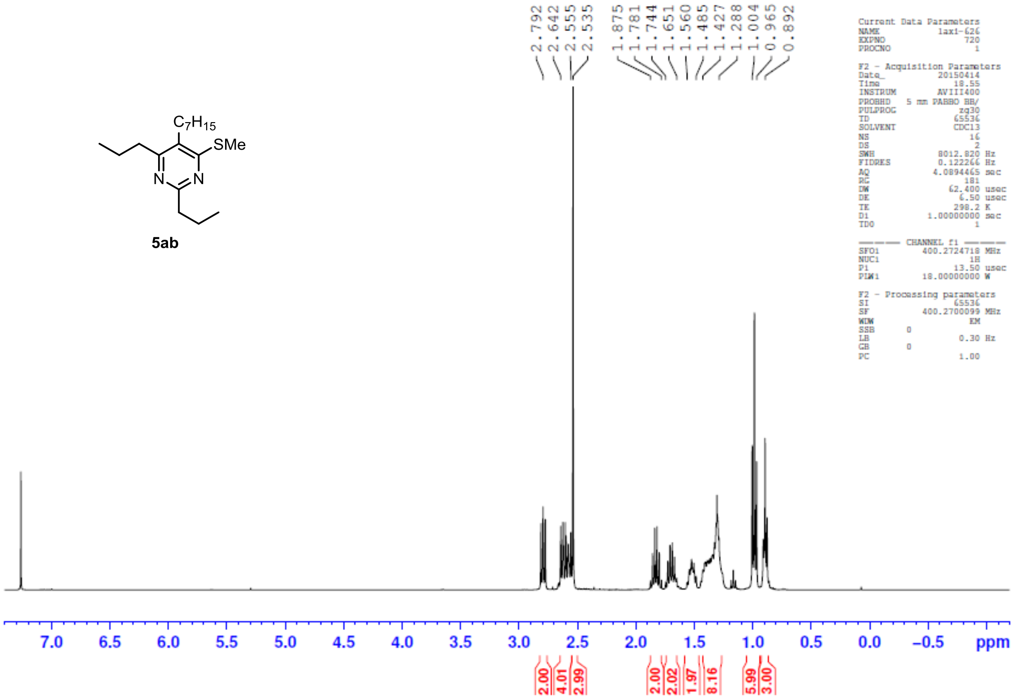

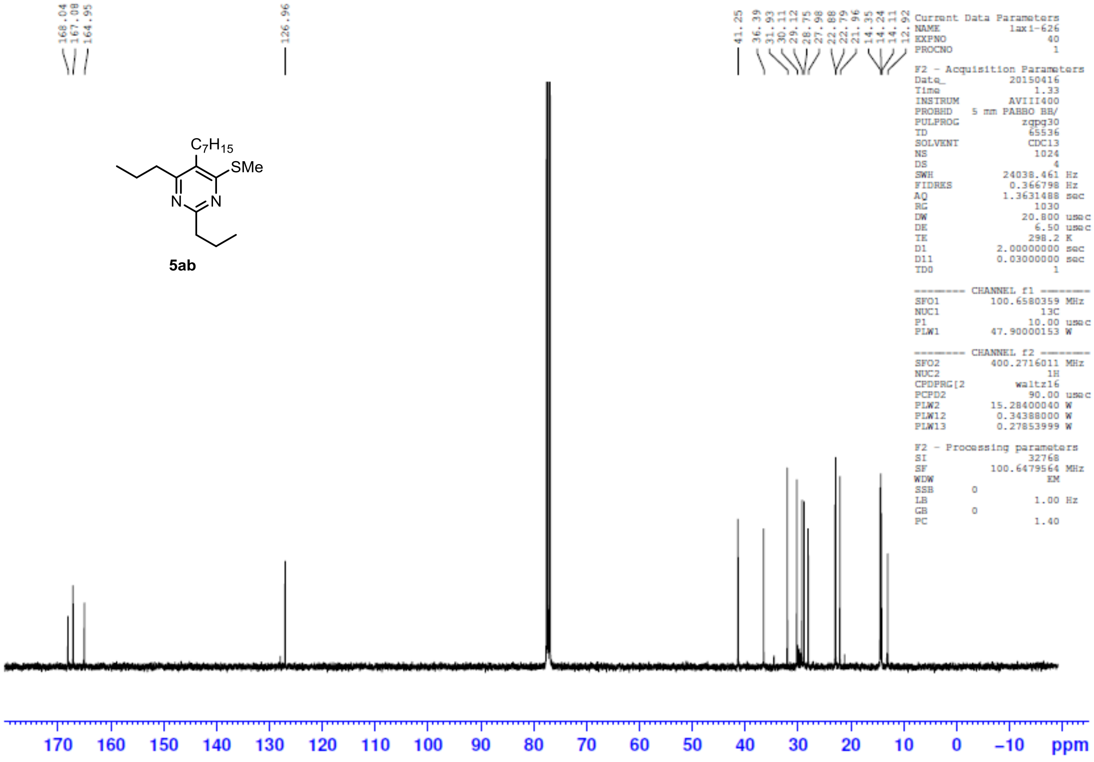

Supplementary Figure 50. ^{1}H NMR and ^{13}C NMR spectra of substrate **5ab**

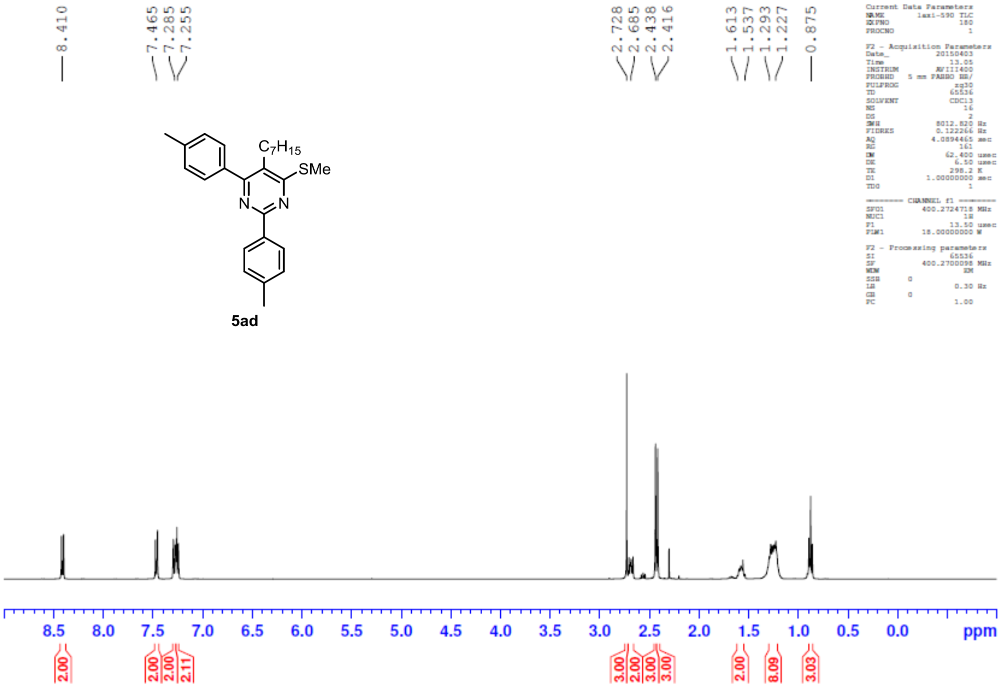

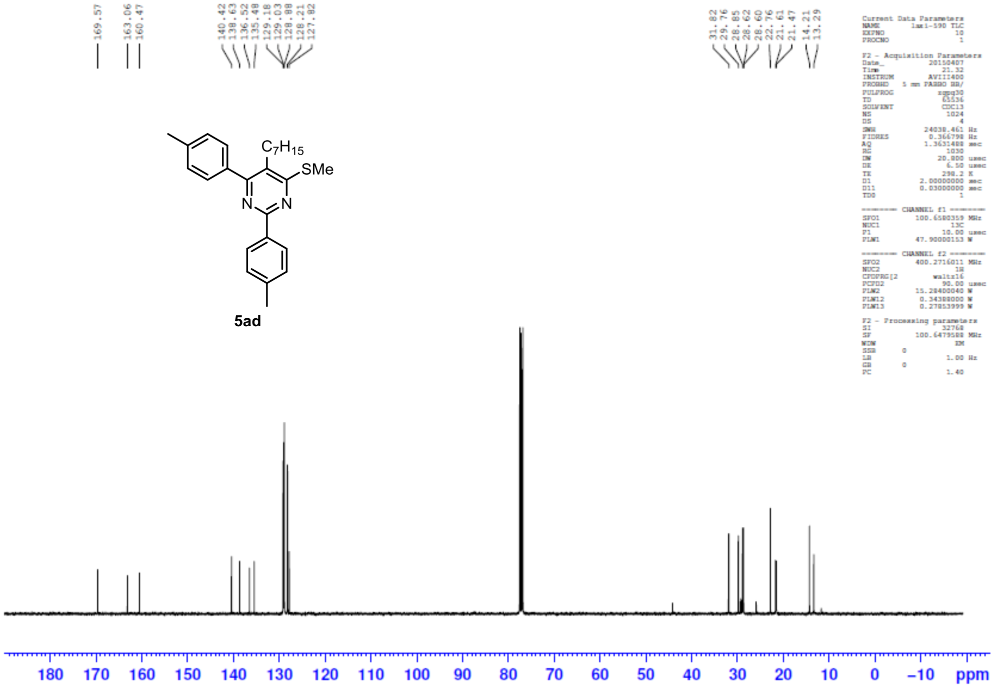

Supplementary Figure 51. ^{1}H NMR and ^{13}C NMR spectra of substrate **5ad**

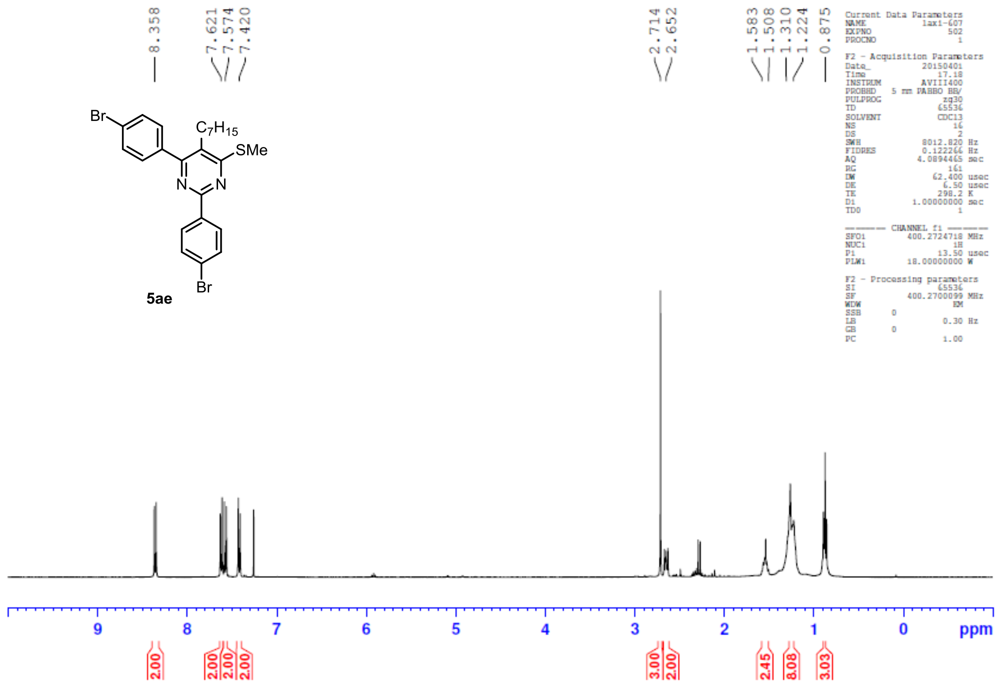

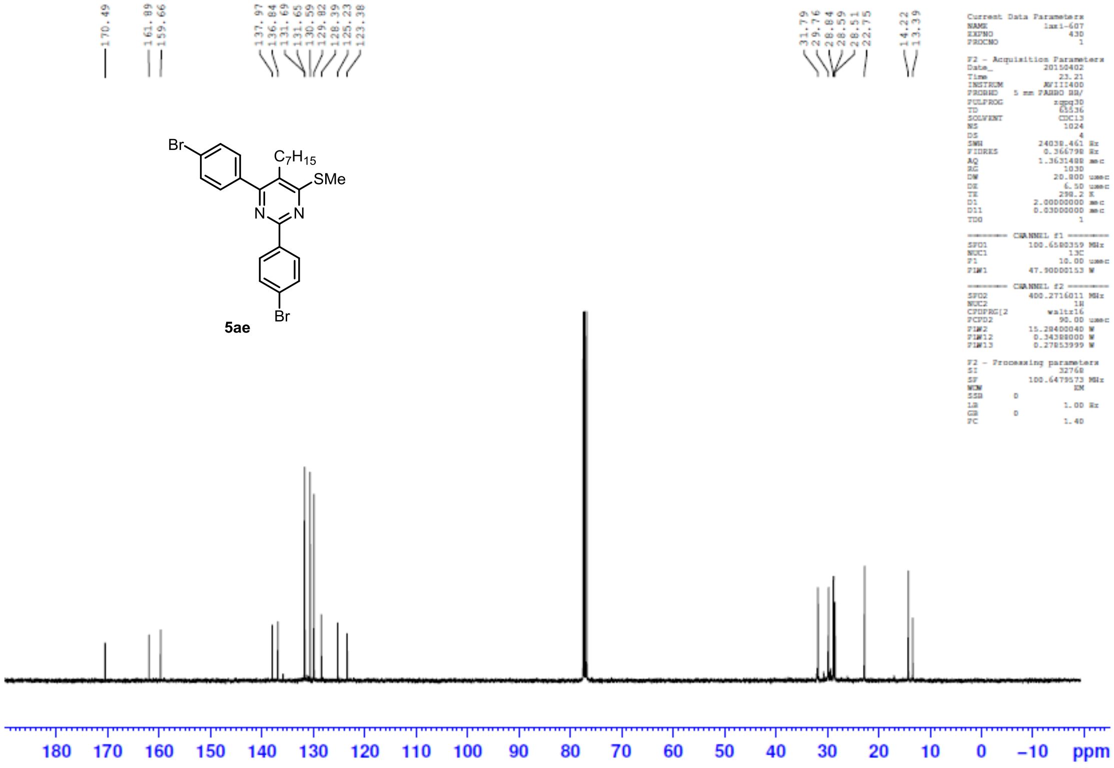

Supplementary Figure 52. ^{1}H NMR and ^{13}C NMR spectra of substrate **5ae**

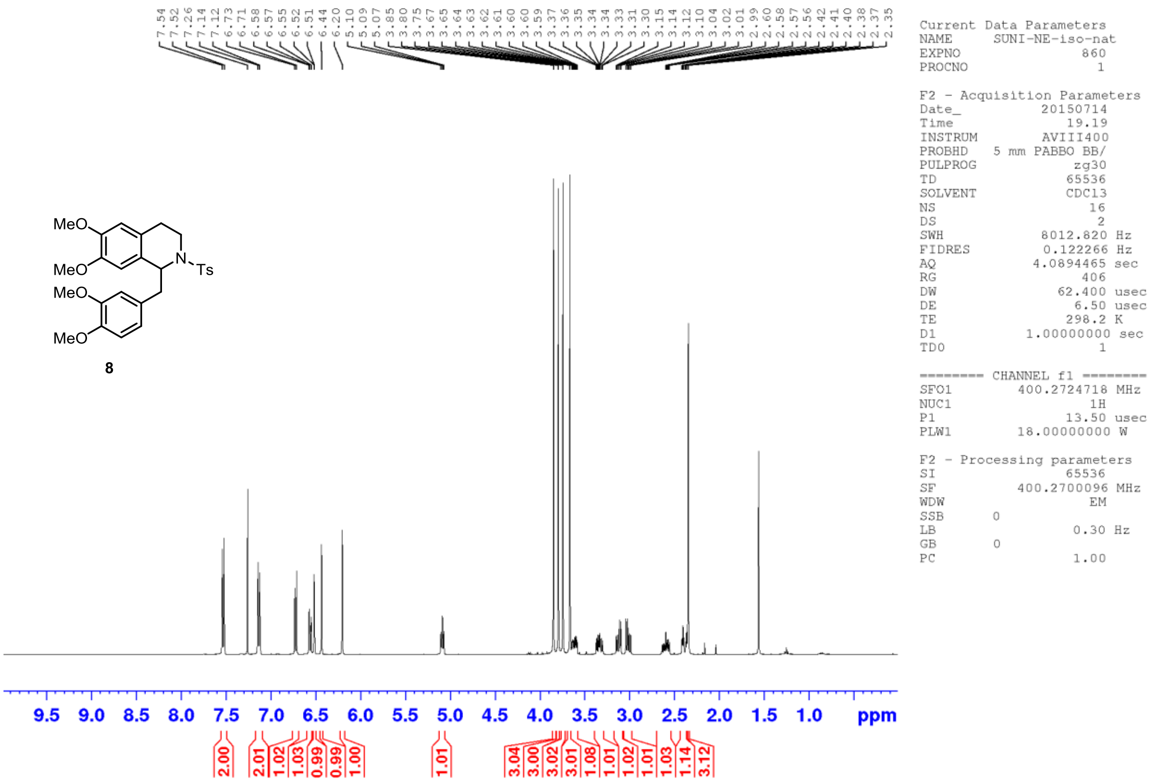

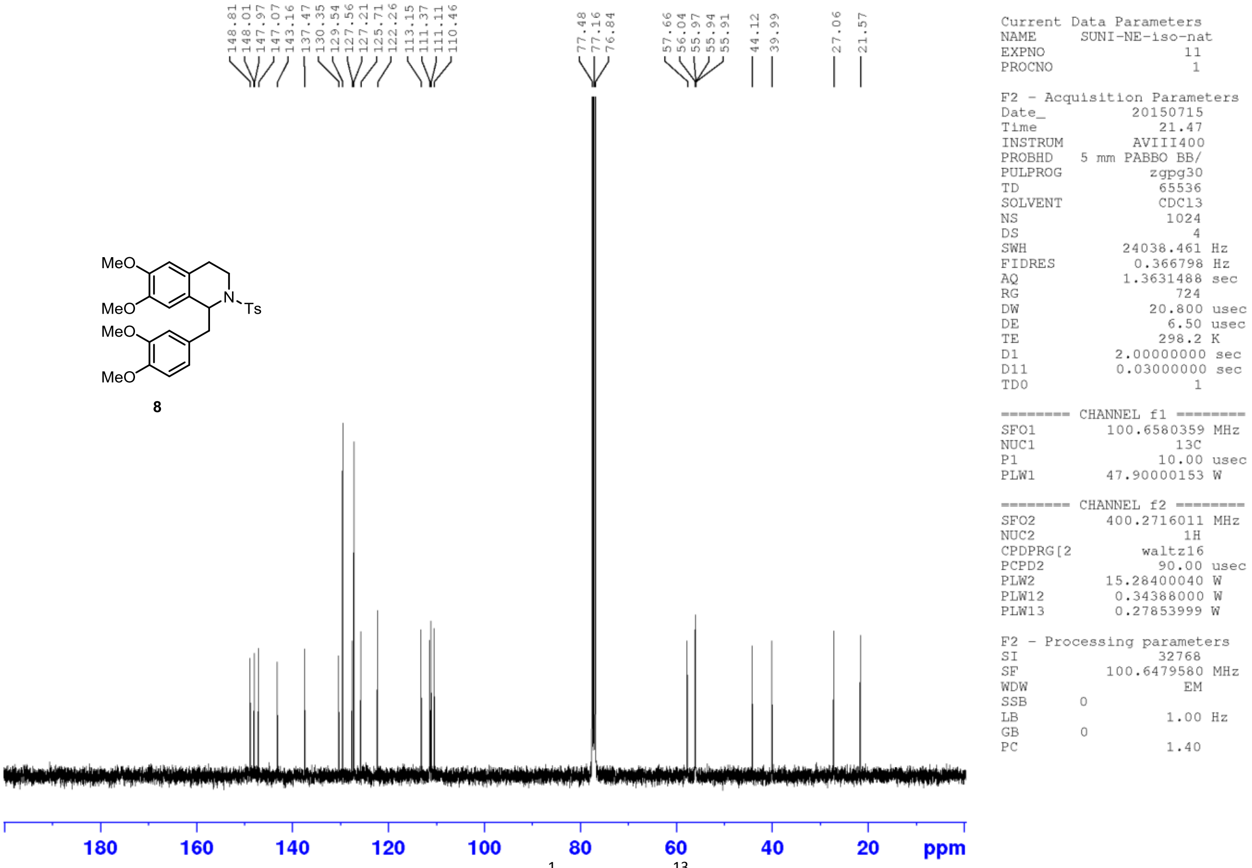

Supplementary Figure 53. ^{1}H NMR and ^{13}C NMR spectra of substrate **8**

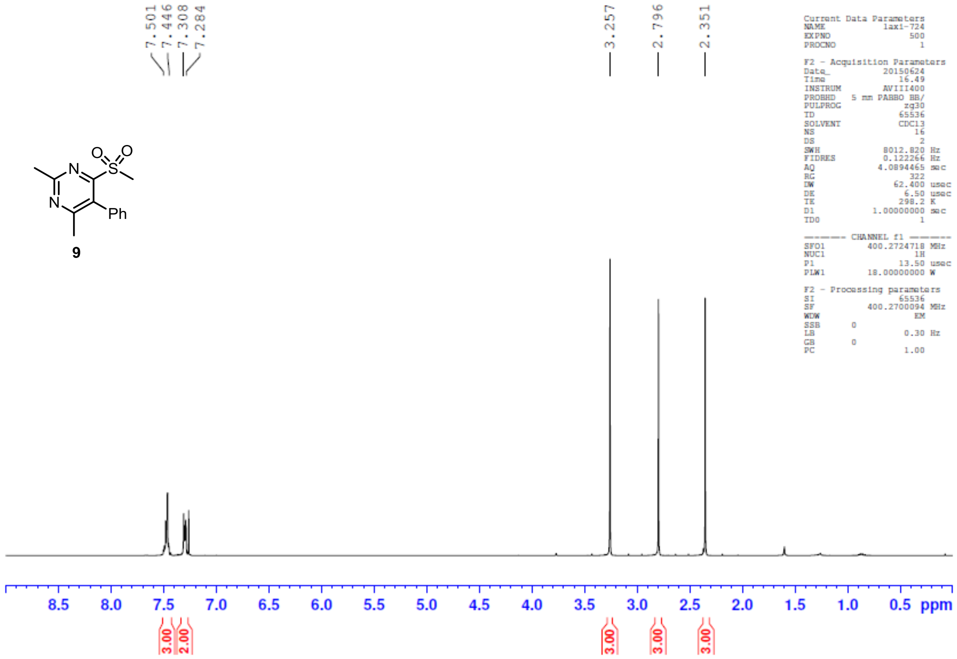

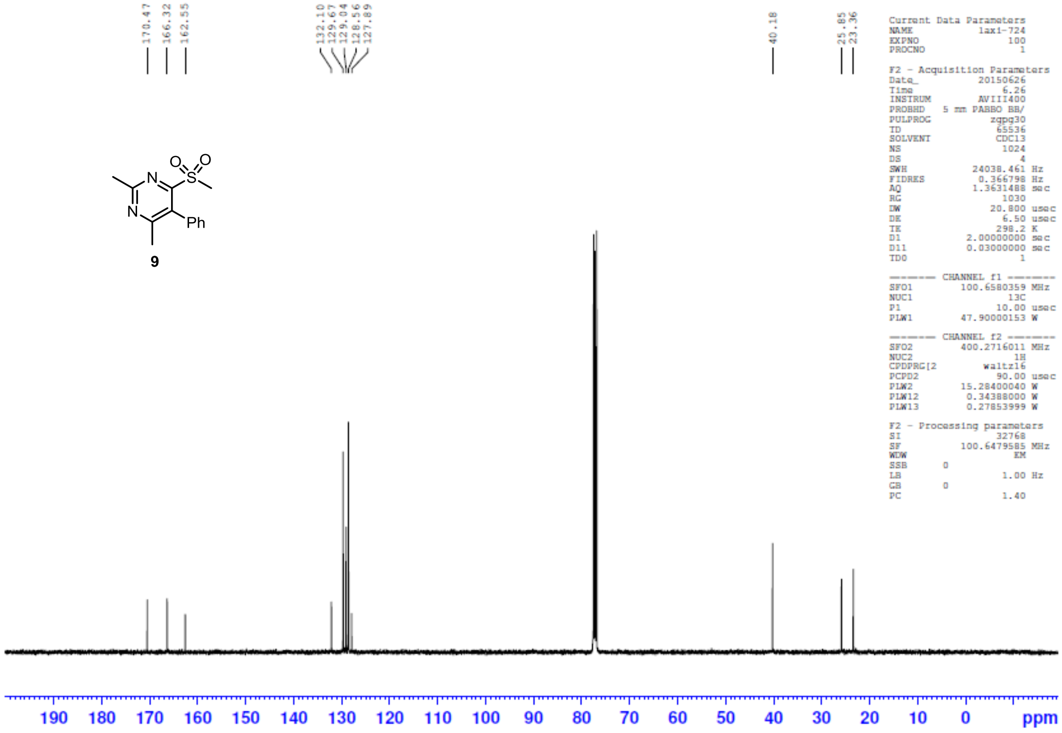

Supplementary Figure 54. ^{1}H NMR and ^{13}C NMR spectra of substrate **9**

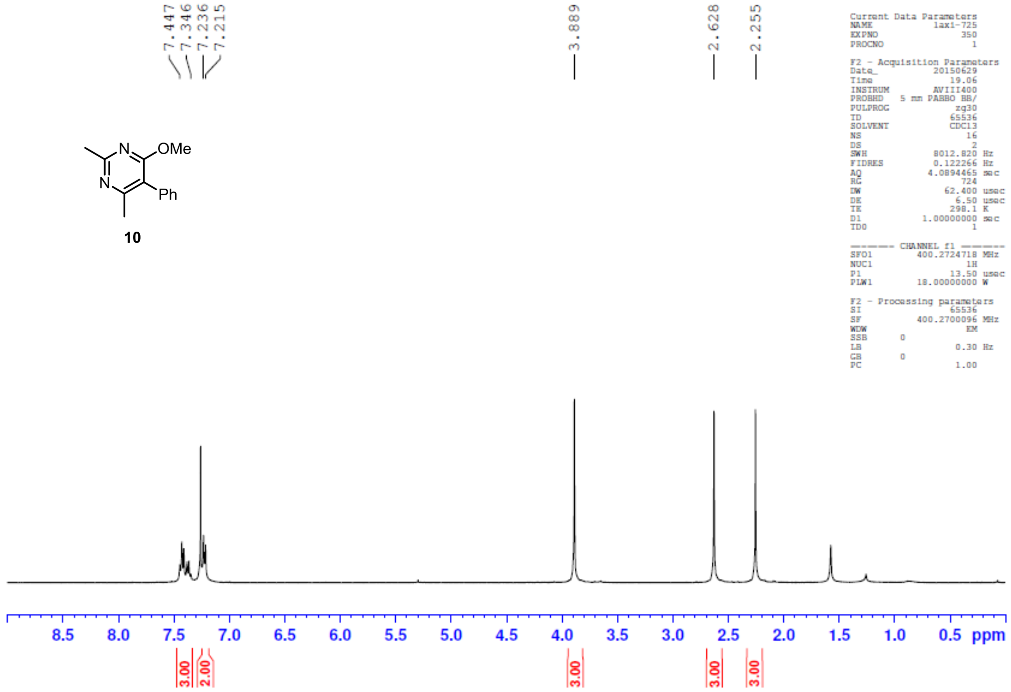

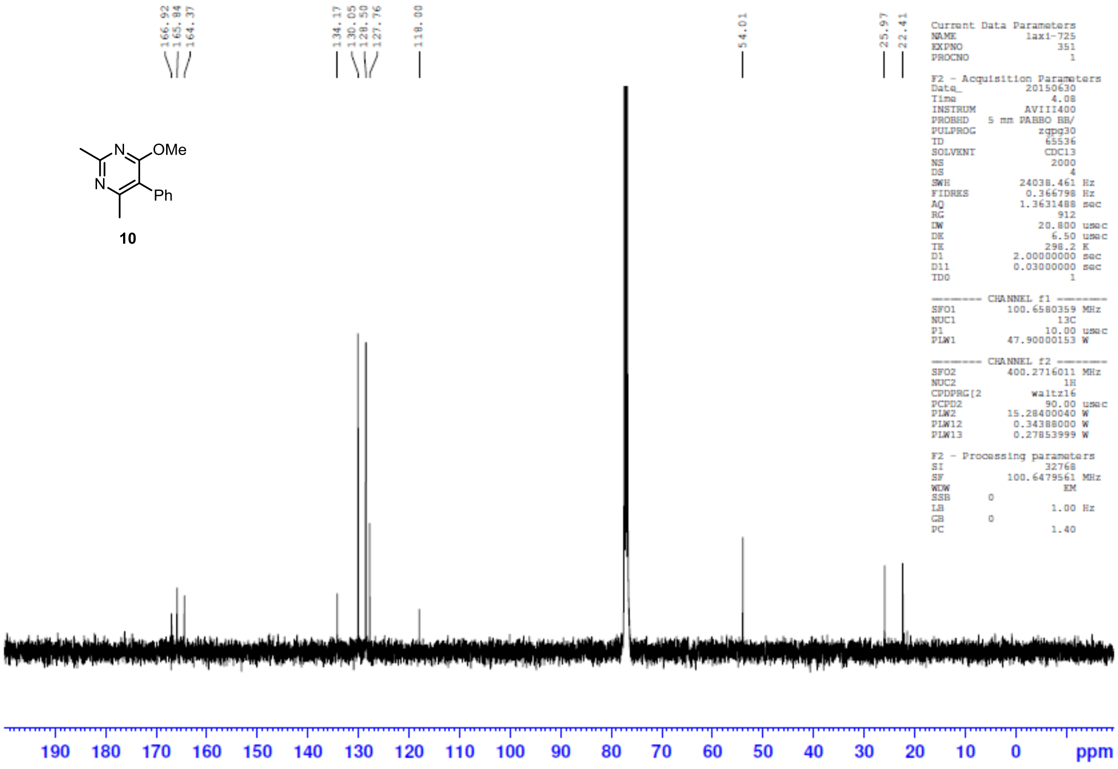

Supplementary Figure 55. ^{1}H NMR and ^{13}C NMR spectra of substrate **10**

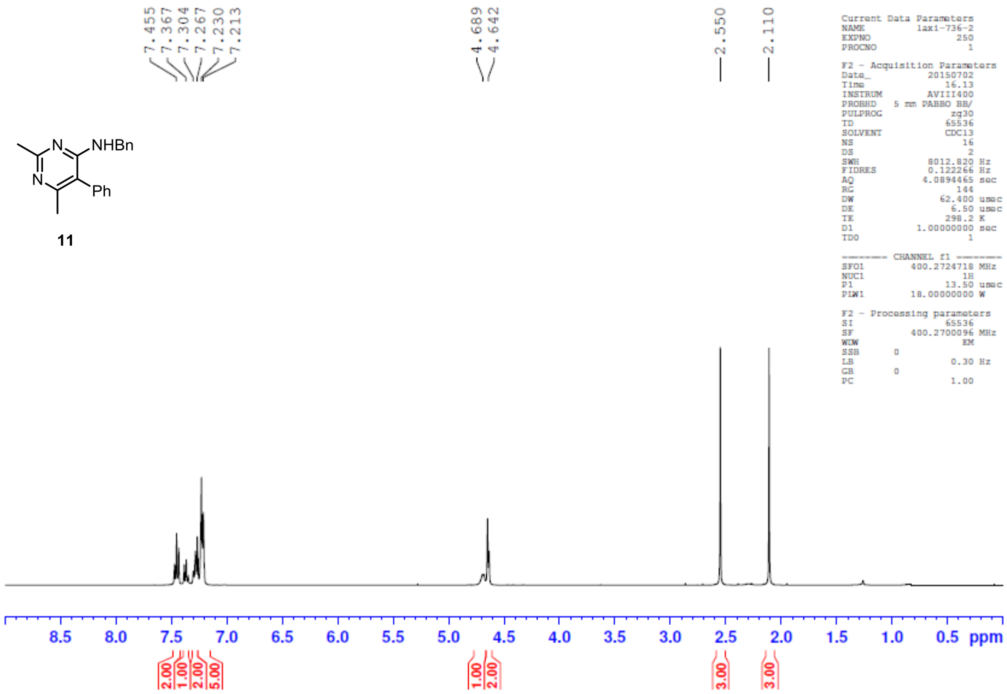

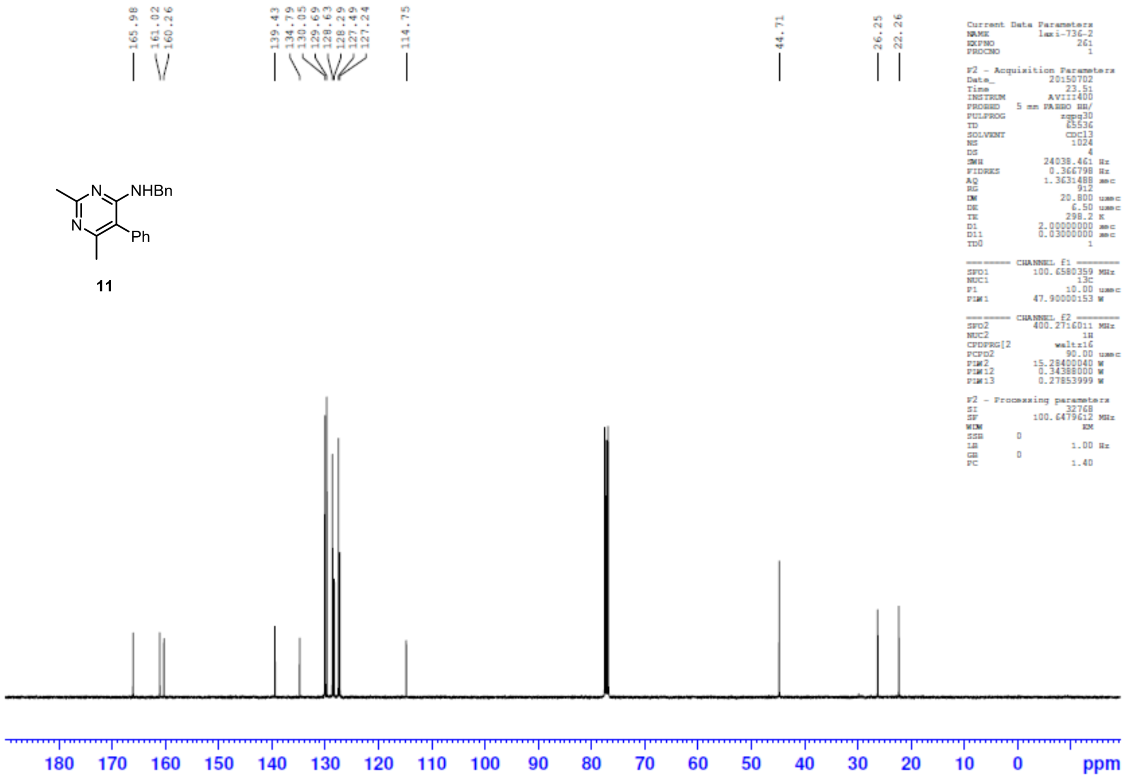

Supplementary Figure 56. ^{1}H NMR and ^{13}C NMR spectra of substrate **11**

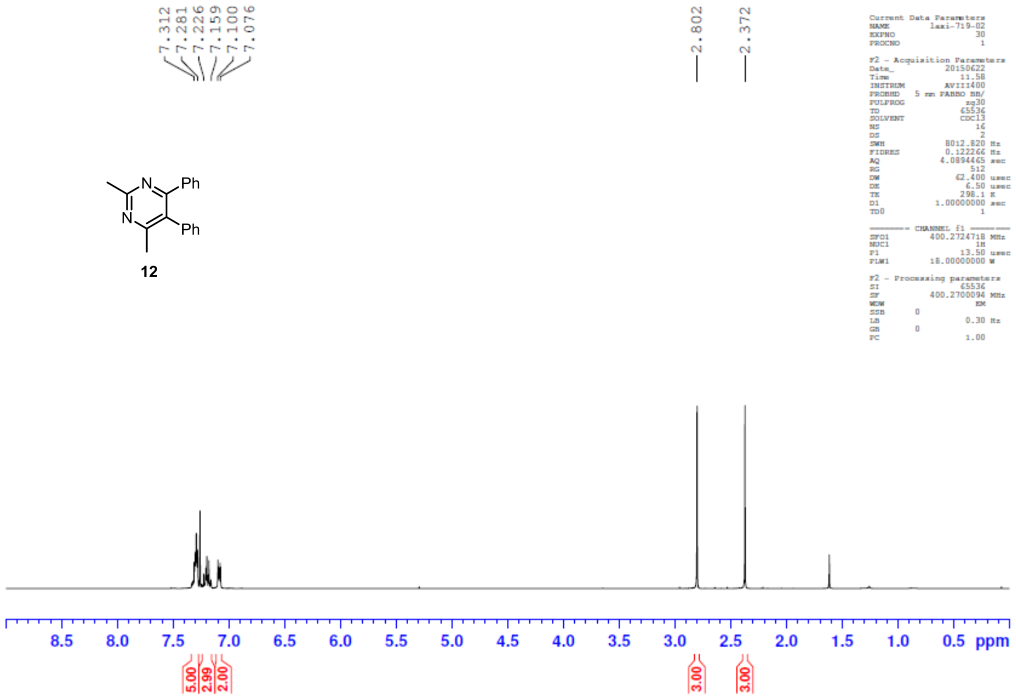

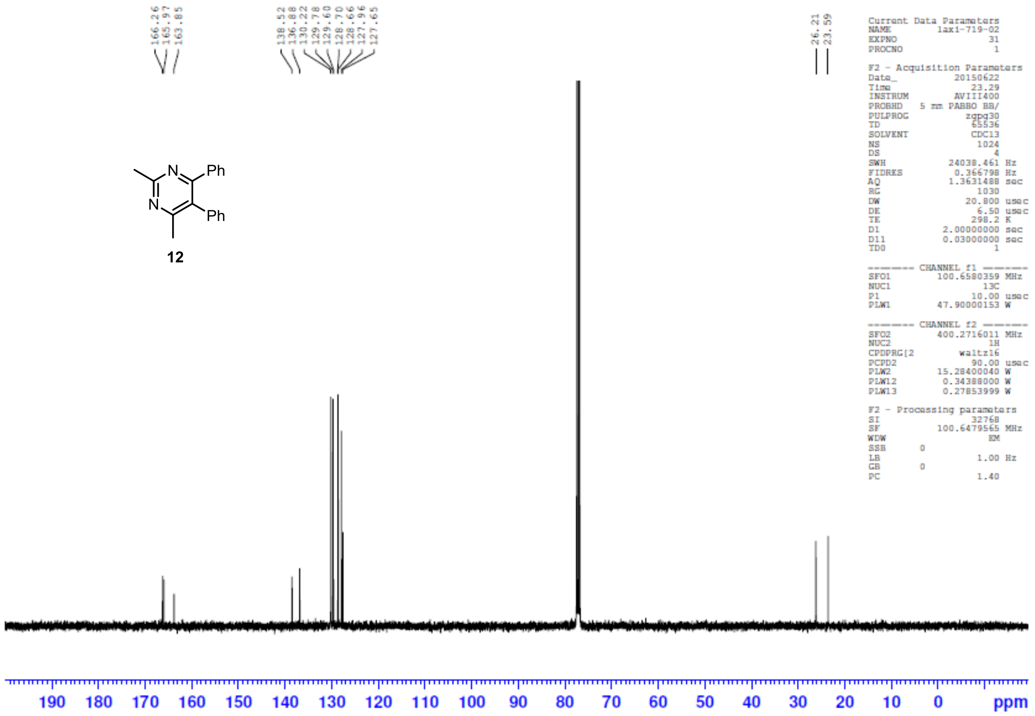

Supplementary Figure 57. ^{1}H NMR and ^{13}C NMR spectra of substrate **12**

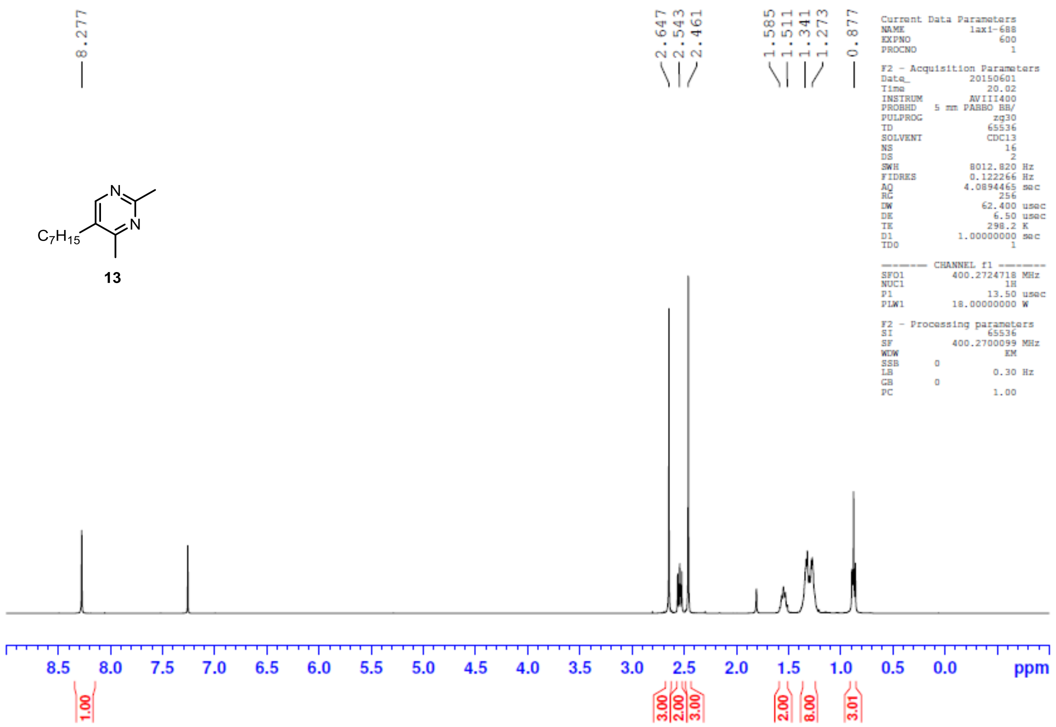

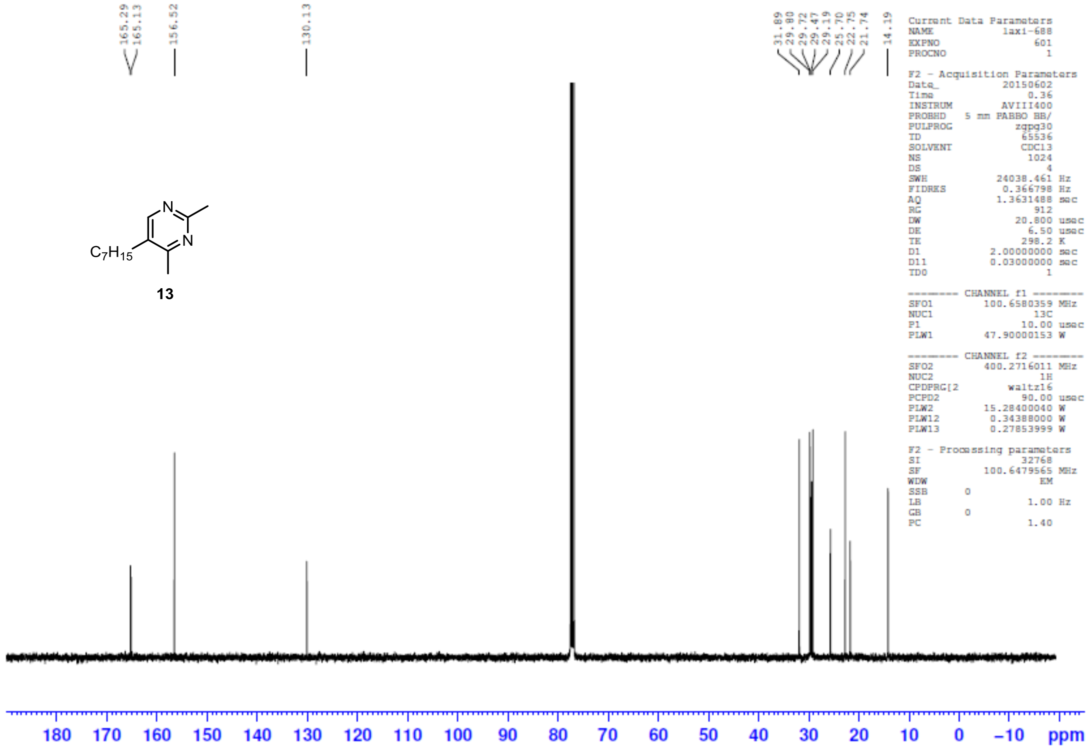

Supplementary Figure 58. ^{1}H NMR and ^{13}C NMR spectra of substrate **13**

Report

Sample Information

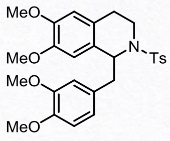

8

Sample Name	: Laxi-536(Rac) ap
Sample ID	:
Vial#	: 1
Injection Volume	: 8
Data File	: Laxi-536(Rac) ap_19.02.2015_1_005.lcd
Method File	: Run_Heptan70%_EtOH30%F1.lcm
Batch File	: 19.02.2015_1.lcb
Report Format File	: REPORTLux Hep7_EtOH3F1.lsr
Date Acquired	: 19.02.2015 13:12:55
Date Processed	: 19.02.2015 13:37:52

Method Description:
Column:Lux-3 Cellulose-3
150x4,6mm Particle Size 3 micrometer
Solvent System:(n-Heptan+0,1%IPA)/EtOH 7:3
Flow=1 ml/min T=25°C

Chromatogram

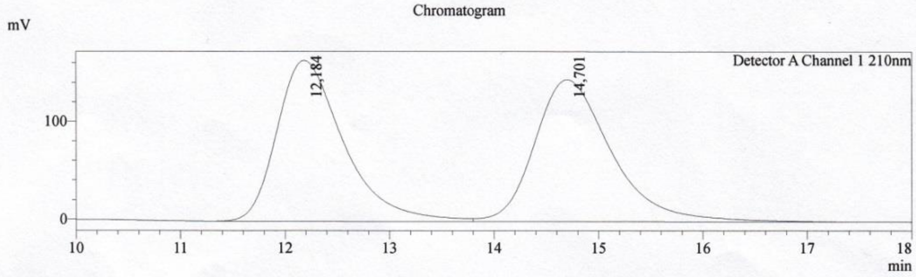

Peak Table

Detector A Channel 1 210nm

Peak#	Ret. Time	Area	Area%
1	12,184	7309808	49,665
2	14,701	7408387	50,335
Total		14718196	100,000

Supplementary Figure 59. HPLC spectra for racemic product **8**

Report

Sample Information

Sample Name : Laxi-537
Sample ID :
Vial# : 3
Injection Volume : 8
Data File : Laxi-537_19.02.2015_1_001.lcd
Method File : Run_Heptan70%_EtOH30%F1.lcm
Batch File : 19.02.2015_1.lcb
Report Format File : REPORTLux Hep7_EtOH3F1.lsr
Date Acquired : 19.02.2015 13:32:57
Date Processed : 19.02.2015 13:56:21

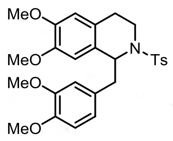

8

Method Description:
Column:Lux-3 Cellulose-3
150x4,6mm Particle Size 3 micrometer
Solvent System:(n-Heptan+0,1%IPA)/EtOH 7:3
Flow=1 ml/min T=25°C

Chromatogram

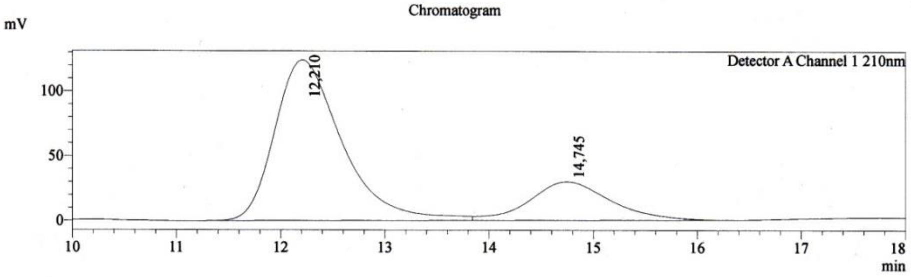

Peak Table

Detector A Channel 1 210nm

Peak#	Ret. Time	Area	Area%
1	12,210	5566492	77,594
2	14,745	1607333	22,406
Total		7173825	100,000

Supplementary Figure 60. HPLC spectra for chiral product **8**

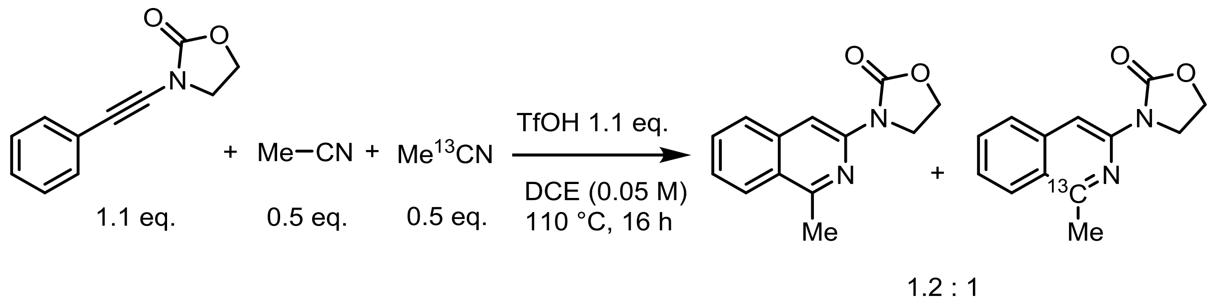

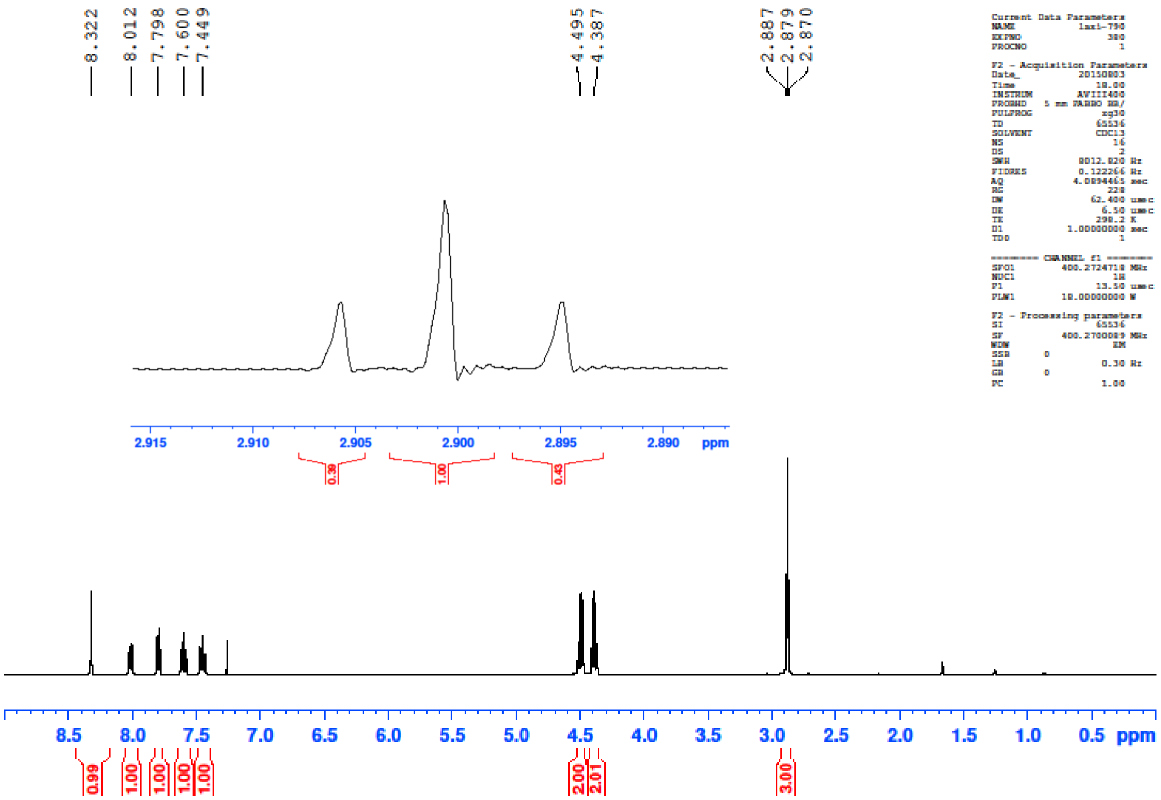

Supplementary Figure 61. Kinetic isotope effect.

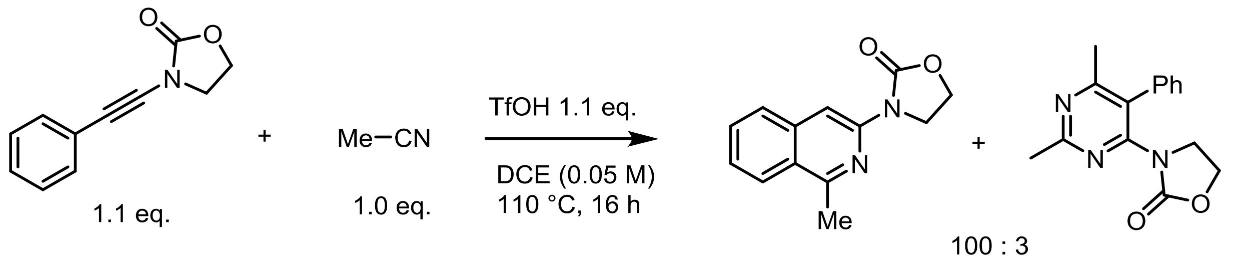

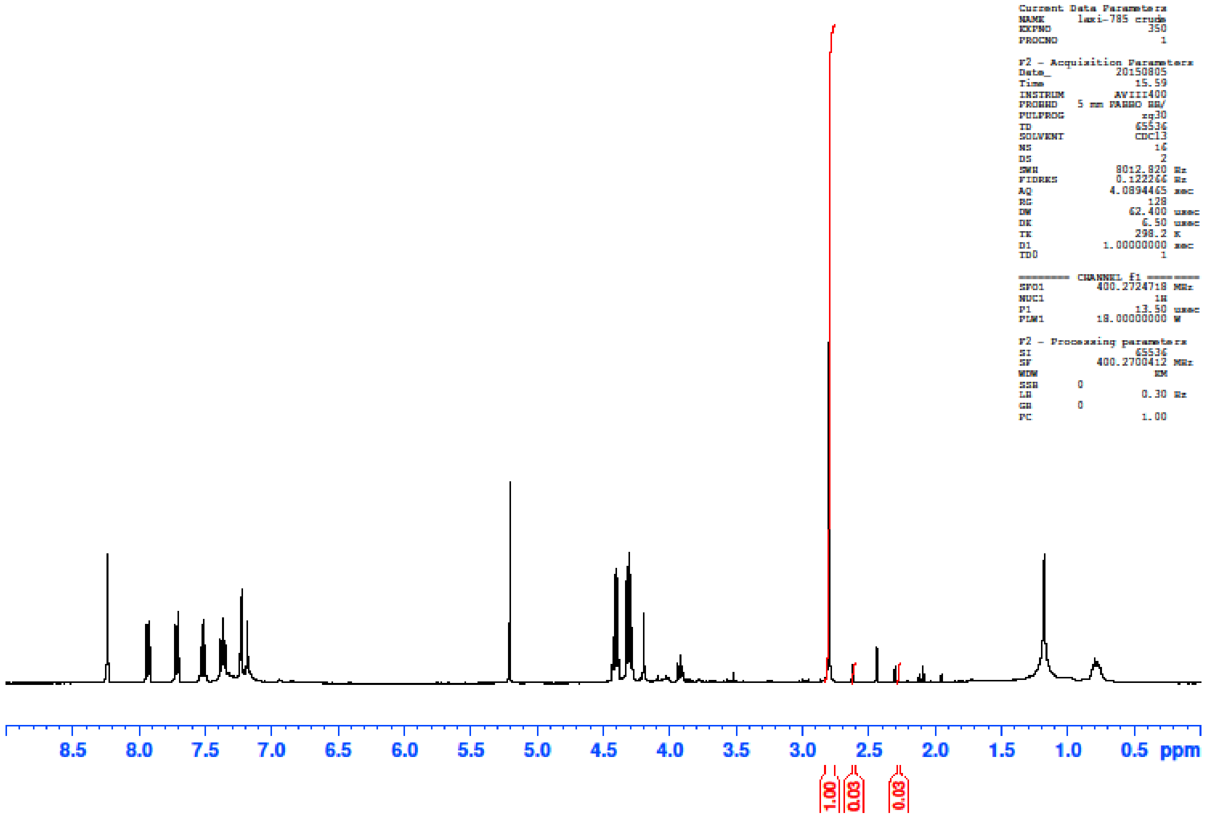

Supplementary Figure 62. Orthogonality in the synthesis of heterocycles (using 1.0 eq. of MeCN).

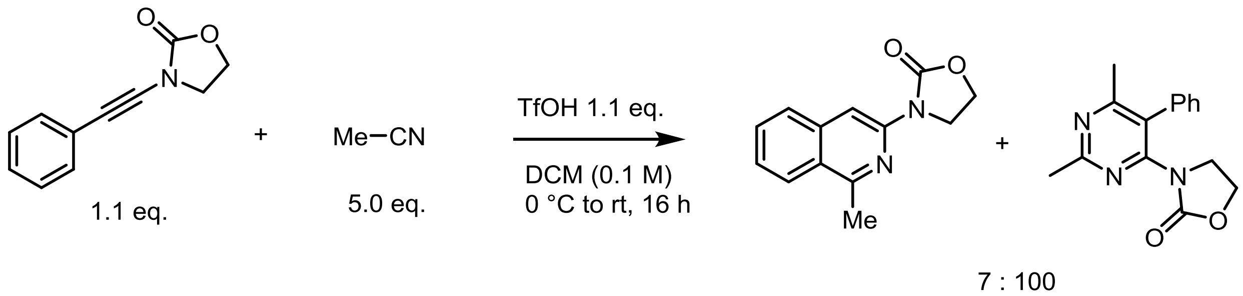

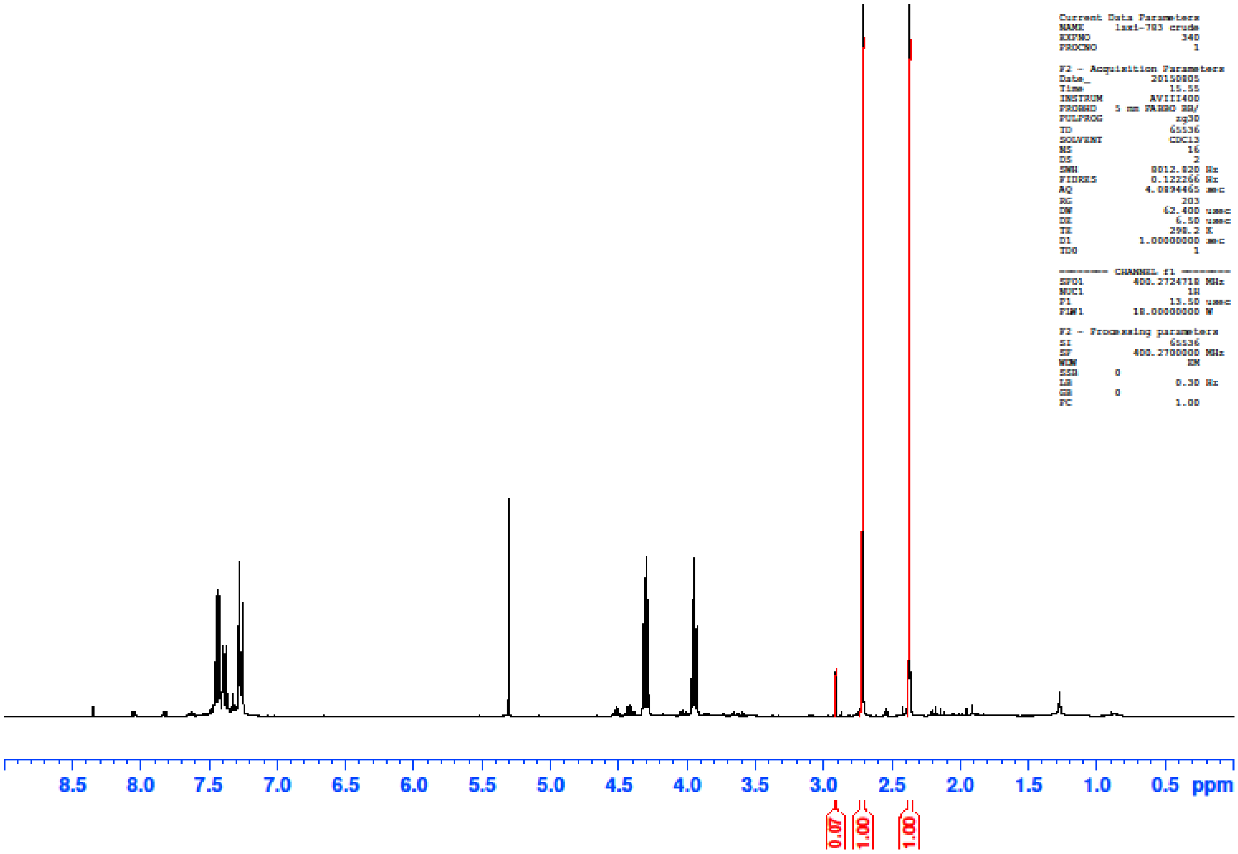

Supplementary Figure 63. Orthogonality in the synthesis of heterocycles (using 5.0 eq. of MeCN).

2,4-bis(4-bromophenyl)-5-heptyl-6-(methylthio)pyrimidine.

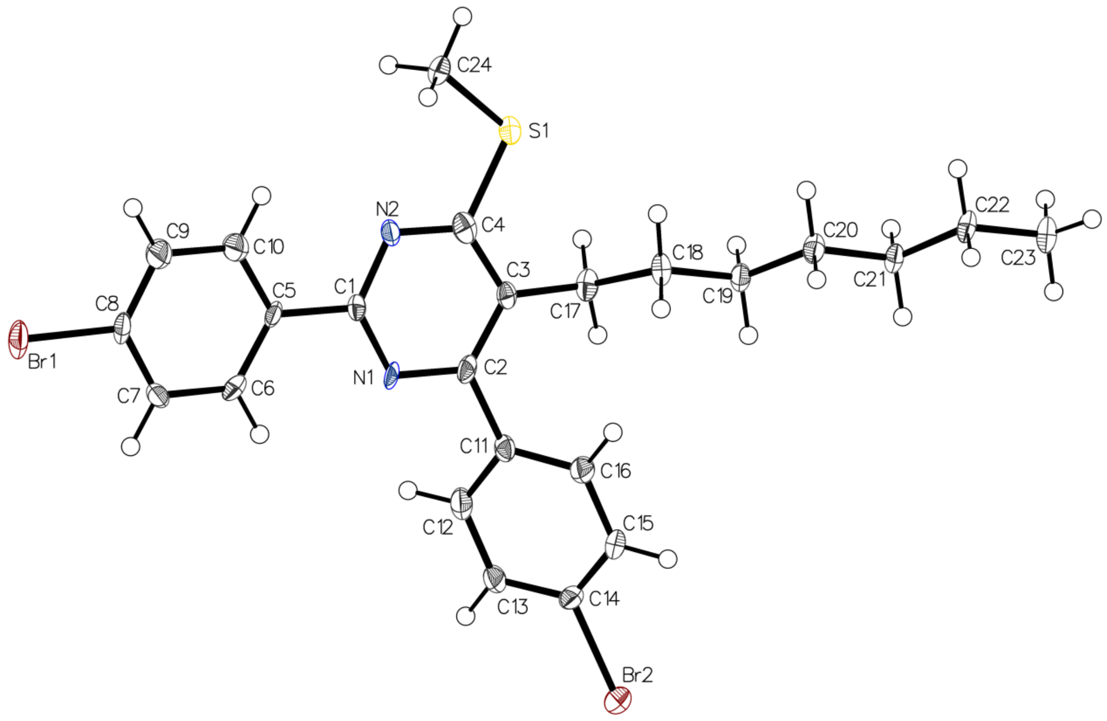

Supplementary Figure 64. Asymmetric Unit of [**laxi607**], drawn with 50% displacement ellipsoids.

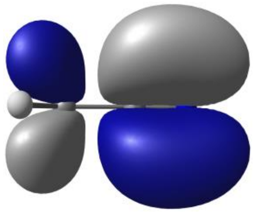

Acetonitrile HOMO

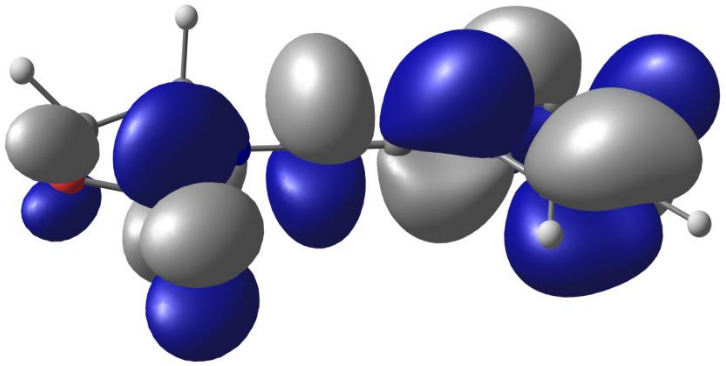

Oxazolidinone derivative LUMO

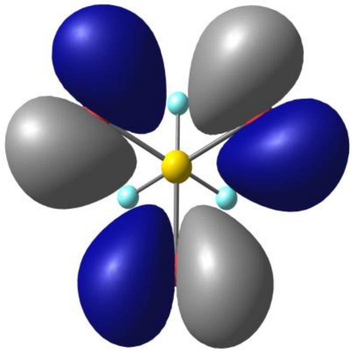

TfO^{-} HOMO

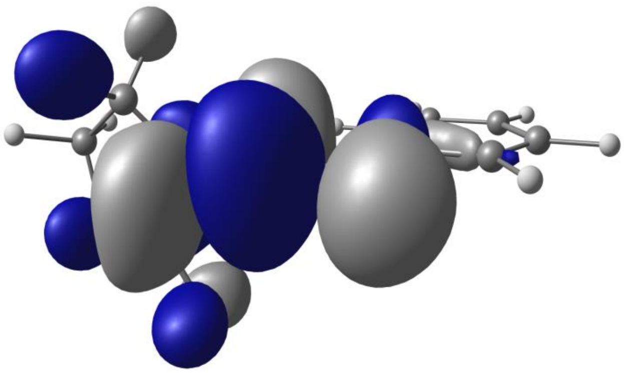

Protonated oxazolidinone derivative LUMO

Supplementary Figure 65. HOMO and LUMO orbital shapes for acetonitrile, TfO^{-} anion, and neutral and protonated oxazolidinone derivatives.

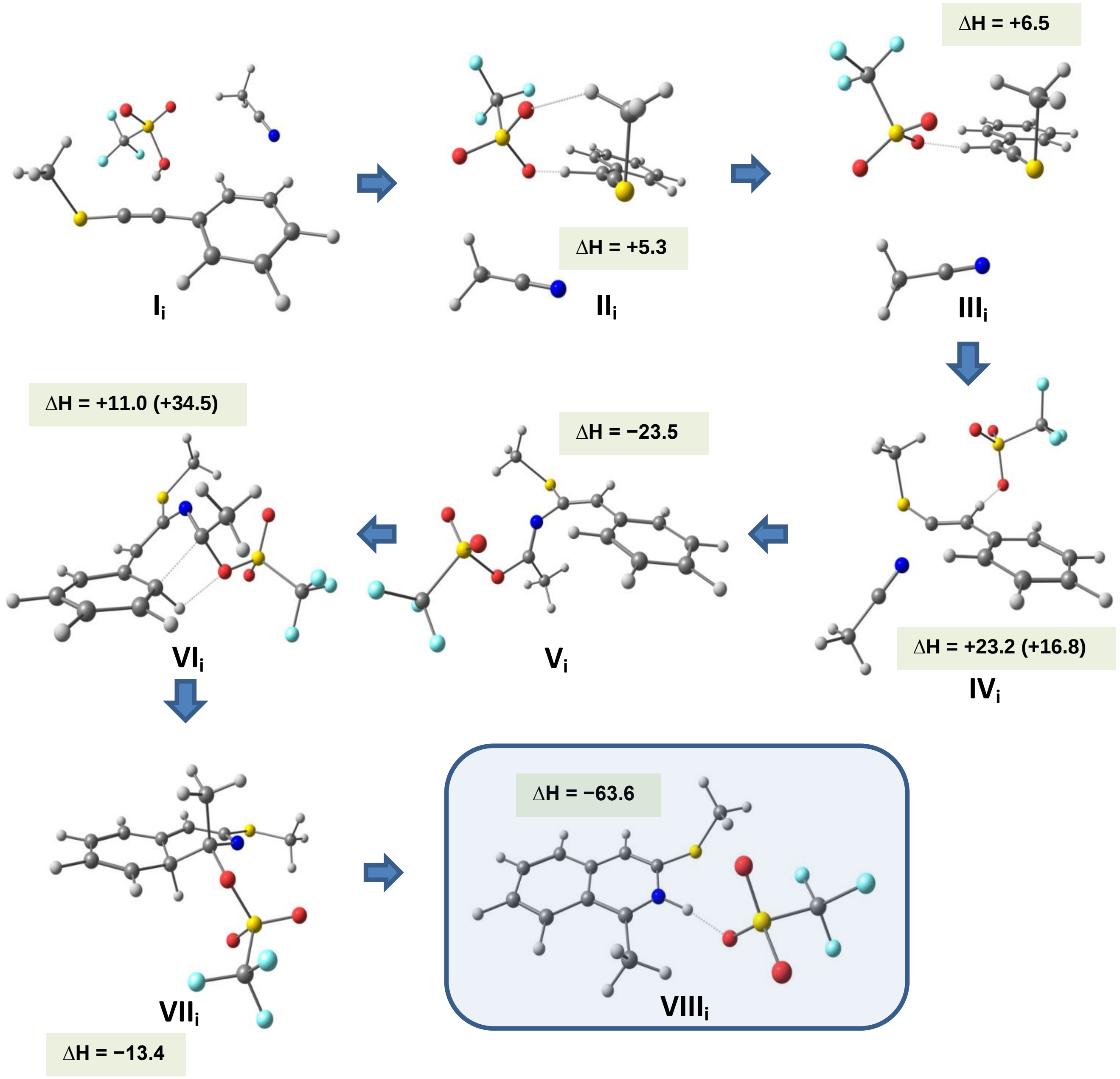

Supplementary Figure 66. Proposed mechanism for the isoquinoline scaffold formation in the case of the methylthio derivative. Values, in Kcal mol^{-1}, are always referred to the starting materials (preassociation complex). In parenthesis, for transition states, the relative values with respect to the intermediate from which they are formed.

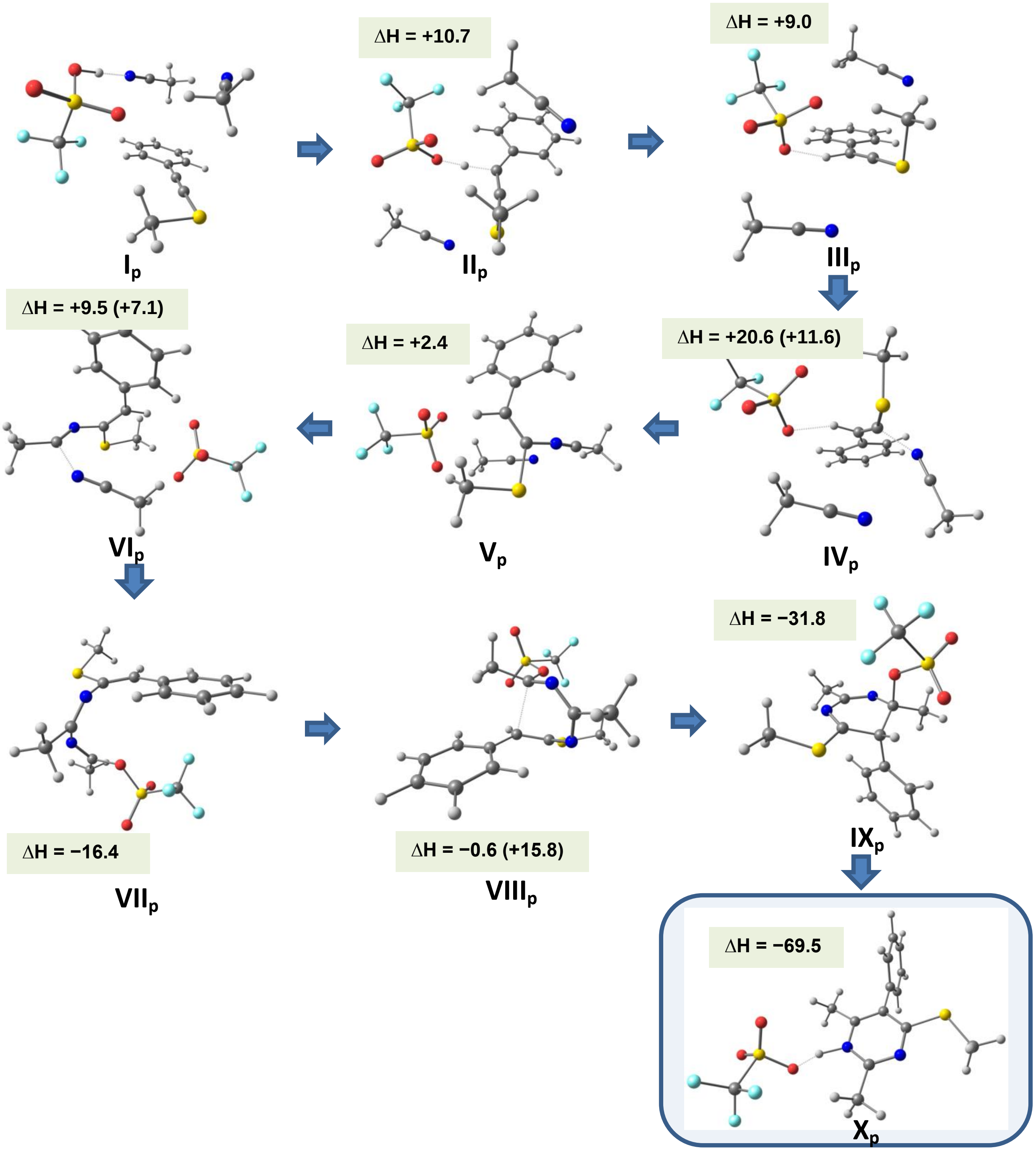

Supplementary Figure 67. Proposed mechanism for the pyrimidine scaffold formation in the case of the methylthio derivative. Values, in Kcal mol^{-1}, are always referred to the starting materials (preassociation complex). In parenthesis, for transition states, the relative values with respect to the intermediate from which they are formed.

Supplementary Table 1. Optimization of the synthesis of isoquinoline 3aa.[a]

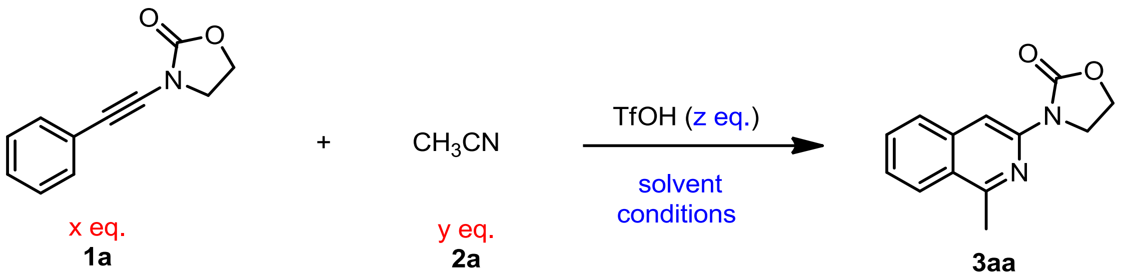

Entry	x	y	z	Solvent	Conditions	Yield[b]
1	1.0	5.0	1.0	DCM (0.4 M)	rt, 24 h	13%
2	1.0	5.0	1.0	DCE (0.4 M)	85 °C, 16 h	26%
3	1.0	2.0	1.0	DCE (0.4 M)	80 °C, 16 h	42%
4	1.0	2.0	1.0	neat	80 °C, 16 h	10%
5	1.1	1.0	1.1	DCE (0.4 M)	80 °C, 16 h	50%
6	1.1	1.0	1.1	DCE (0.1 M)	110 °C, 16 h	78%
7	1.1	1.0	0.2	DCE (0.1 M)	110 °C, 16 h	12%
8	1.1	1.0	1.1	DCE (0.05 M)	110 °C, 16 h	85%
9	1.1	1.0	1.1	DCE (0.05 M)	110 °C, 16 h	60%[c]
**10**	**1.1**	**1.0**	**1.1**	**DCE (0.05 M)**	**120 °C, 1 h**	**89%[d]**

[a] All reactions were conducted in 0.2 mmol scale. [b] Yield of isolated product. [c] $HNTf_2$ was used instead of TfOH. [d] Microwave irradiation for 1 h. DCM = dichloromethane, DCE = 1, 2-dichloroethane.

Supplementary Table 2. Optimization of the synthesis of pyrimidine **5aa**.[a]

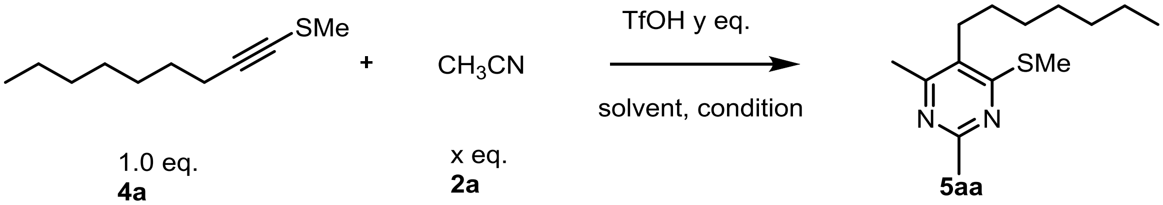

Entry	X	Y	Solvent	condition	Yield (%)[b]
1	2.1	1.0	DCE, 0.05 M	110 ˚C, 12 h	36
2	2.1	1.0	DCE, 0.05 M	50 ˚C, 24 h	36
3	2.1	1.0	DCM, 0.05 M	rt, 24 h	46
4	-	1.0	CH_3CN, 0.05 M	rt, 24 h	67
5	-	0.2	CH_3CN, 0.05 M	rt, 24 h	10
6	-	1.0	CH_3CN, 0.1 M	80 ˚C, 16 h	73
7	-	1.0	CH_3CN, 0.1 M	rt, 16 h	73
8	5.0	1.0	DCM, 0.1 M	rt, 16 h	71

[a] All reactions were conducted in 0.2 mmol scale. [b] Yield of isolated product. DCM = dichloromethane, DCE = 1, 2-dichloroethane.

Supplementary Table 3. Experimental parameter and CCDC-Code.

Sample	Machine	Source	Temp.	Detector Distance	Time/ Frame	#Frames	Frame width	CCDC
	Bruker		[K]	[mm]	[s]		[°]	
Laxi607	D8	Mo	100 (2)	35	20	3528	0.4	1423496

Supplementary Table 4. Sample and crystal data of [**laxi607**].

**Chemical formula**	C24H26Br2N2S	**Crystal system**	orthorhombic	
**Formula weight [g/mol]**	534.35	**Space group**	*Pna2₁*	
**Temperature [K]**	100	**Z**	4	
**Measurement method**	\Φ and \ω scans	**Volume [Å^3]**	2276.7(5)	
**Radiation (Wavelength [Å])**	MoKα (λ = 0.71073)	**Unit cell dimensions [Å] and [°]**	20.254(3)	90
**Crystal size [mm^3]**	0.536 × 0.114 × 0.058		24.972(3)	90
**Crystal habit**	clear colorless block		4.5015(6)	90
**Density (calculated) [g/cm^3]**	1.559	**Absorption coefficient [mm^{-1}]**	3.666	
**Abs. correction Tmin**	0.5168	**Abs. correction Tmax**	0.746	
**Abs. correction type**	multi-scan	**F(000) [e$^-$]**	1080	

Supplementary Table 5. Data collection and structure refinement of [**laxi607**].

**Index ranges**	-24 ≤ h ≤ 24, -30 ≤ k ≤ 30, -4 ≤ l ≤ 5	**Theta range for data collection [°]**	3.832 to 50.696	
**Reflections number**	22002	**Data / restraints / parameters**	3953/1/264	
**Refinement method**	Least squares	**Final R indices**	all data	R1 = 0.0487, wR2 = 0.0769
**Function minimized**	Σ w(F_o^2 - F_c^2)^2		I>2σ(I)	R1 = 0.0362, wR2 = 0.0734
**Goodness-of-fit on F^2**	1.024	**Weighting scheme**	w=1/[σ^2(F_o^2)+(0.0391P)^2+ 0.8315P]	
**Largest diff. peak and hole [e Å^{-3}]**	0.47/-0.41		where P=(F_o^2+2F_c^2)/3	

Supplementary method

General information

All reactions were carried out in flame-dried glassware under an atmosphere of argon. All solvents were distilled from appropriate drying agents prior to use. All reagents were used as received from commercial suppliers unless otherwise stated. Neat infra-red spectra were recorded using a Perkin-Elmer Spectrum 100 FT-IR spectrometer. Wavelengths (ν) are reported in cm^{-1}. Mass spectra were obtained using a Finnigan MAT 8200 (70 eV) or an Agilent 5973 (70 eV) spectrometer, using electrospray ionization (ESI). Accurate mass determinations were obtained on a Bruker APEX III FT-MS (7 T magnet). All ^{1}H-NMR and ^{13}C-NMR experiments were recorded using Bruker AV-400 spectrometers. Chemical shifts (δ) are quoted in ppm and coupling constants (*J*) are quoted in Hz. Reaction progress was monitored by thin layer chromatography (TLC) performed on aluminum plates coated with kieselgel F254 with 0.2 mm thickness. Visualization was achieved by a combination of ultraviolet light (254 nm) and potassium permanganate. Flash column chromatography was performed using silica gel 60 (230-400 mesh, Merck and co.).

General procedure for the synthesis of starting materials

General procedure A:

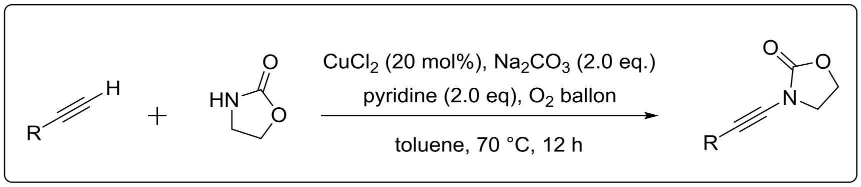

To a flask were added $CuCl_2$ (20 mol%), 2-oxazolidone or sulfonamide (5.0 eq.) and Na_2CO_3 (2.0 eq.). The reaction flask was purged with oxygen for 15 min. A solution of pyridine (2.0 eq.) in dry toluene (0.2 M) was added. A balloon filled with oxygen was connected to the flask and the flask was heated at 70 °C. After 15 min, a solution of alkyne (1.0 eq.) in dry toluene (0.2 M) was added over 4 h using syringe pump. After this addition, the mixture was allowed to stir at 70 °C for another 12 h and was then cooled to rt. The reaction mixture was concentrated under reduced pressure and the residue was purified by flash chromatography on silica gel with hexane/ethyl acetate.

General procedure B:

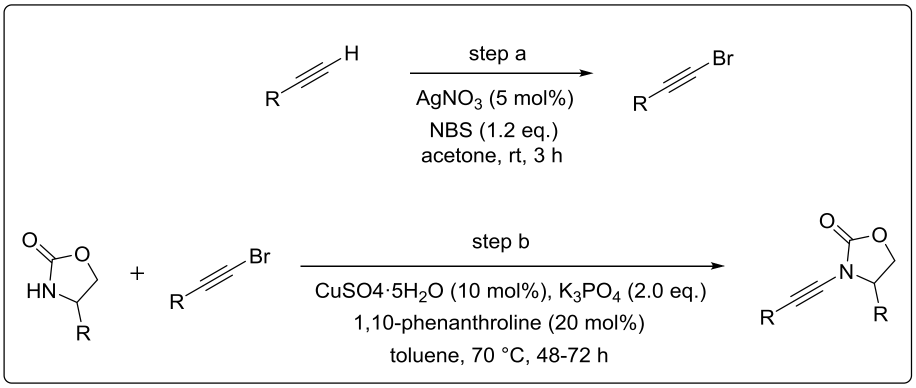

Step a, synthesis of alkyne bromide: A solution of alkyne (1.0 eq.), *N*-bromosuccinimide (1.2 eq.), and silver nitrate (5 mol%) in acetone (0.3 M) was stirred at room temperature for 3 h. Acetone was then removed on a rotary evaporator. The resulting product was dissolved in pentane, and the solution was passed through a short silica gel column. The reaction mixture was then concentrated under reduced pressure giving alkyne bromide as a light yellow liquid.

Step b, cross coupling of oxazolidinone with alkyne bromide: Alkyne bromide (1.0 eq.) obtained from step a, oxazolidinone (1.2 eq.), K_3PO_4 (2.0 eq.), $CuSO_4{\cdot}5H_2O$ (10 mol%), 1,10-phenanthroline (20 mol%) in toluene (0.5 M) was heated at 80 °C for 48 h. After cooling to room temperature, the reaction mixture was concentrated under reduced pressure and the residue was purified by flash chromatography on silica gel.

General procedure C:

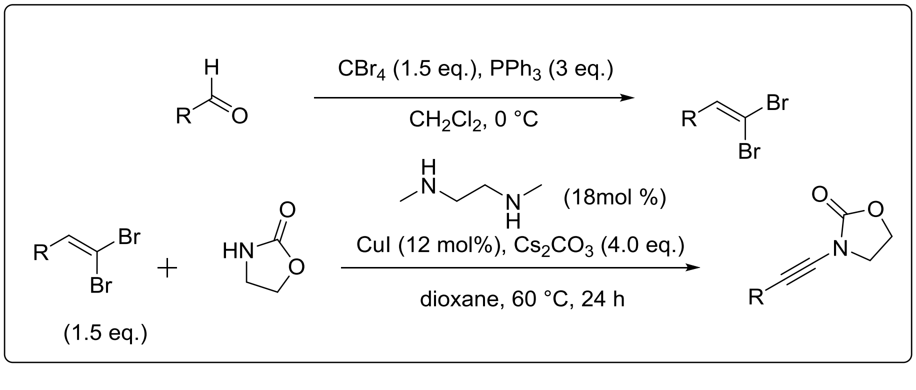

Step a, synthesis of 1,1-dibromoalkene: A round-bottomed flask was charged with aldehydes (10 mmol), CBr_4 (15 mmol) and CH_2Cl_2 (80 mL). Then the flask was placed in an ice-water bath. A solution of PPh_3 (30

mmol) in CH_2Cl_2 (70 mL) was added through dropping funnel in 15 min. TLC analysis was performed until the spot of carbonyl compound disappeared. In general, the reaction finished in 15 minutes and white precipitate formed. Then petroleum ether (100 mL) was added and more precipitate would form. The precipitate was filtrated off, and washed with petroleum ether or ethyl ether. After solvent evaporated off in vacuum, the product was isolated by silicagel chromatography.

Step b, cross coupling of oxazolidinone: A 15 mL pressure tube was charged with oxazolidinone (1.6 mmol), 1,1-dibromo-1-alkene (2.4 mmol), Cs_2CO_3 (2.1 g, 6.4 mmol), and copper(I) iodide (38 mg, 0.2 mmol). The tube was fitted with a rubber septum, evacuated under high vacuum and backfilled with argon. Dry and degassed 1,4-dioxane or DMF (3 mL) and *N*,*N'*-dimethylethylenediamine (30 µL, 0.3 mmol) were next added, the rubber septa was replaced by Teflon-coated screw cap and the light blue-green suspension was heated at the temperature. The brownish suspension was cooled to rt. When the reaction was run in 1,4-dioxane, the crude reaction mixture was filtered over a plug of silica gel (washed with EtOAc), and concentrated. When the reaction was run in DMF, the crude reaction mixture was diluted with water, extracted with diethyl ether and the combined organic layers were washed with brine, dried over $MgSO_4$, filtered and concentrated. The crude residue was in both cases purified by flash chromatography over silica gel.

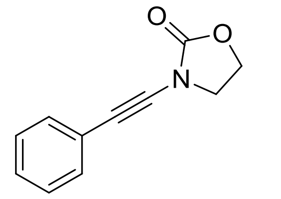

1a

3-(phenylethynyl)oxazolidin-2-one (1a)

General procedure B was performed with phenyl acetylene (1.13 g, 10 mmol) and 2-oxazolidone (1.05 g, 12 mmol). Purification by flash column chromatography (hexane/ethyl acetate = 1/1) afforded the title compound (1.5 g, 83%) as white solid.

IR (neat) ν_{max}: 2986, 2918, 2259, 1760, 1701, 1477, 1420, 1198, 1164, 1083, 1032, 970, 748, 693, 625;

^{1}H-NMR (400 MHz, $CDCl_3$): δ 7.46-7.42 (m, 2H), 7.32-7.28 (m, 3H), 4.50-4.46 (m, 2H), 4.02-3.98 (m, 2H);

^{13}C-NMR (100 MHz, $CDCl_3$): δ 156.0, 131.7 (2C), 128.4 (2C), 128.3, 122.3, 79.1, 71.3, 63.2, 47.2;

HRMS (ESI+): exact mass calculated for $[M+Na]^+$ ($C_{11}H_9NaNO_2$) requires *m/z* 210.0633, found *m/z* 210.0518.

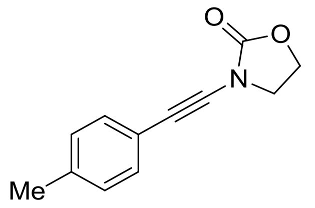

1b

3-(p-tolylethynyl)oxazolidin-2-one (1b)

General procedure B was performed with 4-dimethylphenyl acetylene (1.16 mg, 10 mmol) and 2-oxazolidone (1.04 g, 12 mmol). Purification by flash column chromatography (hexane/ethyl acetate = 1/1) afforded the title compound (1.5 g, 75%) as white solid.

IR (neat) ν_{max}: 2986, 2915, 2260, 1756, 1604, 1476, 1415, 1339, 1215, 1164, 1090, 1030, 970, 840, 746, 706;

^{1}H-NMR (400 MHz, $CDCl_3$): δ 7.33 (d, *J* = 8.0, 0.9 Hz, 2H), 7.11 (d, *J* = 8.0 Hz, 2H), 4.49-4.45 (m, 2H), 4.01-3.97 (m, 2H), 2.34 (s, 3H);

^{13}C-NMR (100 MHz, $CDCl_3$): δ 156.1, 138.5, 131.8 (2C), 129.2 (2C), 119.2, 78.4, 71.4, 63.1, 47.2, 21.5;

HRMS (ESI+): exact mass calculated for $[M+Na]^+$ ($C_{12}H_{11}NaNO_2$) requires *m/z* 224.0790, found *m/z* 224.0673.

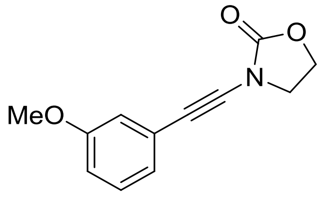

1c

3-((3-methoxyphenyl)ethynyl)oxazolidin-2-one (1c)

General procedure B was performed with 3′,4′-dimethoxyphenyl acetylene (500 mg, 3.8mmol) and 2-oxazolidone (394 mg, 4.4 mmol). Purification by flash column chromatography (hexane/ethyl acetate = 1/1) afforded the title compound (500 mg, 61%) as white solid.

IR (neat) ν_{max}: 2920, 2255, 1761, 1600, 1576, 1477, 1453, 1428, 1407, 1249, 1201, 1144, 1091, 1036, 972, 783, 748, 712, 688, 628;

^{1}H-NMR (400 MHz, $CDCl_3$): δ 7.21 (t, *J* = 7.9, 1H), 7.03 (dt, *J* = 7.6, 1.3 Hz, 1H), 6.97 (dd, J = 2.6, 1.3 Hz, 1H), 6.86 (ddd, *J* = 8.3, 2.6, 0.95 Hz, 1H), 4.51-4.47 (m, 2H), 4.03-3.99 (m, 2H), 3.80 (s, 3H);

^{13}C-NMR (100 MHz, $CDCl_3$): δ 159.5, 156.0, 129.5, 124.2, 123.4, 116.4, 115.0, 78.9, 71.4, 63.2, 55.5, 47.2;

HRMS (ESI+): exact mass calculated for $[M+Na]^+$ ($C_{12}H_{11}NaNO_3$) requires *m/z* 240.0739, found *m/z* 240.0624.

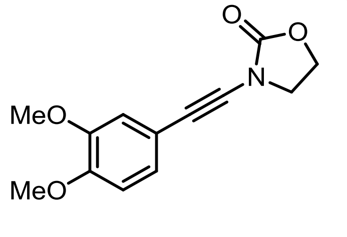

1d

3-((3,4-dimethoxyphenyl)ethynyl)oxazolidin-2-one (1d)

General procedure A was performed with 3′,4′-dimethoxyphenyl acetylene (162 mg, 1 mmol) and 2-oxazolidone (435 mg, 5 mmol). Purification by flash column chromatography (hexane/ethyl acetate = 1/1) afforded the title compound (175 mg, 59%) as white solid.

IR (neat) ν_{max}: 2934, 2255, 1768, 1516, 1423, 1401, 1235, 1137, 1024, 749;

^{1}H-NMR (400 MHz, $CDCl_3$): δ 7.05 (dd, *J* = 8.3, 1.9 Hz, 1H), 6.96 (d, *J* = 1.8 Hz, 1H), 6.80 (d, *J* = 8.4 Hz, 1H), 4.46-4.50 (m, 2H), 4.02-3.98 (m, 2H), 3.88 (s, 3H), 3.87 (s, 3H);

^{13}C-NMR (100 MHz, $CDCl_3$): δ 156.1, 149.7, 148.8, 125.4, 114.9, 114.4, 111.1, 77.6, 71.3, 63.1, 56.1, 56.0, 47.3;

HRMS (ESI+): exact mass calculated for $[M+Na]^+$ ($C_{13}H_{13}NaNO_4$) requires *m/z* 270.0742, found *m/z* 270.0743.

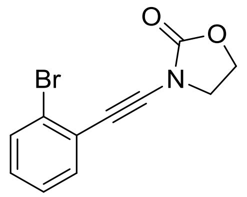

1e

3-((2-bromophenyl)ethynyl)oxazolidin-2-one (1e)

General procedure C was performed with 2-dimethoxyphenyl acetylene (1 g, 8 mmol) and 2-oxazolidone (1.05 g, 20 mmol). Purification by flash column chromatography (hexane/ethyl acetate = 1/1) afforded the title compound (570 mg, 57%) as white solid.

IR (neat) ν_{max}: 2921, 2852, 2257, 1758, 1634, 1478, 1408, 1252, 1198, 1119, 1088, 1028, 971, 749, 624;

^{1}H-NMR (400 MHz, $CDCl_3$): δ 7.53 (dd, *J* = 8.1, 0.95 Hz, 1H), 7.42 (dd, *J* = 7.8, 1.7 Hz, 1H), 7.21 (td, J = 7.6, 1.2 Hz, 1H), 7.11 (td, *J* = 7.8, 1.7 Hz, 1H), 4.47-4.43 (m, 2H), 4.01-3.97 (m, 2H);

^{13}C-NMR (100 MHz, $CDCl_3$): δ 155.6, 132.9, 132.3, 129.2, 127.0, 124.9, 124.5, 83.5, 70.2, 63.3, 46.9;

HRMS (ESI+): exact mass calculated for $[M+Na]^+$ ($C_{11}H_8BrNaNO_2$) requires *m/z* 287.9738, found *m/z* 287.9640.

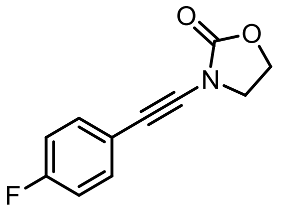

1f

3-((4-fluorophenyl)ethynyl)oxazolidin-2-one (1f)

General procedure A was performed with 1-Ethynyl-4-fluorobenzene (240 mg, 2 mmol) and 2-oxazolidone (871 mg, 10 mmol). Purification by flash column chromatography (DCM to DCM/Methanol =100/1) afforded the title compound (244 mg, 59%) as white solid.

IR (neat) ν_{max}: 2910, 2267, 1754, 1469, 1423, 1216, 1165, 1090, 834;

^{1}H-NMR (400 MHz, $CDCl_3$): δ 7.44-7.41 (m, 2H), 7.03-6.98 (m, 2H), 4.51-4.47 (m, 2H), 4.02-3.98 (m, 2H);

^{13}C-NMR (100 MHz, $CDCl_3$): δ 162.7 (d, $J_{F\text{-}C}$ = 249 Hz), 156.0, 133.8 (d, $J_{F\text{-}C}$ = 8.1 Hz, 2C), 118.4 (d, $J_{F\text{-}C}$ = 3.7 Hz), 115.8 (d, $J_{F\text{-}C}$ = 22 Hz, 2C), 78.7, 70.3, 63.2, 47.2;

HRMS (ESI+): exact mass calculated for $[M+Na]^+$ ($C_{11}H_8FNaNO_2$) requires *m/z* 228.0437, found *m/z* 228.0424.

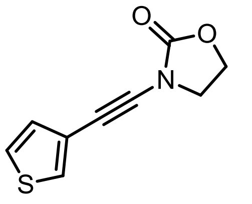

1g

3-(thiophen-2-ylethynyl)oxazolidin-2-one (1g)

General procedure A was performed with 3-Ethynylthiophene (216 mg, 2 mmol) and 2-oxazolidone (871 mg, 10 mmol). Purification by flash column chromatography (DCM to DCM/Methanol =100/1) afforded the title compound (236 mg, 61%) as white solid.

IR (neat) ν_{max}: 3107, 2919, 2256, 1775, 1477, 1436, 1198, 1085, 973, 784;

^{1}H-NMR (400 MHz, $CDCl_3$): δ 7.46 (s, 1H), 7.26 (m, 1H), 7.11 (d, *J* = 4.9 Hz, 1H), 4.48 (m, 2H), 3.99 (m, 2H);

^{13}C-NMR (100 MHz, $CDCl_3$): δ 156.0, 130.2, 129.3, 125.4, 121.1, 78.5, 66.5, 63.2, 47.2;

HRMS (ESI+): exact mass calculated for $[M+Na]^+$ ($C_9H_7NaNO_2S$) requires *m/z* 216.0095, found *m/z* 216.0091.

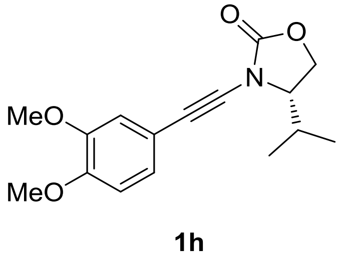

1h

(S)-3-((3,4-dimethoxyphenyl)ethynyl)-4-isopropyloxazolidin-2-one (1h)

General procedure B was performed with 3′,4′-dimethoxyphenyl acetylene (162 mg, 1 mmol) and (*S*)-4-isopropyl 2-oxazolidone (155 mg, 1.2 mmol). Purification by flash column chromatography (hexane/ethyl acetate = 1/1) afforded the title compound (100 mg, 35%) as white solid.

IR (neat) ν_{max}: 2961, 2838, 2252, 1770, 1596, 1489, 1463, 1421, 1320, 1262, 1237, 1198, 1137, 1087, 1023, 854, 810, 764, 617;

^{1}H-NMR (400 MHz, $CDCl_3$): δ 7.04 (dd, *J* = 8.3, 1.9 Hz, 1H), 6.95 (d, *J* = 1.9 Hz, 2H), 6.79 (d, *J* = 8.3 Hz, 1H), 4.41 (t, *J* = 8.3 Hz, 1H), 4.21-4.17 (m, 1H), 4.05-4.01 (m, 1H), 3.88 (s, 3H), 3.87 (s, 3H), 1.04-1.01 (m, 6H);

^{13}C-NMR (100 MHz, $CDCl_3$): δ 156.2, 149.7, 148.8, 125.3, 114.8, 114.6, 111.1, 77.07, 72.3, 64.9, 62.3, 56.1, 56.0, 29.4, 17.4, 15.4;

HRMS (ESI+): exact mass calculated for $[M+Na]^+$ ($C_{16}H_{19}NaNO_4$) requires *m/z* 312.1314, found *m/z* 312.1210.

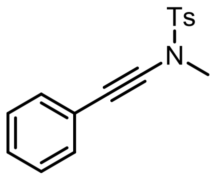

1i

N,4-dimethyl-N-(phenylethynyl)benzenesulfonamide (**1i**)

General procedure A was performed with phenyl acetylene (204 mg, 2 mmol) and N-methyl-p-toluenesulfonamide (1.85 g, 10 mmol). Purification by flash column chromatography (DCM to DCM/Methanol =100/1) afforded the title compound (348 mg, 61%) as white solid.

IR (neat) ν_{max}: 3059, 2929, 2234, 1597, 1365, 1158, 1089, 964, 755;

^{1}H-NMR (400 MHz, $CDCl_3$): δ 7.84 (d, *J* = 8.3 Hz, 2H), 7.38-7.35 (m, 4H), 7.30-7.28 (m, 3H), 3.16 (s, 3H), 2.46 (s, 3H);

^{13}C-NMR (100 MHz, $CDCl_3$): δ 144.9, 133.5 (2C), 131.6 (2C), 130.0 (2C), 128.4 (2C), 128.0, 128.0, 122.9, 84.1, 69.2, 39.5, 21.8;

HRMS (ESI+): exact mass calculated for $[M+Na]^+$ ($C_{16}H_{15}NaNO_2S$) requires *m/z* 308.0721, found *m/z* 308.0712.

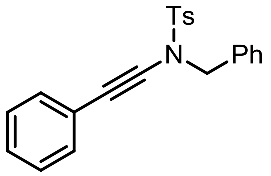

1j

N,4-dimethyl-N-(phenylethynyl)benzenesulfonamide (**1j**)

General procedure A was performed with phenyl acetylene (306 mg, 3 mmol) and N-benzyl-p-toluenesulfonamide (3.92 g, 15 mmol). Purification by flash column chromatography (DCM to DCM/Methanol =100/1) afforded the title compound (629 mg, 58%) as white solid.

IR (neat) ν_{max}: 3032, 1699, 1597, 1453, 1352, 1164, 1087, 697;

^{1}H-NMR (400 MHz, $CDCl_3$): δ 7.80 (d, *J* = 8.3 Hz, 2H), 7.34-7.30 (m, 7H), 7.24 (s, 5H), 4.59 (s, 2H), 2.45 (s, 3H);

^{13}C-NMR (100 MHz, $CDCl_3$): δ 144.8, 134.9, 134.6, 131.3, 129.9, 129.0, 128.7, 128.5, 128.3, 127.9, 127.8, 123.0, 82.9, 71.6, 55.9, 21.8;

HRMS (ESI+): exact mass calculated for $[M+Na]^+$ ($C_{22}H_{19}NaNO_2S$) requires *m/z* 384.1034, found *m/z* 384.1030.

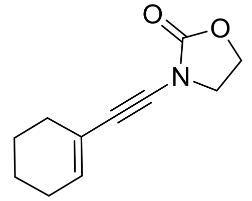

1k

3-(cyclohex-1-en-1-ylethynyl)oxazolidin-2-one (1k)

General procedure A was performed with cyclohexyl acetylene (1 g, 1 mmol) and 2-oxazolidone (435 mg, 5 mmol). Purification by flash column chromatography (hexane/ethyl acetate = 1/1) afforded the title compound (1.2 g, 67%) as white solid.

IR (neat) ν_{max}: 2931, 2851, 2248, 1760, 1699, 1479, 1419, 1326, 1200, 1140, 1071, 1034, 919, 749, 658;

^{1}H-NMR (400 MHz, $CDCl_3$): δ 6.13-6.10 (m, 1H), 4.45-4.40 (m, 2H), 3.92-3.88 (m, 2H), 2.14-2.06 (m, 4H), 1.66-1.55 (m, 4H);

^{13}C-NMR (100 MHz, $CDCl_3$): δ 156.2, 135.6, 119.7, 76.6, 72.9, 63.0, 47.3, 29.5, 25.8, 22.5, 21.6;

HRMS (ESI+): exact mass calculated for $[M+Na]^+$ ($C_{11}H_{13}NaNO_2$) requires *m/z* 214.0946, found *m/z* 214.0830.

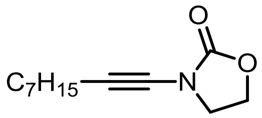

1l

3-(non-1-yn-1-yl)oxazolidin-2-one (1l)

General procedure A was performed with 1-nonyne (373 mg, 3 mmol) and 2-oxazolidone (1306 mg, 15 mmol). Purification by flash column chromatography (hexane/ethyl acetate = 7/3) afforded the title compound (330 mg, 37%) as yellowish oil.

IR (neat) ν_{max}: 2924, 2855, 2268, 1763, 1412, 1196, 1109, 1034, 749;

^{1}H-NMR (400 MHz, $CDCl_3$): δ 4.42-4.38 (m, 2H), 3.88-3.84 (m, 2H), 2.29 (t, *J* = 7.2 Hz, 2H), 1.55-1.49 (m, 2H), 1.41-1.35 (m, 2H), 1.32-1.26 (m, 6H), 0.88 (t, *J* = 7.0 Hz, 3H);

^{13}C-NMR (100 MHz, $CDCl_3$): δ 156.7, 71.4, 70.2, 62.9, 47.2, 31.9, 29.0 (3C), 22.8, 18.6, 14.2;

HRMS (ESI+): exact mass calculated for $[M+Na]^+$ ($C_{12}H_{19}NaNO_2$) requires *m/z* 232.1313, found *m/z* 232.1302.

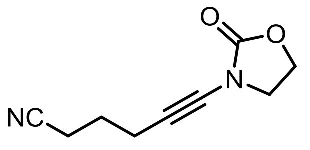

1m

6-(2-oxazolidin-3-yl)hex-5-ynenitrile (1m)

General procedure A was performed with 5-cyano-1-pentyne (466 mg, 5 mmol) and 2-oxazolidone (2177 mg, 25 mmol). Purification by flash column chromatography (hexane/ethyl acetate = 1/1) afforded the title compound (330 mg, 37%) as yellowish oil.

IR (neat) ν_{max}: 2920, 2270, 2248, 1760, 1478, 1414, 1303, 1201, 1113, 1033, 973, 750;

^{1}H-NMR (400 MHz, $CDCl_3$): δ 4.44-4.40 (m, 2H), 3.90-3.86 (m, 2H), 2.53-2.49 (m, 4H), 1.93-1.86 (m, 2H);

^{13}C-NMR (100 MHz, $CDCl_3$): δ 156.6, 119.3, 72.1, 68.5, 63.0, 46.9, 24.7, 17.8, 16.3;

HRMS (ESI+): exact mass calculated for $[M+Na]^+$ ($C_9H_{10}NaN_2O_2$) requires *m/z* 201.0640, found *m/z* 201.0638.

General procedure for the synthesis of isoquinolines

Under Argon atmosphere, mixture of ynamide (0.22 mmol, 1.1 eq.) and nitrile (0.2 mmol, 1.0 eq.) in dichloroethane (4 mL) was added dropwise of TfOH (0.22 mmol, 1.1 eq.) at 0 °C. The reaction was warm up immediately to RT for 30 min. Then the reaction was irradiated with microwave at 120 °C for 1 h or reflux at 120 °C for 16 h. The reaction was quenched with Na_2CO_3 sat. sol. (5 mL) after cooled to rt, extracted with DCM (3 x 5 mL), dried over Na_2SO_4 and the solvent was removed in vacuo. The crude product was purified by column chromatography on silica gel with hexane/ethyl acetate (7/3 or 1/1).

Characterization of structurally new compounds

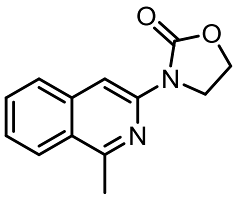

3aa

3-(1-methylisoquinolin-3-yl)oxazolidin-2-one (3aa)

IR (neat) ν_{max}: 2996, 2928, 1740, 1699, 1388, 1223, 1117, 1040, 750;

^{1}H-NMR (400 MHz, $CDCl_3$): δ 8.34 (s, 1H), 8.03 (dd, *J* = 8.4, 0.9 Hz, 1H), 7.81 (d, *J* = 8.4 Hz, 1H), 7.61 (m, 1H), 7.46 (m, 1H), 4.51 (m, 2H), 4.41 (m, 2H), 2.90 (s, 3H);

^{13}C-NMR (100 MHz, $CDCl_3$): δ 157.6, 155.4, 145.0, 138.0, 130.3, 127.6, 125.7, 125.5, 124.9, 106.0, 62.2, 44.6, 22.2;

HRMS (ESI+): exact mass calculated for $[M+Na]^+$ ($C_{13}H_{12}NaN_2O_2$) requires *m/z* 251.0796, found *m/z* 251.0785.

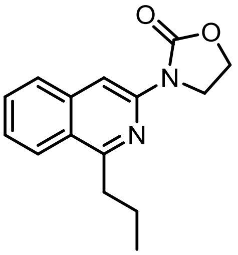

3ab

3-(1-propylisoquinolin-3-yl)oxazolidin-2-one (**3ab**)

IR (neat) ν_{max}: 2959, 2925, 1754, 1701, 1568, 1401, 1197, 1033, 754;

^{1}H-NMR (400 MHz, $CDCl_3$): δ 8.33 (s, 1H), 8.07 (dd, *J* = 8.5, 0.9 Hz, 1H), 7.81 (d, *J* = 8.3 Hz, 1H), 7.59 (m, 1H), 7.45 (m, 1H), 4.51 (m, 2H), 4.42 (m, 2H), 3.21 (t, *J* = 7.7 Hz, 2H), 1.91 (m, 2H), 1.05 (t, *J* = 7.4 Hz, 3H);

^{13}C-NMR (100 MHz, $CDCl_3$): δ 160.9, 155.4, 145.0, 138.3, 130.1, 127.8, 125.6, 125.1, 124.4, 105.8, 62.2, 44.6, 36.7, 22.2, 14.3;

HRMS (ESI+): exact mass calculated for $[M+Na]^+$ ($C_{15}H_{16}NaN_2O_2$) requires *m/z* 279.1109, found *m/z* 279.1100.

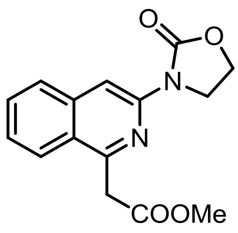

3ac

methyl 2-(3-(2-oxooxazolidin-3-yl)isoquinolin-1-yl)acetate (**3ac**)

IR (neat) ν_{max}: 2922, 2853, 1739, 1589, 1430, 1247, 1118, 752;

^{1}H-NMR (400 MHz, $CDCl_3$): δ 8.44 (s, 1H), 7.96 (dd, *J* = 8.5, 0.8 Hz, 1H), 7.84 (d, *J* = 8.5 Hz, 1H), 7.63 (m, 1H), 7.48 (m, 1H), 4.51 (m, 2H), 4.38 (m, 2H), 4.29 (s, 2H), 3.73 (s, 3H);

^{13}C-NMR (100 MHz, $CDCl_3$): δ 170.8, 155.3, 153.1, 145.0, 138.5, 130.6, 127.8, 126.3, 124.8, 124.6, 107.3, 62.2, 52.3, 44.5, 41.7;

HRMS (ESI+): exact mass calculated for $[M+Na]^+$ ($C_{15}H_{14}NaN_2O_2$) requires *m/z* 309.0851, found *m/z* 309.0843.

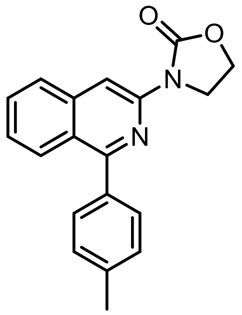

3ad

3-(1-(p-tolyl)isoquinolin-3-yl)oxazolidin-2-one (**3ad**)

IR (neat) ν_{max}: 3029, 2971, 2919, 1755, 1620, 1586, 1557, 1480, 1446, 1422, 1405, 1389, 1364, 1227, 1185, 1134, 115, 1050, 1033, 879, 854, 829, 752, 725, 693, 621

^{1}H-NMR (400 MHz, $CDCl_3$): δ 8.48 (s, 1H), 8.07 (dd, *J* = 8.5, 0.9 Hz, 1H), 7.88 (d, *J* = 8.4 Hz, 1H), 7.63 (m, 3H), 7.41 (m, 1H), 7.35 (dd, *J* = 7.8, 0.6 Hz, 2H), 4.51 (m, 2H), 4.44 (m, 2H), 2.48 (s, 3H);

^{13}C-NMR (100 MHz, $CDCl_3$): δ 159.1, 155.5, 145.3, 139.1, 138.9, 136.5, 130.3, 130.1 (2C), 129.2 (2C), 127.5, 127.4, 125.9, 124.1, 106.8, 62.3, 44.6, 21.5;

HRMS (ESI+): exact mass calculated for $[M+Na]^+$ ($C_{19}H_{16}N_2NaO_2$) requires *m/z* 327.1109, found *m/z*. 327.1112.

3-(1-(4-bromophenyl)isoquinolin-3-yl)oxazolidin-2-one (**3ae**)

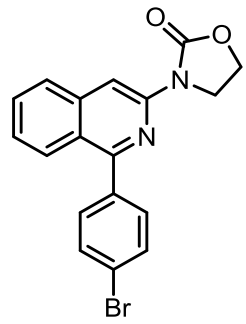

3ae

IR (neat) ν_{max}: 1753, 1621, 1586, 1404, 1226, 1117, 1012, 853;

^{1}H-NMR (400 MHz, $CDCl_3$): δ 8.52 (s, 1H), 7.99 (dd, *J* = 8.6, 0.9 Hz, 1H), 7.90 (d, *J* = 8.4 Hz, 1H), 7.66 (m, 3H), 7.60 (m, 2H), 7.43 (m, 1H), 4.52 (m, 2H), 4.42 (m, 2H);

^{13}C-NMR (100 MHz, $CDCl_3$): δ 157.8, 155.4, 145.3, 139.2, 138.2, 131.8 (2C), 131.7 (2C), 130.5, 127.6, 126.9, 126.2, 123.8, 123.4, 107.3, 62.3, 44.6;

HRMS (ESI+): exact mass calculated for $[M+Na]^+$ ($C_{18}H_{13}BrN_2NaO_2$) requires *m/z* 391.0058, found *m/z* 391.0056.

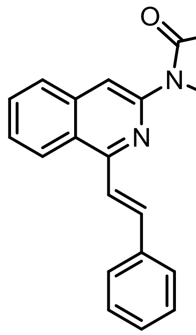

3af

(E)-3-(1-styrylisoquinolin-3-yl)oxazolidin-2-one (3af)

IR (neat) ν_{max}: 2920, 2853, 1745, 1579, 1553, 1445, 1400, 1115, 743, 690;

^{1}H-NMR (400 MHz, $CDCl_3$): δ 8.42 (s, 1H), 8.25 (dd, *J* = 8.6, 0.7 Hz, 1H), 7.92 (s, 2H), 7.81 (d, *J* = 8.4 Hz, 1H), 7.67 (m, 2H), 7.60 (m, 1H), 7.50-7.41 (m, 3H), 7.35 (m, 1H), 4.57-4.47 (m, 4H);

^{13}C-NMR (100 MHz, $CDCl_3$): δ 155.4, 152.6, 145.3, 139.0, 136.9, 135.9, 130.2, 129.0 (2C), 128.9, 127.7, 127.6 (2C), 125.9, 124.2, 124.1, 122.4, 107.2, 62.2, 44.5;

HRMS (ESI+): exact mass calculated for $[M+Na]^+$ ($C_{20}H_{16}N_2NaO_2$) requires *m/z* 339.1109, found *m/z* 339.1103.

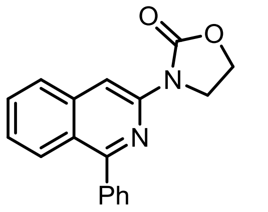

3ag

3-(1-phenylisoquinolin-3-yl)oxazolidin-2-one (**3ag**)

IR (neat) ν_{max}: 3058, 2917, 1748, 1620, 1401, 1244, 1116, 1050, 752;

^{1}H-NMR (400 MHz, $CDCl_3$): δ 8.50 (s, 1H), 8.05 (dd, *J* = 8.6, 0.9 Hz, 1H), 7.89 (d, *J* = 8.4 Hz, 1H), 7.71 (m, 2H), 7.63 (m, 1H), 7.52 (m, 3H), 7.41 (m, 1H), 4.51 (m, 2H), 4.44 (m, 2H);

^{13}C-NMR (100 MHz, $CDCl_3$): δ 159.0, 155.5, 145.3, 139.3, 139.1, 130.3, 130.2 (2C), 128.9, 128.4 (2C), 127.5, 127.4, 126.0, 124.0, 107.0, 62.2, 44.6;

HRMS (ESI+): exact mass calculated for $[M+Na]^+$ ($C_{18}H_{14}N_2NaO_2$) requires *m/z* 313.0953, found *m/z* 313.0947.

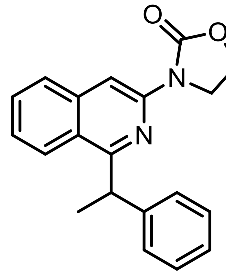

3ah

3-(1-(1-phenylethyl)isoquinolin-3-yl)oxazolidin-2-one (**3ah**)

IR (neat) ν_{max}: 2970, 2924, 1752, 1589, 1425, 1400, 1246, 1118, 751;

^{1}H-NMR (400 MHz, $CDCl_3$): δ 8.37 (s, 1H), 8.12 (d, *J* = 8.6 Hz, 1H), 7.79 (d, *J* = 8.3 Hz, 1H), 7.54 (m, 1H), 7.38 (m, 1H), 7.32 (m, 2H), 7.26 (m, 2H), 7.17 (m, 1H), 5.05 (q, 1H), 4.55-4.43 (m, 4H), 1.80 (d, *J* = 7.0 Hz, 3H);

^{13}C-NMR (100 MHz, $CDCl_3$): δ 161.9, 155.4, 145.6, 144.8, 138.7, 130.0, 128.6 (2C), 127.8, 127.7 (2C), 126.4, 125.8, 125.0, 124.0, 106.1, 62.2, 44.6, 42.9, 21.9;

HRMS (ESI+): exact mass calculated for $[M+Na]^+$ ($C_{20}H_{18}N_2NaO_2$) requires *m/z* 341.1266, found *m/z* 341.1262.

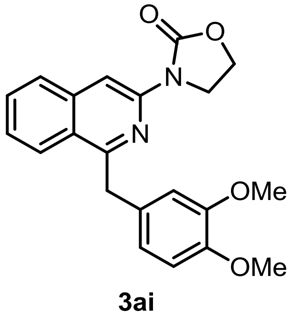

3ai

3-(1-(3,4-dimethoxybenzyl)isoquinolin-3-yl)oxazolidin-2-one (**3ai**)

IR (neat) ν_{max}: 2921, 2834, 1749, 1587, 1511, 1423, 1400, 1232, 1115, 1027, 750;

^{1}H-NMR (400 MHz, $CDCl_3$): δ 8.38 (s, 1H), 8.09 (dd, *J* = 8.5, 0.8 Hz, 1H), 7.81 (d, *J* = 8.4 Hz, 1H), 7.58 (m, 1H), 7.42 (m, 1H), 6.83 (s, 1H), 6.76 (s, 1H), 6.76 (s, 1H), 4.53 (s, 2H), 4.49 (m, 2H), 4.34 (m, 2H), 3.82 (s, 3H), 3.81 (s, 3H);

^{13}C-NMR (100 MHz, $CDCl_3$): δ 159.1, 155.4, 149.0, 147.7, 145.0, 138.6, 131.9, 130.2, 127.8, 125.9, 125.4, 124.4, 121.0, 112.4, 111.3, 106.4, 62.2, 56.0 (2C), 44.5, 41.0;

HRMS (ESI+): exact mass calculated for $[M+Na]^+$ ($C_{21}H_{20}N_2NaO_4$) requires *m/z* 387.1321, found *m/z* 387.1316.

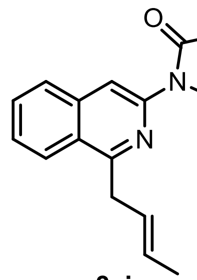

3aj

3-(1-(but-2-en-1-yl)isoquinolin-3-yl)oxazolidin-2-one (3aj)

IR (neat) ν_{max}: 2920, 1749, 1587, 1425, 1401, 1117, 750;

^{1}H-NMR (400 MHz, $CDCl_3$): δ 8.35 (s, 1H), 8.08 (d, *J* = 8.8 Hz, 1H), 7.82 (d, *J* = 8.2 Hz, 1H), 7.60 (m, 1H), 7.45 (m, 1H), 5.84-5.76 (m, 1H), 5.63-5.55 (m, 1H), 4.54-4.50 (m, 2H), 4.46-4.41 (m, 2H), 3.95 (dt, *J* = 6.9, 1.3 Hz, 2H), 1.68 (dt, *J* = 6.9, 1.5 Hz, 3H);

^{13}C-NMR (100 MHz, $CDCl_3$): δ 159.4, 155.4, 145.1, 138.4, 130.2, 128.0, 127.8, 127.3, 125.7, 125.4, 124.3, 106.2, 62.2, 44.6, 38.8, 18.1;

HRMS (ESI+): exact mass calculated for $[M+Na]^+$ ($C_{16}H_{16}N_2NaO_2$) requires *m/z* 291.1109, found *m/z* 291.1104.

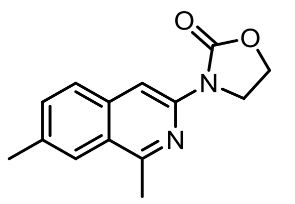

3ba

3-(1,7-dimethylisoquinolin-3-yl)oxazolidin-2-one (3ba)

IR (neat) ν_{max}: 2996, 2918, 1740, 1591, 1568, 1444, 1410, 1392, 1326, 1287, 1247, 1224, 1204, 1187, 1118, 1055, 1042, 868, 807, 755, 716;

^{1}H-NMR (400 MHz, $CDCl_3$): δ 8.28 (s, 1H), 7.78 (bs, 1H), 7.72 (d, *J* = 8.5 Hz, 1H), 7.45 (dd, *J* = 8.5, 1.6 Hz, 1H), 4.52-4.43 (m, 2H), 4.27-4.23 (m, 2H), 2.87 (s, 3H), 2.53 (s, 3H);

^{13}C-NMR (100 MHz, $CDCl_3$): δ 156.7, 155.4, 144.4, 136.2, 135.5, 132.6, 127.4, 125.0, 124.3, 106.0, 62.2, 44.6, 22.2, 22.1;

HRMS (ESI+): exact mass calculated for $[M+H]^+$ ($C_{14}H_{15}N_2O_2$) requires *m/z* 243.1055, found *m/z* 243.1055.

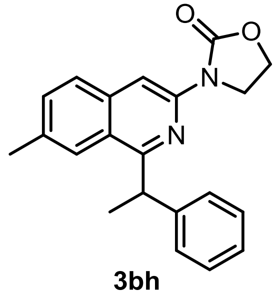

3bh

3-(7-methyl-1-(1-phenylethyl)isoquinolin-3-yl)oxazolidin-2-one (3bh)

IR (neat) ν_{max}: 2971, 2924, 1753, 1592, 1586, 1565, 1481, 1432, 1406, 1225, 1120, 1088, 1048, 872, 756, 703;

^{1}H-NMR (400 MHz, $CDCl_3$): δ 8.32 (bs, 1H), 7.88 (bs, 1H), 7.71 (d, *J* = 8.4 Hz, 1H), 7.40 (dd, *J* = 8.5, 1.5 Hz 1H), 7.34-7.31 (m, 2H), 7.27-7.23 (m, 2H), 7.18-7.14 (m, 1H), 5.03 (q, *J* = 8.4 Hz, 1H); 4.56-4.39 (m, 4H), 2.46 (bs, 3H),1.78 (d, *J* = 7.0 Hz, 3H)

^{13}C-NMR (100 MHz, $CDCl_3$): δ 161.1, 155.4, 145.6, 144.2, 137.0, 135.5, 132.4, 128.6 (2C), 127.8, 127.7 (2C), 126.4, 124.2, 123.7, 106.1, 62.2, 44.6, 42.7, 22.2, 22.0;

HRMS (ESI+): exact mass calculated for $[M+Na]^+$ ($C_{21}H_{20}N_2NaO_2$) requires *m/z* 355.1525, found *m/z* 355.1411.

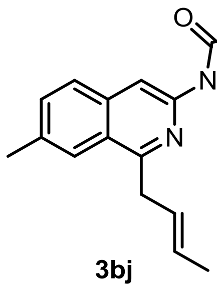

3bj

(E)-3-(1-(but-2-en-1-yl)-7-methylisoquinolin-3-yl)oxazolidin-2-one (3bj)

IR (neat) ν_{max}: 2987, 1754, 1422, 1264, 1147, 1044, 896, 731, 703;

^{1}H-NMR (400 MHz, $CDCl_3$): δ 8.30 (s, 1H), 7.82 (bs, 1H), 7.72 (d, *J* = 8.5 Hz, 1H), 7.44 (dd, *J* = 8.5, 1.7 Hz, 1H), 5.85-5.77 (m, 1H), 5.65-5.54 (m, 1H), 4.53-4.47 (m, 2H), 4.43-4.39 (m, 2H), 3.91 (dt, *J* = 6.4, 1.3 Hz, 2H), 2.52 (s, 3H), 1.69 (dq, *J* = 6.4, 1.4 Hz,3H);

^{13}C-NMR (100 MHz, $CDCl_3$): δ 158.5, 155.4, 144.5, 140.8, 136.7, 135.5, 132.5, 128.1, 127.6, 127.2, 124.5, 106.1, 62.2, 44.2, 38.7, 22.1, 18.2;

HRMS (ESI+): exact mass calculated for $[M+Na]^+$ ($C_{17}H_{18}N_2NaO_2$) requires *m/z* 305.1368, found *m/z* 305.1272.

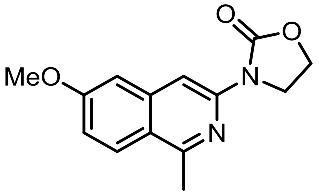

3ca

3-(6-methoxy-1-methylisoquinolin-3-yl)oxazolidin-2-one (3ca)

IR (neat) ν_{max}: 2990, 2920, 1754, 1619, 1575, 1414, 1390, 1244, 1218, 1120, 1073, 1058, 895, 815, 754, 708, 6197;

^{1}H-NMR (400 MHz, $CDCl_3$): δ 8.24 (s, 1H), 7.91 (d, *J* = 8.7, 1H), 7.09-7.06 (m, 2H), 4.52-4.49 (m, 2H), 4.41-4.37 (m, 2H), 3.92 (s, 3H), 3.83 (bs, 3H);

^{13}C-NMR (100 MHz, $CDCl_3$): δ 160.9, 156.8, 155.4, 145.6, 140.2, 127.3, 120.6, 118.9, 105.4, 104.8, 62.2, 55.6, 44.6, 22.2;

HRMS (ESI+): exact mass calculated for $[M+Na]^+$ ($C_{14}H_{14}N_2NaO_3$) requires *m/z* 281.1004, found *m/z* 281.0907.

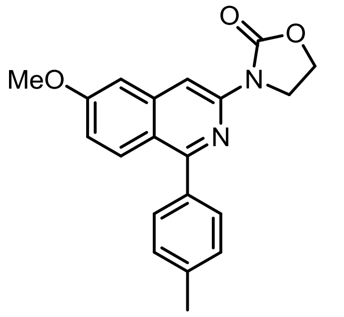

3cd

3-(6-methoxy-1-(p-tolyl)isoquinolin-3-yl)oxazolidin-2-one (3cd)

IR (neat) ν_{max}: 2995, 2921, 1745, 1621, 1592, 1560, 1463, 1424, 1227, 1164, 1118, 1050, 1023, 874, 833, 755, 713, 631;

^{1}H-NMR (400 MHz, $CDCl_3$): δ 8.28 (bs, 1H), 7.85 (d, *J* = 9.3, 1H), 7.48 (d, *J* = 8.3 Hz, 2H), 7.23 (d, *J* = 7.8 Hz, 2H), 7.04 (d, *J* = 2.5 Hz, 1H), 6.92 (dd, *J* = 9.3, 2.5 Hz, 1H), 4.43-4.38 (m, 2H), 4.35-4.31 (m, 2H), 3.84 (s, 3H), 2.36 (s, 3H);

^{13}C-NMR (100 MHz, $CDCl_3$): δ 160.9, 158.5, 155.5, 145.9, 141.3, 138.8, 136.6, 130.0 (2C), 129.2 (2C), 129.1, 119.9, 119.2, 106.0, 104.6, 62.3, 55.6, 44.6, 21.5;

HRMS (ESI+): exact mass calculated for $[M+H]^+$ ($C_{20}H_{19}N_2O_3$) requires *m/z* 335.1317, found *m/z*. 335.1385.

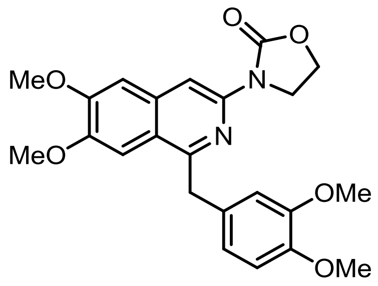

3di

3-(1-(3,4-dimethoxybenzyl)-6,7-dimethoxyisoquinolin-3-yl)oxazolidin-2-one (3di)

IR (neat) ν_{max}: 2932, 1754, 1511, 1422, 1256, 1214, 1158, 1121, 996, 758;

^{1}H-NMR (400 MHz, $CDCl_3$): δ 8.24 (s, 1H), 7.24 (s, 1H), 6.81 (s, 1H), 6.76 (s, 1H), 6.76 (s, 1H), 4.48 (m, 2H), 4.44 (s, 2H), 4.32 (t, 2H), 3.97 (s, 3H), 3.89 (s, 3H), 3.82 (s, 3H), 3.79 (s, 3H);

^{13}C-NMR (100 MHz, $CDCl_3$): δ 156.2, 155.4, 153.0, 149.2, 149.0, 147.7, 144.3, 135.6, 132.1, 120.8, 120.1, 112.3, 111.3, 105.6, 105.5, 103.6, 62.2, 56.1, 56.0 (2C), 55.9, 44.5, 41.5;

HRMS (ESI+): exact mass calculated for $[M+Na]^+$ ($C_{23}H_{24}N_2NaO_6$) requires *m/z* 447.1532, found *m/z* 447.1531.

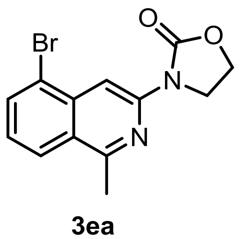

3ea

3-(5-bromo-1-methylisoquinolin-3-yl)oxazolidin-2-one (3ea)

IR (neat) ν_{max}: 2970, 2922, 1750, 1612, 1581, 1480, 1439, 1417, 1390, 1320,1229, 1200, 1087, 1048, 864, 747, 633;

^{1}H-NMR (400 MHz, $CDCl_3$): δ 8.71 (s, 1H), 8.01 (dt, *J* = 8.5, 0.9 Hz, 1H), 7.91 (dd, *J* = 7.4, 0.9 Hz, 1H), 7.30 (dd, *J* = 8.5, 7.4 Hz, 1H), 4.55-4.50 (m, 2H), 4.43-4.39 (m, 2H), 2.91 (s, 3H);

^{13}C-NMR (100 MHz, $CDCl_3$): δ 158.3, 146.3, 137.3, 134.2, 128.3, 125.9, 125.8, 125.2, 122.6, 105.3, 62.1, 44.6, 22.5;

HRMS (ESI+): exact mass calculated for $[M+Na]^+$ ($C_{13}H_{11}BrNaN_2O_2$) requires *m/z* 307.0004, found *m/z* 307.0078.

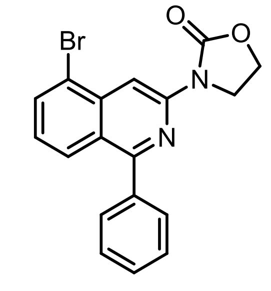

3eg

3-(5-bromo-1-phenylisoquinolin-3-yl)oxazolidin-2-one (3eg)

IR (neat) ν_{max}: 2988, 2916, 1755, 1610, 1578, 1479, 1439, 1420, 1389, 1322, 1229, 1135, 1122, 1048, 1030, 868, 751, 703, 647;

^{1}H-NMR (400 MHz, $CDCl_3$): δ 8.89 (s, 1H), 8.01 (d, *J* = 8.5 Hz, 1H), 7.93 (d, *J* = 7.4 Hz, 2H), 7.69-7.66 (m, 2H), 7.54-7.53 (m, 3H), 4.55-4.51 (m, 2H), 4.46-4.42 (m, 2H);

^{13}C-NMR (100 MHz, $CDCl_3$): δ 159.9, 155.3, 146.6, 139.0 (2C), 138.3, 134.2 (2C), 130.3, 129.2, 128.5, 127.3, 125.9, 125.1, 122.3, 106.3, 62.2, 44.5;

HRMS (ESI+): exact mass calculated for $[M+Na]^+$ ($C_{18}H_{13}BrNaN_2O_2$) requires *m/z* 391.0160, found *m/z* 391.0048.

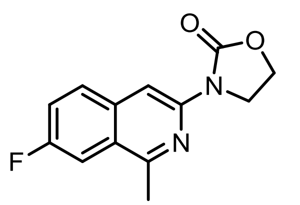

3fa

3-(7-fluoro-1-methylisoquinolin-3-yl)oxazolidin-2-one (3fa)

IR (neat) ν_{max}: 2994, 2970, 2921, 1752, 1589, 1482, 1444, 1411, 1371, 1242, 1183, 1133, 1117, 1067, 1044, 965, 930, 871, 756, 716;

^{1}H-NMR (400 MHz, $CDCl_3$): δ 8.35 (s, 1H), 7.83-7.80 (m, 1H), 7.62 (d, *J* = 9.9, 2.5 Hz, 1H), 7.41 (td, *J* = 8.6, 2.5 Hz, 1H), 4.54-4.50 (m, 2H), 4.42-4.37 (m, 2H), 2.85 (s, 3H);

^{13}C-NMR (100 MHz, $CDCl_3$): δ 161.3, 158.8, 156.8 (d, $J_{F\text{-}C}$ = 5.9 Hz), 155.4, 144.8(d, $J_{F\text{-}C}$ =2.8 Hz), 130.1 (d, $J_{F\text{-}C}$ = 8.3 Hz), 125.2 (d, $J_{F\text{-}C}$ = 7.9 Hz), 121.0 (d, $J_{F\text{-}C}$ = 25.5 Hz), 108.8 (d, $J_{F\text{-}C}$ = 21.0 Hz), 105.9 (d, $J_{F\text{-}C}$ = 1.2 Hz), 62.2, 44.6, 22.3;

HRMS (ESI+): exact mass calculated for $[M+Na]^+$ ($C_{13}H_{11}FN_2NaO_2$) requires *m/z* 269.0805, found *m/z* 269.0690.

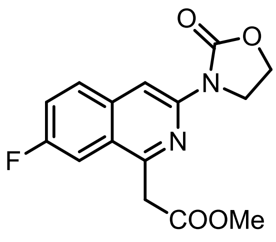

3fc

methyl 2-(7-fluoro-3-(2-oxooxazolidin-3-yl)isoquinolin-1-yl)acetate (3fc)

IR (neat) ν_{max}: 3094, 2995, 2850, 1752, 1590, 1482, 1440, 1409, 1341, 1244, 1175, 1118, 1060, 930, 875, 820, 756, 716;

^{1}H-NMR (400 MHz, $CDCl_3$): δ 8.47 (s, 1H), 7.88-7.84 (m, 1H), 7.57 (dd, *J* = 9.7, 2.0 Hz, 1H), 7.43 (td, *J* = 8.6 (2.4 Hz, 1H), 4.54-4.50 (m, 2H), 4.40-4.35 (m, 2H), 4.23 (s, 2H), 3.74 (s, 3H) ;

^{13}C-NMR (100 MHz, $CDCl_3$): δ 170.5, 161.6, 159.1, 155.3, (d, $J_{F\text{-}C}$ = 6.4 Hz), 144.9, 135.6, 130.3 (d, $J_{F\text{-}C}$ = 8.7 Hz), 125.0, 121.5 (d, $J_{F\text{-}C}$ = 25.8 Hz), 108.3(d, $J_{F\text{-}C}$ = 21.7 Hz), 107.3, 62.3, 52.5, 44.5, 41.7;

HRMS (ESI+): exact mass calculated for $[M+Na]^+$ ($C_{15}H_{13}FN_2NaO_2$) requires *m/z* 327.0859, found *m/z* 327.0746.

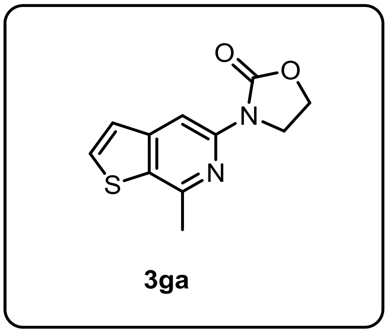

3-(7-methylthieno[2,3-c]pyridin-5-yl)oxazolidin-2-one (3ga)

IR (neat) ν_{max}: 2992, 2920, 1745, 1582, 1558, 1480, 1416, 1378, 1306, 1232, 1198, 1103, 1085, 1044, 859, 821, 753, 728;

^{1}H-NMR (400 MHz, $CDCl_3$): δ 8.41 (s, 1H), 7.63 (d, *J* = 5.4 Hz 1H), 7.34 (d, *J* = 5.4 Hz 1H), 4.52-4.47 (m, 2H), 4.38-4.34 (m, 2H), 2.71 (s, 3H);

^{13}C-NMR (100 MHz, $CDCl_3$): δ 155.4, 150.8, 147.3, 146.6, 132.2, 131.2, 124.2, 104.3, 62.1, 44.9, 23.4;

HRMS (ESI+): exact mass calculated for $[M+H]^+$ ($C_{11}H_{11}N_2O_2S$) requires *m/z* 235.0463, found *m/z* 235.0534.

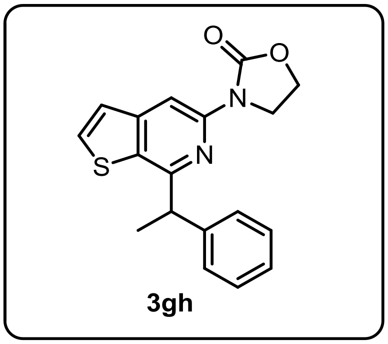

3-(7-(1-phenylethyl)thieno[2,3-c]pyridin-5-yl)oxazolidin-2-one (3gh)

IR (neat) ν_{max}: 3026, 2971, 2925, 1753, 1581, 1555, 1480, 1416, 1372, 1230, 1197, 1100, 863, 756, 732, 701;

^{1}H-NMR (400 MHz, $CDCl_3$): δ 8.51 (s, 1H), 7.66 (d, *J* = 5.4 Hz, 1H), 7.48 (m, d, *J* = 7.4 Hz, 2H), 7.40-7.34 (m, 3H), 7.30-7.26 (m, 1H), 4.64-4.50 (m, 5H), 1.87 (d, *J* = 6.9 Hz, 3H);

^{13}C-NMR (100 MHz, $CDCl_3$): δ 156.3, 155.4, 147.8, 146.5, 144.0, 132.3, 130.5, 128.5 (2C), 128.0 (2C), 126.8, 124.0, 104.5, 62.2, 46.7, 44.9, 20.7;

HRMS (ESI+): exact mass calculated for $[M+H]^+$ ($C_{18}H_{17}N_2O_2S$) requires *m/z* 325.0932, found *m/z* 325.1007.

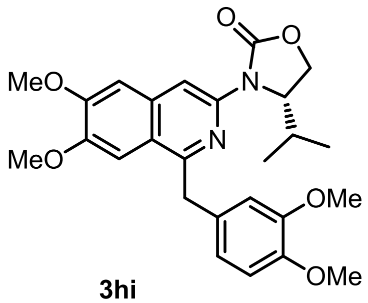

3hi

3-(1-(3,4-dimethoxybenzyl)-6,7-dimethoxyisoquinolin-3-yl)-4-isopropyloxazolidin-2-one (3hi)

IR (neat) ν_{max}: 3005, 2970, 1755, 1594, 1573, 1511, 1422, 1368, 1231, 1215, 1160, 1049, 874, 763;

^{1}H-NMR (400 MHz, $CDCl_3$): δ 8.19 (s, 1H), 7.28 (s, 1H), 7.09 (s, 1H), 6.79 (s, 1H), 6.79 (s, 1H), 6.76 (br s, 1H), 4.86 (dt, *J* = 8.8, 3.6 Hz, 1H), 4.49 (d, *J* = 15.2 Hz, 1H), 4.40 (d, *J* = 20.8 Hz, 1H), 4.38 (dd, *J* = 9.0, 3.3 Hz, 1H), 4.26 (dd, *J* = 9.0, 3.9 Hz, 1H), 3.99 (s, 3H), 3.93 (s, 3H), 3.84 (s, 3H), 3.78 (s, 3H); 2.37-2.28 (m, 1H), 1.33 (d, *J* = 7.1 Hz, 3H), 1.18 (d, *J* = 7.0 Hz, 3H);

^{13}C-NMR (100 MHz, $CDCl_3$): δ 156.3, 155.9, 153.0, 149.4, 149.1, 147.7, 143.9, 135.7, 132.0, 121.2, 120.2, 112.5, .111.4, 107.6, 105.7, 103.6, 63.1, 59.3, 56.2, 56.1, 56.0, 55.9, 41.5, 28.5, 18.0, 14.6;

HRMS (ESI+): exact mass calculated for $[M+H]^+$ ($C_{26}H_{31}N_2O_6$) requires *m/z* 467.1743, found *m/z* 467.2104, found m/z 467.2181.

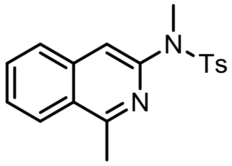

3ia

N,4-dimethyl-N-(1-methylisoquinolin-3-yl)benzenesulfonamide (3ia)

IR (neat) ν_{max}: 2957, 2853, 1626, 1588, 1375, 1176;

^{1}H-NMR (400 MHz, $CDCl_3$): δ 8.04 (d, *J* = 8.6, 1H), 7.83 (d, *J* = 8.4 Hz, 1H), 7.77 (s, 1H), 7.67-7.63 (m, 1H), 7.56-7.52 (m, 3H), 7.20 (d, *J* = 8.2 Hz, 2H), 3.54 (s, 3H), 2.78 (s, 3H), 2.39 (s, 3H);

^{13}C-NMR (100 MHz, $CDCl_3$): δ 158.0, 147.5, 143.5, 137.6, 135.4, 130.3, 129.4 (2C), 128.0 (2C), 127.8, 126.9, 126.1, 125.5, 115.1, 36.2, 22.0, 21.7;

HRMS (ESI+): exact mass calculated for $[M+Na]^+$ ($C_{18}H_{18}N_2NaO_2S$) requires *m/z* 349.0987, found *m/z* 349.0986.

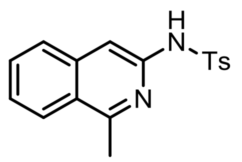

3ja

4-methyl-N-(1-methylisoquinolin-3-yl)benzenesulfonamide (3ja)

IR (neat) ν_{max}: 2922, 2853, 1663, 1623, 1445, 1371, 1155, 1090, 746, 661;

^{1}H-NMR (400 MHz, $CDCl_3$): δ 7.98 (dd, *J* = 8.4, 0.9 Hz, 1H), 7.81 (d, *J* = 8.3 Hz, 2H), 7.73 (d, *J* = 8.2 Hz, 1H), 7.60 (m, 1H), 7.54 (s, 1H), 7.44 (m, 1H), 7.21 (d, *J* = 8.0 Hz, 2H), 2.80 (s, 3H), 2.33 (s, 3H);

^{13}C-NMR (100 MHz, $CDCl_3$): δ 159.1, 144.4, 144.1, 138.0, 136.7, 130.7, 129.8 (2C), 127.5 (2C), 127.2, 125.9, 125.7, 125.3, 104.8, 21.9, 21.6;

HRMS (ESI+): exact mass calculated for $[M+H]^+$ ($C_{17}H_{17}N_2O_2S$) requires *m/z* 313.1011, found *m/z* 313.1001.

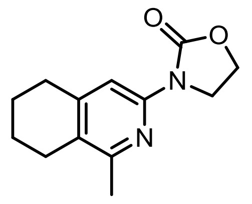

3ka

3-(1-methyl-5,6,7,8-tetrahydroisoquinolin-3-yl)oxazolidin-2-one (3ka)

IR (neat) ν_{max}: 2922, 2858, 1741, 1594, 1572, 1458, 1424, 1399, 1231, 1205, 1118, 1059, 1043, 980, 902, 756;

^{1}H-NMR (400 MHz, $CDCl_3$): δ 7.71 (s, 1H), 4.46-4.42 (m, 2H), 4.28-4.23 (m, 2H), 2.76 (t, *J* = 2.6 Hz, 2H), 2.58 (t, *J* = 2.6 Hz, 2H), 2.35 (s, 3H), 1.87-1.81 (m, 2H), 1.78-1.72 (m, 2H);

^{13}C-NMR (100 MHz, $CDCl_3$): δ 155.4, 154.9, 148.2, 147.4, 126.5, 110.5, 62.1, 44.3, 30.1, 25.8, 23.3, 22.4, 22.0;

HRMS (ESI+): exact mass calculated for $[M+H]^+$ ($C_{13}H_{17}N_2O_2$) requires *m/z* 233.1212, found *m/z* 233.1280.

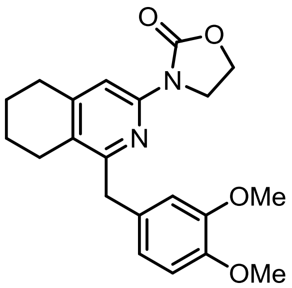

3ki

3-(1-(3,4-dimethoxybenzyl)-5,6,7,8-tetrahydroisoquinolin-3-yl)oxazolidin-2-one (3ki)

IR (neat) ν_{max}: 2995, 2927, 2856, 1755, 1592, 1566, 1513, 1453, 1404, 1320, 1232, 1140, 1114, 1029, 758;

^{1}H-NMR (400 MHz, $CDCl_3$): δ 7.76 (s, 1H), 6.77-6.75 (m, 2H), 6.68 (dd, *J* = 8.2, 1.9 Hz, 1H), 4.46-4.42 (m, 2H), 4.24-4.20 (m, 2H), 3.98 (m, 2H), 3.84 (s, 3H), 3.83 (s, 3H), 2.77 (t, *J* = 6.2 Hz, 2H), 2.62 (t, *J* = 6.2 Hz, 2H), 1.80-1.71 (m, 4H);

^{13}C-NMR (100 MHz, $CDCl_3$): δ 156.5, 155.3, 149.0, 148.9, 147.6, 147.5, 131.9, 126.6, 120.9, 112.5, 111.3, 110.9, 62.1, 56.1 (2C), 44.3, 40.8, 30.2, 25.4, 23.2, 22.3;

HRMS (ESI+): exact mass calculated for $[M+Na]^+$ ($C_{21}H_{24}N_2NaO_4$) requires *m/z* 391.1174, found *m/z* 391.1624.

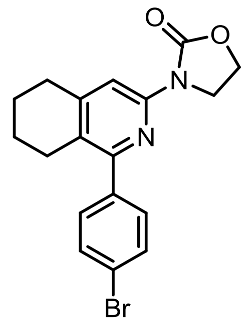

3ke

3-(1-(4-bromophenyl)-5,6,7,8-tetrahydroisoquinolin-3-yl)oxazolidin-2-one (3ke)

IR (neat) ν_{max}: 2923, 2853, 1758, 1460, 1511, 1377, 1400, 1264, 736, 706;

^{1}H-NMR (400 MHz, $CDCl_3$): δ 7.92 (s, 1H), 7.55 (d, *J* = 8.5 Hz, 2H), 7.37 (d, *J* = 8.5 Hz, 2H), 4.47-4.43 (m, 2H), 4.27-4.23 (m, 2H), 2.88 (t, *J* = 6.3 Hz, 2H), 2.64 (t, *J* = 6.3 Hz, 2H), 1.84-1.78 (m, 2H), 1.74-1.69 (m, 2H);

^{13}C-NMR (100 MHz, $CDCl_3$): δ 155.4, 155.0, 149.7, 148.0, 139.4, 131.3 (2C), 130.9 (2C), 126.5, 122.3, 112.0, 62.2, 44.3, 30.0, 27.4, 23.3, 22.4

HRMS (ESI+): exact mass calculated for $[M+Na]^+$ ($C_{18}H_{17}N_2BrNaO_2$) requires *m/z* 395.0473, found *m/z* 395.0377.

General procedure for the synthesis of thioalkynes

In a dry argon-flushed Schlenk flask, alkyne (5 mmol) was dissolved in THF (5 mL). The solution was cooled to -78 °C and nBuLi (5.5 mmol, 3.44 mL, 1.6 M in hexane) was added dropwise. After 10 min of stirring at -78 °C, dimethyl disulfide (565 mg, 6 mmol) was added and the solution was allowed to warm to room temperature and stirred for 1 h. The reaction mixture was quenched with saturated aqueous NH4Cl-solution and extracted with EtOAc (3 x 5 mL). The combined organic layers were dried over Na_2SO_4 and the solvent was removed in vacuo. The crude product was purified by column chromatography (Al_2O_3 netural, pentane).

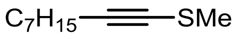

4a

methyl(non-1-yn-1-yl)sulfane (4a)

General procedure was performed with 1-nonyne (621 mg, 5 mmol) and nBuLi (5.5 mmol, 3.44 mL, 1.6 M in hexane) and dimethyl disulfide (565 mg, 6 mmol). Purification by column chromatography with Al_2O_3 (pentane) afforded the title compound (340 mg, 40%) as colourless oil.

IR (neat) ν_{max}: 2927, 2856, 1462, 1433, 1276, 976, 765;

^{1}H-NMR (400 MHz, $CDCl_3$): δ 2.35 (s, 3H), 2.28 (t, *J* = 7.1 Hz, 2H), 1.54-1.47 (m, 2H), 1.39-1.27 (m, 8H), 0.89 (t, *J* = 7.0 Hz, 3H);

^{13}C-NMR (100 MHz, $CDCl_3$): δ 93.5, 69.9, 31.9, 29.0, 28.9 (2C), 22.8, 20.2, 19.5, 14.2;

HRMS (ESI+): exact mass calculated for M^+ ($C_{10}H_{18}S$) requires *m/z* 170.1129, found *m/z* 170.1123.

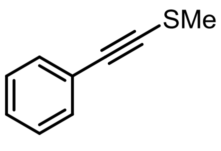

4b

methyl(phenylethynyl)sulfane (4b)

General procedure was performed with phenylacetylene (511 mg, 5 mmol) and nBuLi (5.5 mmol, 3.44 mL, 1.6 M in hexane) and dimethyl disulfide (565 mg, 6 mmol). Purification by column chromatography with Al_2O_3 (pentane) afforded the title compound (578 mg, 78%) as colourless oil.

IR (neat) ν_{max}: 2926, 2167, 1486, 1440, 1313, 977, 690;

^{1}H-NMR (400 MHz, $CDCl_3$): δ 7.43-7.40 (m, 2H), 7.30-7.28 (m, 3H), 2.48 (s, 3H);

^{13}C-NMR (100 MHz, $CDCl_3$): δ 131.6 (2C), 128.4 (2C), 128.2, 123.6, 92.0, 81.1, 19.6;

HRMS (ESI+): exact mass calculated for M^+ (C_9H_8S) requires *m*/*z* 148.0347, found *m*/*z* 148.0338.

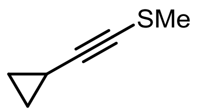

4c

(cyclopropylethynyl)(methyl)sulfane (4c)

General procedure was performed with cyclopropylacetylene (132 mg, 2 mmol) and nBuLi (2.2 mmol, 1.38 mL, 1.6 M in hexane) and dimethyl disulfide (283 mg, 3 mmol). Purification by column chromatography with Al_2O_3 (pentane) afforded the title compound (117 mg, 52%) as colourless oil.

IR (neat) ν_{max}: 2925, 2854, 2363, 2337, 1460, 1276, 914, 755;

^{1}H-NMR (400 MHz, $CDCl_3$): δ 2.33 (s, 3H), 1.36-1.30 (m, 1H), 0.81-0.76 (m, 2H), 0.75-0.69 (m, 2H);

^{13}C-NMR (100 MHz, $CDCl_3$): δ 97.2, 65.9, 19.6, 8.96 (2C), 0.82;

HRMS (ESI+): exact mass calculated for M^+ (C_6H_8S) requires *m*/*z* 112.0347, found *m*/*z* 112.0344.

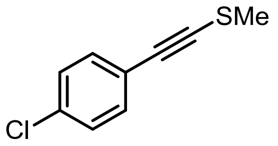

4d

((4-chlorophenyl)ethynyl)(methyl)sulfane (4d)

General procedure was performed with 1-chloro-4-ethynylbenzene (273 mg, 2 mmol) and nBuLi (2.2 mmol, 1.38 mL, 1.6 M in hexane) and dimethyl disulfide (283 mg, 3 mmol). Purification by column chromatography with Al_2O_3 (pentane) afforded the title compound (219 mg, 60%) as colourless oil.

IR (neat) ν_{max}: 2926, 2853, 2167, 1490, 1313, 1100, 1014, 830;

^{1}H-NMR (400 MHz, $CDCl_3$): δ 7.34-7.32 (m, 2H), 7.27-7.25 (m, 2H, overlap with $CHCl_3$), 2.48 (s, 3H);

^{13}C-NMR (100 MHz, $CDCl_3$): δ 134.2, 132.8 (2C), 128.8 (2C), 122.1, 90.9, 82.4, 19.5;

HRMS (ESI+): exact mass calculated for M^+ (C_9H_7SCl) requires *m/z* 181.9957, found *m/z* 181.9952.

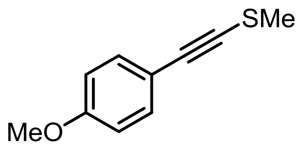

4e

((4-methoxyphenyl)ethynyl)(methyl)sulfane (4e)

General procedure was performed with 4-ethynylanisole (132 mg, 2 mmol) and nBuLi (2.2 mmol, 1.38 mL, 1.6 M in hexane) and dimethyl disulfide (283 mg, 3 mmol). Purification by column chromatography with Al_2O_3 (pentane) afforded the title compound (143 mg, 40%) as colourless oil.

IR (neat) ν_{max}: 3003, 2928, 2836, 1604, 1506, 1289, 1249, 1172, 1032, 832, 750;

^{1}H-NMR (400 MHz, $CDCl_3$): δ 7.37 (d, *J* = 8.9 Hz, 2H), 6.82 (d, *J* = 8.9 Hz, 2H), 3.80 (s, 3H), 2.46 (s, 3H);

^{13}C-NMR (100 MHz, $CDCl_3$): δ 159.8, 133.5 (2C), 115.7, 114.1 (2C), 91.8, 79.1, 55.4, 19.6;

HRMS (ESI+): exact mass calculated for M^+ ($C_{10}H_{10}OS$) requires *m/z* 178.0452, found *m/z* 178.0446.

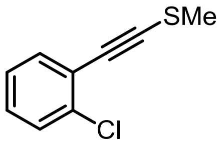

4f

((2-chlorophenyl)ethynyl)(methyl)sulfane (4f)

General procedure was performed with 1-chloro-2-ethynylbenzene (273 mg, 2 mmol) and nBuLi (2.2 mmol, 1.38 mL, 1.6 M in hexane) and dimethyl disulfide (283 mg, 3 mmol). Purification by column chromatography with Al_2O_3 (pentane) afforded the title compound (219 mg, 60%) as colourless oil.

IR (neat) ν_{max}: 3067, 2927, 2171, 1469, 1434, 1313, 1261, 1059, 750;

^{1}H-NMR (400 MHz, $CDCl_3$): δ 7.43-7.41 (m, 1H), 7.39-7.36 (m, 1H), 7.21-7.18 (m, 2H), 2.52 (s, 3H);

^{13}C-NMR (100 MHz, $CDCl_3$): δ 135.6, 132.9, 129.4, 128.9, 126.5, 123.5, 89.0, 87.1, 19.6;

HRMS (ESI+): exact mass calculated for M^+ (C_9H_7ClS) requires *m/z* 181.9957, found *m/z* 183.9952.

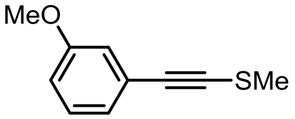

4g

((3-methoxyphenyl)ethynyl)(methyl)sulfane (4g)

General procedure was performed with 3-ethynylanisole (132 mg, 2 mmol) and nBuLi (2.2 mmol, 1.38 mL, 1.6 M in hexane) and dimethyl disulfide (283 mg, 3 mmol). Purification by column chromatography with Al_2O_3 (pentane) afforded the title compound (125 mg, 35%) as colourless oil.

IR (neat) ν_{max}: 3001, 2928, 2834, 2162, 1598, 1575, 1285, 1158, 1044, 777;

^{1}H-NMR (400 MHz, $CDCl_3$): δ 7.20 (t, *J* = 8.0 Hz, 1H), 7.01 (dt, *J* = 7.7, 1.2 Hz, 1H), 6.94 (dd, *J* = 1.5, 1.1 Hz, 1H), 6.85 (ddd, *J* = 8.3, 2.7, 1.0 Hz, 1H), 3.79 (s, 3H), 2.48 (s, 3H);

^{13}C-NMR (100 MHz, $CDCl_3$): δ 159.5, 129.5, 124.5, 124.1, 116.3, 114.9, 92.0, 81.0, 55.4, 19.5;

HRMS (ESI+): exact mass calculated for M^+ ($C_{10}H_{10}OS$) requires *m/z* 178.0452, found *m/z* 178.0444.

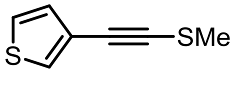

4h

3-((methylthio)ethynyl)thiophene (4h)

General procedure was performed with 3-Ethynylthiophene (216 mg, 2 mmol) and nBuLi (2.2 mmol, 1.38 mL, 1.6 M in hexane) and dimethyl disulfide (283 mg, 3 mmol). Purification by column chromatography with Al_2O_3 (pentane) afforded the title compound (170 mg, 55%) as colourless oil.

IR (neat) ν_{max}: 3105, 2925, 2163, 1432, 1354, 1311, 976, 785;

^{1}H-NMR (400 MHz, $CDCl_3$): δ 7.44 (dd, *J* = 2.9, 1.1 Hz, 1H), 7.24 (dd, *J* = 5.0, 2.9 Hz, 1H), 7.10 (dd, *J* = 5.0, 1.1 Hz, 1H), 2.46 (s, 3H);

^{13}C-NMR (100 MHz, $CDCl_3$): δ 130.2, 129.3, 125.3, 122.6, 86.9, 80.5, 19.5;

HRMS (ESI+): exact mass calculated for M^+ ($C_7H_6S_2$) requires *m/z* 153.9911, found *m/z* 153.9904.

General procedure for the synthesis of pyrimidines with methyl(non-1-yn-1-yl)sulfane.

Under Argon, TfOH (0.20 mmol, 1.0 eq.) was slowly added to a cold (0 ˚C) solution of thio-alkyne (0.2 mmol, 1.0 eq) and nitrile (1.0 mmol, 5.0 eq.) in DCM (2 mL). The result mixture was warm up to rt and stirred for 16 h at ambient temperature. The reaction was quenched with $NaHCO_3$ sat. sol. (5 mL) after cooled to rt, extracted with DCM (3 x 5 mL), dried over Na_2SO_4. 4-Dimethylaminopyridine (12.2 mg, 0.1 mmol) was added before the filtration. Then the solvent was removed in vacuo. After obtain the crude proton nmr, the crude product was purified by column chromatography on silica gel with hexane/ethyl acetate (20:1 or 5:1).

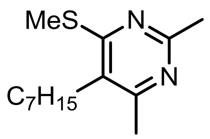

5aa

5-heptyl-2,4-dimethyl-6-(methylthio)pyrimidine (5aa)

IR (neat) ν_{max}: 2924, 2854, 1530, 1407, 1357, 1157, 854;

^{1}H-NMR (400 MHz, $CDCl_3$): δ 2.57-2.53 (m, 5H), 2.50 (s, 3H), 2.37 (s, 3H), 1.52-1.45 (m, 2H), 1.38-1.25 (m, 8H), 0.86 (t, *J* = 7.1 Hz, 3H);

^{13}C-NMR (100 MHz, $CDCl_3$): δ 167.9, 163.9, 161.4, 127.2, 31.9, 30.0, 29.1, 28.2, 27.9, 25.7, 22.7, 21.4, 14.2, 12.8;

HRMS (ESI+): exact mass calculated for $[M+H]^+$ ($C_{14}H_{25}N_2S$) requires *m/z* 253.1738, found *m/z* 253.1722.

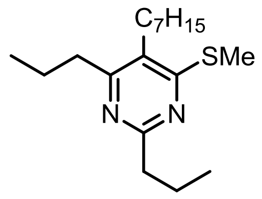

5ab

5-heptyl-4-(methylthio)-2,6-dipropylpyrimidine (5ab)

IR (neat) ν_{max}: 2959, 2927, 2349, 1550, 1464, 1403, 750;

^{1}H-NMR (400 MHz, $CDCl_3$): δ 2.79 (t, *J* = 7.7 Hz, 2H), 2.64-2.55 (m, 4H), 2.54 (s, 3H), 1.87-1.78 (m, 2H), 1.74-1.65 (m, 2H), 1.56-1.48 (m, 2H), 1.43-1.29 (m, 8H), 1.00-0.96 (m, 6H), 0.89 (t, *J* = 6.9 Hz, 3H);

^{13}C-NMR (100 MHz, $CDCl_3$): δ 168.0, 167.1, 164.9, 127.0, 41.2, 36.4, 31.9, 30.1, 29.1, 28.7, 28.0, 22.9, 22.8, 22.0, 14.3, 14.2, 14.1, 12.9;

HRMS (ESI+): exact mass calculated for $[M+H]^+$ ($C_{18}H_{33}N_2S$) requires *m/z* 309.2364, found *m/z* 309.2356.

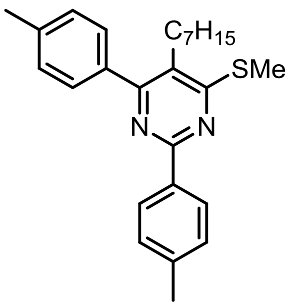

5ad

5-heptyl-4-(methylthio)-2,6-di-p-tolylpyrimidine (5ad)

IR (neat) ν_{max}: 2924, 2854, 1525, 1504, 1390, 1172, 767;

^{1}H-NMR (400 MHz, $CDCl_3$): δ 8.41 (d, *J* = 8.2 Hz, 2H), 7.47 (d, *J* = 8.1 Hz, 2H), 7.29 (d, *J* = 7.9 Hz, 2H), 7.26 (d, *J* = 8.0 Hz, 2H), 2.73 (s, 3H), 2.69 (t, *J* = 8.3 Hz, 2H), 2.44 (s, 3H), 2.42 (s, 3H), 1.61-1.54 (m, 2H), 1.29-1.23 (br, 8H), 0.87 (t, *J* = 7.1 Hz, 3H);

^{13}C-NMR (100 MHz, $CDCl_3$): δ 169.6, 163.1, 160.5, 140.4, 138.6, 136.5, 135.5, 129.2 (2C), 129.0 (2C), 128.9 (2C), 128.2 (2C), 127.8, 31.8, 29.8, 28.8, 28.6 (2C), 22.8, 21.6, 21.5, 14.2, 13.3;

HRMS (ESI+): exact mass calculated for $[M+H]^+$ ($C_{26}H_{33}N_2S$) requires *m/z* 405.2364, found *m/z* 405.2370.

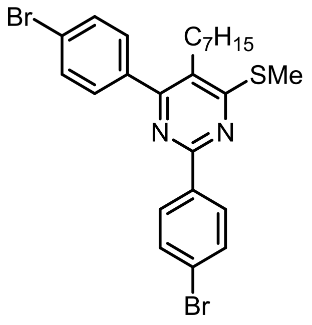

5ae

2,4-bis(4-bromophenyl)-5-heptyl-6-(methylthio)pyrimidine (5ae)

IR (neat) ν_{max}: 2925, 2854, 1522, 1486, 1389, 1011, 803;

^{1}H-NMR (400 MHz, $CDCl_3$): δ 8.36 (d, *J* = 8.7 Hz, 2H), 7.62 (d, *J* = 8.5 Hz, 2H), 7.57 (d, *J* = 8.7 Hz, 2H), 7.42 (d, *J* = 8.6 Hz, 2H), 2.71 (s, 3H), 2.65 (t, *J* = 8.2 Hz, 2H), 1.58-1.51 (m, 2H), 1.31-1.22 (br, 8H), 0.88 (t, *J* = 7.1 Hz, 3H);

^{13}C-NMR (100 MHz, $CDCl_3$): δ 170.5, 161.9, 159.7, 138.0, 136.8, 131.7 (2C), 131.6 (2C), 130.6 (2C), 129.8 (2C), 128.4, 125.2, 123.4, 31.8, 29.8, 28.8, 28.6, 28.5, 22.7, 14.2, 13.4;

HRMS (ESI+): exact mass calculated for $[M+H]^+$ ($C_{24}H_{27}Br_2N_2S$) requires *m/z* 533.0262, found *m/z* 533.0235.

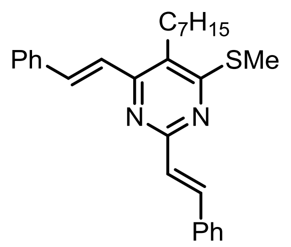

5af

5-heptyl-4-(methylthio)-2,6-di((E)-styryl)pyrimidine (5af)

IR (neat) ν_{max}: 2924, 2854, 1633, 1449, 1394, 971, 744;

^{1}H-NMR (400 MHz, $CDCl_3$): δ 8.09 (d, *J* = 15.6 Hz, 1H), 8.01 (d, *J* = 15.9 Hz, 1H), 7.64 (m, 4H), 7.43-7.38 (m, 4H), 7.36-7.31 (m, 2H), 7.27 (d, *J* = 15.5 Hz, 1H), 7.23 (d, *J* = 15.9 Hz, 1H), 2.78 (t, *J* = 8.2 Hz, 2H), 2.66 (s, 3H), 1.67-1.59 (m, 2H), 1.50-1.31 (br, 8H), 0.90 (t, *J* = 6.9 Hz, 3H);

^{13}C-NMR (100 MHz, $CDCl_3$): δ 169.5, 160.7, 156.5, 137.4, 136.9, 136.7 (2C), 129.1 (2C), 128.9 (2C), 128.8 (2C), 128.5 (2C), 127.8 (2C), 127.7, 127.0, 122.4, 31.9, 29.9, 29.2, 29.1, 27.7, 22.8, 14.2, 13.2;

HRMS (ESI+): exact mass calculated for $[M+H]^+$ ($C_{28}H_{33}N_2S$) requires *m/z* 429.2364, found *m/z* 429.2355.

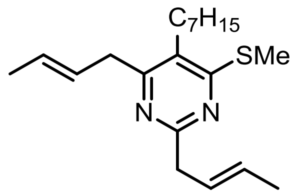

5aj

2,4-di((E)-but-2-en-1-yl)-5-heptyl-6-(methylthio)pyrimidine (5aj)

IR (neat) ν_{max}: 2926, 2854, 2337, 1536, 1276, 966, 750;

^{1}H-NMR (400 MHz, $CDCl_3$): δ 5.84-5.78 (m, 1H), 5.63-5.56 (m, 2H), 5.52-5.43 (m, 1H), 3.55 (dd, *J* = 6.8, 1.2 Hz, 2H), 3.39 (dd, *J* = 6.2, 1.3 Hz, 2H), 2.59-2.54 (m, 2+3H), 1.70 (dd, *J* = 6.4, 1.4 Hz, 3H), 1.66 (dd, *J* = 6.3, 1.4 Hz, 3H), 1.55-1.47 (m, 2H), 1.41-1.26 (br, 8H), 0.89 (t, *J* = 6.9 Hz, 3H);

^{13}C-NMR (100 MHz, $CDCl_3$): δ 168.7, 165.9, 163.4, 127.9, 127.6, 127.5, 127.3, 127.0, 42.7, 38.3, 31.9, 30.1, 29.1, 28.4, 27.9, 22.8, 18.2, 18.1, 14.2, 12.9;

HRMS (ESI+): exact mass calculated for $[M+H]^+$ ($C_{20}H_{33}N_2S$) requires *m/z* 333.2364, found *m/z* 333.2344.

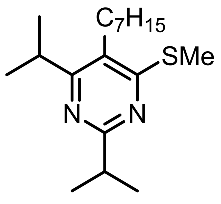

5ak

5-heptyl-2,4-diisopropyl-6-(methylthio)pyrimidine (5ak)

IR (neat) ν_{max}: 2960, 2925, 1540, 1467, 1400, 1161, 806;

^{1}H-NMR (400 MHz, $CDCl_3$): δ 3.13-3.07 (m, 2H), 2.60-2.56 (m, 2H), 2,55 (s, 3H), 1.55-1.48 (m, 2H), 1.45-1.30 (m, 8+6H), 1.22 (d, *J* = 6.7 Hz, 6H), 0.90 (t, *J* = 7.0 Hz, 3H);

^{13}C-NMR (100 MHz, $CDCl_3$): δ 171.2, 169.5, 167.5, 125.7, 37.4, 32.0, 30.6, 30.2, 29.2 (2C), 27.7, 22.8, 22.1 (2C), 21.9 (2C), 14.3, 12.9;

HRMS (ESI+): exact mass calculated for $[M+H]^+$ ($C_{18}H_{33}N_2S$) requires *m/z* 309.2364, found *m/z* 309.2357.

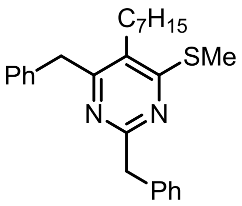

5al

2,4-dibenzyl-5-heptyl-6-(methylthio)pyrimidine (5al)

IR (neat) ν_{max}: 2925, 2854, 2328, 1535, 1392, 1276, 750;

^{1}H-NMR (400 MHz, $CDCl_3$): δ 7.41 (d, *J* = 7.4 Hz, 2H), 7.30 (t, *J* = 7.3 Hz, 2H), 7.24-7.17 (m, 6H), 4.19 (s, 2H), 4.06 (s, 2H), 2.54 (t, *J* = 8.6 Hz, 2H), 2.47 (s, 3H), 1.33-1.25 (m, 10H), 0.90 (t, *J* = 7.2 Hz, 3H);

^{13}C-NMR (100 MHz, $CDCl_3$): δ 169.3, 165.6, 163.2, 139.0, 138.6, 129.6 (2C), 128.8 (2C), 128.5 (2C), 128.3 (2C), 128.1, 126.5, 126.4, 45.8, 41.0, 31.9, 30.1, 29.0, 28.2, 28.1, 22.8, 14.2, 13.0;

HRMS (ESI+): exact mass calculated for $[M+H]^+$ ($C_{26}H_{33}N_2S$) requires *m/z* 405.2364, found *m/z* 405.2359.

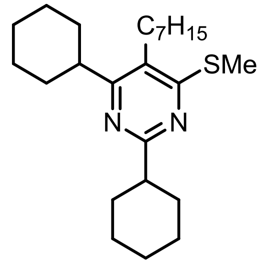

5am

2,4-dicyclohexyl-5-heptyl-6-(methylthio)pyrimidine (5am)

IR (neat) ν_{max}: 2923, 2851, 1533, 1449, 1401, 1098, 871;

^{1}H-NMR (400 MHz, $CDCl_3$): δ 2.77-2.70 (m, 2H), 2.59-2.55 (m, 2H), 2.54 (s, 3H), 2.02-1.99 (br, 2H), 1.83-1.70 (m, 8H), 1.64-1.60 (m, 4H), 1.53-1.27 (m, 16H), 0.90 (t, *J* = 6.9 Hz, 3H);

^{13}C-NMR (100 MHz, $CDCl_3$): δ 170.2, 168.6, 167.3, 125.9, 47.1, 41.4, 32.0 (2C), 31.9 (2C), 30.0 (2C), 29.2, 29.1, 27.7, 26.7 (2C), 26.4 (2C), 26.1 (2C), 22.8, 14.3, 12.9;

HRMS (ESI+): exact mass calculated for $[M+H]^+$ ($C_{24}H_{41}N_2S$) requires *m/z* 389.2990, found *m/z* 389.2990.

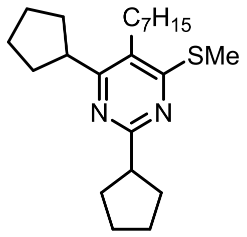

5an

2,4-dicyclopentyl-5-heptyl-6-(methylthio)pyrimidine (5an)

IR (neat) ν_{max}: 2924, 2856, 1534, 1463, 1405, 1097, 930;

^{1}H-NMR (400 MHz, $CDCl_3$): δ 3.27-3.19 (m, 2H), 2.60 (t, *J* = 8.1 Hz, 2H), 2.53 (s, 3H), 2.03-1.91 (m, 2H), 1.90-1.84 (br, 6H), 1.82-1.77 (m, 2H), 1.69-1.63 (m, 4H), 1.53-1.47 (m, 2H), 1.42-1.29 (br, 10H), 0.91-0.88 (m, 3H);

^{13}C-NMR (100 MHz, $CDCl_3$): δ 170.5, 168.3, 167.2, 126.4, 48.6, 42.1, 33.5 (2C), 32.9 (2C), 32.0, 30.1, 29.2 (2C), 27.9, 26.5 (2C), 26.1 (2C), 22.8, 14.3, 12.9;

HRMS (ESI+): exact mass calculated for $[M+H]^+$ ($C_{22}H_{37}N_2S$) requires *m/z* 361.2677, found *m/z* 361.2673.

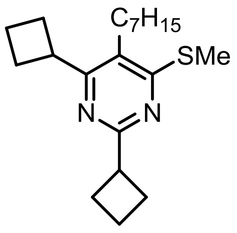

5ao

2,4-dicyclobutyl-5-heptyl-6-(methylthio)pyrimidine (5ao)

IR (neat) ν_{max}: 2928, 2856, 1534, 1410, 1307, 1098, 750;

^{1}H-NMR (400 MHz, $CDCl_3$): δ 3.75-3.66 (m, 2H), 2.57 (s, 3H), 2.55-2.46 (m, 6H), 2.38-2.29 (m, 2H), 2.24-2.16 (m, 2H), 2.07-2.00 (m, 2H), 1.98-1.88 (m, 2H), 1.50-1.29 (br, 10H), 0.90 (t, *J* = 7.0 Hz, 3H);

^{13}C-NMR (100 MHz, $CDCl_3$): δ 169.1, 167.3, 166.1, 126.3, 43.4, 38.2, 32.0, 30.2, 29.2, 28.8, 28.0 (2C), 27.8, 27.6 (2C), 22.8, 18.6, 18.5, 14.3, 12.9;

HRMS (ESI+): exact mass calculated for $[M+H]^+$ ($C_{20}H_{33}N_2S$) requires *m/z* 333.2364, found *m/z* 333.2361.

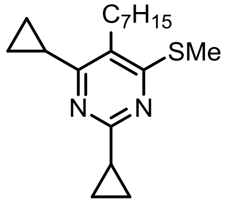

5ap

2,4-dicyclopropyl-5-heptyl-6-(methylthio)pyrimidine (5ap)

IR (neat) ν_{max}: 2924, 2853, 1535, 1430, 1310, 919;

^{1}H-NMR (400 MHz, $CDCl_3$): δ 2.68 (t, *J* = 8.2 Hz, 2H), 2.49 (s, 3H), 2.08-1.94 (m, 2H), 1.60-1.53 (m, 2H), 1.43-1.28 (br, 8H), 1.14-1.10 (m, 2H), 1.05-1.01 (m, 2H), 0.94-0.87 (m, 7H);

^{13}C-NMR (100 MHz, $CDCl_3$): δ 167.5, 166.6, 165.1, 126.2, 32.0, 30.0, 29.2, 28.8, 27.7, 22.8, 17.9, 14.2, 12.8, 12.7, 9.9 (2C), 9.8 (2C);

HRMS (ESI+): exact mass calculated for $[M+H]^+$ ($C_{18}H_{29}N_2S$) requires *m/z* 305.2051, found *m/z* 305.2045.

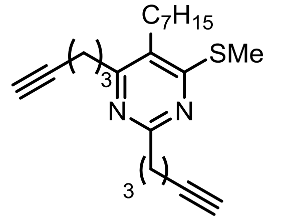

5aq

5-heptyl-4-(methylthio)-2,6-di(pent-4-yn-1-yl)pyrimidine (5aq)

IR (neat) ν_{max}: 2925, 2853, 2330, 1633, 1555, 1390, 1276, 750;

^{1}H-NMR (400 MHz, $CDCl_3$): δ 2.93 (t, *J* = 7.3 Hz, 2H), 2.78 (t, *J* = 7.8 Hz, 2H), 2.60 (t, *J* = 8.1 Hz, 2H), 2.54 (s, 3H), 2.29 (qd, *J* = 6.6, 2.2 Hz, 4H), 2.10-2.02 (m, 2H), 1.99-1.89 (m, 4H), 1.55-1.49 (m, 2H), 1.43-1.29 (br, 8H), 0.89 (t, *J* = 6.9 Hz, 3H);

^{13}C-NMR (100 MHz, $CDCl_3$): δ 168.4, 166.2, 164.0, 127.5, 84.5, 84.2, 69.0, 68.6, 37.9, 32.8, 32.0, 30.1, 29.1, 28.6, 27.9, 27.7, 27.1, 22.8, 18.4, 18.3, 14.2, 13.0;

HRMS (ESI+): exact mass calculated for $[M+H]^+$ ($C_{22}H_{33}N_2S$) requires *m/z* 357.2364, found *m/z* 357.2345.

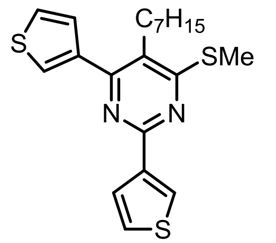

5ar

5-heptyl-4-(methylthio)-2,6-di(thiophen-3-yl)pyrimidine (5ar)

IR (neat) ν_{max}: 2923, 2852, 1517, 1430, 1343, 1086, 846, 776;

^{1}H-NMR (400 MHz, $CDCl_3$): δ 8.28 (dd, *J* = 3.2, 1.0 Hz, 1H), 7.93 (dd, *J* = 5.0, 1.0 Hz, 1H), 7.68 (dd, *J* = 3.1, 1.2 Hz, 1H), 7.49 (dd, *J* = 5.0, 1.2 Hz, 1H), 7.41 (dd, *J* = 3.0, 2.1 Hz, 1H), 7.34 (dd, *J* = 3.1, 1.9 Hz, 1H), 2.77 (t, *J* = 8.5 Hz, 2H), 2.68 (s, 3H), 1.67-1.60 (m, 2H), 1.38-1.29 (br, 8H), 0.90 (t, *J* = 7.0 Hz, 3H);

^{13}C-NMR (100 MHz, $CDCl_3$): δ 170.1, 157.9, 157.5, 142.1, 140.0, 128.9, 127.7, 127.3 (2C), 126.3, 125.7, 125.6, 31.9, 29.9, 29.0, 28.8, 28.5, 22.8, 14.2, 13.3;

HRMS (ESI+): exact mass calculated for $[M+H]^+$ ($C_{20}H_{25}N_2S_3$) requires *m/z* 389.1180, found *m/z* 389.1179.

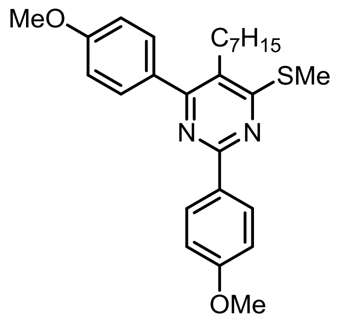

5as

5-heptyl-2,4-bis(4-methoxyphenyl)-6-(methylthio)pyrimidine (5as)

IR (neat) ν_{max}: 2925, 2853, 1607, 1503, 1390, 1247, 1163, 1031, 875;

^{1}H-NMR (400 MHz, $CDCl_3$): δ 8.47 (d, *J* = 8.9 Hz, 2H), 7.54 (d, *J* = 8.8 Hz, 2H), 7.01-6.96 (m, 4H), 3.88 (s, 3H), 3.87 (s, 3H), 2.71 (s, 3H), 2.70-2.67 (m, 2H), 1.61-1.54 (m, 2H), 1.30-1.18 (br, 8H), 0.87 (t, *J* = 7.1 Hz, 3H);

^{13}C-NMR (100 MHz, $CDCl_3$): δ 169.5, 162.5, 161.6, 160.2 (2C), 131.9, 130.9, 130.4 (2C), 129.8 (2C), 127.2, 113.8 (4C), 55.5 (2C), 31.8, 29.8, 28.9, 28.6 (2C), 22.8, 14.2, 13.3;

HRMS (ESI+): exact mass calculated for $[M+H]^+$ ($C_{26}H_{33}N_2O_2S$) requires *m/z* 437.2263, found *m/z* 437.2261.

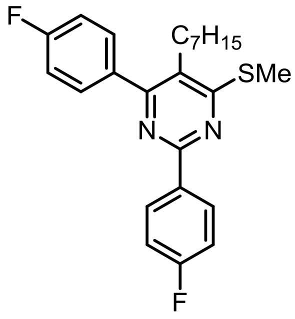

5at

2,4-bis(4-fluorophenyl)-5-heptyl-6-(methylthio)pyrimidine (5at)

IR (neat) ν_{max}: 2927, 2856, 1603, 1504, 1390, 1226, 878;

^{1}H-NMR (400 MHz, $CDCl_3$): δ 8.52-8.48 (m, 2H), 7.56-7.52 (m, 2H), 7.20-7.10 (m, 4H), 2.72 (s, 3H), 2.67 (t, *J* = 8.2 Hz, 2H), 1.59-1.51 (m, 2H), 1.29-1.21 (br, 8H), 0.87 (t, *J* = 7.1 Hz, 3H);

^{13}C-NMR (100 MHz, $CDCl_3$): δ 170.3, 165.2 (d, $J_{F\text{-}C}$ = 144.1 Hz), 162.7 (d, $J_{F\text{-}C}$ = 142.4 Hz), 162.0, 159.6, 135.2 (d, $J_{F\text{-}C}$ = 3.7 Hz), 134.1 (d, $J_{F\text{-}C}$ = 3.0 Hz), 130.8 (d, $J_{F\text{-}C}$ = 8.1 Hz, 2C), 130.3 (d, $J_{F\text{-}C}$ = 8.1 Hz, 2C), 128.0, 115.5 ($J_{F\text{-}C}$ = 3.6 Hz, 2C), 115.3 ($J_{F\text{-}C}$ = 3.6 Hz, 2C), 31.8, 29.8, 28.8, 28.6, 28.5, 22.7, 14.2, 13.4;

HRMS (ESI+): exact mass calculated for $[M+H]^+$ ($C_{24}H_{27}F_2N_2S$) requires *m/z* 413.1863, found *m/z* 413.1860.

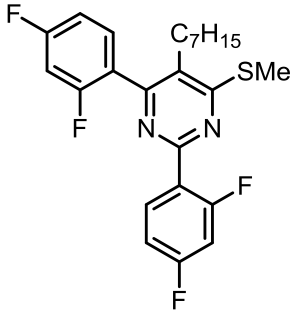

5au

2,4-bis(2,4-difluorophenyl)-5-heptyl-6-(methylthio)pyrimidine (5au)

IR (neat) ν_{max}: 2927, 2856, 1600, 1502, 1389, 1267, 1142, 968, 850;

^{1}H-NMR (400 MHz, $CDCl_3$): δ 8.17-8.11 (m, 1H), 7.42-7.36 (m, 1H), 7.01 (td, *J* = 8.3, 2.3 Hz, 1H), 6.97-6.88 (m, 3H), 2.67 (s, 3H), 2.56 (t, *J* = 8.2 Hz, 2H), 1.49-1.42 (m, 2H), 1.26-1.15 (br, 8H), 0.85 (t, *J* = 7.2 Hz, 3H);

^{13}C-NMR (100 MHz, $CDCl_3$): δ 170.4, 165.0 (dd, $J_{F\text{-}C}$ = 57.2, 12 Hz), 162.8 (td, $J_{F\text{-}C}$ = 32.9, 11.8 Hz), 160.8 (dd, $J_{F\text{-}C}$ = 27.9, 12.5 Hz), 158.9 (d, $J_{F\text{-}C}$ = 2.6 Hz), 158.5 (d, $J_{F\text{-}C}$ = 12.5 Hz), 157.7, 133.2 (dd, $J_{F\text{-}C}$ = 10.3, 3.0 Hz), 132.1 (dd, $J_{F\text{-}C}$ = 9.6, 5.1 Hz), 129.9, 123.0 (dd, $J_{F\text{-}C}$ = 5.8, 3.7 Hz), 122.9 (m), 111.9 (dd, $J_{F\text{-}C}$ = 21.3, 3.7 Hz), 111.4 (dd, $J_{F\text{-}C}$ = 21.3, 4.4 Hz), 105.1 (t, $J_{F\text{-}C}$ = 25.6 Hz), 104.4 (t, $J_{F\text{-}C}$ = 25.7 Hz), 31.7, 29.7, 28.8, 28.6, 27.9, 22.7, 14.2, 13.3;

HRMS (ESI+): exact mass calculated for $[M+H]^+$ ($C_{24}H_{25}F_4N_2S$) requires *m/z* 449.1675, found *m/z* 449.1667.

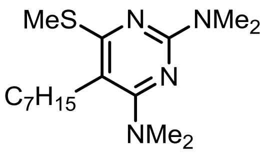

5av

2,4-di(dimethylamino)-5-heptyl-6-(methylthio)pyrimidine (5av)

IR (neat) ν_{max}: 2922, 2851, 1550, 1517, 1377, 1136, 970, 795;

^{1}H-NMR (400 MHz, $CDCl_3$): δ 3.13 (s, 6H), 2.92 (s, 6H), 2.53-2.49 (m+s, 5H), 1.55 (br, 2H), 1.33-1.29 (br, 8H), 0.90-0.87 (br, 3H);

^{13}C-NMR (100 MHz, $CDCl_3$): δ 167.7, 165.3, 159.0, 106.6, 41.5 (2C), 36.8 (2C), 32.1, 30.1, 29.2, 28.8, 28.1, 22.8, 14.3, 13.3;

HRMS (ESI+): exact mass calculated for $[M+H]^+$ ($C_{16}H_{31}N_4S$) requires *m/z* 311.2269, found *m/z* 311.2261.

General procedure for the synthesis of pyrimidines with acetonitrile

Under Argon, TfOH (0.20 mmol, 1.0 eq.) was slowly added to a cold (0 ˚C) solution of thio-alkyne (0.2 mmol, 1.0 eq) in acetonitrile (2 mL). The result mixture was warm up to rt and stirred for 16 h at ambient temperature. The reaction was quenched with $NaHCO_3$ sat. sol. (5 mL) after cooled to rt, extracted with DCM (3 x 5 mL), dried over Na_2SO_4. 4-Dimethylaminopyridine (12.2 mg, 0.1 mmol) was

added before the filtration. Then the solvent was removed in vacuo. After obtain the crude proton nmr, the crude product was purified by column chromatography on silica gel with hexane/ethyl acetate (5:1).

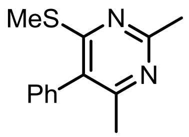

5ba

2,4-dimethyl-6-(methylthio)-5-phenylpyrimidine (5ba)

IR (neat) ν_{max}: 2925, 2851, 1528, 1404, 1360, 1230, 1006, 859, 659;

^{1}H-NMR (400 MHz, $CDCl_3$): δ 7.48-7.40 (m, 3H), 7.22-7.20 (m, 2H), 2.68 (s, 3H), 2.45 (s, 3H), 2.18 (s, 3H);

^{13}C-NMR (100 MHz, $CDCl_3$): δ 168.8, 165.6, 161.4, 135.5, 129.6 (2C), 129.1 (2C), 129.0, 128.5, 26.1, 22.4, 13.2;

HRMS (ESI+): exact mass calculated for $[M+H]^+$ ($C_{13}H_{15}N_2S$) requires *m/z* 231.0956, found *m/z* 231.0945.

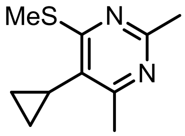

5ca

5-cyclopropyl-2,4-dimethyl-6-(methylthio)pyrimidine (5ca)

IR (neat) ν_{max}: 2926, 2854, 1530, 1418, 1276, 859;

^{1}H-NMR (400 MHz, $CDCl_3$): δ 2.58 (s, 3H), 2.52 (s, 3H), 2.51 (s, 3H), 1.60-1.54 (m, 1H), 1.09-1.04 (m, 2H), 0.63-0.59 (m, 2H);

^{13}C-NMR (100 MHz, $CDCl_3$): δ 170.7, 164.2, 163.8, 126.7, 25.8, 22.4, 12.9, 9.3, 8.7 (2C);

HRMS (ESI+): exact mass calculated for $[M+H]^+$ ($C_{10}H_{15}N_2S$) requires *m/z* 195.0956, found *m/z* 195.0952.

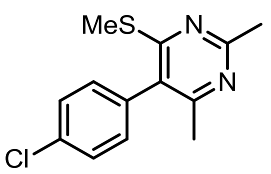

5da

5-(4-chlorophenyl)-2,4-dimethyl-6-(methylthio)pyrimidine (5da)

IR (neat) ν_{max}: 2968, 1531, 1408, 1090, 826;

^{1}H-NMR (400 MHz, $CDCl_3$): δ 7.44 (dt, *J* = 8.5, 2.0 Hz, 2H), 7.15 (dt, *J* = 8.5, 2.0 Hz, 2H), 2.68 (s, 3H), 2.46 (s, 3H), 2.17 (s, 3H);

^{13}C-NMR (100 MHz, $CDCl_3$): δ 168.9, 165.9, 161.4, 134.7, 133.8, 131.1 (2C), 129.4 (2C), 127.9, 26.1, 22.3, 13.2;

HRMS (ESI+): exact mass calculated for $[M+H]^+$ ($C_{13}H_{14}ClN_2S$) requires *m/z* 265.0566, found *m/z* 265.0562.

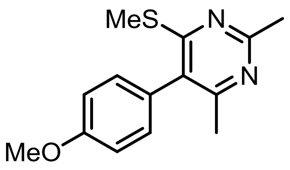

5ae

5-(4-methoxyphenyl)-2,4-dimethyl-6-(methylthio)pyrimidine (5ea)

IR (neat) ν_{max}: 2926, 2835, 1531, 1408, 1247, 830;

^{1}H-NMR (400 MHz, $CDCl_3$): δ 7.13 (dt, *J* = 8.8, 2.2 Hz, 2H), 6.99 (dt, *J* = 8.8, 2.2 Hz, 2H), 3.86 (s, 3H), 2.67 (s, 3H), 2.45 (s, 3H), 2.19 (s, 3H);

^{13}C-NMR (100 MHz, $CDCl_3$): δ 169.2, 165.4, 161.7, 159.8, 130.8 (2C), 128.7, 127.5, 114.5 (2C), 55.4, 26.1, 22.4, 13.3;

HRMS (ESI+): exact mass calculated for $[M+H]^+$ ($C_{14}H_{17}N_2OS$) requires *m/z* 261.1062, found *m/z* 261.1056.

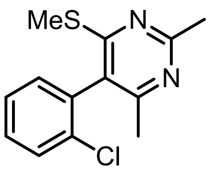

5fa

5-(2-chlorophenyl)-2,4-dimethyl-6-(methylthio)pyrimidine (5fa)

IR (neat) ν_{max}: 2927, 1534, 1431,1408, 870, 756;

^{1}H-NMR (400 MHz, $CDCl_3$): δ 7.54-7.52 (m, 1H), 7.41-7.35 (m, 2H), 7.21-7.19 (m, 1H), 2.70 (s, 3H), 2.48 (s, 3H), 2.15 (s, 3H);

^{13}C-NMR (100 MHz, $CDCl_3$): δ 168.8, 166.3, 162.0, 134.3, 134.2, 131.5, 130.3, 130.2, 127.5, 126.5, 26.2, 22.0, 13.0;

HRMS (ESI+): exact mass calculated for $[M+H]^+$ ($C_{13}H_{14}ClN_2S$) requires *m/z* 265.0566, found *m/z* 265.0563.

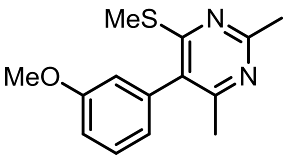

5ga

5-(3-methoxyphenyl)-2,4-dimethyl-6-(methylthio)pyrimidine (5ga)

IR (neat) ν_{max}: 2926, 2834, 1579, 1532, 1426, 1288, 995, 863;

^{1}H-NMR (400 MHz, $CDCl_3$): δ 7.38 (t, *J* = 7.9 Hz, 1H), 6.96 (ddd, *J* = 8.4, 2.7, 0.9 Hz, 1H), 6.80-6.75 (m, 2H), 3.83 (s, 3H), 2.67 (s, 3H), 2.45 (s, 3H), 2.20 (s, 3H);

^{13}C-NMR (100 MHz, $CDCl_3$): δ 168.7, 165.6, 161.3, 160.1, 136.8, 130.2, 128.9, 121.9, 115.3, 113.9, 55.4, 26.1, 22.4, 13.2;

HRMS (ESI+): exact mass calculated for $[M+H]^+$ ($C_{14}H_{17}N_2SO$) requires *m/z* 261.1062, found *m/z* 261.1056.

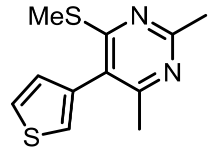

5ha

2,4-dimethyl-6-(methylthio)-5-(thiophen-3-yl)pyrimidine (5ha)

IR (neat) ν_{max}: 2925, 2854, 1522, 1420, 1346, 1274, 1182, 854, 787;

^{1}H-NMR (400 MHz, $CDCl_3$): δ 7.45 (dd, *J* = 4.9, 3.0 Hz, 1H), 7.24 (dd, *J* = 2.9, 1.3 Hz, 1H), 7.00 (dd, *J* = 4.9, 1.2 Hz, 1H), 2.66 (s, 3H), 2.46 (s, 3H), 2.23 (s, 3H);

^{13}C-NMR (100 MHz, $CDCl_3$): δ 169.5, 165.6, 162.1, 134.9, 128.5, 126.4, 125.2, 124.4, 26.1, 22.4, 13.2;

HRMS (ESI+): exact mass calculated for $[M+H]^+$ ($C_{11}H_{13}N_2S_2$) requires *m/z* 237.0520, found *m/z* 237.0511.

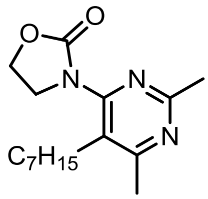

7la

3-(5-heptyl-2,6-dimethylpyrimidin-4-yl)oxazolidin-2-one (7la)

IR (neat) ν_{max}: 2924, 2855, 1765, 1568, 1398, 1213, 1037, 761;

^{1}H-NMR (400 MHz, $CDCl_3$): δ 4.49 (t, *J* = 8.1 Hz, 2H), 4.16 (t, *J* = 8.1 Hz, 2H), 2.67 (t, *J* = 8.0 Hz, 2H), 2.56 (s, 3H), 2.51 (s, 3H), 1.46-1.39 (m, 2H), 1.28-1.23 (br, 8H), 0.84 (t, *J* = 7.1 Hz, 3H);

^{13}C-NMR (100 MHz, $CDCl_3$): δ 168.6, 164.5, 156.1, 155.8, 125.7, 63.0, 45.9, 31.8, 29.7, 29.1, 29.0, 27.0, 25.3, 22.7, 22.4, 14.1;

HRMS (ESI+): exact mass calculated for $[M+Na]^+$ ($C_{16}H_{25}N_3NaO_2$) requires *m/z* 314.1844, found *m/z* 314.1835.

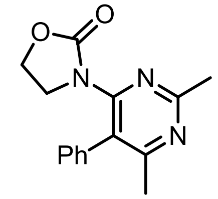

7aa

3-(2,6-dimethyl-5-phenylpyrimidin-4-yl)oxazolidin-2-one (7aa)

IR (neat) ν_{max}: 2919, 2853, 1775, 1569, 1410, 1262, 1082, 758;

^{1}H-NMR (400 MHz, $CDCl_3$): δ 7.45-7.37 (m, 3H), 7.27-7.24 (m, 2H, overlapping with solvent), 4.30 (t, *J* = 8.1 Hz, 2H), 3.94 (t, *J* = 7.4 Hz, 2H), 2.71 (s, 3H), 2.36 (s, 3H);

^{13}C-NMR (100 MHz, $CDCl_3$): δ 168.1, 166.5, 156.0, 154.8, 134.7, 129.5 (2C), 128.7 (2C), 128.2, 126.7, 62.7, 45.8, 25.8, 23.3;

HRMS (ESI+): exact mass calculated for $[M+Na]^+$ ($C_{15}H_{15}N_3NaO_2$) requires *m/z* 292.1062, found *m/z* 292.1056.

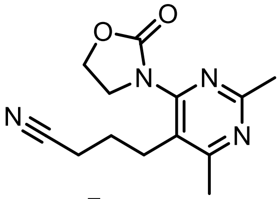

7ma

4-(2,4-dimethyl-6-(2-oxooxazolidin-3-yl)pyrimidin-5-yl)butanenitrile (7ma)

IR (neat) ν_{max}: 3398, 2926, 2246, 1760, 1569, 1401, 1222, 1035, 761;

^{1}H-NMR (400 MHz, $CDCl_3$): δ 4.53 (t, *J* = 8.1 Hz, 2H), 4.21 (t, *J* = 7.52 Hz, 2H), 2.84 (t, *J* = 8.0 Hz, 2H), 2.58 (s, 3H), 2.54 (s, 3H), 2.35 (t, *J* = 6.8 Hz, 2H), 1.96-1.89 (m, 2H);

^{13}C-NMR (100 MHz, $CDCl_3$): δ 168.6, 165.3, 156.5, 156.1, 122.8, 119.3, 63.2, 45.9, 26.3, 25.4, 24.3, 22.5, 17.3;

HRMS (ESI+): exact mass calculated for $[M+Na]^+$ ($C_{13}H_{16}N_4NaO_2$) requires *m/z* 283.1171, found *m/z* 283.1166.

Further manipulation of isoquinoline and pyrimidine products.

Compound **3di** (212 mg, 0.5 mmol) was dissolved in AcOH (10 mL). The solution was submitted to a pre-set H-Cube reactor cyclically (H_2 100 bar, 40 ˚C) and was analyzed by TLC. After completely consuming of the starting material (1 h), the reactor was rinsed in succession by AcOH (10 mL) and Methonal (10 mL). Combined solution was evaporated under vacuum. The crude product was protected by TsCl in DCM, with triethylamine as the base. After filtration, the residue was purified by flash column chromatography (Hexane/ethyl acetate = 1:1) on silica gel to afford compound *rac*-**8-Ts** (246 mg, 99%).

Compound **3hi** (117 mg, 0.25 mmol) was dissolved in AcOH (10 mL). The solution was submitted to a pre-set H-Cube reactor cyclically (H_2 100 bar, 40 ˚C) and was analyzed by TLC. After completely consuming of the starting material (1 h), the reactor was rinsed in succession by AcOH (10 mL) and Methonal (10 mL). Combined solution was evaporated under vacuum. The crude product was protected by TsCl in DCM, with triethylamine as the base. After filtration, the residue was purified by flash column chromatography (Hexane/ethyl acetate = 1:1) on silica gel to afford compound (*S*)-**8-Ts** (74 mg, 60%).

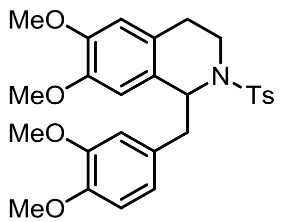

8-Ts

1-(3,4-dimethoxybenzyl)-6,7-dimethoxy-2-tosyl-1,2,3,4-tetrahydroisoquinoline (**8**)

IR (neat) ν_{max}: 3622, 2935, 2834, 1513, 1462, 1317, 1229, 1153, 1091, 1025, 973, 810, 72.9;

^{1}H-NMR (400 MHz, $CDCl_3$): δ 7.53 (d, *J* = 8.3 Hz, 2H), 7.14 (d, *J* = 18.0 Hz, 2H), 6.72 (d, *J* = 1 Hz, 1H), 6.56 (dd, *J* = 8.0, 1.9 Hz, 1H), 6.51 (d, *J* = 1.9 Hz, 1H), 6.44 (s, 1H), 6.20 (s, 1H), 5.09 (t, *J* = 6.5 Hz, 1H), 3.85 (s, 3H), 3.80 (s, 3H), 3.75 (s, 3H), 3.67 (s, 3H), 3.62 (ddd, *J* = 13.4, 5.9, 3.8 Hz, 1H), 3.34 (ddd, *J* = 14.9, 10.3, 4.7 Hz, 1H), 3.13 (dd, *J* = 13.5, 5.8 Hz, 1H), 3.02 (dd, *J* = 13.5, 7.1 Hz, 1H), 2.60 (ddd, *J* = 16.3, 10.3, 6.1 Hz, 1H), 2.39 (dt, *J* = 16.2, 4.3 Hz, 1H), 2.35 (s, 3H);

^{13}C-NMR (100 MHz, $CDCl_3$): δ 148.8, 148.0, 147.9, 147.0, 143.2, 137.5, 130.3, 129.5 (2C), 127.6, 127.2 (2C), 125.7, 122.3, 113.2, 111.4, 111.1, 110.5, 57.7, 56.0, 56.0, 55.9, 55.9, 44.1, 40.0, 27.1, 21.6;

HRMS (ESI+): exact mass calculated for $[M+Na]^+$ (C27H31NNaO6S) requires *m/z* 520.1770, found *m/z* 520.1767.

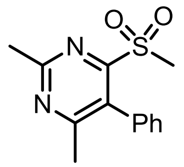

9

2,4-dimethyl-6-(methylsulfonyl)-5-phenylpyrimidine (9)

To a solution of **5ba** (23 mg, 0.1 mmol) in DCM (3 mL) was slowly added 3-chloroperoxyvenzoic acid (70% purity) (51.8 mg, 0.21 mmol). The resulting mixture was stirred at room temperature for 5 h before quenching with 5 mL $Na_2S_2O_3$ (5%) solution. The layers were shaken and separated. The aqueous phase was extracted with DCM (2X5 mL). The combined organic layers were washed with saturated $NaHCO_3$ solution (5 mL) and dried over $MgSO_4$. The solvent was removed under vacuum. The crude product was purified by flash column chromatography (Hexane/ethyl acetate = 1:1) on silica gel to afford the title compound (25.7 mg, 98%) as white solid.

IR (neat) ν_{max}: 2929, 2363, 2339, 1561, 1412, 1310, 1130, 748;

^{1}H-NMR (400 MHz, $CDCl_3$): δ 7.50-7.45 (m, 3H), 7.31-7.28 (m, 2H), 3.26 (s, 3H), 2.80 (s, 3H), 2.35 (s, 3H);

^{13}C-NMR (100 MHz, $CDCl_3$): δ 170.5, 166.3, 162.5, 132.1, 129.7 (2C), 129.0, 128.6 (2C), 127.9, 40.2, 25.9, 23.4;

HRMS (ESI+): exact mass calculated for $[M+Na]^+$ ($C_{13}H_{14}N_2SNaO_2$) requires *m/z* 285.0674, found *m/z* 285.0662.

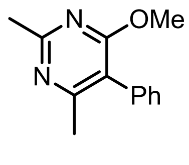

10

4-methoxy-2,6-dimethyl-5-phenylpyrimidine (10)

To a solution of sodium methoxide (21.6 mg, 0.4 mmol) in methanol (2 mL) was added **9** (26.2 mg, 0.1 mmol). The resulting mixture was heated to reflux for 24 h. The solvent was removed under vacuum. The residue was diluted with water (5 mL) and then extracted with DCM (3X5 mL). The combined organic layers were dried over $MgSO_4$. The solvent was removed under vacuum. The crude product was

purified by flash column chromatography (Hexane/ethyl acetate = 1:1) on silica gel to afford the title compound (18.6 mg, 87%) as white solid.

IR (neat) ν_{max}: 2952, 2926, 1574, 1551, 1418, 1389, 1096, 701;

^{1}H-NMR (400 MHz, $CDCl_3$): δ 7.45-7.35 (m, 3H), 7.24-7.22 (m, 2H), 3.89 (s, 3H), 2.63 (s, 3H), 2.26 (s, 3H);

^{13}C-NMR (100 MHz, $CDCl_3$): δ 166.9, 165.8, 164.4, 134.2, 130.1 (2C), 128.5 (2C), 127.8, 118.0, 54.0, 26.0, 22.4;

HRMS (ESI+): exact mass calculated for $[M+H]^+$ ($C_{13}H_{15}N_2O$) requires *m/z* 215.1184, found *m/z* 215.1178.

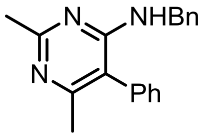

11

N-benzyl-2,6-dimethyl-5-phenylpyrimidin-4-amine (11)

Compound **9** (26.2 mg, 0.1 mmol) was mixed with benzyl amine (0.44 mL, 4 mmol) under Argon. The mixture was stirred at 110 ˚C for 12 h. The result mixture was transferred to a column with silica gel (Hexane/ethyl acetate = 5:7) to afford the title compound (27.8 mg, 96%) as white solid.

IR (neat) ν_{max}: 3432, 2921, 2852, 1564, 1498, 1431, 1139, 699;

^{1}H-NMR (400 MHz, $CDCl_3$): δ 7.45 (t, *J* = 7.6 Hz, 2H), 7.37 (tt, *J* = 7.4, 1.3 Hz, 1H), 7.30-7.27 (m, 2H), 7.23-7.21 (m, 5H), 4.69 (br, 1H), 4.64 (d, *J* = 5.6 Hz, 2H), 2.55 (s, 3H), 2.11 (s, 3H);

^{13}C-NMR (100 MHz, $CDCl_3$): δ 166.0, 161.0, 160.3, 139.4, 134.8, 130.1 (2C), 129.7 (2C), 128.6 (2C), 128.3, 127.5 (2C), 127.2, 114.8, 44.7, 26.3, 22.3;

HRMS (ESI+): exact mass calculated for $[M+H]^+$ ($C_{19}H_{20}N_3$) requires *m/z* 290.1657, found *m/z* 290.1653.

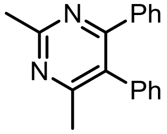

12

2,4-dimethyl-5,6-diphenylpyrimidine (12)

To a solution of compound **9** (26.2 mg, 0.1 mmol) in THF (2 mL) was added 0.1 mL (0.2 mmol) phenylmagnesium chloride solution (2 mol/L in THF). The resulting mixture was refluxed for 24 h before quenching with saturated NH_4Cl (5 mL) at rt. The mixture was extracted with MTBE (3X10 mL). The combined organic layers were dried over $MgSO_4$. The solvent was removed under vacuum. The crude product was purified by flash column chromatography (Hexane/ethyl acetate = 1:1) on silica gel to afford the title compound (18.5 mg, 71%) as white solid.

IR (neat) ν_{max}: 2923, 2853, 1533, 1407, 1177, 993, 755;

^{1}H-NMR (400 MHz, $CDCl_3$): δ 7.31-7.28 (m, 5H), 7.23-7.16 (m, 3H), 7.10-7.08 (m, 2H), 2.80 (s, 3H), 2.37 (s, 3H);

^{13}C-NMR (100 MHz, $CDCl_3$): δ 166.3, 166.0, 163.9, 138.5, 136.9, 130.2 (2C), 129.8 (2C), 129.6 (2C), 128.7 (2C), 128.0 (2C), 127.7, 26.2, 23.6;

HRMS (ESI+): exact mass calculated for $[M+H]^+$ ($C_{18}H_{17}N_2$) requires *m/z* 261.1392, found *m/z* 261.1380.

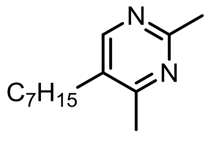

13

5-heptyl-2,4-dimethylpyrimidine (13)

To a solution of **5aa** (23.8 mg, 0.1 mmol) in acetone (2 mL) was added an excess of Raney 2800 Ni (washed with acetone three times *just prior to use*; Caution! Raney Ni is pyrophoric if allowed to dry out). The resulting mixture was stirred vigorously at room temperature for 6 h under an atmosphere of H_2 (balloon). The mixture was diluted with ethyl acetate and filtrated. The Raney Ni was washed with ethyl acetate. The combined solution was concentrated and purified by flash column chromatography (Hexane/ethyl acetate = 5:1) on silica gel to afford the title compound (17.9 mg, 87%) as colorless oil.

IR (neat) ν_{max}: 2925, 2855, 1582, 1552, 1437, 1030, 936;

^{1}H-NMR (400 MHz, $CDCl_3$): δ 8.28 (s, 1H), 2.65 (s, 3H), 2.54 (t, *J* = 7.9 Hz, 2H), 2.46 (s, 3H), 1.59-1.51 (m, 2H), 1.34-1.27 (br, 8H), 0.88 (t, *J* = 7.0 Hz, 3H);

^{13}C-NMR (100 MHz, $CDCl_3$): δ 165.3, 165.1, 156.5, 130.1, 31.9, 29.8, 29.7, 29.5, 29.2, 25.7, 22.8, 21.7, 14.2;

HRMS (ESI+): exact mass calculated for $[M+H]^+$ ($C_{13}H_{23}N_2$) requires *m/z* 207.1861, found *m/z* 207.1851

Kinetic isotope effect.

Under Argon atmosphere, mixture of **1a** (0.22 mmol, 1.1 eq.) and CH_3CN (0.1 mmol, 1.0 eq.) and $CH_3{}^{13}CN$ (0.1 mmol, 1.0 eq.) in dichloroethane (4 mL) was added dropwise of TfOH (0.22 mmol, 1.1 eq.) at 0 °C. The reaction was warm up immediately to RT for 30 min. Then the reaction refluxed at 115 °C for 16 h. The reaction was quenched with Na_2CO_3 sat. sol. (5 mL) after cooled to rt, extracted with DCM (3 x 5 mL), dried over Na_2SO_4 and the solvent was removed in vacuo. The crude product was purified by column chromatography on silica gel with hexane/ethyl acetate (7/3). The result was shown in Supplementary Figure 61.

Crystal data of compound 5ae.

The X-ray intensity data were measured on a Bruker D8-Venture equipped with multilayer monochromators, Mo K/a INCOATEC micro focus sealed tube and Kryoflex II cooling device. The structure was solved by direct methods and refined by full-matrix least-squares techniques. Non-hydrogen atoms were refined with anisotropic displacement parameters. Hydrogen atoms were inserted at calculated positions and refined with a riding model respectively as rotating systems. The following software were used: Frame integration, *Bruker SAINT software package*[i] using a narrow-frame algorithm, Absorption correction, *SADABS*[ii] , structure solution, *SHELXS-97*[iii], refinement, *SHELXL-2013*[iv], *OLEX2*[v], *SHELXLE*[vi], molecular diagram, *OLEX2*[vii]. Experimental data and CCDC-Code can be found in Supplementary Table 3. Crystal data, data collection parameters, and structure refinement details are given in Supplementary Tables 4 and 5. Molecular Structure in "Ortep View" is displayed in Supplementary Figure 64.

Computational details.

DFT calculations were carried out at with the B3LYP functional[viii] and the 6-31G* basis set,[ix] as implemented in the Gaussian 09 suite.[x] The nature of the optimized structures, intermediates and transition states, was assessed through frequency calculations. Reported energies are enthalpic values at room temperature. 3D structures were generated using the Chemcraft software.[xi] Result was shown in Supplementary Figures 65.

Cartesian coordinates and energies for all the structures appearing in Supplementary Figures 66 and 67.

Acetonitrile

E = −132.7549284
H = −132.704747
G = −132.733302

xyz

7	0.000245000	1.440782000	0.000000000
6	0.000000000	0.280520000	0.000000000
6	-0.000174000	-1.180918000	0.000000000
1	1.026202000	-1.561139000	0.000000000
1	-0.513435000	-1.560972000	0.888906000
1	-0.513435000	-1.560972000	-0.888906000

TfOH

E = −961.9991175
H = −961.951767
G = −961.993766

xyz

16	0.852895000	-0.148471000	0.075889000
6	-1.008549000	0.009452000	-0.001823000
9	-1.543022000	-0.924677000	0.779534000
9	-1.361344000	1.219398000	0.436727000
9	-1.424560000	-0.153419000	-1.252832000
8	1.260898000	0.173359000	1.435985000
8	1.215893000	-1.372425000	-0.605998000
8	1.256911000	1.097896000	-0.897986000

1	1.495691000	1.856471000	-0.330144000

Starting oxazolidinone derivative

E = −629.7321335
H = −629.540830
G = −629.594141

xyz

6	1.973118000	-0.082504000	0.016293000
6	2.635412000	1.161405000	0.016604000
6	4.026489000	1.215518000	0.002568000
6	4.781191000	0.039934000	-0.013534000
6	4.132341000	-1.196977000	-0.014694000
6	2.741596000	-1.263027000	0.000905000
6	0.548630000	-0.139514000	0.031465000
6	-0.663994000	-0.159848000	0.039859000
7	-2.007044000	-0.230331000	0.066543000
6	-2.841800000	0.895613000	-0.014052000
8	-4.131685000	0.453500000	-0.080545000
6	-4.187625000	-0.966839000	0.134382000
6	-2.759040000	-1.469103000	-0.134266000
8	-2.513561000	2.049727000	-0.028733000
1	2.047319000	2.073783000	0.027941000
1	4.523899000	2.181743000	0.003493000
1	5.866556000	0.087500000	-0.025106000
1	4.712068000	-2.116181000	-0.027138000
1	2.237868000	-2.225048000	0.001037000
1	-4.929797000	-1.387065000	-0.546521000
1	-4.496921000	-1.151120000	1.168909000
1	-2.625403000	-1.842404000	-1.157420000
1	-2.442228000	-2.242652000	0.570054000

Starting methylthio derivative

E = −745.9020589
H = −745.751504
G = −745.800432

xyz

6	0.999151000	-0.085889000	-0.009351000
6	1.835737000	-1.219207000	0.040166000
6	3.219988000	-1.071288000	0.050331000
6	3.796181000	0.200878000	0.014875000

6	2.974790000	1.329854000	-0.032682000
6	1.589374000	1.193510000	-0.046538000
6	-0.418232000	-0.232894000	-0.021905000
6	-1.630251000	-0.354912000	-0.031057000
16	-3.299247000	-0.620804000	-0.055413000
1	1.387591000	-2.207573000	0.069755000
1	3.852252000	-1.954433000	0.087531000
1	4.876947000	0.311591000	0.024170000
1	3.415331000	2.322961000	-0.060819000
1	0.950832000	2.070773000	-0.085852000
6	-3.940175000	1.095192000	0.108507000
1	-3.608001000	1.708574000	-0.731266000
1	-3.616107000	1.535488000	1.053478000
1	-5.030278000	1.014015000	0.095525000

A) Isoquinoline route

A.1) Oxazolidinone derivative pathway

Starting oxazolidinone derivative + acetonitrile + TfOH system (pre-association complex in Scheme X1) (**I**$_i$)

E = −1724.5233642
H = −1724.230116
G = −1724.323719

xyz

6	3.400951000	-1.188828000	0.045578000
6	4.528559000	-1.939621000	0.366239000
6	5.766091000	-1.316305000	0.545553000
6	5.872314000	0.068571000	0.397779000
6	4.753040000	0.828704000	0.068473000
6	3.501915000	0.208120000	-0.110026000
6	2.344881000	0.972725000	-0.443334000
6	1.336602000	1.585208000	-0.720322000
7	-1.329736000	3.443051000	2.277960000
6	-1.637903000	2.492050000	2.867740000
6	-2.027413000	1.291296000	3.600653000
8	-2.137536000	-0.417888000	0.776928000
16	-2.574801000	-1.584594000	0.009021000
8	-3.694625000	-2.401253000	0.437241000
6	-1.085296000	-2.706105000	-0.100799000
9	-0.082246000	-2.065900000	-0.710233000
8	-2.781562000	-1.206642000	-1.519804000
9	-0.708517000	-3.037650000	1.136110000

9	-1.386525000	-3.807524000	-0.783528000
1	2.435788000	-1.666707000	-0.088838000
1	4.440031000	-3.016647000	0.480286000
1	6.642485000	-1.906458000	0.798705000
1	6.832150000	0.559100000	0.535426000
1	4.835200000	1.904569000	-0.052843000
1	-1.247764000	1.019571000	4.319211000
1	-2.961342000	1.467565000	4.143482000
1	-2.170843000	0.468011000	2.893868000
1	-2.155750000	-0.450716000	-1.785727000
7	0.214536000	2.270439000	-1.012701000
8	-0.952176000	0.560923000	-2.051863000
6	-0.877486000	1.707043000	-1.633382000
6	0.006794000	3.698901000	-0.734409000
1	0.223087000	3.922269000	0.311535000
1	0.639308000	4.306930000	-1.390293000
8	-1.859344000	2.613595000	-1.741303000
6	-1.497471000	3.832876000	-1.039662000
1	-2.087162000	3.882070000	-0.122943000
1	-1.737895000	4.671483000	-1.694237000

Protonation TS (**II**$_{\mathrm{i}}$)

E = −1724.5053961
H = −1724.217996
G = −1724.308264
ῡ = −957.2 cm^{-1}

xyz

6	4.274711000	1.028340000	0.310182000
6	5.588749000	0.685505000	0.610234000
6	5.900747000	-0.618408000	1.007581000
6	4.892104000	-1.578423000	1.100344000
6	3.573612000	-1.242705000	0.797706000
6	3.250872000	0.066158000	0.401462000
6	1.869904000	0.410583000	0.092848000
6	1.142222000	1.389920000	-0.215005000
7	-3.279744000	2.251239000	2.358786000
6	-3.625555000	1.175970000	2.628134000
6	-4.054563000	-0.178092000	2.966004000
8	0.337702000	-1.688050000	0.319514000
16	-1.197895000	-1.505413000	0.215848000
8	-1.903572000	-2.207754000	1.287789000
6	-1.572107000	-2.444559000	-1.349068000
9	-0.908061000	-1.902958000	-2.372369000
8	-1.552463000	-0.104396000	-0.060524000

9	-1.215718000	-3.722759000	-1.217262000
9	-2.884477000	-2.374373000	-1.590309000
1	4.029974000	2.039123000	-0.003059000
1	6.371585000	1.434969000	0.533873000
1	6.928010000	-0.883533000	1.241994000
1	5.130636000	-2.592732000	1.407245000
1	2.789057000	-1.989334000	0.869049000
1	-5.079961000	-0.343615000	2.620359000
1	-3.394447000	-0.909212000	2.485849000
1	-4.022516000	-0.322657000	4.050615000
1	0.963198000	-0.664974000	0.163520000
7	0.213427000	2.250240000	-0.489445000
8	0.395470000	2.269493000	-2.835651000
6	-0.203559000	2.558829000	-1.843074000
6	-0.689981000	2.896862000	0.486804000
1	-0.945163000	2.214458000	1.293752000
1	-0.214934000	3.799902000	0.882008000
8	-1.349758000	3.245283000	-1.759285000
6	-1.893303000	3.201405000	-0.411293000
1	-2.635617000	2.404056000	-0.366786000
1	-2.357121000	4.165689000	-0.209813000

Protonated intermediate (**III**$_{i}$)

E = −1724.5089272
H = −1724.216342
G = −1724.306820

xyz

6	-3.552587000	0.674244000	-0.096011000
6	-4.871635000	0.236371000	-0.044475000
6	-5.234005000	-0.977388000	-0.638950000
6	-4.271887000	-1.755510000	-1.285711000
6	-2.948238000	-1.322941000	-1.344049000
6	-2.580881000	-0.103625000	-0.749829000
6	-1.174870000	0.294473000	-0.838132000
6	-0.530410000	1.292278000	-0.323052000
7	-1.346509000	0.631469000	2.839747000
6	-1.406214000	-0.526559000	2.784066000
6	-1.471253000	-1.982258000	2.688106000
8	0.760343000	-1.710077000	-1.613582000
16	2.074705000	-1.115066000	-1.225882000
8	3.225539000	-1.491280000	-2.046336000
6	2.404654000	-1.895076000	0.434404000
9	1.476498000	-1.468133000	1.342181000
8	1.957457000	0.330584000	-0.868438000

9	2.333059000	-3.227578000	0.385739000
9	3.605780000	-1.542294000	0.906995000
1	-3.271460000	1.614223000	0.369839000
1	-5.620066000	0.841626000	0.458707000
1	-6.266425000	-1.313244000	-0.598203000
1	-4.551207000	-2.697578000	-1.748410000
1	-2.189596000	-1.921245000	-1.840576000
1	-0.552974000	-2.356165000	2.226538000
1	-1.581441000	-2.425516000	3.682489000
1	-2.324344000	-2.277534000	2.069535000
1	-0.476662000	-0.381301000	-1.388484000
7	0.175591000	2.217637000	0.172348000
6	0.483691000	3.471942000	-0.559055000
8	-0.178477000	3.917378000	-1.441836000
8	1.588667000	3.969468000	-0.012659000
6	2.212246000	3.017082000	0.898011000
1	2.960285000	2.455713000	0.334992000
1	2.667274000	3.594580000	1.701825000
6	1.066626000	2.112695000	1.355830000
1	0.527601000	2.486804000	2.228360000
1	1.362477000	1.076075000	1.494486000

Acetonitrile addition TS (**IV**$_i$)

E = −1724.5007738
H = −1724.208782
G = −1724.293165
ῡ = −184.8 cm^{-1}

xyz

6	-1.375503000	3.186667000	-0.785623000
6	-0.426906000	2.286108000	-1.577761000
7	0.786250000	2.359896000	-0.720939000
6	0.715375000	3.555220000	0.126331000
8	-0.498016000	4.079081000	-0.030368000
6	1.667145000	1.439902000	-0.551968000
7	1.197640000	0.682068000	1.412301000
6	0.460234000	0.435587000	2.271084000
6	-0.448602000	0.099093000	3.355685000
8	1.609693000	3.959825000	0.805902000
6	2.626108000	0.667339000	-1.012783000
6	3.110189000	-0.655179000	-0.615976000
6	2.238193000	-1.657798000	-0.155983000
6	2.748949000	-2.910667000	0.176717000
6	4.114334000	-3.176274000	0.052687000
6	4.980342000	-2.188039000	-0.424532000

6	4.479577000	-0.937001000	-0.770494000
8	-2.151494000	0.466907000	0.686409000
16	-1.778405000	-0.837123000	0.068978000
6	-3.408442000	-1.558117000	-0.464222000
9	-3.228708000	-2.744551000	-1.058912000
8	-1.016017000	-0.698565000	-1.203002000
8	-1.226844000	-1.834875000	1.012304000
9	-4.214679000	-1.728741000	0.593320000
9	-4.019376000	-0.735703000	-1.333769000
1	1.171899000	-1.474496000	-0.094949000
1	2.067698000	-3.681821000	0.523687000
1	4.503417000	-4.156813000	0.313441000
1	6.041178000	-2.394798000	-0.532850000
1	5.149719000	-0.167485000	-1.145953000
1	0.046768000	0.225329000	4.322844000
1	-1.331193000	0.740732000	3.289546000
1	-0.774393000	-0.934748000	3.208363000
1	3.146354000	1.155948000	-1.841759000
1	-0.769209000	1.249491000	-1.654946000
1	-0.170439000	2.700459000	-2.558173000
1	-2.008275000	3.815525000	-1.411251000
1	-1.965944000	2.603835000	-0.075870000

Acetonitrile addition intermediate (**V**$_i$)

E = −1724.5571694
H = −1724.261740
G = −1724.342922

xyz

6	1.231160000	-2.944949000	-0.616700000
7	2.091733000	-1.892518000	-0.064181000
6	3.384799000	-2.366919000	0.121449000
8	3.385395000	-3.719230000	-0.074084000
6	2.036244000	-4.189939000	-0.230726000
6	1.710346000	-0.527377000	-0.076121000
7	0.305385000	-0.415012000	-0.041665000
6	-0.304850000	0.135772000	0.916798000
6	0.181819000	0.777248000	2.178810000
8	4.380792000	-1.748681000	0.413345000
6	2.582882000	0.499744000	-0.180689000
6	2.285872000	1.939453000	-0.245745000
6	3.236200000	2.837959000	0.277310000
6	3.035141000	4.215690000	0.223364000
6	1.881321000	4.733880000	-0.367725000
6	0.939315000	3.858152000	-0.912927000

6	1.137012000	2.479190000	-0.858332000
8	-1.695974000	0.216843000	0.852942000
16	-2.479894000	-0.453019000	-0.467919000
8	-2.369310000	-1.902886000	-0.447716000
8	-2.258639000	0.348129000	-1.658782000
6	-4.171813000	-0.009944000	0.211374000
9	-5.069230000	-0.430738000	-0.677495000
9	-4.367705000	-0.620934000	1.375252000
9	-4.269981000	1.305837000	0.364853000
1	4.140182000	2.441570000	0.733365000
1	3.783504000	4.885181000	0.639574000
1	1.723545000	5.807954000	-0.415019000
1	0.048903000	4.249807000	-1.398133000
1	0.408918000	1.819557000	-1.319687000
1	1.702250000	-4.615368000	0.722647000
1	2.029334000	-4.967568000	-0.996380000
1	0.231611000	-2.929637000	-0.181269000
1	1.133032000	-2.828592000	-1.703950000
1	3.631311000	0.226520000	-0.203087000
1	0.105544000	1.866379000	2.085429000
1	1.227310000	0.516712000	2.350218000
1	-0.435506000	0.459838000	3.023938000

Friedel-Crafts-like TS (**VI**$_i$)

E = −1724.5061238
H = −1724.213000
G = −1724.290197
ῡ = −475.6 cm^{-1}

xyz

6	1.472923000	-0.525292000	0.141830000
7	0.750238000	-0.080712000	1.193627000
6	-0.074438000	0.937447000	1.065468000
6	-0.578557000	1.650236000	2.290750000
6	2.110333000	0.334626000	-0.765040000
6	2.010159000	1.737189000	-0.642710000
6	3.179853000	2.539843000	-0.788287000
6	3.193327000	3.845811000	-0.358937000
6	2.028215000	4.449256000	0.194181000
6	0.851154000	3.747911000	0.228328000
6	0.789013000	2.381537000	-0.211932000
8	-1.190452000	0.736828000	0.076120000
16	-2.136766000	-0.578028000	0.266718000
8	-2.648056000	-0.676709000	1.628898000
8	-1.571106000	-1.743152000	-0.403275000

6	-3.512343000	0.075119000	-0.825055000
9	-4.444261000	-0.873801000	-0.899858000
9	-4.035290000	1.175112000	-0.288098000
9	-3.044790000	0.344384000	-2.040345000
1	4.086555000	2.075388000	-1.167047000
1	4.111772000	4.423425000	-0.423565000
1	2.067923000	5.481296000	0.531289000
1	-0.067751000	4.237074000	0.542117000
1	-0.096936000	2.133804000	-0.784428000
1	2.952225000	-0.081123000	-1.304052000
1	-1.224156000	2.487563000	2.018356000
1	0.274274000	2.024593000	2.862298000
1	-1.153853000	0.955622000	2.911692000
7	1.806167000	-1.877083000	0.219436000
1	0.155307000	-2.834873000	1.109976000
6	1.237388000	-2.750920000	1.248817000
1	1.436863000	-2.363282000	2.248724000
6	1.981998000	-4.062892000	0.960141000
1	1.336136000	-4.942015000	0.992796000
1	2.833022000	-4.213589000	1.633125000
8	2.485320000	-3.924399000	-0.379837000
6	2.471430000	-2.619062000	-0.760735000
8	2.966591000	-2.228439000	-1.788957000

Friedel-Crafts-like intermediate (**VII**$_{\mathbf{i}}$)

E = −1724.5467269
H = −1724.251001
G = −1724.327946

xyz

6	1.885529000	-0.098084000	0.175875000
7	0.630914000	-0.352053000	0.358861000
6	-0.276459000	0.751374000	0.522793000
6	-0.486828000	1.073958000	2.002820000
6	2.445958000	1.249774000	0.091526000
6	1.587789000	2.279253000	-0.120144000
6	2.011112000	3.660295000	-0.190677000
6	1.104789000	4.666199000	-0.249630000
6	-0.329685000	4.405979000	-0.240618000
6	-0.805844000	3.149429000	-0.253411000
6	0.114087000	1.960455000	-0.349712000
8	-1.587331000	0.331324000	-0.045860000
16	-2.047589000	-1.228620000	0.016135000
8	-1.960140000	-1.775082000	1.364987000
8	-1.579852000	-1.977185000	-1.142012000

6	-3.848088000	-0.825587000	-0.297604000
9	-4.517101000	-1.978655000	-0.337493000
9	-4.329201000	-0.067200000	0.687114000
9	-3.987927000	-0.189331000	-1.459534000
1	3.076806000	3.869600000	-0.150555000
1	1.443736000	5.698343000	-0.275304000
1	-1.009797000	5.253281000	-0.223153000
1	-1.873263000	2.948790000	-0.264464000
1	0.036007000	1.586312000	-1.388233000
1	3.514807000	1.392090000	0.169388000
1	-1.286764000	1.808101000	2.130830000
1	0.437308000	1.486469000	2.420031000
1	-0.745524000	0.160350000	2.540547000
7	2.713867000	-1.216765000	0.088660000
1	1.475271000	-2.720801000	-0.720646000
6	2.141548000	-2.562619000	0.133922000
1	1.553132000	-2.702747000	1.042751000
6	3.413170000	-3.424159000	0.090957000
1	3.347926000	-4.256579000	-0.611878000
1	3.691119000	-3.806957000	1.078264000
8	4.459058000	-2.540487000	-0.357905000
6	4.063487000	-1.244153000	-0.276182000
8	4.802249000	-0.315146000	-0.492791000

Final N-protonated product (**VIII**$_i$)

E = −1724.6306219
H = −1724.333251
G = −1724.410481

xyz

6	-0.418045000	3.383891000	0.515357000
7	-0.243080000	2.234994000	-0.281903000
6	0.856028000	2.452985000	-1.240374000
6	1.624211000	3.552018000	-0.509433000
8	0.617844000	4.216578000	0.293941000
6	-1.125537000	1.167601000	-0.280994000
7	-0.633394000	-0.042170000	-0.725447000
6	-1.351578000	-1.172828000	-0.789298000
6	-2.701643000	-1.158069000	-0.365705000
6	-3.243148000	0.075338000	0.123613000
6	-2.436323000	1.233089000	0.144922000
6	-3.522935000	-2.320210000	-0.396429000
6	-4.825158000	-2.258992000	0.037302000
6	-5.363520000	-1.036898000	0.518047000
6	-4.595657000	0.102502000	0.561155000

6	-0.656811000	-2.406371000	-1.292174000
8	1.916638000	-0.577798000	-1.331298000
16	2.980027000	-0.519053000	-0.246504000
6	2.424190000	-1.905136000	0.868210000
9	2.430819000	-3.076890000	0.212635000
8	-1.332315000	3.620004000	1.267136000
8	4.313602000	-0.903660000	-0.706943000
8	2.843616000	0.690323000	0.588903000
9	3.202489000	-2.009504000	1.945626000
9	1.152777000	-1.681669000	1.282544000
1	-5.006203000	1.036926000	0.932167000
1	-6.394863000	-1.006234000	0.857650000
1	-5.448139000	-3.147770000	0.014923000
1	-3.114149000	-3.257165000	-0.758325000
1	0.402889000	-0.141803000	-0.945686000
1	-2.823288000	2.173851000	0.509378000
1	-1.233330000	-2.864549000	-2.102095000
1	-0.566361000	-3.141034000	-0.484359000
1	0.347910000	-2.177339000	-1.649095000
1	0.442459000	2.790603000	-2.199884000
1	1.469429000	1.566412000	-1.387460000
1	2.066109000	4.295413000	-1.173079000
1	2.378113000	3.123231000	0.155042000

A.2) Methylthio derivative pathway

Starting methylthio derivative + acetonitrile + TfOH system (pre-association complex in Scheme X1) ($\mathbf{I}_i$)

E = −1840.6784723
H = −1840.426422
G = −1840.516214

xyz

6	2.887992000	-0.122393000	0.248678000
6	4.165660000	-0.673294000	0.182666000
6	5.278347000	0.157936000	0.036855000
6	5.113643000	1.543941000	-0.044314000
6	3.840628000	2.101446000	0.018324000
6	2.709565000	1.271572000	0.166043000
6	1.410095000	1.869767000	0.227964000
6	0.384686000	2.539215000	0.250811000
16	-1.016241000	3.469841000	0.400348000
6	-1.596808000	3.565505000	-1.346759000
7	2.679713000	-3.784535000	0.201074000
6	1.673005000	-4.300190000	-0.058078000
6	0.404505000	-4.946643000	-0.384573000

8	-1.660827000	0.072232000	-1.605564000
16	-1.716624000	-0.825757000	-0.458886000
8	-1.688842000	-2.271512000	-0.605137000
6	-3.274840000	-0.422204000	0.490446000
9	-3.327318000	0.887106000	0.731745000
8	-0.613598000	-0.415954000	0.633702000
9	-4.319193000	-0.780897000	-0.255753000
9	-3.292833000	-1.092228000	1.638725000
1	2.030008000	-0.776446000	0.365218000
1	4.273723000	-1.751542000	0.243458000
1	6.274649000	-0.273079000	-0.014107000
1	5.979043000	2.191279000	-0.157409000
1	3.703966000	3.176597000	-0.044900000
1	-2.519167000	4.151931000	-1.319088000
1	-0.854896000	4.075352000	-1.963723000
1	-1.799710000	2.564672000	-1.729857000
1	0.119478000	-5.640844000	0.412376000
1	-0.379810000	-4.191878000	-0.497455000
1	0.497420000	-5.506902000	-1.320407000
1	-0.175497000	0.455360000	0.410133000

Protonation TS (**II**$_i$)

E = −1840.6643589
H = −1840.417938
G = −1840.502597
ῡ = −812.7 cm^{-1}

xyz

6	-3.889006000	0.198729000	0.036859000
6	-4.959298000	-0.647498000	-0.231966000
6	-4.727472000	-1.963771000	-0.643973000
6	-3.419833000	-2.430223000	-0.789434000
6	-2.341446000	-1.586150000	-0.532340000
6	-2.567856000	-0.264530000	-0.111942000
6	-1.436526000	0.607850000	0.163360000
6	-1.133067000	1.700281000	0.725716000
16	-0.484105000	3.073707000	1.318054000
6	0.048855000	2.570222000	3.016290000
7	0.202029000	3.286964000	-1.997861000
6	1.065105000	2.693783000	-2.499021000
6	2.154494000	1.947805000	-3.123886000
8	0.789706000	-0.331869000	-0.830097000
16	1.997441000	-0.419571000	0.125696000
8	3.248723000	-0.090429000	-0.558772000
6	2.061917000	-2.252657000	0.448839000

9	0.873792000	-2.672704000	0.914413000
8	1.712090000	0.199402000	1.427858000
9	2.337157000	-2.914097000	-0.677892000
9	3.000992000	-2.523164000	1.357984000
1	-4.063050000	1.223356000	0.351818000
1	-5.976156000	-0.281812000	-0.120125000
1	-5.566133000	-2.622619000	-0.851418000
1	-3.237373000	-3.452677000	-1.107271000
1	-1.323312000	-1.946290000	-0.636266000
1	0.562439000	3.439657000	3.434177000
1	-0.820500000	2.318653000	3.625326000
1	0.733014000	1.726646000	2.918902000
1	2.885428000	2.639338000	-3.554966000
1	2.648366000	1.314070000	-2.380022000
1	1.761409000	1.308309000	-3.920436000
1	-0.278542000	0.188258000	-0.288154000

Protonated intermediate (**III**$_{i}$)

E = −1840.6670337
H = −1840.416092
G = −1840.502184

xyz

6	4.016902000	0.009873000	-0.337553000
6	5.171553000	-0.715637000	-0.068774000
6	5.101086000	-1.893624000	0.683769000
6	3.872358000	-2.343563000	1.170331000
6	2.710520000	-1.620450000	0.909138000
6	2.776366000	-0.439093000	0.150633000
6	1.530697000	0.278726000	-0.101549000
6	1.212198000	1.294032000	-0.837195000
16	0.662484000	2.573616000	-1.627277000
6	0.022075000	1.857314000	-3.208375000
7	0.163153000	3.464228000	1.459359000
6	-0.774436000	3.156770000	2.071816000
6	-1.955936000	2.758698000	2.832189000
8	-0.780375000	-0.909637000	1.028148000
16	-1.973274000	-0.413539000	0.259996000
8	-3.038741000	0.162116000	1.097458000
6	-2.707446000	-1.976836000	-0.429646000
9	-1.813006000	-2.603501000	-1.211525000
8	-1.594828000	0.370621000	-0.942461000
9	-3.063986000	-2.807241000	0.556978000
9	-3.789072000	-1.695265000	-1.167743000
1	4.069850000	0.929085000	-0.914780000

1	6.128988000	-0.364656000	-0.442950000
1	6.006399000	-2.457316000	0.891156000
1	3.817683000	-3.256892000	1.755297000
1	1.747254000	-1.959346000	1.279043000
1	-0.412275000	2.693576000	-3.761315000
1	0.837913000	1.406738000	-3.775040000
1	-0.740037000	1.126916000	-2.931343000
1	-2.666446000	3.590002000	2.883378000
1	-2.436720000	1.900525000	2.345940000
1	-1.669049000	2.477731000	3.850574000
1	0.597518000	-0.125193000	0.407781000

Acetonitrile addition TS (**IV**$_i$)

E = −1840.6399379
H = −1840.389398
G = −1840.472378
ῡ = −338.1 cm^{-1}

xyz

6	1.219745000	1.461547000	-0.124347000
6	2.229434000	2.028005000	0.676994000
6	2.304165000	3.408286000	0.833904000
6	1.373752000	4.241328000	0.204381000
6	0.350554000	3.687321000	-0.566823000
6	0.262569000	2.306615000	-0.722047000
6	1.048875000	0.032781000	-0.354185000
6	1.782886000	-1.057893000	-0.253477000
7	3.624157000	-0.600122000	0.099878000
6	4.753465000	-0.720086000	-0.128779000
6	6.174622000	-0.871474000	-0.409137000
16	1.690422000	-2.727637000	-0.272053000
6	0.894585000	-3.142873000	1.341834000
8	-1.624359000	-1.289571000	0.945190000
16	-2.431537000	-1.037084000	-0.277284000
8	-3.359710000	-2.097682000	-0.685319000
8	-1.615574000	-0.441084000	-1.389303000
6	-3.519263000	0.387813000	0.221948000
9	-4.330003000	0.747754000	-0.784044000
9	-4.275215000	0.067603000	1.281295000
9	-2.769125000	1.459329000	0.553142000
1	2.930570000	1.389693000	1.200609000
1	3.080287000	3.837328000	1.461836000
1	1.436020000	5.318459000	0.333887000
1	-0.389968000	4.328808000	-1.035303000
1	-0.550466000	1.861329000	-1.287012000

1	0.734663000	-4.224245000	1.308359000
1	1.566674000	-2.892034000	2.164786000
1	-0.058862000	-2.609547000	1.400746000
1	6.485978000	-0.133134000	-1.154470000
1	6.756225000	-0.723691000	0.506142000
1	6.369756000	-1.876230000	-0.797982000
1	0.041765000	-0.254042000	-0.751029000

Acetonitrile addition intermediate (**V**$_i$)

E = −1840.7184693
H = −1840.463937
G = −1840.541304

xyz

6	-1.625387000	1.567359000	-0.010534000
7	-0.397174000	0.879588000	-0.022988000
6	0.179373000	0.512477000	1.036892000
6	-0.230662000	0.607661000	2.474398000
6	-2.842861000	0.990215000	-0.111283000
6	-3.202119000	-0.433491000	-0.154380000
6	-4.530480000	-0.784948000	0.157622000
6	-4.954259000	-2.110952000	0.127596000
6	-4.061179000	-3.122359000	-0.231721000
6	-2.746430000	-2.789236000	-0.565457000
6	-2.316117000	-1.464298000	-0.527367000
8	1.425756000	-0.110299000	0.942766000
16	2.154967000	-0.233136000	-0.559154000
8	2.516684000	1.080882000	-1.063962000
8	1.500478000	-1.247495000	-1.367253000
6	3.704588000	-1.001936000	0.166801000
9	4.536333000	-1.230352000	-0.847695000
9	4.267205000	-0.164193000	1.031717000
9	3.404625000	-2.146720000	0.771418000
1	-5.233530000	-0.001381000	0.431303000
1	-5.983330000	-2.353831000	0.379452000
1	-4.388661000	-4.158108000	-0.260788000
1	-2.047977000	-3.566097000	-0.865524000
1	-1.299042000	-1.228257000	-0.818978000
1	-3.682967000	1.679143000	-0.143605000
1	-0.221061000	-0.387576000	2.930525000
1	-1.231699000	1.034189000	2.547512000
1	0.477361000	1.236965000	3.023753000
16	-1.492931000	3.347998000	0.148679000
6	-0.285518000	3.745817000	-1.177709000
1	-0.720718000	3.559160000	-2.162170000

1	-0.064282000	4.811226000	-1.073789000
1	0.633037000	3.168481000	-1.059693000

Friedel-Crafts-like TS (**VI**$_i$)

E = −1840.6607703
H = −1840.408880
G = −1840.481522
ῡ = −496.9 cm^{-1}

xyz

6	-1.462355000	1.583260000	-0.167477000
7	-0.814231000	1.013076000	0.853397000
6	-0.353992000	-0.222754000	0.770735000
6	0.031535000	-0.948229000	2.031789000
6	-2.393684000	0.905608000	-0.975088000
6	-2.730947000	-0.440393000	-0.726845000
6	-4.097285000	-0.849699000	-0.744553000
6	-4.480767000	-2.039706000	-0.174621000
6	-3.521328000	-2.920527000	0.403707000
6	-2.187231000	-2.622481000	0.320951000
6	-1.733754000	-1.391875000	-0.272987000
8	0.658792000	-0.450871000	-0.314140000
16	2.079103000	0.351522000	-0.321320000
8	2.278559000	1.130829000	0.894255000
8	2.271933000	0.917653000	-1.644283000
6	3.198669000	-1.143878000	-0.198906000
9	4.461847000	-0.725698000	-0.249073000
9	2.984478000	-1.773007000	0.959893000
9	2.960513000	-1.978958000	-1.207077000
1	-4.842812000	-0.163126000	-1.137802000
1	-5.533878000	-2.306765000	-0.143962000
1	-3.853496000	-3.851754000	0.854106000
1	-1.445100000	-3.342921000	0.655905000
1	-0.879814000	-1.507219000	-0.931000000
1	-3.119726000	1.525983000	-1.495370000
1	0.349421000	-1.970320000	1.817386000
1	-0.828033000	-0.972768000	2.705927000
1	0.853557000	-0.418594000	2.525091000
16	-1.442405000	3.352857000	-0.291117000
6	-0.311075000	3.819614000	1.064967000
1	-0.778617000	3.652928000	2.036475000
1	0.618894000	3.252205000	1.000868000
1	-0.110472000	4.885183000	0.927218000

Friedel-Crafts-like intermediate (**VII**$_i$)

E = −1840.7022834
H = −1840.447755
G = −1840.519412

xyz

6	0.940393000	1.281250000	-0.149940000
6	0.428733000	2.669138000	0.131703000
6	1.203264000	3.752947000	-0.047595000
6	2.597030000	3.619777000	-0.453766000
6	3.187761000	2.407542000	-0.591098000
6	2.451611000	1.189025000	-0.337896000
6	3.017102000	-0.043576000	-0.301593000
6	2.161725000	-1.193168000	-0.003602000
16	2.859801000	-2.772810000	-0.398655000
6	1.514701000	-3.888241000	0.126924000
7	0.982116000	-1.115159000	0.508751000
6	0.492660000	0.199414000	0.855355000
6	0.810464000	0.522078000	2.315590000
8	-1.244068000	-0.595772000	-1.585699000
16	-1.796386000	-0.751554000	-0.243625000
8	-2.145826000	-2.058899000	0.287530000
6	-3.341589000	0.301138000	-0.159000000
9	-3.056448000	1.561499000	-0.499671000
8	-0.995931000	0.148451000	0.844815000
9	-4.224559000	-0.199735000	-1.022704000
9	-3.854396000	0.278265000	1.068705000
1	0.497029000	0.977325000	-1.115345000
1	-0.617759000	2.759438000	0.408464000
1	0.801084000	4.750759000	0.105024000
1	3.175740000	4.524277000	-0.621327000
1	4.240748000	2.326897000	-0.848810000
1	1.856809000	-4.901941000	-0.094661000
1	1.326869000	-3.776299000	1.195993000
1	0.599279000	-3.666948000	-0.423821000
1	0.484015000	-0.309173000	2.944183000
1	1.889994000	0.656493000	2.431195000
1	0.305349000	1.437485000	2.635114000
1	4.073485000	-0.196828000	-0.501400000

Final N-protonated product (**VIII**$_{i}$)

E = −1840.7834835
H = −1840.527750
G = −1840.602810

xyz

6	-3.099539000	-0.733113000	-0.094875000
6	-4.033606000	-1.791948000	0.052482000
6	-5.363772000	-1.517251000	0.279976000
6	-5.815023000	-0.178474000	0.366284000
6	-4.931688000	0.869494000	0.228390000
6	-3.555589000	0.619178000	-0.002220000
6	-2.607853000	1.669189000	-0.128450000
6	-1.282472000	1.391693000	-0.341137000
16	-0.063390000	2.665320000	-0.575852000
6	0.840699000	2.603901000	1.029437000
7	-0.890734000	0.072417000	-0.449330000
6	-1.714129000	-0.969953000	-0.334954000
6	-1.126983000	-2.345792000	-0.465943000
8	1.784746000	-0.528161000	1.454121000
16	2.395170000	-1.055736000	0.211390000
8	2.859230000	-2.445970000	0.225926000
6	3.924756000	-0.026394000	-0.040172000
9	3.602873000	1.279486000	-0.114280000
8	1.575017000	-0.691249000	-1.009650000
9	4.770912000	-0.191422000	0.984162000
9	4.547958000	-0.371059000	-1.172010000
1	-3.695310000	-2.820187000	-0.008218000
1	-6.073033000	-2.331119000	0.394972000
1	-6.867832000	0.020492000	0.544365000
1	-5.274965000	1.897766000	0.297170000
1	1.707562000	3.254488000	0.890742000
1	0.210334000	2.995951000	1.830522000
1	1.185977000	1.591631000	1.251265000
1	-0.100170000	-2.300511000	-0.831148000
1	-1.119223000	-2.845176000	0.510502000
1	-1.724611000	-2.955462000	-1.150374000
1	-2.924798000	2.702778000	-0.046911000
1	0.146315000	-0.144100000	-0.643132000

B) Pyrimidine route

B.1) Oxazolidinone derivative pathway

Starting oxazolidinone derivative + 2 acetonitrile + TfOH system (pre-association complex in Scheme X2)

($\mathbf{I_p}$)

E = −1857.2847149
H = −1856.939577
G = −1857.045337

xyz

6	-3.724721000	-1.548476000	1.804924000
6	-2.339872000	-1.981828000	1.690550000
8	1.727408000	-0.533130000	0.915025000
16	2.271635000	-1.879205000	0.735869000
6	1.968361000	-2.307457000	-1.055648000
9	2.554160000	-1.383144000	-1.827004000
7	-1.237271000	-2.320614000	1.605685000
8	3.661523000	-2.185181000	1.016053000
8	1.361751000	-2.965948000	1.446348000
9	2.472776000	-3.502699000	-1.342767000
9	0.652560000	-2.311743000	-1.300215000
1	-4.111927000	-1.288446000	0.814665000
1	-4.331462000	-2.352194000	2.233721000
1	-3.760060000	-0.667951000	2.456271000
1	0.389534000	-2.673802000	1.508386000
7	-2.511111000	0.967632000	3.508874000
6	-1.426740000	1.336133000	3.319190000
6	-0.063903000	1.797722000	3.075664000
1	0.507501000	1.016392000	2.562796000
1	-0.073735000	2.693983000	2.446271000
1	0.427692000	2.033010000	4.025115000
6	-2.400615000	0.737539000	-1.427468000
6	-3.617511000	1.396383000	-1.159593000
6	-4.831091000	0.750461000	-1.383270000
6	-4.857538000	-0.555236000	-1.882781000
6	-3.654652000	-1.217121000	-2.148162000
6	-2.435303000	-0.583448000	-1.919032000
6	-1.154780000	1.396398000	-1.215357000
6	-0.097628000	1.963178000	-1.035247000
1	-3.596779000	2.412439000	-0.778408000
1	-5.761025000	1.272232000	-1.173713000
1	-5.806748000	-1.050751000	-2.067698000
1	-3.665617000	-2.232477000	-2.535805000
1	-1.500788000	-1.100281000	-2.111487000
7	1.095226000	2.567388000	-0.899561000
6	1.337055000	3.600958000	0.011435000
8	0.537469000	4.142998000	0.732139000
8	2.660187000	3.905094000	-0.033167000
6	3.313941000	3.164412000	-1.082826000
1	3.473051000	3.838286000	-1.931028000
1	4.277470000	2.819889000	-0.704358000
1	2.593548000	1.083565000	-0.910209000
6	2.349499000	2.016737000	-1.424910000
1	2.276125000	1.832017000	-2.499529000

Protonation TS ($\mathbf{II_p}$)

E = −1857.2701564
H = −1856.930098
G = −1857.034182
ῡ = −827.0 cm^{-1}

xyz

6	2.508494000	1.375621000	1.445954000
7	2.076878000	1.611689000	0.051732000
6	3.182342000	2.023526000	-0.780842000
8	4.271862000	2.079044000	-0.007859000
6	4.035030000	1.552620000	1.318644000
6	0.867400000	1.480590000	-0.397045000
6	-0.269559000	1.144730000	-0.810802000
6	-1.594668000	1.686968000	-1.078499000
6	-1.919106000	3.010177000	-0.719561000
6	-3.189400000	3.516077000	-0.978622000
6	-4.152676000	2.715039000	-1.599852000
6	-3.840430000	1.402153000	-1.956409000
6	-2.571822000	0.887088000	-1.695205000
8	3.110341000	2.303469000	-1.941161000
8	-0.162298000	-1.459087000	-1.060945000
16	-0.177195000	-2.246245000	0.282765000
6	-1.980449000	-2.697585000	0.415124000
9	-2.377387000	-3.366067000	-0.667791000
8	0.088411000	-1.371766000	1.431911000
8	0.540450000	-3.510410000	0.144370000
9	-2.173948000	-3.451664000	1.498292000
9	-2.716194000	-1.578931000	0.528329000
7	3.668064000	-1.120668000	-0.352869000
6	3.469422000	-2.066049000	-0.995219000
6	3.207194000	-3.258386000	-1.794546000
6	-2.051282000	1.001814000	2.808475000
6	-1.045068000	2.059613000	2.792913000
7	-0.235205000	2.891312000	2.765144000
1	-1.171460000	3.627096000	-0.230779000
1	-3.427840000	4.538236000	-0.697871000
1	-5.142383000	3.114205000	-1.804254000
1	-4.585534000	0.775784000	-2.438784000
1	-2.331194000	-0.134389000	-1.970901000
1	4.056216000	-3.946564000	-1.736073000
1	2.308741000	-3.751019000	-1.411130000
1	3.047454000	-2.980425000	-2.840934000
1	-0.142243000	-0.285077000	-0.938817000
1	-1.605788000	0.070166000	2.446232000
1	-2.428072000	0.851551000	3.824990000
1	-2.886493000	1.271396000	2.154792000
1	4.561152000	0.601231000	1.389599000

1	4.440456000	2.273900000	2.030191000
1	2.033663000	2.108463000	2.101289000
1	2.225375000	0.365020000	1.743134000

Protonated intermediate (**III$_p$**)

E = −1857.2830051
H = −1856.938022
G = −1857.039039

xyz

6	-1.459598000	3.340174000	-0.600699000
6	-2.641652000	4.056569000	-0.751918000
6	-3.759716000	3.453674000	-1.338053000
6	-3.694592000	2.127872000	-1.770535000
6	-2.512893000	1.403842000	-1.626612000
6	-1.387155000	2.007467000	-1.041868000
6	-0.172995000	1.201914000	-0.913087000
6	0.942149000	1.421996000	-0.290435000
7	3.497310000	-0.850028000	-1.026112000
6	3.133829000	-1.806847000	-1.573465000
6	2.674513000	-3.014735000	-2.249978000
8	-0.188325000	-1.711861000	-1.372426000
16	-0.195225000	-2.307458000	-0.001664000
8	0.455225000	-3.623713000	0.103075000
6	-2.003343000	-2.646961000	0.288024000
9	-2.726290000	-1.520484000	0.088384000
8	0.132564000	-1.330942000	1.073543000
9	-2.462104000	-3.586848000	-0.542031000
9	-2.217631000	-3.047973000	1.552538000
1	-0.593771000	3.807382000	-0.141066000
1	-2.693066000	5.086902000	-0.412211000
1	-4.680226000	4.018723000	-1.455555000
1	-4.563043000	1.656449000	-2.221285000
1	-2.456047000	0.366852000	-1.943279000
1	3.528083000	-3.574409000	-2.644788000
1	2.108936000	-3.627332000	-1.539973000
1	2.001191000	-2.743154000	-3.067613000
1	-0.163262000	0.181638000	-1.354096000
7	-0.440438000	2.184075000	2.643380000
6	-1.163716000	1.285767000	2.781153000
6	-2.055870000	0.142164000	2.940901000
1	-1.482375000	-0.780471000	2.816187000
1	-2.528964000	0.163816000	3.927397000
1	-2.830282000	0.161188000	2.168856000
7	2.041607000	1.460505000	0.324348000

6	3.275286000	2.070583000	-0.251806000
8	3.294410000	2.713440000	-1.252535000
8	4.259339000	1.815166000	0.597210000
6	3.879143000	0.846264000	1.611776000
1	4.258055000	-0.123027000	1.288834000
1	4.346388000	1.163254000	2.544025000
1	1.945841000	1.560433000	2.416750000
6	2.345853000	0.883015000	1.660008000
1	1.869827000	-0.096300000	1.731746000

First acetonitrile addition TS (**IV**$_{\mathbf{p}}$)

E = −1857.2695101
H = −1856.925134
G = −1857.023739
ῡ = −225.3 cm^{-1}

xyz

6	2.257182000	-1.328845000	-1.081411000
6	3.618171000	-1.078658000	-1.323078000
6	4.523491000	-2.136994000	-1.356500000
6	4.082848000	-3.446930000	-1.150362000
6	2.728071000	-3.701267000	-0.923066000
6	1.813096000	-2.651563000	-0.900723000
6	1.244183000	-0.275565000	-1.058112000
6	1.217381000	0.963720000	-0.622485000
7	2.885661000	1.279100000	0.453706000
6	3.335494000	1.460759000	1.506062000
6	3.909305000	1.675514000	2.825358000
8	-1.850677000	0.331704000	-1.149536000
16	-2.206317000	-0.522718000	0.015237000
8	-2.357655000	0.226690000	1.289705000
8	-1.403568000	-1.769878000	0.121627000
6	-3.927306000	-1.117179000	-0.366885000
9	-4.374941000	-1.913986000	0.615595000
9	-4.765535000	-0.077134000	-0.485033000
9	-3.942121000	-1.808463000	-1.514303000
1	3.954113000	-0.065025000	-1.511898000
1	5.573276000	-1.940421000	-1.556214000
1	4.792922000	-4.269024000	-1.180060000
1	2.381772000	-4.719722000	-0.771816000
1	0.756370000	-2.832800000	-0.723331000
1	4.780749000	1.027910000	2.962809000
1	4.214540000	2.720081000	2.939382000
1	3.138165000	1.423122000	3.561430000
1	0.236071000	-0.529237000	-1.407218000

7	0.977778000	0.454252000	3.433115000
6	0.186588000	-0.395372000	3.408636000
6	-0.797046000	-1.471884000	3.380414000
1	-0.855836000	-1.888093000	2.368684000
1	-0.520706000	-2.249018000	4.100262000
1	-1.785100000	-1.072335000	3.622400000
7	0.550079000	2.071512000	-0.584646000
6	0.460924000	2.975749000	-1.718210000
8	1.127603000	2.912950000	-2.707730000
8	-0.478135000	3.880996000	-1.426419000
6	-1.209686000	3.503251000	-0.225648000
1	-1.462488000	4.422033000	0.302894000
1	-2.107022000	2.963862000	-0.533783000
1	-0.777125000	1.776624000	1.053269000
6	-0.254355000	2.586477000	0.541825000
1	0.399520000	3.130774000	1.231786000

First acetonitrile addition intermediate (**V_p**)

E = −1857.2941386
H = −1856.948276
G = −1857.044628

xyz

6	-2.690341000	2.314936000	-1.305508000
6	-2.967920000	3.678414000	-1.253720000
6	-1.938993000	4.593295000	-1.014953000
6	-0.629184000	4.138544000	-0.845628000
6	-0.339143000	2.777721000	-0.911137000
6	-1.375034000	1.845862000	-1.121552000
6	-1.026827000	0.425371000	-1.187909000
6	-1.735602000	-0.609857000	-0.674766000
7	-1.924230000	0.274546000	3.163569000
6	-0.769157000	0.364836000	3.247913000
6	0.681344000	0.470158000	3.346757000
8	2.079387000	0.086312000	-1.391285000
16	2.500208000	0.139511000	0.033343000
8	2.304287000	1.453675000	0.689942000
6	4.348095000	-0.065038000	-0.042516000
9	4.668859000	-1.231812000	-0.623613000
8	2.045044000	-1.031087000	0.836190000
9	4.904964000	0.927278000	-0.750442000
9	4.871471000	-0.051016000	1.193192000
1	-3.486783000	1.614866000	-1.544321000
1	-3.983550000	4.029296000	-1.416133000
1	-2.156823000	5.657116000	-0.974806000

1	0.175177000	4.845880000	-0.665388000
1	0.676057000	2.422501000	-0.760841000
1	1.154030000	-0.404944000	2.887771000
1	1.042094000	1.334391000	2.779999000
1	0.980055000	0.558098000	4.396066000
1	-0.053143000	0.183530000	-1.614112000
7	-2.842991000	-0.365424000	0.122778000
6	-3.699475000	-0.261592000	0.885154000
6	-4.787102000	-0.140326000	1.826924000
1	-5.441331000	0.687303000	1.533111000
1	-5.356661000	-1.076211000	1.823630000
1	-4.350512000	0.048241000	2.813792000
7	-1.385643000	-1.964336000	-0.720154000
6	-2.318244000	-3.001356000	-0.714745000
8	-3.515078000	-2.907324000	-0.552336000
8	-1.664946000	-4.167191000	-0.909748000
6	-0.234523000	-3.936873000	-0.912487000
1	0.158239000	-4.228776000	0.066110000
1	0.205228000	-4.563608000	-1.688759000
1	0.081870000	-2.201757000	-2.229940000
6	-0.069329000	-2.434143000	-1.168216000
1	0.734194000	-1.986443000	-0.579045000

Second acetonitrile addition TS (**VI_p**)

E = −1857.2836703
H = −1856.937896
G = −1857.029689
$\bar{\upsilon} = -277.8\ cm^{-1}$

xyz

6	-2.163653000	1.575377000	-0.803441000
6	-3.536420000	1.471288000	-1.095328000
6	-4.387685000	2.554249000	-0.886089000
6	-3.885443000	3.756518000	-0.382110000
6	-2.522050000	3.874697000	-0.099829000
6	-1.663634000	2.800142000	-0.317162000
6	-1.203256000	0.488607000	-1.001450000
6	-1.385178000	-0.838158000	-0.823484000
7	-2.596820000	-1.357432000	-0.324023000
6	-3.180791000	-1.555777000	0.697635000
6	-4.415143000	-2.130463000	1.247149000
7	-2.304161000	-0.872578000	2.233438000
6	-1.177341000	-0.777231000	2.516749000
6	0.206439000	-0.623675000	2.904943000
8	2.256629000	-0.816719000	0.376437000

16	2.245412000	0.630355000	0.037675000
8	1.455951000	1.474369000	0.976780000
8	1.999655000	0.925891000	-1.397750000
6	4.002577000	1.176950000	0.309760000
9	4.137168000	2.482993000	0.043040000
9	4.367501000	0.964540000	1.583818000
9	4.834853000	0.491552000	-0.488173000
1	-3.929829000	0.553998000	-1.524006000
1	-5.442967000	2.464882000	-1.130589000
1	-4.551344000	4.600200000	-0.222218000
1	-2.124364000	4.809355000	0.285777000
1	-0.604274000	2.880463000	-0.087625000
1	-4.153648000	-2.910203000	1.968693000
1	-4.976967000	-1.352085000	1.770654000
1	-5.011133000	-2.553382000	0.434566000
1	-0.194732000	0.785043000	-1.283297000
1	0.618822000	0.287505000	2.442444000
1	0.286633000	-0.584599000	3.996318000
1	0.776890000	-1.471277000	2.510617000
7	-0.446011000	-1.819528000	-1.153799000
6	-0.132549000	-2.881602000	-0.313155000
8	-0.672164000	-3.157376000	0.739370000
8	0.855149000	-3.607272000	-0.868402000
6	1.460782000	-2.864207000	-1.953101000
1	2.386420000	-2.427958000	-1.575458000
1	1.660526000	-3.564706000	-2.765425000
1	-0.127573000	-1.997824000	-3.232857000
6	0.440175000	-1.770284000	-2.322316000
1	0.931446000	-0.799823000	-2.411965000

Second acetonitrile addition intermediate (**VII_p**)

E = −1857.3226126
H = −1856.974111
G = −1857.063109

xyz

6	1.083978000	3.272536000	-1.295846000
6	2.363628000	3.804262000	-1.161141000
6	3.049965000	3.677976000	0.048189000
6	2.438703000	3.014828000	1.115301000
6	1.161992000	2.474158000	0.981076000
6	0.454181000	2.592722000	-0.233111000
6	-0.896243000	2.079528000	-0.464932000
6	-1.675497000	1.298033000	0.314589000
7	-1.357827000	0.906511000	1.627624000

6	-1.326000000	-0.332294000	1.960364000
6	-1.035834000	-0.733540000	3.381049000
8	1.602194000	-2.474808000	-1.834196000
16	1.749498000	-2.261833000	-0.404708000
8	0.457193000	-1.422367000	0.212344000
6	2.952510000	-0.843219000	-0.137864000
9	4.162864000	-1.311462000	-0.440778000
8	2.087837000	-3.346796000	0.504903000
9	2.639117000	0.161581000	-0.937623000
9	2.922297000	-0.459929000	1.132980000
1	0.555661000	3.376501000	-2.240785000
1	2.824490000	4.318464000	-2.000591000
1	4.048782000	4.091953000	0.158425000
1	2.962376000	2.915717000	2.063099000
1	0.697730000	1.965930000	1.817146000
1	-1.850256000	-1.347698000	3.781418000
1	-0.123172000	-1.341100000	3.420514000
1	-0.907036000	0.157751000	3.997359000
1	-1.319250000	2.324606000	-1.434792000
7	-1.645961000	-1.400560000	1.106945000
6	-0.854511000	-1.941176000	0.288759000
6	-1.226724000	-3.053920000	-0.636066000
1	-1.136031000	-2.712650000	-1.671107000
1	-0.576989000	-3.923833000	-0.492198000
1	-2.260173000	-3.336840000	-0.435678000
7	-2.951537000	0.887472000	-0.174702000
6	-3.122683000	0.076268000	-1.284443000
8	-2.286334000	-0.316449000	-2.063423000
8	-4.448734000	-0.247115000	-1.387406000
6	-5.200045000	0.482249000	-0.401850000
1	-5.666411000	1.347668000	-0.886424000
1	-5.976881000	-0.174996000	-0.006733000
1	-4.094205000	0.186303000	1.472155000
6	-4.159069000	0.904287000	0.644349000
1	-4.349199000	1.899960000	1.054923000

Pyrimidine scaffold formation TS (**VIII$_p$**)

E = −1857.2918924
H = −1856.945201
G = −1857.029964
ῡ = −368.7 cm^{-1}

xyz

6	2.830464000	-0.148698000	-0.711047000
6	3.266472000	-1.148084000	-1.605965000

6	4.607929000	-1.511139000	-1.693212000
6	5.559171000	-0.877682000	-0.891436000
6	5.147289000	0.122922000	-0.008913000
6	3.804115000	0.482561000	0.087494000
6	1.391719000	0.173723000	-0.704990000
6	0.779959000	1.306931000	-0.127578000
7	1.157727000	1.838783000	1.047380000
6	1.205700000	1.076986000	2.124071000
7	0.950306000	-0.237209000	2.117753000
6	0.583362000	-0.960202000	1.096056000
6	1.182654000	-2.317427000	0.851944000
6	1.553199000	1.714577000	3.435622000
8	-0.879155000	-0.888969000	0.871932000
16	-1.713469000	-1.880887000	-0.107796000
8	-1.307082000	-1.703387000	-1.501774000
8	-1.843223000	-3.213397000	0.463258000
6	-3.349165000	-0.991695000	0.115570000
9	-3.271910000	0.233501000	-0.406106000
9	-4.268351000	-1.695801000	-0.544402000
9	-3.667206000	-0.921817000	1.399434000
1	2.536772000	-1.639417000	-2.246024000
1	4.910174000	-2.285669000	-2.393290000
1	6.607128000	-1.156895000	-0.956158000
1	5.878387000	0.631744000	0.614160000
1	3.511581000	1.273343000	0.767467000
1	2.089548000	1.014872000	4.080657000
1	2.142423000	2.619329000	3.270141000
1	0.624071000	2.005075000	3.941237000
1	0.850266000	-0.273883000	-1.532953000
1	0.768641000	-3.014909000	1.589153000
1	0.967992000	-2.700358000	-0.147292000
1	2.261676000	-2.253636000	0.989313000
7	-0.339280000	1.849598000	-0.740891000
6	-1.347925000	2.585936000	-0.079077000
8	-1.500964000	2.757244000	1.097660000
8	-2.192036000	3.083170000	-1.028181000
6	-1.673701000	2.830817000	-2.344833000
1	-2.510074000	2.578720000	-2.998644000
1	-1.185214000	3.741190000	-2.709743000
1	0.207290000	1.772012000	-2.784103000
6	-0.680445000	1.677568000	-2.154137000
1	-1.144320000	0.701663000	-2.329783000

Pyrimidine intermediate (**IX$_p$**)

E = −1857.3508002
H = −1857.000863

G = −1857.086086

xyz

6	2.263501000	-0.910002000	-0.510028000
6	2.579336000	-1.161274000	-1.852492000
6	3.829033000	-1.667319000	-2.207268000
6	4.787334000	-1.922036000	-1.225113000
6	4.487304000	-1.662196000	0.112418000
6	3.235252000	-1.158768000	0.468219000
6	0.864299000	-0.383080000	-0.176004000
6	0.896155000	0.762752000	0.818046000
7	0.794297000	0.578568000	2.097508000
6	0.577685000	-0.735786000	2.531291000
7	0.158817000	-1.729656000	1.820464000
6	-0.078655000	-1.457327000	0.430123000
6	-0.161820000	-2.753751000	-0.351981000
6	0.838035000	-0.943180000	3.995496000
8	-1.498437000	-0.846889000	0.485328000
16	-2.219181000	-0.085563000	-0.727452000
8	-2.103072000	1.359623000	-0.551583000
8	-1.928333000	-0.698786000	-2.020198000
6	-3.964002000	-0.566039000	-0.253863000
9	-4.251777000	-0.113850000	0.964151000
9	-4.791981000	-0.016076000	-1.142976000
9	-4.091729000	-1.890564000	-0.285278000
1	1.845770000	-0.943411000	-2.622726000
1	4.055184000	-1.853139000	-3.253631000
1	5.763098000	-2.312807000	-1.500611000
1	5.228460000	-1.849519000	0.884775000
1	3.025366000	-0.955688000	1.513125000
1	0.675334000	-1.987126000	4.266784000
1	1.863156000	-0.645109000	4.246049000
1	0.170877000	-0.300361000	4.581832000
1	0.440937000	-0.033913000	-1.116715000
1	-0.933260000	-3.380407000	0.102318000
1	-0.398259000	-2.579455000	-1.403200000
1	0.795133000	-3.276141000	-0.275761000
7	1.024988000	2.060023000	0.385607000
6	1.195377000	2.509494000	-0.939077000
8	1.374109000	1.848448000	-1.928428000
8	1.155932000	3.862300000	-0.934298000
6	0.734621000	4.353709000	0.353099000
1	1.329105000	5.237936000	0.587549000
1	-0.322325000	4.628538000	0.283765000
1	0.150051000	3.044622000	2.032434000
6	0.964178000	3.184164000	1.320481000
1	1.903536000	3.263134000	1.878466000

Final N-protonated product (**X**$_p$)

E = −1857.3998465
H = −1857.050274
G = −1857.138442

xyz

6	0.182149000	0.663472000	-0.607254000
6	1.500270000	0.335640000	-0.266968000
6	1.768396000	-1.052646000	-0.141607000
7	0.816953000	-2.000923000	-0.212601000
6	-0.415557000	-1.631713000	-0.512482000
7	-0.722047000	-0.339704000	-0.750627000
6	2.491209000	1.394849000	0.085966000
6	2.908281000	1.518292000	1.419032000
6	3.798881000	2.526006000	1.790431000
6	4.279329000	3.421791000	0.834733000
6	3.868817000	3.304103000	-0.494793000
6	2.982544000	2.295967000	-0.868599000
6	-1.511320000	-2.646865000	-0.612865000
8	-3.250727000	0.034060000	-1.283762000
16	-3.996897000	0.849536000	-0.244414000
8	-3.084234000	1.348245000	0.809050000
6	-0.356184000	2.048621000	-0.784347000
8	-4.979726000	1.777888000	-0.803103000
6	-5.001657000	-0.457875000	0.619684000
9	-4.186577000	-1.388301000	1.172276000
9	-5.732215000	0.079670000	1.600782000
9	-5.818135000	-1.082662000	-0.235890000
1	2.522582000	0.833469000	2.169805000
1	4.107839000	2.615437000	2.828145000
1	4.970604000	4.208341000	1.123923000
1	4.245137000	3.993188000	-1.245325000
1	2.687349000	2.189568000	-1.907802000
1	-1.921783000	-2.664809000	-1.628475000
1	-1.114950000	-3.630844000	-0.361451000
1	-2.339500000	-2.388875000	0.053433000
1	-1.757441000	-0.106218000	-0.970677000
1	-0.710513000	2.181698000	-1.814003000
1	-1.225767000	2.182158000	-0.128174000
1	0.399090000	2.802086000	-0.570002000
7	3.040262000	-1.544367000	0.126041000
6	4.197341000	-1.170839000	-0.597628000
8	4.288026000	-0.334507000	-1.453479000
8	5.214777000	-1.974308000	-0.199580000
6	4.774275000	-2.846399000	0.860145000

1	5.247217000	-3.818571000	0.716466000
1	5.088220000	-2.419341000	1.818439000
6	3.250337000	-2.873699000	0.710799000
1	2.907936000	-3.662121000	0.032360000
1	2.722916000	-2.974399000	1.661284000

B.2) Methylthio derivative pathway

Starting methylthio derivative + 2 acetonitrile + TfOH system (pre-association complex in Scheme X2) (**I_p**)

E = −1973.4508472
H = −1973.146554
G = −1973.247926

xyz

6	-2.650061000	-2.156783000	2.155593000
6	-1.223573000	-2.201585000	1.867553000
8	2.017616000	0.414509000	0.885019000
16	2.977605000	-0.630185000	0.527848000
6	2.692785000	-0.975285000	-1.284537000
9	2.823537000	0.164311000	-1.967833000
7	-0.089014000	-2.230490000	1.643793000
8	4.405133000	-0.453623000	0.710290000
8	2.553580000	-2.025158000	1.156315000
9	3.573099000	-1.865276000	-1.730743000
9	1.454119000	-1.454323000	-1.459428000
1	-3.213499000	-2.127205000	1.217686000
1	-2.941840000	-3.043184000	2.727555000
1	-2.860397000	-1.254087000	2.740243000
1	1.556218000	-2.069407000	1.339878000
7	-2.088289000	0.762680000	3.590545000
6	-1.164251000	1.407860000	3.313001000
6	0.000253000	2.215609000	2.963642000
1	0.762906000	1.587704000	2.492254000
1	-0.284568000	2.999614000	2.254472000
1	0.419471000	2.680175000	3.861801000
6	-2.696647000	0.287116000	-1.203107000
6	-4.090289000	0.327351000	-0.994528000
6	-4.860376000	-0.820909000	-1.162586000
6	-4.261894000	-2.026168000	-1.542115000
6	-2.879583000	-2.075496000	-1.748046000
6	-2.099209000	-0.934009000	-1.579023000
6	-1.901389000	1.457952000	-1.043712000
6	-1.208023000	2.451949000	-0.921653000
16	-0.300745000	3.864710000	-0.730336000
1	-4.555927000	1.263719000	-0.703576000

1	-5.934083000	-0.773288000	-1.001673000
1	-4.867789000	-2.917277000	-1.681601000
1	-2.406041000	-3.007616000	-2.044999000
1	-1.025717000	-0.972609000	-1.730652000
6	1.374075000	3.287976000	-1.233845000
1	1.369765000	2.992672000	-2.284739000
1	1.694454000	2.457645000	-0.603126000
1	2.040076000	4.143717000	-1.094860000

Protonation TS (**II$_p$**)

E = −1973.4280992
H = −1973.129540
G = −1973.230069
ῡ = −976.7 cm^{-1}

xyz

6	-2.129130000	0.149957000	-1.147110000
6	-3.457478000	0.618482000	-1.120307000
6	-4.473369000	-0.125347000	-1.710854000
6	-4.182766000	-1.344927000	-2.330619000
6	-2.869989000	-1.819223000	-2.356039000
6	-1.845103000	-1.078010000	-1.770408000
6	-1.062159000	0.921971000	-0.531232000
6	-0.837572000	1.954619000	0.157238000
16	-0.278263000	3.249687000	0.984010000
6	-0.287681000	2.658913000	2.737392000
8	1.288209000	-0.163727000	-0.857636000
16	2.033202000	-0.632365000	0.417188000
8	1.288369000	-0.298783000	1.640835000
8	3.460857000	-0.329356000	0.340844000
6	1.889786000	-2.482047000	0.244757000
9	2.449115000	-3.079055000	1.298198000
9	0.590621000	-2.829919000	0.190249000
9	2.490578000	-2.896747000	-0.870636000
6	3.714933000	1.985452000	-2.253117000
6	2.513722000	2.798733000	-2.080912000
7	1.560214000	3.445497000	-1.938957000
7	-3.051392000	0.560072000	2.953577000
6	-2.532290000	-0.444285000	2.689392000
6	-1.855287000	-1.692081000	2.347288000
1	-3.678995000	1.562396000	-0.632349000
1	-5.494405000	0.244638000	-1.685883000
1	-4.979094000	-1.923210000	-2.791012000
1	-2.641213000	-2.766649000	-2.835722000
1	-0.825313000	-1.447869000	-1.784907000

1	0.066312000	3.505873000	3.330468000
1	-1.298675000	2.373329000	3.029809000
1	0.398389000	1.814883000	2.820456000
1	4.602941000	2.625134000	-2.277590000
1	3.805571000	1.271817000	-1.427987000
1	3.658129000	1.427092000	-3.192860000
1	0.199530000	0.399327000	-0.649260000
1	-0.788145000	-1.500679000	2.196876000
1	-1.980917000	-2.424964000	3.150465000
1	-2.270920000	-2.104054000	1.422504000

Protonated intermediate (**III$_{\mathbf{p}}$**)

E = −1973.4347143
H = −1973.132169
G = −1973.228369

xyz

6	-3.512405000	0.445601000	-0.928717000
6	-4.528454000	-0.401446000	-1.355461000
6	-4.218289000	-1.581944000	-2.040372000
6	-2.886999000	-1.917943000	-2.294902000
6	-1.861216000	-1.073624000	-1.875479000
6	-2.169945000	0.116093000	-1.191991000
6	-1.059897000	0.966513000	-0.772107000
6	-0.986826000	2.029179000	-0.031176000
16	-0.680412000	3.317424000	0.858728000
6	-0.284281000	2.650468000	2.540156000
7	1.671458000	3.416368000	-1.591278000
6	2.594212000	2.756286000	-1.839293000
6	3.756371000	1.927093000	-2.144630000
8	1.497372000	-0.313327000	-1.145621000
16	2.045208000	-0.560487000	0.229508000
8	3.474541000	-0.246765000	0.380114000
6	1.939674000	-2.412880000	0.377381000
9	0.662713000	-2.816619000	0.195615000
8	1.149249000	-0.080643000	1.315789000
9	2.701635000	-3.014102000	-0.541251000
9	2.330272000	-2.814600000	1.593785000
1	-3.750370000	1.356061000	-0.387183000
1	-5.564046000	-0.144843000	-1.152438000
1	-5.016741000	-2.239808000	-2.372391000
1	-2.646312000	-2.837120000	-2.820927000
1	-0.820428000	-1.329526000	-2.049635000
1	0.163675000	3.486499000	3.082488000
1	-1.210145000	2.315033000	3.009210000

1	0.421181000	1.826339000	2.410045000
1	4.628017000	2.558806000	-2.343270000
1	3.961550000	1.258489000	-1.301701000
1	3.550383000	1.310800000	-3.024776000
1	-0.028864000	0.638019000	-1.074671000
7	-2.924733000	0.581990000	2.622778000
6	-2.343288000	-0.420937000	2.548439000
6	-1.577488000	-1.658001000	2.434385000
1	-0.542120000	-1.413911000	2.172954000
1	-1.599495000	-2.207272000	3.380763000
1	-1.997974000	-2.290026000	1.646263000

First acetonitrile addition TS (**IV$_p$**)

E = −1973.4164907
H = −1973.113678
G = −1973.209027
ῡ = −312.3 cm^{-1}

xyz

6	-1.785141000	1.744124000	-0.204049000
6	-3.015438000	2.119572000	-0.774819000
6	-3.590627000	3.343453000	-0.445207000
6	-2.949724000	4.206710000	0.448676000
6	-1.718019000	3.850601000	1.002424000
6	-1.129692000	2.633228000	0.670785000
6	-1.097569000	0.491372000	-0.494750000
6	-1.445346000	-0.703298000	-0.929059000
7	-3.405593000	-0.883513000	-0.763251000
6	-4.121230000	-1.479971000	-0.069967000
6	-4.983327000	-2.244599000	0.815604000
16	-0.882126000	-2.145721000	-1.529563000
6	-0.178190000	-1.677717000	-3.168965000
8	2.118988000	-0.429815000	-1.410432000
16	2.508348000	-0.351100000	0.017534000
8	2.929597000	-1.615739000	0.648219000
8	1.547311000	0.458368000	0.839301000
6	4.040512000	0.704462000	0.006849000
9	4.492336000	0.893663000	1.255606000
9	5.008296000	0.118775000	-0.711707000
9	3.780555000	1.905965000	-0.533272000
1	-3.503636000	1.465081000	-1.487218000
1	-4.536582000	3.631336000	-0.895597000
1	-3.403588000	5.161507000	0.700056000
1	-1.209665000	4.526061000	1.684383000
1	-0.162935000	2.351603000	1.078558000

1	0.110641000	-2.620465000	-3.641224000
1	-0.934545000	-1.173834000	-3.772717000
1	0.699293000	-1.057028000	-2.982085000
1	-4.336844000	-2.700129000	1.575683000
1	-5.715665000	-1.586952000	1.293753000
1	-5.506789000	-3.023921000	0.253469000
1	-0.017085000	0.480500000	-0.242075000
7	-1.996383000	-2.645447000	2.101487000
6	-0.903900000	-2.380121000	2.393906000
6	0.466614000	-2.033764000	2.754895000
1	0.658864000	-0.976639000	2.543105000
1	0.637981000	-2.240067000	3.816120000
1	1.183436000	-2.597639000	2.150332000

First acetonitrile addition intermediate ($\mathbf{V_p}$)

E = −1973.4483976
H = −1973.142696
G = −1973.236618

xyz

6	-3.120268000	-1.781109000	0.974671000
6	-3.613002000	-3.016968000	0.564519000
6	-2.761822000	-3.940139000	-0.049144000
6	-1.412742000	-3.627532000	-0.231567000
6	-0.906223000	-2.400711000	0.191489000
6	-1.764120000	-1.450650000	0.781561000
6	-1.196649000	-0.170511000	1.212831000
6	-1.784704000	1.049963000	1.116668000
16	-1.250024000	2.649637000	1.695498000
6	0.388255000	2.264928000	2.404622000
7	-2.164971000	1.537873000	-2.739430000
6	-1.020199000	1.396962000	-2.878605000
6	0.416720000	1.221487000	-3.045401000
8	1.900080000	-0.602348000	1.296057000
16	2.293616000	-0.130220000	-0.057393000
8	1.846322000	-1.004126000	-1.169356000
6	4.146864000	-0.298864000	-0.063732000
9	4.693567000	0.449547000	0.906870000
8	2.065357000	1.324178000	-0.277790000
9	4.506707000	-1.575440000	0.134041000
9	4.649476000	0.104430000	-1.241628000
1	-3.775770000	-1.091162000	1.499935000
1	-4.655978000	-3.268036000	0.738994000
1	-3.147013000	-4.904370000	-0.369695000
1	-0.745317000	-4.344262000	-0.701300000

1	0.139644000	-2.153341000	0.037952000
1	0.729545000	3.213498000	2.827900000
1	0.303482000	1.522684000	3.201498000
1	1.073134000	1.934349000	1.620269000
1	0.958886000	1.757514000	-2.258352000
1	0.690199000	0.167926000	-2.926835000
1	0.725608000	1.581822000	-4.031748000
1	-0.171785000	-0.199404000	1.584124000
7	-2.959133000	1.178128000	0.392060000
6	-3.841830000	1.366157000	-0.327434000
6	-4.942798000	1.566220000	-1.244623000
1	-5.595597000	0.686736000	-1.227036000
1	-5.516182000	2.451540000	-0.951275000
1	-4.508308000	1.697952000	-2.242513000

Second acetonitrile addition TS (**VI$_p$**)

E = −1973.4357521
H = −1973.131391
G = −1973.219696
ῡ = −288.7 cm^{-1}

xyz

6	1.781179000	1.384418000	0.776858000
6	3.143321000	1.698779000	0.950031000
6	3.621148000	2.973161000	0.652474000
6	2.749940000	3.957396000	0.178900000
6	1.393906000	3.662044000	0.020580000
6	0.906141000	2.393302000	0.325840000
6	1.214113000	0.063641000	1.083014000
6	1.813621000	-1.140704000	0.932516000
7	3.077453000	-1.253342000	0.334169000
6	3.590994000	-1.341503000	-0.744059000
6	4.911900000	-1.461003000	-1.380877000
16	1.235244000	-2.757782000	1.402884000
6	-0.323590000	-2.377436000	2.275837000
7	2.401549000	-1.244236000	-2.166202000
6	1.254485000	-1.117802000	-2.338166000
6	-0.147777000	-0.975896000	-2.644661000
8	-2.129104000	-1.331579000	-0.358245000
16	-2.370913000	0.111870000	-0.071382000
8	-1.884501000	1.031920000	-1.134350000
8	-2.023728000	0.521282000	1.309091000
6	-4.222732000	0.276860000	-0.150538000
9	-4.593202000	1.542237000	0.089716000
9	-4.671021000	-0.072826000	-1.366782000

9	-4.805940000	-0.516794000	0.759548000
1	3.821471000	0.956066000	1.360626000
1	4.672224000	3.203774000	0.806800000
1	3.124009000	4.951641000	-0.050479000
1	0.707427000	4.424339000	-0.337311000
1	-0.147246000	2.164495000	0.196124000
1	-0.662641000	-3.339782000	2.668630000
1	-0.140202000	-1.690477000	3.105151000
1	-1.065888000	-1.975695000	1.583743000
1	4.923015000	-2.331340000	-2.043075000
1	5.100166000	-0.570219000	-1.988078000
1	5.678097000	-1.560054000	-0.608578000
1	0.183046000	0.062646000	1.431269000
1	-0.488658000	0.049426000	-2.439622000
1	-0.323828000	-1.241945000	-3.692447000
1	-0.753059000	-1.603334000	-1.973359000

Second acetonitrile addition intermediate (**VII$_p$**)

E = −1973.4802336
H = −1973.172726
G = −1973.257651

xyz

6	-0.633250000	-3.171459000	1.102841000
6	0.322072000	-4.144552000	0.822402000
6	0.905189000	-4.210047000	-0.444602000
6	0.515586000	-3.289183000	-1.420240000
6	-0.432645000	-2.307724000	-1.139378000
6	-1.032065000	-2.225472000	0.135011000
6	-2.056222000	-1.250331000	0.522268000
6	-2.518852000	-0.166615000	-0.147027000
16	-3.878209000	0.857654000	0.436805000
6	-4.679564000	-0.188211000	1.697358000
7	-2.072868000	0.214283000	-1.420274000
6	-1.464115000	1.312774000	-1.656501000
6	-1.052280000	1.673483000	-3.057917000
8	2.309675000	1.543279000	2.021798000
16	2.261182000	1.456493000	0.570716000
8	0.696362000	1.304696000	0.046474000
6	2.739445000	-0.289026000	0.065388000
9	4.049747000	-0.398693000	0.281998000
8	2.964745000	2.406400000	-0.278872000
9	2.079906000	-1.161273000	0.808075000
9	2.477101000	-0.470973000	-1.222182000
1	-1.084730000	-3.135320000	2.092158000

1	0.608285000	-4.855338000	1.593663000
1	1.649583000	-4.969214000	-0.669540000
1	0.954540000	-3.334581000	-2.414198000
1	-0.729286000	-1.607620000	-1.910360000
1	-5.619745000	0.311969000	1.945129000
1	-4.893646000	-1.181516000	1.294350000
1	-4.077596000	-0.274321000	2.606004000
1	-1.511661000	2.622765000	-3.355319000
1	0.035387000	1.805355000	-3.111601000
1	-1.360876000	0.884473000	-3.745542000
1	-2.486637000	-1.436347000	1.501219000
7	-1.219807000	2.320872000	-0.706611000
6	-0.239698000	2.371509000	0.077027000
6	-0.021725000	3.459225000	1.081196000
1	0.054576000	3.040826000	2.089224000
1	0.906989000	4.002550000	0.872988000
1	-0.862300000	4.151114000	1.026512000

Pyrimidine scaffold formation TS (**VIII$_p$**)

E = −1973.4531322
H = −1973.147480
G = −1973.228810
ῡ = −353.2 cm^{-1}

xyz

6	2.654983000	-0.463307000	-0.664951000
6	2.967693000	-1.746403000	-1.160168000
6	4.234219000	-2.300228000	-0.992544000
6	5.231306000	-1.582508000	-0.329254000
6	4.943583000	-0.304457000	0.154815000
6	3.674976000	0.250203000	-0.005008000
6	1.293183000	0.044155000	-0.908030000
6	0.851517000	1.372820000	-0.780327000
7	1.222862000	2.219883000	0.193142000
6	1.116875000	1.852488000	1.462621000
7	0.640965000	0.673491000	1.861813000
6	0.229990000	-0.325485000	1.122905000
6	0.715271000	-1.724614000	1.388628000
16	-0.360509000	1.902335000	-1.959068000
6	-1.135622000	3.329135000	-1.125006000
6	1.567566000	2.807810000	2.530321000
8	-1.177098000	-0.207294000	0.782555000
16	-2.090279000	-1.440944000	0.202604000
8	-1.601916000	-1.889181000	-1.094885000
8	-2.403114000	-2.390391000	1.261144000

6	-3.610191000	-0.374691000	-0.069258000
9	-3.358506000	0.579458000	-0.959986000
9	-4.569731000	-1.176529000	-0.528516000
9	-3.989978000	0.170018000	1.081911000
1	2.202944000	-2.307714000	-1.692196000
1	4.443614000	-3.291222000	-1.386741000
1	6.221104000	-2.010438000	-0.196565000
1	5.715016000	0.270405000	0.660951000
1	3.481294000	1.252708000	0.358102000
1	-0.362692000	3.950242000	-0.669214000
1	-1.664894000	3.891721000	-1.897625000
1	-1.844275000	2.991238000	-0.365291000
1	2.477733000	2.425834000	3.007726000
1	1.772386000	3.789411000	2.100357000
1	0.803279000	2.888418000	3.309969000
1	0.724707000	-0.556845000	-1.615114000
1	0.205037000	-2.097416000	2.284719000
1	0.512089000	-2.407699000	0.562109000
1	1.787356000	-1.695630000	1.577894000

Pyrimidine intermediate (**IX$_{\mathbf{p}}$**)

E = −1973.5059429
H = −1973.197248
G = −1973.278357

xyz

6	0.181679000	-0.669886000	1.099784000
6	0.861322000	-0.195170000	-0.216308000
6	0.758185000	1.321768000	-0.254737000
7	0.733579000	2.056476000	0.802802000
6	0.757986000	1.381322000	2.041650000
7	0.499617000	0.136684000	2.251100000
6	2.300922000	-0.680471000	-0.385536000
6	2.551450000	-1.758284000	-1.246416000
6	3.846134000	-2.248143000	-1.418321000
6	4.912311000	-1.662950000	-0.734170000
6	4.674299000	-0.584285000	0.119097000
6	3.379182000	-0.095384000	0.291755000
16	0.690289000	2.034006000	-1.855633000
6	0.628825000	3.812746000	-1.453566000
6	1.091981000	2.269932000	3.204069000
8	-1.312328000	-0.391806000	0.930532000
16	-2.313117000	-1.324446000	0.081537000
8	-1.656574000	-1.891566000	-1.096398000
6	0.391576000	-2.135979000	1.440610000

8	-3.113535000	-2.161604000	0.961319000
6	-3.404504000	0.073601000	-0.513803000
9	-2.687389000	0.923793000	-1.249481000
9	-4.372637000	-0.454488000	-1.262823000
9	-3.938265000	0.715570000	0.521368000
1	1.724730000	-2.216343000	-1.783732000
1	4.020015000	-3.084080000	-2.090350000
1	5.922005000	-2.041171000	-0.868234000
1	5.498831000	-0.117969000	0.651567000
1	3.213977000	0.751574000	0.950182000
1	0.619423000	4.339955000	-2.410317000
1	-0.274815000	4.041394000	-0.886800000
1	1.506023000	4.098864000	-0.871176000
1	1.115781000	1.693784000	4.129948000
1	2.059248000	2.760378000	3.041127000
1	0.344090000	3.067784000	3.282345000
1	0.281954000	-0.610494000	-1.043690000
1	-0.228638000	-2.387039000	2.304366000
1	0.147294000	-2.791517000	0.601443000
1	1.438980000	-2.287168000	1.711688000

Final N-protonated product ($\mathbf{X_p}$)

E = −1973.5660549
H = −1973.257321
G = −1973.341354

xyz

6	-0.709864000	-0.465823000	-0.603925000
6	-1.972518000	-0.005435000	-0.239555000
6	-2.105371000	1.397704000	-0.067718000
7	-1.090077000	2.259542000	-0.244310000
6	0.091507000	1.769667000	-0.586323000
7	0.285322000	0.447679000	-0.766729000
6	-3.122748000	-0.931175000	-0.019064000
6	-3.225061000	-1.659711000	1.175553000
6	-4.298386000	-2.526185000	1.381516000
6	-5.279158000	-2.672590000	0.398783000
6	-5.184771000	-1.950230000	-0.792031000
6	-4.112920000	-1.082546000	-1.001299000
16	-3.667768000	2.047583000	0.412429000
6	-3.296438000	3.831339000	0.542661000
6	1.261593000	2.682587000	-0.786511000
8	2.745546000	-0.246226000	-1.318796000
16	3.380876000	-1.089883000	-0.229846000
8	2.401306000	-1.444140000	0.822021000

6	-0.335935000	-1.896261000	-0.831631000
8	4.270003000	-2.140143000	-0.726675000
6	4.502233000	0.141780000	0.601199000
9	3.783060000	1.190581000	1.068526000
9	5.132872000	-0.415986000	1.639040000
9	5.413984000	0.617018000	-0.254372000
1	-2.462147000	-1.546084000	1.940894000
1	-4.366491000	-3.086745000	2.309423000
1	-6.113993000	-3.348836000	0.559898000
1	-5.943635000	-2.063216000	-1.561072000
1	-4.039013000	-0.523197000	-1.929930000
1	-4.236150000	4.297770000	0.848532000
1	-2.526763000	4.012156000	1.294049000
1	-2.974380000	4.232619000	-0.418915000
1	1.598761000	2.646820000	-1.828650000
1	0.971343000	3.702306000	-0.532835000
1	2.107434000	2.363822000	-0.170532000
1	1.286733000	0.108782000	-0.998130000
1	0.106212000	-2.013229000	-1.827795000
1	0.439260000	-2.183799000	-0.109789000
1	-1.200678000	-2.552082000	-0.740272000

Supplementary References

[i] Bruker SAINT V7.68A & V8.32B Copyright © 2005-2015 Bruker AXS

[ii] Sheldrick, G. M. (1996). *SADABS.* University of Göttingen, Germany.

[iii] Sheldrick, G.M. (2008). Acta Cryst. A64, 112-122

[iv] Sheldrick, G.M. (2008). Acta Cryst. A64, 112-122

[v] Dolomanov, O.V., Bourhis, L.J., Gildea, R.J, Howard, J.A.K. & Puschmann, H. , OLEX2, (2009), J. Appl. Cryst. 42, 339-341

[vi] C. B. Huebschle, G. M. Sheldrick and B. Dittrich, ShelXle: a Qt graphical user interface for SHELXL, J. Appl. Cryst., 44, (2011) 1281-1284

[vii] Dolomanov, O.V., Bourhis, L.J., Gildea, R.J, Howard, J.A.K. & Puschmann, H. , OLEX2, (2009), J. Appl. Cryst. 42, 339-341

[viii] a) Becke, A. D. (1988). Phys. Rev. A 38, 3098–3100. b) Lee, C.; Yang, W.; Parr, R. G. (1988). Phys. Rev. B 37, 785–789. c) Becke, A. D. (1993). J. Chem. Phys. 98, 5648–5652.

[ix] Frisch, M. J.; Trucks, G. W.; Schlegel, H. B.; Scuseria, G. E.; Robb, M. A.; Cheeseman, J. R.; Scalmani, G.; Barone, V.; Mennucci, B.; Petersson, G. A.; Nakatsuji, H.; Caricato, M.; Li, X.; Hratchian, H. P.; Izmaylov, A. F.; Bloino, J.; Zheng, G.; Sonnenberg, J. L.; Hada, M.; Ehara, M.; Toyota, K.; Fukuda, R.; Hasegawa, J.; Ishida, M.; Nakajima, T.; Honda, Y.; Kitao, O.; Nakai, H.; Vreven, T.; Montgomery, Jr., J. A.; Peralta, J. E.; Ogliaro, F.; Bearpark, M.; Heyd, J. J.; Brothers, E.; Kudin, K. N.; Staroverov, V. N.; Keith, T.; Kobayashi, R.; Normand, J.; Raghavachari, K.; Rendell, A.; Burant, J. C.; Iyengar, S. S.; Tomasi, J.; Cossi, M.; Rega, N.; Millam, N. J.; Klene, M.; Knox, J. E.; Cross, J. B.; Bakken, V.; Adamo, C.; Jaramillo, J.; Gomperts, R.; Stratmann, R. E.; Yazyev, O.; Austin, A. J.; Cammi, R.; Pomelli, C.; Ochterski, J. W.; Martin, R. L.; Morokuma, K.; Zakrzewski, V. G.; Voth, G. A.; Salvador, P.; Dannenberg, J.

J.; Dapprich, S.; Daniels, A. D.; Farkas, O.; Foresman, J. B.; Ortiz, J. V.; Cioslowski, J.; Fox, D. J.; Gaussian 09, revision D.01. Gaussian, Inc., Wallingford CT (2013).

[x] Hariharan, P. C.; Pople, J. A. (1974). *Mol. Phys.* 27, 209−214.

[xi] http://www.chemcraftprog.com
